# Photoswitching neutral homoaromatic hydrocarbons

**DOI:** 10.1038/s41557-022-01121-w

**Published:** 2023-01-26

**Authors:** Trung Tran Ngoc, Niklas Grabicki, Elisabeth Irran, Oliver Dumele, Johannes F. Teichert

**Affiliations:** 1 Technische Universität Chemnitz, Institut für Chemie, Fakultät für Naturwissenschaften, Chemnitz, Germany; 2 Technische Universität Berlin, Institut für Chemie, Berlin, Germany; 3 Department of Chemistry, Humboldt Universität zu Berlin and IRIS Adlershof, Berlin, Germany

**Keywords:** Organic chemistry, Photochemistry, Computational chemistry, Synthetic chemistry methodology

## Abstract

Homoaromatic compounds possess an interrupted *π* system but display aromatic properties due to through-space or through-bond interactions. Stable neutral homoaromatic hydrocarbons have remained rare and are typically unstable. Here we present the preparation of a class of stable neutral homoaromatic molecules, supported by experimental evidence (ring current observed by NMR spectroscopy and equalization of bond lengths by X-ray structure analysis) and computational analysis via nucleus-independent chemical shifts (NICS) and anisotropy of the induced current density (ACID). We also show that one homoaromatic hydrocarbon is a photoswitch through a reversible photochemical [1, 11] sigmatropic rearrangement. Our computational analysis suggests that, upon photoswitching, the nature of the homoaromatic state changes in its perimeter from a more pronounced local 6*π* homoaromatic state to a global 10*π* homoaromatic state. These demonstrations of stable and accessible homoaromatic neutral hydrocarbons and their photoswitching behaviour provide new understanding and insights into the study of homoconjugative interactions in organic molecules, and for the design of new responsive molecular materials.

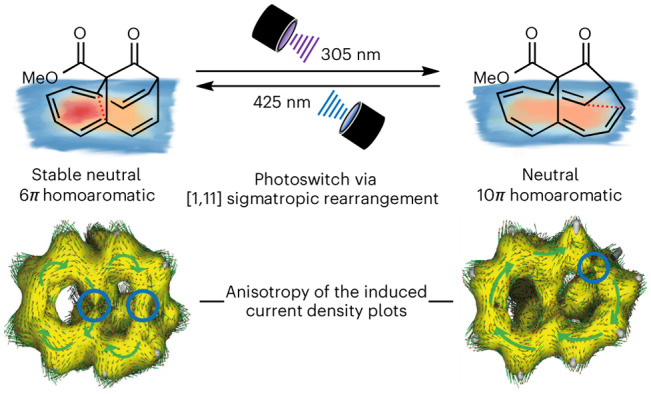

## Abstract (web summary)

Neutral homoaromatic hydrocarbons—which possess an interrupted *π*-system yet display aromatic properties owing to through-space or through-bond interactions—have remained rare as they are typically unstable. Now a class of stable neutral homoaromatic homoannulenes has been synthesized, including one that acts as a photoswitch through a reversible [1, 11] sigmatropic rearrangement.

## Main

Homoaromatic molecules display characteristics of aromatic stabilization yet possess an interrupted cyclic conjugated *π* system^1^. The concept of homoaromaticity, resting on through-bond or through-space homoconjugation^2,3^ (the interaction between disconnected *π* systems, usually interrupted by one or more *sp*^3^-hybridized carbon atoms or cyclopropane units^1,4^), has been highly controversial in the past^1^. The difficulty in identifying clear criteria for the assignment of molecules as homoaromatic^1,5^, as well as the small number of known homoaromatic molecules, has spurred discussions in this field.

The generally accepted criteria^1,4^ for homoaromaticity are as follows: (1) one or more homoconjugative interactions (through-space or through-bond), (2) electron delocalization of (4*n* + 2) *π* electrons in a cyclic structure due to effective overlap of *π* orbitals, (3) bond equalization of single and/or double bonds, (4) magnetic characteristics associated with ‘normal’ aromaticity and (5) resonance stabilizing energy. It should be noted that these criteria have to be regarded as a whole, and structural changes such as consideration of bond lengths alone (which some researchers challenge as a criterion for or against aromaticity^6–8^), without investigation of the magnetic properties of a compound, do not suffice for analysis of homoaromaticity^9,10^. The term ‘equalization’ is somewhat misleading, as, according to this term, only the shortening of single bonds and elongation of double bonds is referenced. However, in homoaromatic compounds, the single and double bond lengths rarely converge to exactly the same length.

**Fig. 1:**
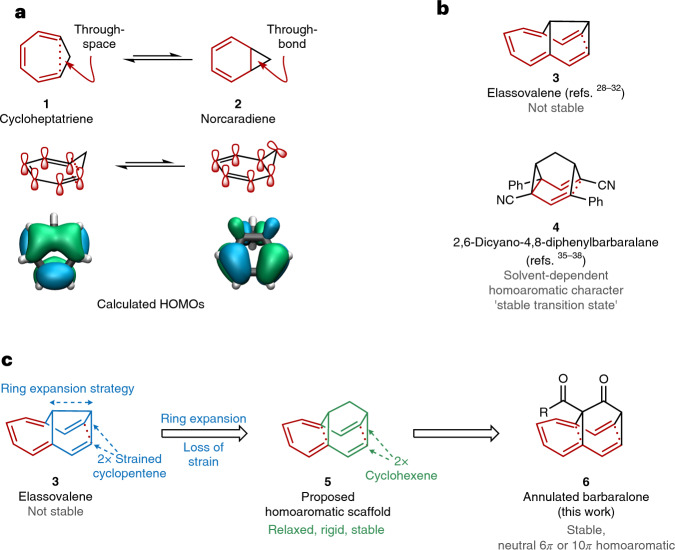
Examples of proposed neutral homoaromatic compounds, and design strategy for annulated barbaralones as neutral homoaromatics. **a**, 1,3,5-Cycloheptatriene and norcaradiene as prototypical homoaromatic compounds. Top, structures; middle, depiction of simplified homoconjugative interactions^1,3,22,24–27^; bottom, calculated HOMOs. **b**, Previously studied candidates for neutral homoaromatics: bridged hydrocarbons as potential neutral homoaromatics^28,30–32,35–38^. **c**, This work: design principles for homoaromatic hydrocarbons, and the conceptual evolution of annulated barbaralones as neutral homoaromatics (R = alkoxy group). For calculations of the HOMOs of **1** and **2**, the B3LYP/def2-TZVP level of theory was used. From the view from the bottom, subtle structural changes in the hydrocarbon backbone are seen that have a substantial impact on the strength of the homoconjugative interaction (see a comparison of the strain distribution in **3**, **5** and **6** in Supplementary Section 1.8.5).

Several ionic homoaromatic compounds have been disclosed^11–13^. However, examples for neutral homoaromatic molecules (especially purely organic ones) remain extremely rare^14–22^ and have generally been heteroatom-containing molecules^14–16,20^. There is only limited experimental and computational evidence of neutral homoaromatic hydrocarbons, spurring the comment in the leading review that ‘there remains a dearth of neutral homoaromatics’^1^. A good example of such a controversial discussion about homoaromatic character in neutral organic compounds relates to the formal ‘homobenzene’ 1,3,5-cycloheptatriene (**1**) and its valence tautomer norcaradiene (**2**). In **1**, interactions between the terminal *π* orbitals of the triene moiety account for the through-space homoconjugation (Fig. 1a). For an explanation of through-bond (cyclopropyl) homoconjugation in **2**, the Walsh orbitals of the cyclopropane moiety are generally considered^23^. Both types of homoconjugation can be visualized via the calculated highest occupied molecular orbitals (HOMOs) of **1** and **2** (Fig. 1a and Supplementary Fig. 40). For the through-space homoconjugation in **1**, a bridging lobe of the HOMO between the terminal carbon atoms of the *π* system can be observed, whereas typical Walsh orbitals are obtained for the cyclopropanyl moiety of **2**. A large number of theoretical studies, including some experimental evidence in support of and against a potential homoaromatic character of **1** or **2**, are available^1,3,22,24–27^, with the most recent investigations supporting a homoaromatic character in **1** (refs. ^22,27^). Underlining the controversy, one of these studies refers to **1** and **2** as bearing the ‘unique distinction of being the subject of the highest number of claims and counter-claims about its homoaromatic stabilization’^27^.

Similarly, elassovalene (**3**)^28,29^, a potential neutral 10*π*-electron homoaromatic, has been the topic of long-standing debate. After independent preparation of the generally unstable **3** by the groups of Paquette and Vogel, evaluation of the spectral data and magnetic criteria, and finally comparison to the related aromatic annulenes, it was concluded that elassovalene **3** is not a neutral homoaromatic hydrocarbon, based on ^1^H NMR analysis^28,30–34^. The group of Quast came close to isolating a homoaromatic hydrocarbon when they found that in barbaralane derivative **4**, the homoaromatic transition state is markedly stabilized in select polar solvents^35–38^. However, in the crystal structure of **4**, only limited bond-length equalization was observed (Fig. 4, below), rendering this molecule the closest candidate for a neutral homoaromatic hydrocarbon so far.

The above-mentioned examples underscore the requirement for combined computational and experimental evidence for the assignment of a homoaromatic electronic structure. However, structural information is difficult to access, because neutral homoaromatics are often transient species^1,18,19,37^ and not crystalline, hampering the identification of bond-length equalization as one of the key criteria for homoaromaticity^6–8^. In this Article we present the synthesis of a class of stable neutral homoaromatic hydrocarbons, homoannulenes **6**, and discuss their homoaromaticity, supported by experimental data (chemical shifts from NMR spectroscopy and bond-length comparison from X-ray structure elucidation) and calculations. We further show that compounds **6** act as a structurally novel photochemical switch through an unprecedented reversible photoinduced [1, 11] sigmatropic rearrangement, forming a novel neutral 10*π* homoaromatic.

## Results and discussion

### Synthesis of stable neutral homoaromatics

Our working hypothesis for the design of stable and isolable neutral homoaromatics originates from elassovalene **3** (Fig. 1c). Extending the bridging unit from a semibullvalene to a barbaralane core, as in **5** (which has been judged not to be homoaromatic based on circumstantial evidence^28,32^), we anticipated that an additional carbon atom in the bridge would create less ring strain compared to elassovalene **3**. This would avoid fast degradation (see the strain energy analysis in Supplementary Discussion Section 1.8.5) and would also facilitate the interaction of both ends of the *π* system through space to allow for a homoconjugative interaction^39,40^. At the same time, the target molecule would in principle bear both a potential 6*π* as well as a 10*π* homoaromatic system with enhanced homoaromatic interactions. To the best of our knowledge, no 10*π* homoaromatic has been reported previously in the literature, which makes the presented example the largest homoaromatic *π* system that has been characterized^1^.

**Fig. 2:**
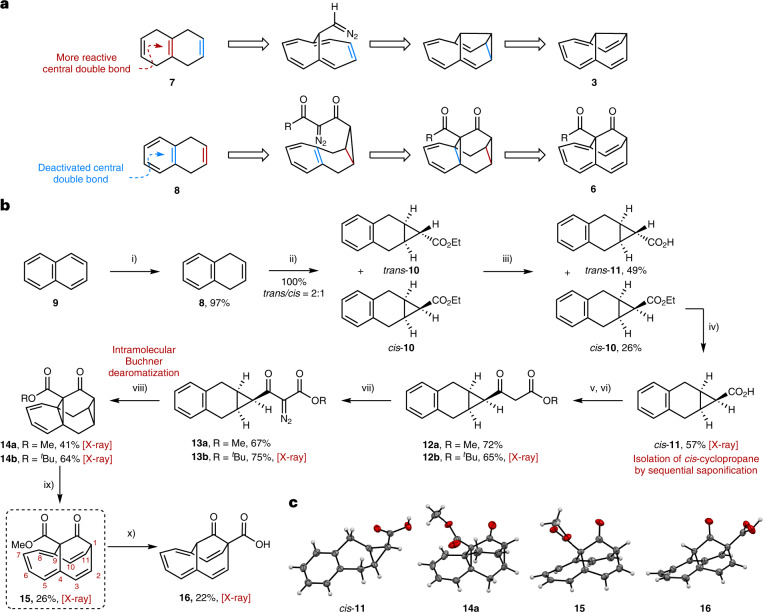
Synthesis of homoannulene ester 15. **a**, Synthetic strategies. Colours indicate the first targeted C=C double bond (red) and the second targeted C=C double bond (blue)^28^. Top: previous approach to elassovalene^28^: double cyclopropanation (inside → outside). Bottom: this work, comprising cyclopropanation and Buchner dearomatization (outside → inside). **b**, Synthesis. Key steps in the synthesis involve a temperature sequential separation of diastereomers as well as a copper-catalysed intramolecular Buchner reaction^33^ to construct the key hydrocarbon framework. Conditions: (i) 2.5 equiv. Na, 2.5 equiv. ^*t*^BuOH, Et_2_O, 21 °C, 14 h; (ii) 1.5 equiv. ethyl diazoacetate, 1.0 mol% Rh_2_(OAc)_4_, CH_2_Cl_2_, 21 °C, 20 h; (iii) 3.0 equiv. KOH, EtOH, 21 °C, 8 h; (iv) 3.0 equiv KOH, EtOH, reflux, 3 h; (v) 1.0 equiv. carbonyldiimidazol, THF, 21 °C, 1 h; (vi) 2.5 equiv. AcOMe or AcO^*t*^Bu, 5.0 equiv. lithium diisopropyl amide, THF, −78 to 21 °C, 3 h; (vii) 1.1 equiv. 4-acetamidobenzenesulfonyl azide, 1.1 equiv. Et_3_N, MeCN, 0 to 21 °C, 3 h; (viii) 10 mol% Cu(hFacac)_2_·*x*H_2_O, C_6_H_5_Cl, reflux, 3 h; (ix) 2.0 equiv. DDQ, C_6_H_5_Cl, 130 °C, 4 h; (x) 4.0 equiv. KOH, MeOH/H_2_O (1:1), 21 °C, 27 h. DDQ, 2,3-dichloro-5,6-dicyano-1,4-benzoquinone; hFacac, hexafluoroacetylacetonate. **c**, Depiction of X-ray structures of selected synthetic intermediates. Several homoaromatic compounds can be accessed by the synthesis, and key structural data of these stable compounds can be obtained from both solution-phase as well as solid-phase investigations.

We targeted the synthesis of a potential 6*π*- or 10*π*-electron homoaromatic homoannulene **6** with a barbaralone-derived framework (Fig. 2). For the related bridged aromatic annulenes^41–43^, semibullvalenes^28,43^ and elassovalene^28^ **3** (Fig. 2a), the synthesis starts from tetrahydronaphthalenes **7** or hexahydroanthracenes, respectively, using the central alkene(s) of hydrogenated polyaromatic compounds such as tetrahydronaphthalene as the synthetic linchpin for a key cyclopropanation (Fig. 2a, inside → outside). We envisioned an inverse approach (outside → inside), utilizing first the outer disubstituted alkene of the related dihydronaphthalene **8**, leaving the benzene ring untouched. In this manner, we could circumvent the inherent higher reactivity of the central tetrasubstituted alkene for the key cyclopropanation. This approach requires a challenging intramolecular dearomative Buchner reaction as the central methodology downstream in the synthesis (Fig. 2b)^33^.

In this vein, we first reduced naphthalene **9** to 1,4-dihydronaphthalene **8**, making the outer double bond accessible for the subsequent rhodium(II)-catalysed cyclopropanation^44^, giving cyclopropane **10** as a diastereomeric mixture of *trans*-**10** and *cis*-**10** (2:1). Separation of the two isomers, *trans*-**10** and *cis*-**10**, was achieved by iterative temperature-dependent saponification with KOH, exploiting the innate reactivity difference of the diastereomeric cyclopropyl esters *trans*-**10** and *cis*-**10**. A first saponification with KOH at room temperature selectively transformed *trans*-**10** to the *trans*-**11** acid and thus facilitated removal of unwanted *trans* isomer. Raising the temperature from room temperature to reflux afforded the *cis-*carboxylic acid *cis*-**11** through subsequent saponification at elevated temperature. In *cis*-**11**, the carboxylic acid is aligned suitably towards the benzene ring, a key necessity for the downstream intramolecular Buchner reaction. It should be noted that this particular Buchner reaction is impeded by the fact that acetoacetate-derived diazo compounds generally display significantly lower reactivity in cyclopropanation reactions^33^. We continued by activation of *all-cis*-**11** and chain elongation by Claisen condensation to form acetoacetate **12**. A subsequent diazo transfer reaction led to the corresponding diazo compounds **13**. The following intramolecular Buchner reaction, vital for our outside → inside strategy, required extensive catalyst optimization. After investigation of a variety of Rh(II) and Cu(II) complexes, we identified Cu(hFacac)_2_ as a suitable catalyst for the desired Buchner dearomatization to give triasteranes **14** (for the optimization see Supplementary Information). Only a few of the catalysts employed gave the desired product **14** at all, whereas the Buchner reaction catalysed by Cu(hFacac)_2_ (hFacac, hexafluoroacetylacetonate) did not show any traces of otherwise competitive C–H insertions. Finally, dehydrogenative oxidation using DDQ (2,3-dichloro-5,6-dicyano-1,4-benzoquinone) gave homoannulene ester **15**. To support the connectivity and stereochemistry of the desired products, crystal structures were obtained for **11**, **12b**–**14a**,**b** and **15**. This allowed the analysis of bond lengths to predict any potential homoaromaticity (see section ‘Bond-length comparison as structural evidence for homoaromaticity’).

When exploring the general chemical reactivity of **15**, we found that saponification conditions unexpectedly led to the formation of the rearranged homoannulene carboxylic acid **16** (a proposed mechanism is outlined in Supplementary Fig. 5). This turned out to be a fortunate discovery, as **16** not only represents yet another neutral homoaromatic compound, but, with the X-ray crystal structure of **16** available, it also serves as key reference compound for the following investigation of the magnetic properties of the neutral homoaromatics **15** and **16**.

### Magnetic characteristics of neutral homoaromatics

**Fig. 3:**
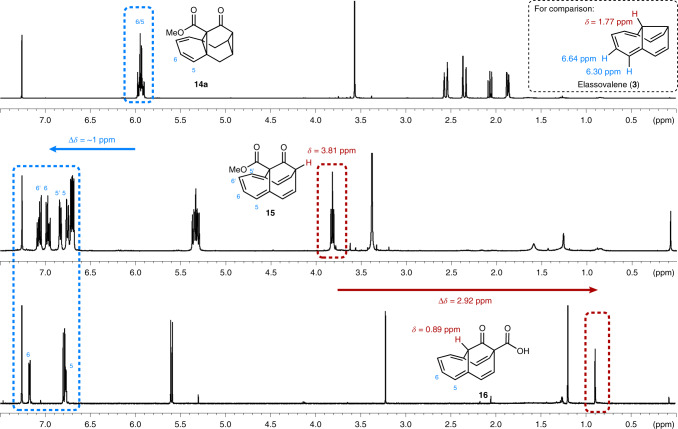
NMR spectroscopy as an indicator for homoaromatic interactions. Comparison of selected ^1^H NMR chemical shifts of triasterane **14a**, homoannulene ester **15** and annulene carboxylic acid **16** as an indication of homoaromatic interactions (400 MHz, CDCl_3_, 298 K). Shown are a significant downfield shift of hydrogen atoms attached to the homoaromatic scaffold (labelled blue) and an upfield shift of the hydrogen atom placed within the ring current of the homoaromatic scaffold (labelled red). Also shown are a comparison to neutral compound **3**, whose homoaromatic character has been refuted, and characterization data from the literature^28,31^.

Homoannulene ester **15** and the rearranged homoannulene carboxylic acid **16** turned out to be ideal probes for the investigation of magnetic properties (induced ring current) indicative of a homoaromatic interaction^1^. The magnetic anisotropy and susceptibility of homoaromatic compounds are verifiable by NMR spectroscopy via marked chemical shifts^1,9,45–48^. Comparing the ^1^H NMR spectra of **14a**, **15** and **16** (Fig. 3), we observed that **15** and **16** display a significant downfield shift of hydrogen atoms 5 and 6 of ~1.00 ppm as compared to **14a**, giving a first indication of the presence of a ring current in the former two. Furthermore, the α-carbonyl methine group of homoannulene ester **15**, facing away from the potential homoaromatic system, displays an expected chemical shift of *δ* = 3.81 ppm (Fig. 3, middle). In stark contrast, the α-carbonyl methine ^1^H NMR resonance of homoannulene carboxylic acid **16**, placed directly above the homoaromatic system, is significantly shifted upfield to *δ* = 0.89 ppm, despite being placed directly adjacent an electron-withdrawing ketone (Fig. 3, bottom). Even taking the anisotropic effect of the double bonds into account^24^, this unusually high upfield shift of Δ*δ* = 2.92 ppm unambiguously indicates the presence of a diamagnetic ring current^47,49^, which shields the methine hydrogen atom of **16**^24,41^. Both key spectral properties (downfield shift of hydrogen atoms 5 and 6 and the shift of the bridgehead methine hydrogen atom) are missing in elassovalene (**3**)^28,31^, whose analogous methine hydrogen atom appears at *δ* = 1.77 ppm (and C_*sp*2_–H appear at 6.30 and 6.64 ppm, respectively). Homoannulene ester **15** and homoannulene carboxylic acid **16** possess an interrupted cyclic *π* system. The observation of a strong ring current therefore indicates the existence of through-space interaction of the *π* system, leading to a delocalization of *π* electrons in **15** and **16** (ref. ^2^) (calculations of the magnetic properties of **15**, which support the experimental data, are shown in section ‘Computational investigation of photoswitchable homoaromatics’). Therefore, the fused barbaralone framework prepared in this study seems to structurally enable a more efficient homoconjugation by an elongated bridge that facilitates the through-space interaction of carbon atoms C(4)···C(9) through steric congestion. Combined, these findings serve as an important indication that homoannulene ester **15** and homoannulene carboxylic acid **16** are neutral homoaromatic compounds.

### Bond-length comparison as structural evidence for homoaromaticity

**Fig. 4:**
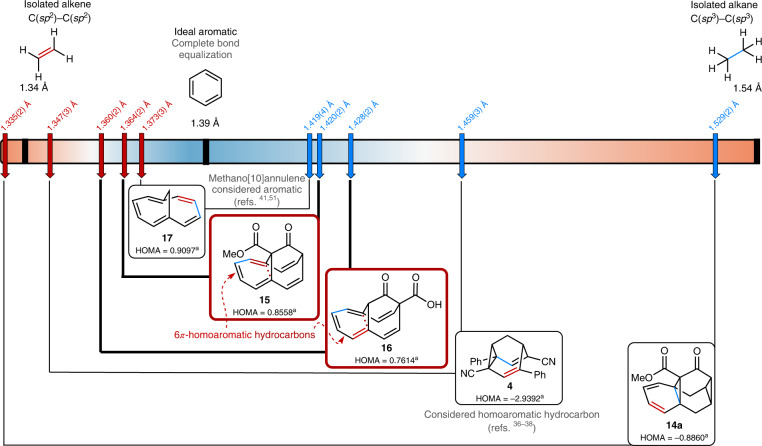
Comparison of the bond lengths of selected (potential) homoaromatics from X-ray diffraction. The shortest double bond is indicated by red arrows and the longest single bond by blue arrows for the (homo)aromatic part of methano[10]annulene (**17**)^41,51^, barbaralane **4** (refs. ^36–38^), triasterane **14a**, homoannulene ester **15** and homoannulene carboxylic acid **16**(refs. ^6–8^). ^a^Please note that in **4** and **14a**, a potential homoaromaticity relies on through-bond homoconjugation, whereas **15**–**17** hinge on through-space homoconjugation. Therefore, the HOMA values of **4** and **14a** differ significantly from those of **15**–**17**. Supplementary Discussion Section 1.4 provides calculations and further discussion. Compounds **15** and **16** display significant bond-length equalization, reaching values associated with aromatic (cyclic conjugated) molecules.

One of the paramount characteristics for homoaromatic compounds is bond-length equalization of the single and double bonds involved^1,2,6–8^. With the X-ray structures of **14a**,**b**, **15** and **16** available for detailed analysis, we observed significant shortening of the single bonds and elongation of the double bonds (Fig. 4). As limits, the bond lengths of isolated C–C single (1.54 Å) and C=C double bonds (1.34 Å), as well as the ‘ideal’ bond-length equalization of benzene (1.39 Å), are employed for comparison^50^. These values are compared to the longest C–C single bond and the shortest C=C double bond, respectively, of the respective neutral homoaromatic molecules **14a**, **15** and **16** (see Supplementary Fig. 3 for a detailed analysis of all bond lengths).

For homoannulene ester **15**, a homoaromatic cycloheptatrienyl fragment seems to be present, as indicated by the bond-length equalization (the shortest double bond is 1.364(2) Å (Fig. 4, red arrows) and the longest single bond is 1.420(2) Å (Fig. 4, blue arrows)). The formal vinyl substituents in the barbaralone substructure show alternating (single and double) bond lengths (~1.45 and ~1.34 Å, respectively), indicating a 6*π* homoaromatic compound with two vinyl substituents. Also, for homoannulene carboxylic acid **16**, equalized bond lengths are observed (1.428(2) and 1.360(2) Å for the longest C–C single and shortest C=C double bond in the cycloheptatriene moiety). In contrast, non-oxidized triasterane **14a** displays the expected bond-length alternation for a 1,3-diene and lacks any indication for homoconjugation. We compared our observations to barbaralane **4** (refs. ^36–38^) (Fig. 4), which to our knowledge is considered the example for a neutral homoaromatic compound (1.459(3) and 1.347(3) Å for the longest C–C single and shortest C=C double bond) closest to exhibiting homoaromatic character in terms of bond lengths^1^. Homoannulene ester **15** and carboxylic acid **16** show significantly higher bond-length equalization than barbaralane **4**, as reflected in the harmonic oscillator model of aromaticity (HOMA) values, which are used for the description of bond-length equilibration. Indeed, the bond-length equalization observed for **15** and **16** is rather comparable to that of methano[10]annulene **17** (ref. ^41^) (1.373(3)–1.419(4) Å (ref. ^51^)), which is considered aromatic^41,52^. The bond-length equalization of **15** and **16**, in addition to the ring current observation discussed above, lead us the conclusion that **15** and **16** are indeed neutral and stable homoaromatic compounds.

### Photoswitching behaviour of stable neutral homoaromatics

**Fig. 5:**
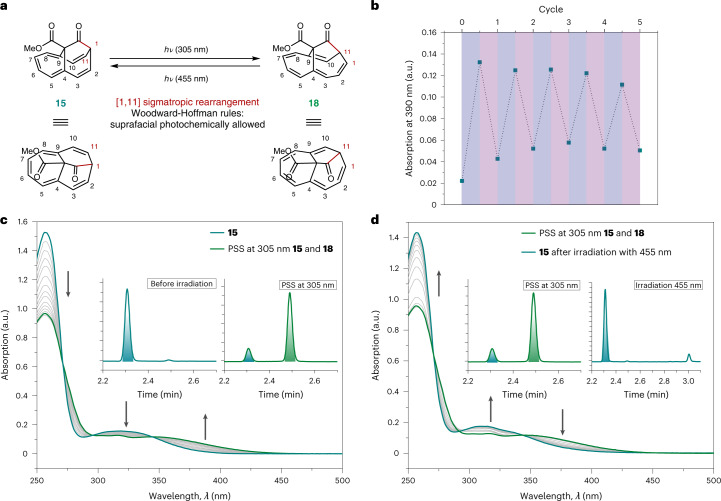
Photoswitchable behaviour of homannulene 15. **a**, Homoannulene ester **15** undergoes reversible [1, 11] sigmatropic rearrangement^54^ at *λ* = 305 nm to ester **18** (only one possible enantiomer is displayed) and back-reaction to **15** at *λ* = 455 nm. **b**, Cycling experiments of the photoreaction. Absorbance at 390 nm shown under alternating irradiation between 305 nm (blue shaded) and 455 nm (red shaded). The change of the absorption intensity indicates a moderate switching fatigue. **c**, UV–vis absorption of **15** (cyan) in cyclohexane (*c*(**15**) = 5.5 × 10^−5^ mol l^−1^) upon irradiation with a 305-nm LED (grey, every 5 s, 80 s total), until PSS_305 nm_ is reached (green). **d**, Back-irradiation into **15** using a 455-nm LED (grey, spectra recorded every 60 s, 11 min total). Insets: HPLC chromatograms at the start and end of the irradiation detected at 350-nm UV–vis absorption. The switching of **15** into **18** works up to a ratio of 16:84 at PSS_305 nm_. Quantitative back-irradiation of **18** is possible at *λ* = 455 nm. Note that the depiction of **18** is just one of the two possible enantiomers formed in the rearrangement. Source data

Extended conjugated *π* systems—especially aromatic ones—express particular reactivity and show the ability to absorb energy in the ultraviolet visible (UV–vis) range. To further elucidate the homoaromatic character of **15**, we investigated its behaviour upon irradiation with light, as has been done for several other homoaromatic compounds^28,35,36,53^. We found that homoannulene ester **15** undergoes a photoinduced rearrangement of the barbaralone framework, forming ester **18** (305 nm light-emitting diode (LED) irradiation for 80 s of a dilute cyclohexane or acetonitrile (MeCN) solution of **15**; Fig. 5a). The thermal stability of photoproduct **18** was assured by heating the solution at the photostationary state (PSS) at 305 nm in MeCN at 55 °C for 9.25 h (Supplementary Figs. 17 and 18). Thermal stability allowed the separation of the two compounds **15** and **18**, present in the PSS, using preparative HPLC. Full characterization of **18** using NMR spectroscopy and high-resolution mass spectrometry analysis allowed the unambiguous determination of its structure. The formation of **18** can be rationalized by an unprecedented [1, 11] sigmatropic rearrangement from **15** to **18**, which is suprafacially allowed under photochemical conditions in accordance with Woodward–Hoffmann rules^54^. The defined isosbestic points in the UV–vis spectra indicate a distinct photochemical rearrangement, supported by HPLC (Fig. 5b,c). Integration of the peak areas of the UV–vis/HPLC traces revealed a **15**/**18** ratio of 16:84 at the PSS (Supplementary Fig. 14). This photochemical reaction was found to be independent of solvent polarity. It was observed that it occurs at equally rapid rates in both cyclohexane and in MeCN (PSS composition of **15**/**18** of 20:80), which indicates a non-polar transition state as typically observed for pericyclic reactions (Supplementary Figs. 8–12).

Photochromic systems are of special interest in the development of functional materials^55^. The prediction and rational design of molecules bearing new motifs that can be reversibly switched from one state into another by an external stimulus is challenging. Attempting the back-reaction of **18** to **15**, we found that the photochemical [1, 11] sigmatropic rearrangement is reversible upon irradiation with a 455-nm LED light source. This renders homoannulene ester **15** a structurally novel photochemical switch^56–58^. The photochromic system can be classified as a *p*-type photoswitch (back-reaction photochemically activated).

We performed multiple cycles of irradiation by alternating between 305-nm and 455-nm LEDs (Fig. 5b). Although the irradiation repeatedly led to the formation of **18** in the PSS and back-formation of **15**, analysis of the UV–vis absorption maxima at 390 nm over several cycles indicated a moderate switching fatigue caused by the formation of trace side products during the photoswitching process (Supplementary Fig. 15). We hypothesized that the formation of rearranged **18** is driven by a homoaromatic stabilization effect and thus turned our attention to the computational electronic and magnetic structure analysis of **15** and **18**.

### Computational investigation of photoswitchable homoaromatics

**Fig. 6:**
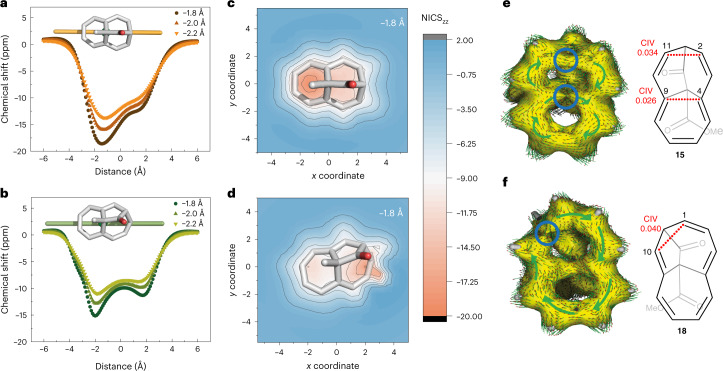
Computational evaluation of the homoaromaticity of Me-15 and Me-18 using NICS^59–64^ scans and ACID plots^65,66^. **a**,**b**, NICS_ZZ_-XY^61,63^ scan below the molecular surface, as indicated by the orange line below Me-**15** and by the green line below Me-**18** at distances of 1.8 Å, 2.0 Å, 2.2 Å for Me-**15** (**a**) and Me-**18** (**b**). **c**,**d**, NICS_ZZ_-XY heat map 1.8 Å below the molecular plane of Me-**15** (**c**) and Me-**18** (**d**) (the region of the red area in **d** originates from the close proximity of the carbon skeleton near C(1,2) to the NICS probe. The scale of the colour code is in ppm. These graphs give the same result as **a** and **b** but for the whole surface below the shown molecules. **e**, ACID plot of Me-**15** at an isosurface value of 0.026. Right: structure of **15**. **f**, ACID plot of Me-**18** at an isosurface value of 0.040, with through-space interactions (highlighted with blue circles) and the depicted vector field (arrows) of anisotropically induced ring currents. Right: structure of **18**. The external magnetic field is aligned orthogonal to the ring planes pointing towards the reader. The green arrows placed on top indicate the overall direction of the small arrows. Clockwise arrangement, in this regard, means a diatropic ring current is observable. Source data

The induced ring currents of (homo)aromatic systems by an external magnetic field were probed computationally as a measure for the homoaromaticity of **15** and **18**. In conjunction with (indirect) experimentally determined diatropicity via ^1^H NMR spectroscopy for the adjacent hydrogen atoms, magnetic indices can be used to theoretically correlate the degree of aromaticity. Two such magnetic indices that are widely used are nucleus-independent chemical shifts (NICS)^59–64^ and anisotropy of the induced current density (ACID)^65,66^, which were calculated for **15** and **18** (Fig. 6 and Supplementary Discussion Section 1.8).

To simplify the calculations, the methyl ester groups in **15** and **18** were replaced by methyl groups (now Me-**15**, Me-**18**). A geometric overlay of Me-**15** with Me-**18** showed slight differences in their homoaromatic skeleton (Supplementary Discussion Section 1.8). Besides the unsymmetrically aligned bridge in Me-**18**, we found through-space distances of 2.31 Å (6*π* current) and 2.47 Å (10*π* current) for Me-**15**, whereas these C–C distances slightly converge for Me-**18** to 2.37 Å (6*π* current) and 2.44 Å (10*π* current). Remarkably, elassovalene **3** shows a 9% longer C–C through-space distance of 2.45 Å for its (6*π* current) when compared to Me-**15**. Due to the non-planar *π*-ring geometry, we calculated NICS_zz_ values at various distances greater than 1.7 Å below the ring centroids. As a result of this non-planar geometry, the distances from the calculated NICS_zz_ values to the molecule are not fixed. A distance greater than 1.7 Å was chosen to minimize the effect of the *σ*-bond electrons on the *π*-electron system^61,62^. These one- and two-dimensional NICS_zz_ scans of Me-**15** (Fig. 6a,c) reveal a diatropic ring current of the C(4) to C(9) fragment of Me-**15**, with NICS(1.8)_zz_ values reaching down to −18.6 ppm. In contrast, the adjacent ring current in the C(2–4)···C(9–11) fragment is only moderately diatropic, with less negative NICS(1.8)_zz_ values of −12.5 ppm. Upon [1, 11] sigmatropic rearrangement to **18**, the diatropic ring currents are redistributed, with NICS(1.8)_zz_ values of −11.3 ppm and −15.2 ppm for the six- and seven-membered fragments, respectively. We interpret this as a shift of the local homoaromatic character from the C(9–11) fragment 6*π* system in Me-**15** to a more evenly distributed 10*π* system in Me-**18**, within the error of this challenging analysis of non-planar *π* systems^61^. In comparison, cycloheptatriene (**1**) and norcaradiene (**2**) show a substantially lower degree of homoaromaticity, with NICS(1.8)_zz_ values of −10.7 ppm and −9.3 ppm, respectively, whereas benzene shows a NICS(1.8)_zz_ value of −20.2 ppm at the same level of theory. Finally, the plotted ACID results unambiguously underscore the conjugation paths of the ring currents (Fig. 6e,f)^65,66^. A defined homoaromatic through-space interaction for Me-**15** can be observed between atoms C(2) and C(11) at a critical isosurface value (CIV) of 0.034, supporting a global 10*π* homoaromatic character, in agreement with the NICS analysis of **15** (in comparison, the CIV for non-planar cycloheptatriene **1** is 0.027 at the same level of theory^65,66^). A second through-space interaction between C(4) and C(9) is observed at a CIV of 0.026, indicating a local 6*π* homoaromatic ring current in **15**.

For **18** the situation is changed again, with a through-space interaction observed between C(1) and C(10) at a CIV of 0.040, indicating an enhanced global 10*π* homoaromaticity. In contrast, no through-space interaction can be observed for the local 6*π* system between C(4) and C(9), even at a CIV as low as 0.01 (Supplementary Fig. 36). We conclude that both **15** and **18** are globally 10*π* homoaromatic, with an enhanced local homoaromaticity in the 6*π* system of **15** and an evenly distributed 10*π* homoaromatic state in **18**. The latter is due to geometric constraints originating from the rearranged scaffold in **18**, allowing for efficient homoconjugation between C(1) and C(10) that facilitates a 10*π*-electron ring current.

## Conclusion

Assigning molecules as homoaromatic has been a long-standing problem, because molecules with sufficient stability and lifetime have been elusive. Here we have described the synthesis of homoannulene ester **15** and the related acid **16**, stable neutral homoaromatic hydrocarbons. Their homoaromatic state was evidenced by ^1^H NMR spectroscopic data, indicating a substantial ring current in addition to a high degree of bond-length equalization in the X-ray crystal structures. A detailed computational analysis by means of NICS-XY-scans scans and ACID isosurface plots revealed the ring currents and conjugation pathways. The local 6*π* homoaromatic state of homoannulene ester **15** can be photoswitched at 305 nm to a global 10*π* homoaromatic state by an unprecedented photochemical [1, 11] sigmatropic rearrangement to ester **18**. This photoreaction is reversible, and irradiation of **18** at 455 nm yields back homoannulene **15**. Therefore, **15**/**18** is a photoswitch based on a sigmatropic rearrangement that operates based on different homoaromaticity states. Notably, both **15** and **18** exhibit high thermal stability; the photochemical switch does not suffer from thermal background reactions, as these are thermochemically not allowed according to the Woodward–Hoffmann rules. We hope that interconvertible homoannulenes such as **15**${\leftrightarrows}$**18** may find use as molecular photoswitches based on homoaromatic stabilization effects.

## Online content

Any methods, additional references, Nature Portfolio reporting summaries, source data, extended data, supplementary information, acknowledgements, peer review information; details of author contributions and competing interests; and statements of data and code availability are available at 10.1038/s41557-022-01121-w.

## Supplementary information

Supplementary Information Supplementary discussions, experimental procedures, discussion of computational methods, Figs. 1–44 and Tables 1–23.

Supplementary Data 1 Crystallographic data (.cif, .fcf and .hkl files) of compound *cis*-11 (CCDC 2128305).

Supplementary Data 2 Crystallographic data (.cif, .fcf and .hkl files) of compound 12b (CCDC 2128302).

Supplementary Data 3 Crystallographic data (.cif, .fcf and .hkl files) of compound 13b (CCDC 2128309).

Supplementary Data 4 Crystallographic data (.cif, .fcf and .hkl files) of compound 14a (CCDC 2128304).

Supplementary Data 5 Crystallographic data (.cif, .fcf and .hkl files) of compound 14b (CCDC 2128306).

Supplementary Data 6 Crystallographic data (.cif, .fcf and .hkl files) of compound 15 (CCDC 2128303).

Supplementary Data 7 Crystallographic data (.cif, .fcf and .hkl files) of compound 15 (CCDC 2128308).

Supplementary Data 8 Crystallographic data (.cif, .fcf and .hkl files) of compound 15 (CCDC 2128307).

Supplementary Data 9 Crystallographic data (.cif, .fcf and .hkl files) of compound 15 (CCDC 2133482).

Supplementary Data 10 Example inputs for the computational methods used.

Supplementary Data 11 Computational geometry optimized coordinates of the investigated structures.

Supplementary Data 12 Python script used to extract the NICSzz values from the Gaussian log files.

Supplementary Data 13 Source data Supplementary Fig. 4.

Supplementary Data 14 Source data files for plotted graph in Supplementary Fig. 16.

## Data Availability

Crystallographic data for the structures reported in this Article have been deposited at the Cambridge Crystallographic Data Centre, under deposition nos. 2128305 (*cis*-**11**), 2128302 (**12b**), 2128309 (**13b**), 2128304 (**14a**), 2128306 (**14b**), 2128303 (**15**), 2128308 (**16**), 2128307 (**S9**) and 2133482 (**S11**). Copies of the data can be obtained free of charge via https://www.ccdc.cam.ac.uk/structures/. All other data supporting the findings of this Study are available within the Article and its Supplementary Information. For the calculations, templates of the input files as well as files with the coordinates of calculated structures have been provided. Source data are provided with this paper.

## Source data

Source Data Fig. 5b Source data files for plotted graph in Fig. 5b.
Source Data Fig. 6a Source data files for plotted graph in Fig. 6a.
Source Data Fig. 6b Source data files for plotted graph in Fig. 6b.
Source Data Fig. 6c Source data files for plotted graph in Fig. 6c.
Source Data Fig. 6d Source data files for plotted graph in Fig. 6d.
