# Supplementary material for: Photoswitching neutral homoaromatic hydrocarbons
Source: Nat Chem. 2023 Jan 26;15(3):377–85. doi: 10.1038/s41557-022-01121-w (PMC9986110; doi:10.1038/s41557-022-01121-w)
Supplement: Supplementary file 1 — Supplementary discussions, experimental procedures, discussion of computational methods, Figs. 1–44 and Tables 1–23. [file 41557_2022_1121_MOESM1_ESM.pdf]

nature chemistry

Article

https://doi.org/10.1038/s41557-022-01121-w

Photoswitching neutral homoaromatic hydrocarbons

In the format provided by the authors and unedited

Table of Contents

1 Supporting Information 3

1.1 General Information 3

1.1.1 Solvents 4

1.2 ^{1}H NMR chemical shift comparison 5

1.3 Investigation on conformational asymmetry in homoannulene 15 6

1.4 Comparison of bond lengths from X-ray crystal structures 7

1.4.1 Plot of the C–C-/C=C-bonds lengths 9

1.5 Proposed mechanism for the formation of homoannulene acid 16 and benzotropilidene S9 10

1.6 Synthesis 11

1.6.1 Optimization 12

1.6.2 General procedures 15

1.6.3 Experimental details 17

1.7 UV/vis Absorption Experiments 27

1.7.1 Switching in polar medium: MeCN 29

1.7.2 Photoswitch-Cycling monitored by HPLC (in MeCN) 32

1.7.3 Thermal Stability Test 34

1.7.4 UV/vis absorption spectroscopy and irradiation of purified (isolated) 18 36

1.7.5 Long-time irradiation / Fatigue Experiment 38

1.8 Computational Analysis 39

1.8.1 NICS Scans[17–21] 41

1.8.2 ACID Plots 45

1.8.3 Calculated ^{1}H NMR chemical shifts of 15, 18, Me-15, Me18, norcaradiene, cycloheptatriene, elassovalene and benzene 50

1.8.4 TD-DFT Results, Orbital Energy Levels of 15 and 18 and Orbital Plots 52

1.8.5 Strain Analysis on H-15, H-18, Elassovalene 56

2 Appendix 58

2.1 Crystallographic Data[29] 58

2.2 Spectral Data 92

2.3 Atomic Coordinates, methods and energies of calculated compounds, and TD-DFT results and Fragmentation used for Strain Analysis 226

3 References..237

1 Supporting Information

1.1 General Information

The numbering of the respective molecules in the main manuscript and supporting information do not necessarily follow IUPAC recommendations and were done at our own discretion in order to guide the reader.

All reactions were carried out in flame dried glassware under a nitrogen atmosphere using standard Schlenk techniques. Glassware and stir bars contaminated with transition metals were treated with *aqua regia* (conc. HCl/conc. HNO_3 3:1) prior to cleaning. For cleaning, glassware and stir bars were kept in an *iso*-PrOH/KOH bath overnight, rinsed with H_2O, kept in a citric acid/H_2O bath overnight and finally rinsed with deionized H_2O and dried at 120 °C. Solutions and reagents were added with nitrogen-flushed disposable syringes/needles. Solvents were added using glass syringes and stainless steel needles (stored at 120 °C).

Analytical thin layer chromatography (TLC) was performed on silica gel 60 G/UV_{254} aluminium sheets (*Macherey-Nagel*).

Flash column chromatography was performed on silica gel Davisil LC60A (40-63 µm, pore size 60 Å, *Grace*) using the indicated solvents.

NMR spectra were recorded on Bruker Avance II 400, Bruker Avance III 500 or Bruker Avance III 700 instruments at 298 K at the Institute for Chemistry of *Technische Universität Berlin* and using a Bruker Avance II 300 and a Bruker Avance II 500, Bruker Avance II 600 at 298 K at the Department of Chemistry of the Humboldt University of Berlin. Chemical shifts (δ) are reported in parts per million (ppm) and are referenced to the residual solvent resonance as the internal standard according to the standard literature[1,2]. Data are reported as follows: chemical shift, multiplicity (br s = broad singlet, s = singlet, d = doublet, t = triplet, q = quartet, m = multiplet, m_c = centrosymmetric multiplet), coupling constants (Hz), integration and – if possible – atom assignment. The assignment refers to the atom number shown in the corresponding molecule figure and was achieved *via* analysis of DEPT (DEPT 135) and 2D NMR spectra (COSY, HMQC, HSQC, HMBC, NOESY). If a distinct assignment was not possible, atoms were marked with "*" and are interchangeable. Designation "*Ar*" refers to atoms of an aromatic system where a distinct assignment was not possible.

Melting points (m.p.) were determined using a Leica Galen III melting point apparatus (*Wagner & Munz*).

Infrared (IR) spectra were recorded on a Cary 630 FT-IR spectrometer equipped with an ATR unit (*Agilent Technologies).* Mass spectra (HRMS) were obtained from the Analytical Facility at the Institute for Chemistry at *Technische Universität Berlin* (ESI/APCI: LTQ Orbitrap XL, *Thermo Scientific*; EI: GC-system 5975C, HP-5MS, *Agilent Technologies*). Analytical gas chromatography (GC) of reaction mixtures and pure substances was performed using a gas

chromatograph 430-GC (*Varian Inc.*). The instrument was equipped with a FactorFour VF-WAXms capillary column (*Varian Inc., length*: 30 m, inner diameter: 0.25 mm, film thickness of the stationary phase: 0.25 µm). The following temperature program was used for the analysis: carrier gas N_2; injection temperature 270 °C; detector temperature 270 °C; flow rate 4.0 mL/min; temperature program: 40 °C start temperature, 20 °C/min heating rate to 250 °C for 10 min, then 20 °C/min heating rate to final temperature 260 °C for 5 min. The data was recorded with the program Galaxie 1.9.302.952 (*Varian Inc.*).

Ultra-Performance Liquid Chromatography with Mass Spectrometry coupling (UPLC/MS) was performed with a Waters ACQUITY UPLC H-Class, equipped with a quaternary solvent manager (QSM), a sample manager-flow through needle (SM-FTN), a column heater, a column manager, a ACQUITY UPLC BEH Phenyl 1.7 µm, 2.1x100 mm column, a ACQUITY UPLC BEH C18 1.7 µm, 2.1 x 100 mm column and a photodiode array detector (PDA eλ), and a ACQUITY QDa detector. The mobile phase is a gradient of MeCN/H_2O individually optimized for each separation.

Ultraviolet–visible absorbance spectroscopy was performed on Agilent Cary 50 and Cary 60 instruments connected to a cryostat from Unisoku Scientific Instruments (temperature accuracy ±0.1 K) in 10 × 10 mm quartz cuvettes with 3 mL volume. Weighing of small quantities was performed on a Sartorius ME5 analytical microbalance. Ultraviolet–Visible Fluorescence Spectroscopy was performed on a Varian Cary Eclipse Fluorescence spectrometer using 10.0×10.0 mm quartz cuvettes. 305 nm mounted LED (LG Innotek, LEUVA66H100KU00, FWHM = 13 nm, 850 mW (min), 500 mA), 340 nm mounted LED (Seoul Viosys, CUD4AF1B, FWHM = 10 nm, 430 mW (min), 500 mA), 455 nm mounted LED (Osram OSLON SSL80, LD-CQ7P-1U3U, FWHM = 21 nm, 360 mW (min), 1000 mA).

The parameters for flash column chromatography are given as „(d × h, A/B = a:b, C, #n–m)“, with “d” = column diameter; “h” = filling height; “A/B” = eluent solvents; “a:b” = solvent ratio; “C” = fraction volume and #n–m“ = fraction number.

1.1.1 Solvents

THF and Et_2O were dried over sodium/benzophenone and distilled under N_2 atmosphere prior to use. Et_3N, CH_2Cl_2 and MeOH were dried over CaH_2 and distilled under N_2 atmosphere prior to use. Acetone and EtOH were distilled under reduced pressure prior to use. Solvents (technical grade) for extraction/chromatography (*n*-pentane, cyclohexane, CH_2Cl_2, *tert*-butyl methyl ether, EtOAc) were distilled under reduced pressure prior to use. MeCN for irradiation experiments was purged with Ar prior to use.

1.2 ^{1}H NMR chemical shift comparison

For investigation of a present induced ring current, we compared the ^{1}H NMR chemical shifts of elassovalene **3**[3,4], homoannulenes **15** & **16** and related annulenes **S1–S4**[5,6] (Supplementary Figure 1). The chemical shift of the methine resonance $\delta_{\mathbf{3},\mathrm{methine}}$ = 1.77 ppm of elassovalene **3** (red) does not indicate a present ring current. Compared to the annulene **S1**, which is considered aromatic, the methine resonance $\delta_{\mathbf{S1},\mathrm{methine}}$ = –1.82 ppm is significantly shifted up-field, indicative of an existing ring current. In homoannulene **15**, the methine proton is facing away from the potential homoaromatic system with a chemical shift of $\delta_{\mathbf{15},\mathrm{methine}}$ = 3.81 ppm. The α-carbonyl methine ^{1}H NMR resonance of homoannulene carboxylic acid **16**, placed directly on top of the homoaromatic system, is significantly shifted up-field to $\delta_{\mathbf{16},\mathrm{methine}}$ = 0.89 ppm, evidence for a present ring current. Although the chemical shift is not in the negative ppm range, it should be considered that it is α to a ketone. Comparing it to the annulene **S2**, which also has α-carbonyl methine placed directly on top triene with $\delta_{\mathbf{16},\mathrm{methine}}$ = 2.40 ppm, ^{1}H NMR resonance is not as up-field shifted as in homoannulene **16**, suggesting the absence of a ring current. The related aromatic annulenes **S3** and **S4** show an even higher shielding of their respective methine protons ($\delta_{\mathbf{S3},\mathrm{methine}}$ = –1.16 ppm and $\delta_{\mathbf{S4},\mathrm{methine}}$ = –0.87 ppm). This comparison suggest a present induced ring current in **15** and **16**, with weaker shielding effect compared to the aromatic annulenes **S3** and **S4**, but with the right tendency towards (homo)aromaticity.

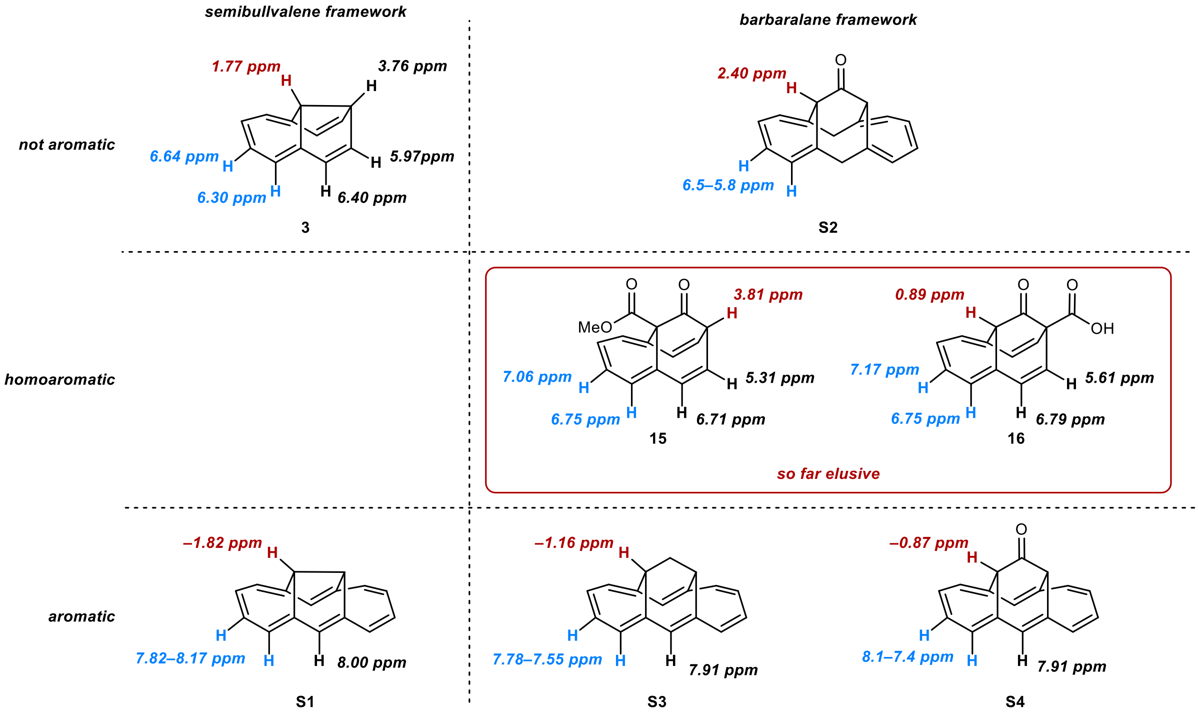

Supplementary Figure 1. Comparison of chemical shifts in ^{1}H NMR of the elassovalene **3**[3,4], homoannulenes **15** & **16** and related annulenes **S1–S4**[5,6].

1.3 Investigation on conformational asymmetry in homoannulene 15

The ^{1}H NMR of the homoannulene **15** showed a distinct AA'XX' system of the olefinic hydrogen atoms. This suggests that the rotation of the ester group is restricted which breaks the plane of symmetry in **15**, resulting in the observed diastereotopic alkene hydrogen atoms. This hypothesis is further supported by the comparison of the ^{1}H NMR spectrum of homoannulene acid **16**, in which no such restriction of rotation for the carboxyl group is expected and no such coupling pattern was observed. To investigate this barrier of rotation in homoannulene **15** we performed a variable temperature NMR study (Supplementary Figure 2). With increasing temperature, the coalesce of the olefinic protons was observed at ca. 100 °C. This supports the notion that there is a restricted rotation for the ester group.

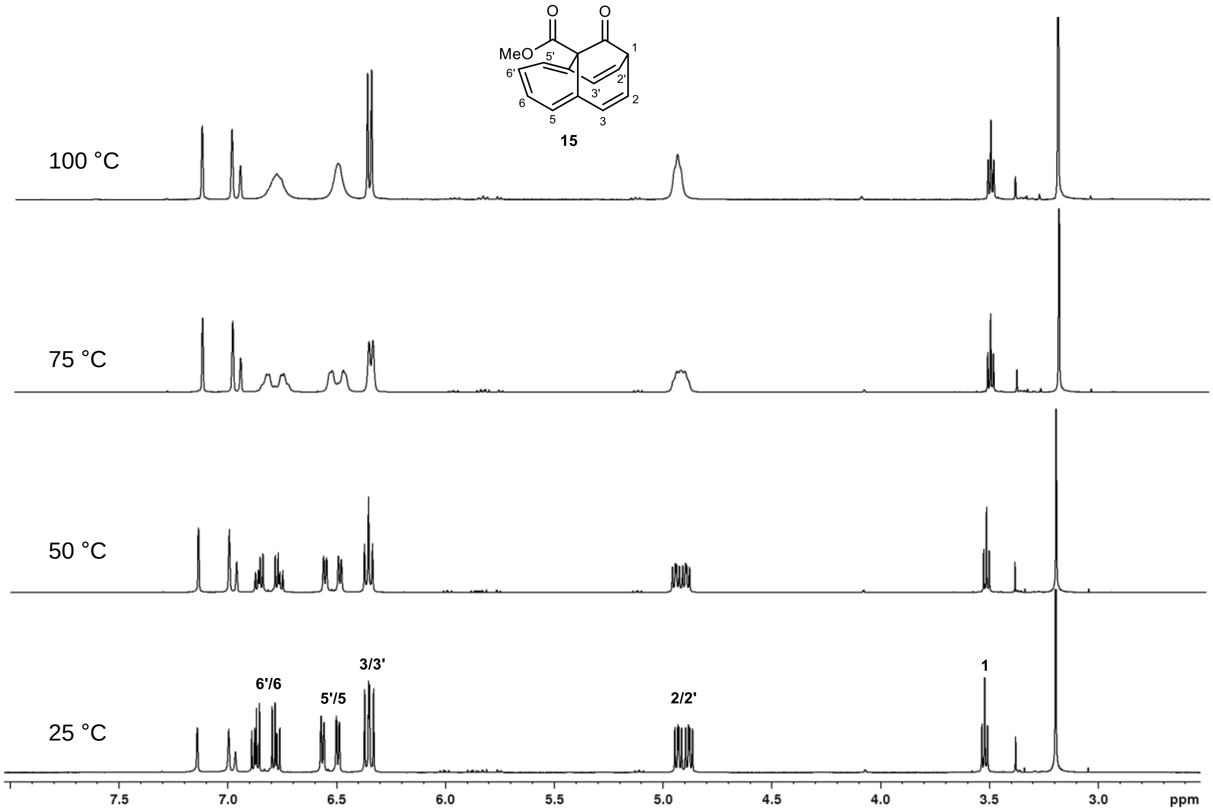

Supplementary Figure 2. ^{1}H NMR spectra of homoannulene **15** at different temperatures (500 MHz, C_6D_5Cl).

1.4 Comparison of bond lengths from X-ray crystal structures

The HOMA[7–9] (Harmonic Oscillator Model of Aromaticity) indices were calculated using the following equation with normalization factor α = 257.7 Å^{-2}, the number of CC-bonds n, distances of i-th ring bond R_i and the reference optimal bond in benzene R_{opt} = 1.388 Å. The HOMA index approaches 1 for aromatic compounds (benzene as reference) and approaches 0 for non-aromatic structures (cyclohexatriene as reference).

$$HOMA = 1 - \frac{\alpha}{n}\sum_{i=1}^{n}(R_i - R_{opt})^2$$

Supplementary Equation 1. HOMA index equation.

For triasterane **14a** HOMA(**14a**) = –0.8860, which tends towards 0 and is considered not aromatic. For annulene **17** the HOMA index is 0.9097, which approaches 1 and is in accordance with the notion that it is aromatic. Homoannulenes **15** and **16** also show the tendency towards 1 with HOMA(**15**) = 0.8558 and HOMA(**15**) = 0.7614, suggesting that **15** and **16** show aromatic properties.

For barbaralane **4** the HOMA index of –2.9392 fails to reflect its homoaromatic character. The HOMA index shows how different to the perfect aromaticity in benzene the molecule in question is. The more different, unequal, and alternating the bonds are compared to benzene, the lower the HOMA index gets. It reflects similarity of the geometric properties to the aromatic geometry. Barbaralane **4** has one more single bond than double bond and the C-C-bond of the cyclopropyl (1.677 Å) differs greatly from the ideal C–C bond in benzene (1.388 Å), which is weighted in the equation disproportionally high. We concluded, that the HOMA index reaches the limit[8] in the case of homoaromatic compounds which do not possess a fully conjugated system, such as in the case of barbaralane **4**.

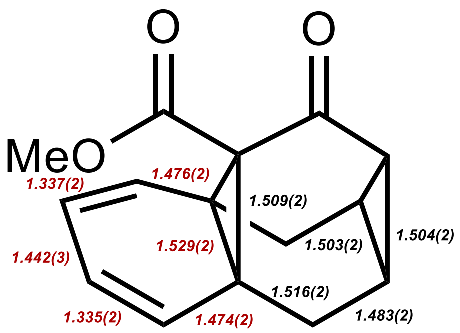

14a
1.34 – 1.53 Å
(cyclohexadiene)
HOMA = –0.8860

C(sp^3)–C(sp^3)	*1.54 Å*
C(sp^3)–C(sp^2)	*1.50 Å*
C(sp^2)–C(sp^2)	*1.47 Å*
C=C (benzene)	*1.39 Å*
C=C (ethylene)	*1.34 Å*

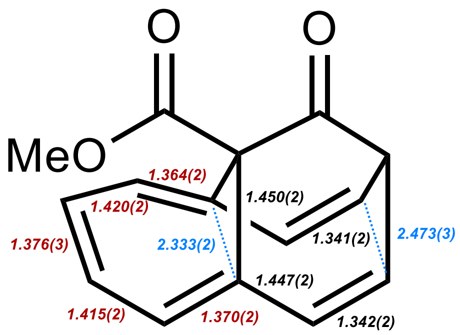

15
1.36 – 1.42 Å
(cycloheptatrien)
HOMA = 0.8558

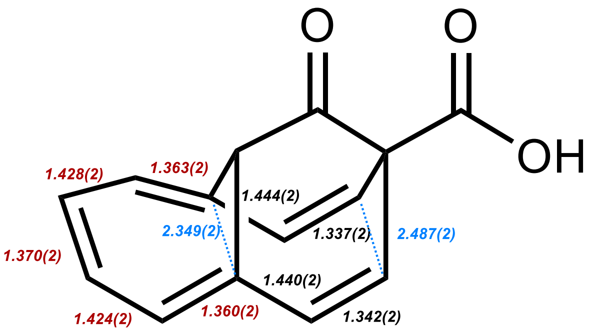

16
1.36 – 1.43 Å
(cycloheptatriene)
HOMA = 0.7614

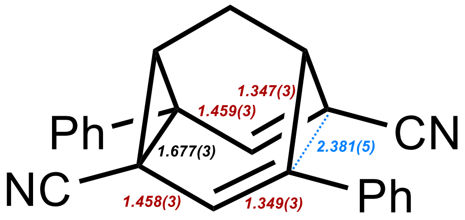

4
Quast
1.35 – 1.46 Å
HOMA = –2.9392

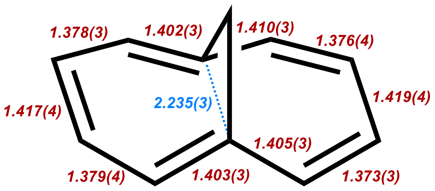

17
Vogel
1.37– 1.42 Å
HOMA = 0.9097

Supplementary Figure 3. Comparison of bond lengths from X-ray structures and HOMA (Harmonic Oscillator Model of Aromaticity) indices of triasterane **14a**, homoannulenes **15** & **16**, barbaralane **4** by Quast[10] and methano[10]annulene **17** by Vogel[11].

1.4.1 Plot of the C–C-/C=C-bonds lengths

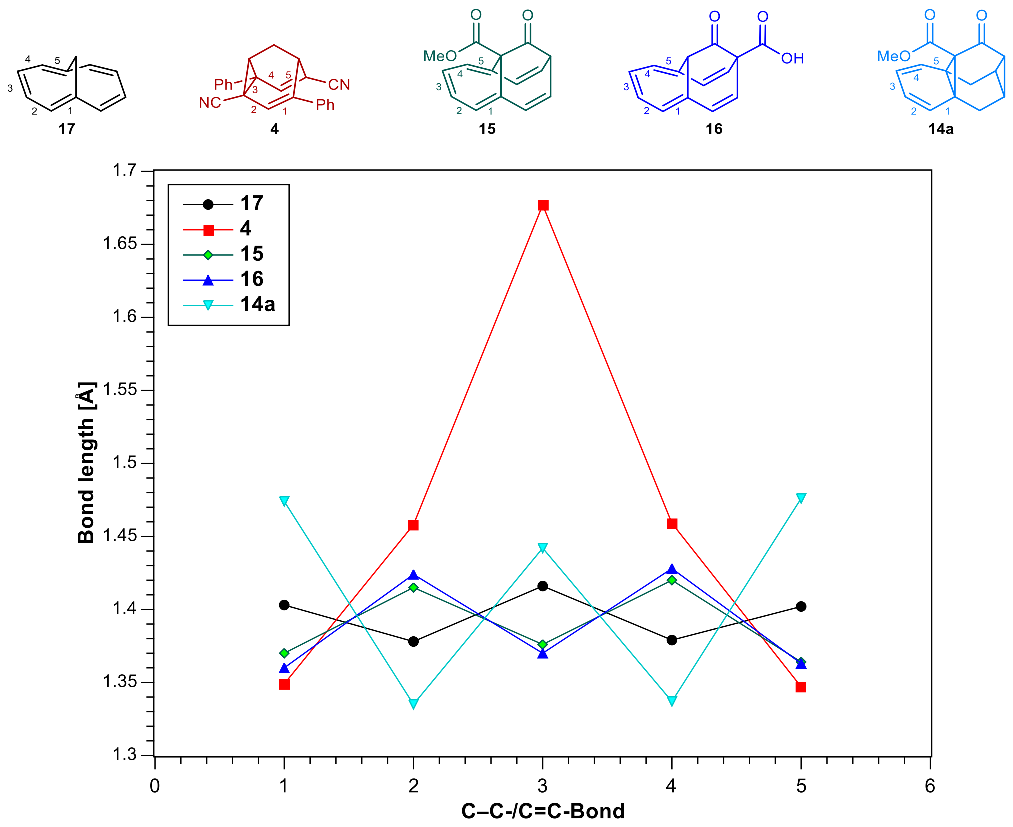

Supplementary Figure 4. Plot of the alternating bond lengths of annulene **17**, barbaralane **4**, homoannulene **15** and **16** and asterane **14a**.

1.5 Proposed mechanism for the formation of homoannulene acid 16 and benzotropilidene S9

For the formation of homoannulene acid **16** and benzotropolidene **S4** we propose that the donation of electrons of the homoaromatic system to the $\sigma^*_{C\text{–}C}$ orbital, which weakens the C–C bond. (Supplementary Figure 5) Due to this interaction the hydroxide anion attacks the ketone carbonyl carbon and breaks the C–C bond in a *retro*-Claisen reaction. After a second deprotonation carbanion **S5** is formed which can react further in two pathways: a) In a Claisen reaction homoannulene **16** is formed and b) the ring contracts and forms the carbanion **S6**. After protonation forming ester **S7** it undergoes a [1,5] *H*-shift forming ester **S8**, which is driven by rearomatization. After a second saponification and work-up the benzotropilidene **S9** is formed (X-ray structure available, see Section 2.1.8).

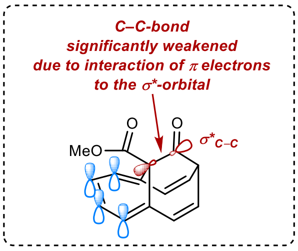

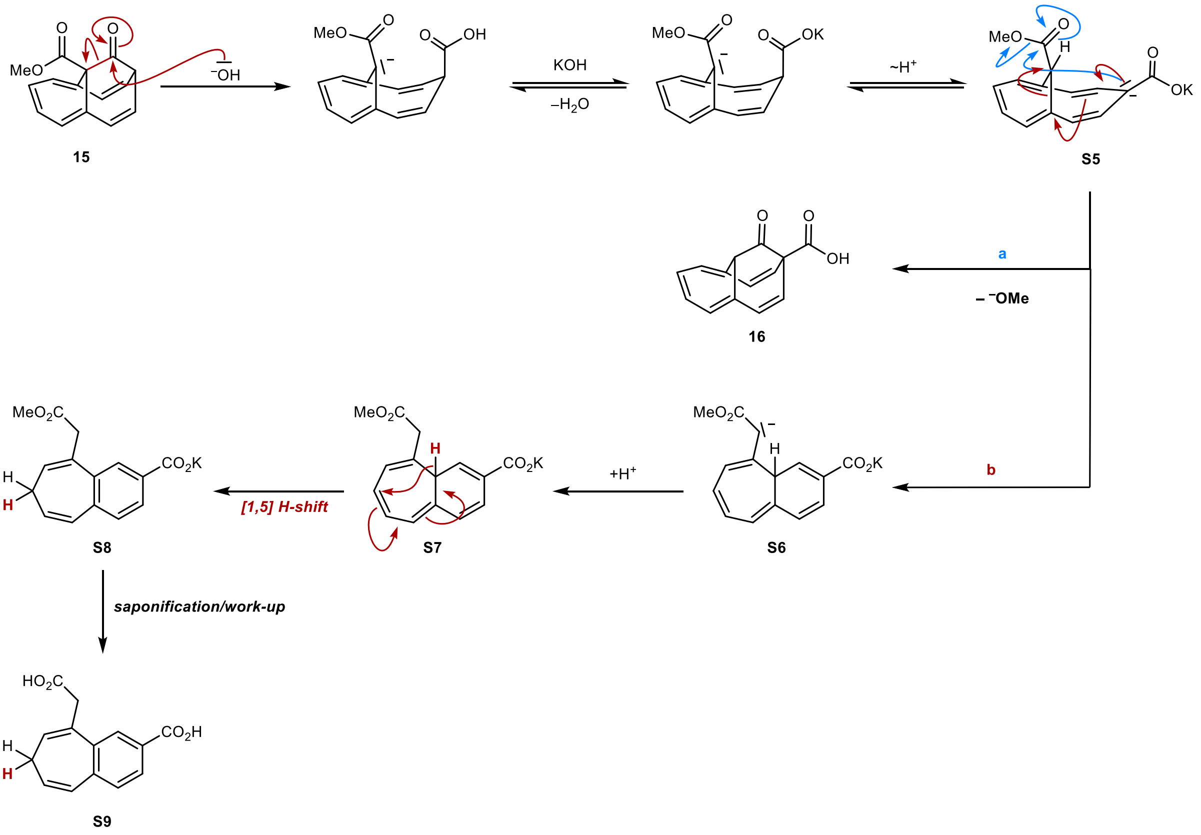

Supplementary Figure 5. Proposed mechanism for the formation of homoannulene acid **16** and benzotropilidene **S6**.

1.6 Synthesis

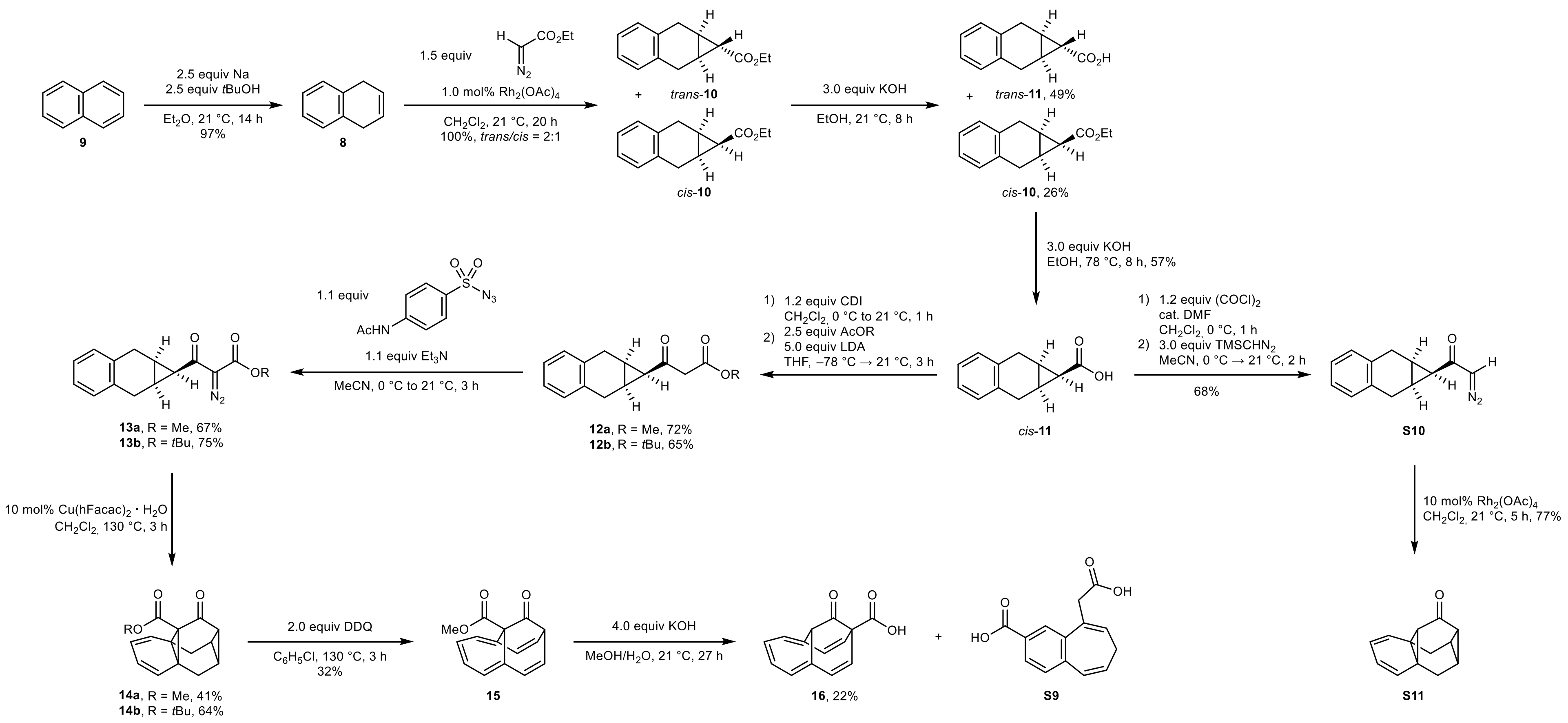

Supplementary Figure 6. Synthesis of homoannulene **15** and homoannulene acid **16**. (Abbr.: CDI = *N*,*N'*-carbonyldiimidazole; hFacac = hexafluoroacetylacetonate; DDQ = 2,3-dichloro-5,6-dicyano-1,4-benzoquinone)

1.6.1 Optimization

1.6.1.1 Dearomative intramolecular cyclopropanation of 13

1.6.1.1.1 Procedure

A flame dried 2 mL microwave *via*l was charged with diazoacetate solution **13** (12.5 mM in CD_2Cl_2, 400 µL, 5.00 µmol, 1.00 equiv) with internal standard (mesitylene). Then catalyst solution (5.00 mM in CD_2Cl_2, 100 µL, 0.500 µmol, 10.0 mol%) was added and the microwave *via*l was sealed. When elevated temperatures were used, the reaction mixtures were heated under microwave reaction conditions (see Supplementary Table 1 & 2). After the indicated reaction time the reaction mixture allowed to cool to room temperature and the solution directly analyzed *via* ^{1}H NMR spectroscopy.

Supplementary Table 1. Optimization of rhodium(II)- and copper(II)-based catalysts for dearomative intramolecular cyclopropanation of **13a**.[a]

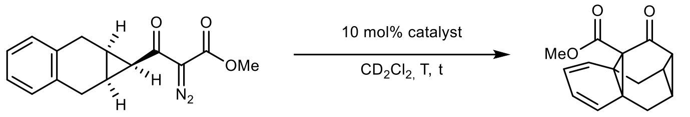

13a **14a**

Entry	Catalyst	Temperature	Time	Conversion[b]	Yield 14a[b]
1	$Rh_2(OAc)_4$	100 °C	1 min	full conv.	0%
2	$Rh_2(esp)_2$	100 °C	1 min	full conv.	0%
3	$Rh_2(TPA)_4$	100 °C	1 min	full conv.	0%
4	$Rh_2(TFA)_4$	100 °C	1 min	full conv.	20%
5[c]	$Rh_2(TFA)_4$	rt	18 h	full conv.	8%
6[c]	$Rh_2(TFA)_4$	0 °C	18 h	full conv.	13%
7[c]	$Rh_2(TFA)_4$	–78 °C	18 h	full conv.	18%
8	$Cu(acac)_2$	130 °C	1 min	no conv.	0%
9	$Cu(TMHD)_2$	130 °C	1 min	no conv.	0%
10	$Cu(tFacac)_2$	130 °C	1 min	no conv.	0%
11	$Cu(hFacac)_2$	130 °C	1 min	full conv.	93%

[a] Reactions were performed with diazoacetate **13a** (5 µmol) and catalyst (0.5 mmol) in CD_2Cl_2 (0.5 mL); [b] Determined *via* ^{1}H NMR spectroscopy with mesitylene as internal standard; [c] Reaction was performed without the use of microwave conditions. Abbr.: esp = α,α,α',α'-tetramethyl-1,3-benzenedipropionate; TPA = triphenylacetate;

TMHD = 2,2,6,6-tetramethyl-3,5-heptandionate; tFacac = trifluoroacetylacetonate; hFacac = hexafluoroacetylacetonate.

Supplementary Table 2. Optimization of rhodium(II)- and copper(II)-based catalysts for the dearomative intramolecular cyclopropanation of **13b**.[a]

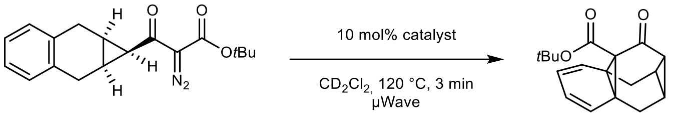

13b **14b**

Entry	Catalyst	Conversion[b]	Yield 14b[b]
1	$Rh_2(OAc)_4$	full conv.	0%
2	$Rh_2(TFA)_4$	full conv.	0%
3	$Rh_2(TPA)_4$	full conv.	0%
4	$Rh_2(esp)_2$	full conv.	0%
5	$Cu(acac)_2$	no conv.	0%
6	$Cu(TMHD)_2$	no conv.	0%
7	$Cu(tfacac)_2$	no conv.	0%
8	$Cu(hfacac)_2$	85%	63%

[a] Reactions were performed with diazoacetate **13b** (5 µmol) and catalyst (0.5 mmol) in CD_2Cl_2 (0.5 mL); [b] Determined *via* ^{1}H NMR spectroscopy with mesitylene as internal standard. Abbr.: esp = $\alpha,\alpha,\alpha',\alpha'$-tetramethyl-1,3-benzenedipropionate; TPA = triphenyl-acetate; TMHD = 2,2,6,6-tetramethyl-3,5-heptandionate; tFacac = trifluoroacetylacetonate; hFacac = hexafluoroacetylacetonate.

1.6.1.2 Oxidative dehydrogenation of 14a

Supplementary Table 3. Probing different conditions for an oxidative dehydrogenation of triasterane **14a**.

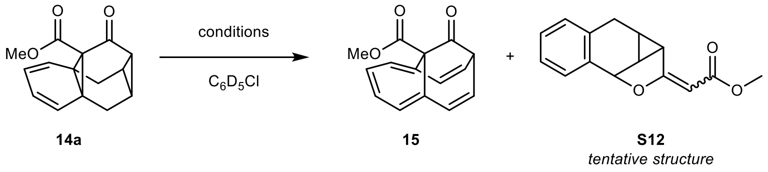

Entry	Conditions	Conversion[b]	Yield 15[b]	Yield S12[b]
1	2.0 equiv DDQ<br>130 °C, 5 h	full conv.	35%	0%
2	2.0 equiv *p*-chloranil<br>130 °C, 3 h	no conv.	0%	0%
3	2.0 equiv *p*-chloranil<br>150 °C, 18 h	no conv.	0%	0%
4	2.0 equiv *o*-chloranil<br>130 °C, 3 h	no conv.	0%	0%
5	10 mol% Pd/C<br>150 °C, 35 h	full conv.	0%	96%
6	10 mol% Pd/C<br>1.0 equiv norbornene<br>150 °C, 35 h	full conv.	0%	94%
7	10 mol% $(Ph_3P)_3RhCl$<br>160 °C, 22 h	full conv.	0%	95%
8	1.0 equiv S_8<br>160 °C, 22 h	no conv.	0%	0%

[a] Reactions were performed in J Youngs NMR tube with triasterane **14a** (12.1 mg, 50.0 µmol, 1.00 equiv) and in C_6D_5Cl (0.5 mL); [b] Determined *via* ^{1}H NMR spectroscopy with mesitylene as internal standard. Abbr.: DDQ = 2,3-dichloro-5,6-dicyano-1,4-benzoquinone

1.6.2 General procedures

1.6.2.1 GP1 – Synthesis of acetoacetate 12

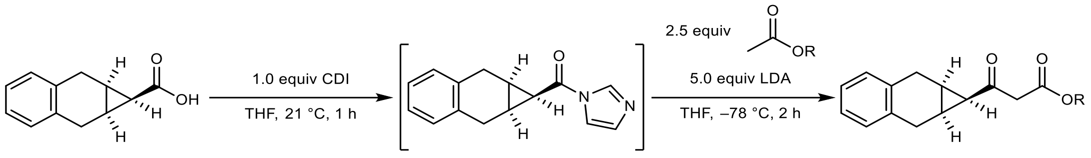

cis-**11** **12**

A Schlenk flask is charged with carboxylic acid *cis*-**11** (1.00 equiv) and dissolved in THF (7.5 mL/mmol carboxylic acid). *N*,*N*'-carbonyldiimidazole (1.00 equiv) is added and the reaction mixture is stirred at room temperature for 3 h. In a separate Schlenk flask di-*iso*-propylamine (5.00 equiv) is dissolved in THF (1.5 mL/mmol di-*iso*-propylamine) and cooled down to –78 °C. *n*BuLi (5.00 equiv) is added dropwise and the reaction mixture is stirred at –78 °C for 1 h. A solution of acetate (2.50 equiv) and THF (1.5 mL/mmol acetate) is added dropwise to the LDA solution, and the mixture is stirred at –78 °C for 1 h. The solution of activated carboxylic acid is *trans*ferred dropwise to the enolate *via* a Teflon canula at –78 °C. After complete addition the reaction mixture is stirred at –78 °C for 3 h and is warmed up to room temperature. The reaction is quenched *via* addition of saturated aqueous NH_4Cl-solution (5 mL/mmol *n*BuLi), the layers are separated, and the aqueous layer is extracted with *tert*-butyl methyl ether (3 × 10 mL/mmol). The combined organic layer was washed with saturated aqueous $NaHCO_3$-solution (2 × 10 mL/mmol) and brine and is dried over Na_2SO_4. After filtration and removal of all volatiles under reduced pressure the crude product is purified *via* flash column chromatography on silica gel.

1.6.2.2 GP2 – Diazo *trans*fer reaction to diazoacetoacetate 13

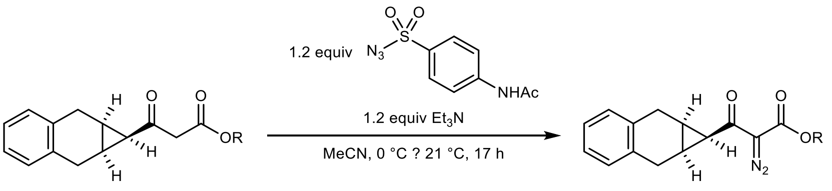

12 **13**

A Schlenk flask is charged with 4-acetamidobenzenesulfonyl azide (1.2 equiv), dissolved in MeCN (10 mL/mmol) and the solution is cooled down to 0 °C. First triethylamine (1.2 equiv) then a solution of acetoacetate **12** (1.0 equiv) and MeCN (3.3 mL/mmol) is slowly added to the reaction mixture. The mixture is stirred at 0 °C for 1 h, is warmed up to room temperature and stirred for additional 15 h. After full conversion (monitored *via* TLC) the formed white precipitate is filtered off *via* a glass frit and washed with MeCN (3.3 mL/mmol). All volatiles of the filtrate are removed under reduced pressure and the crude product is purified *via* flash column chromatography on silica gel.

1.6.2.3 GP3 – Intramolecular Buchner dearomatization to triasterane 14

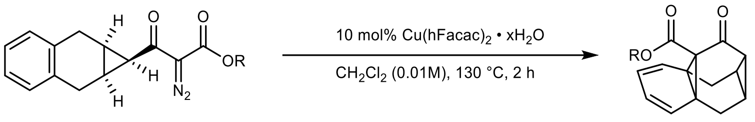

13 **14**

An Ace pressure tube is charged with copper(II) hexafluoroacetylacetonate hydrate (10 mol%) and CH_2Cl_2 (90 mL/mmol). A solution of diazoacetoacetate **13** (1.00 equiv) and CH_2Cl_2 (10 mL/mmol) is added to the catalyst solution and the pressure tube is tightly closed. The reaction mixture is heated to 130 °C in an oil bath for 2 h. After complete conversion (monitored *via* TLC) all volatiles are removed under reduced pressure and the crude product is purified *via* flash column chromatography on silica gel.

Alternative procedure:

A three-neck round-bottom flask is charged with copper(II) hexafluoroacetylacetonate hydrate (10 mol%) and chlorobenzene (45 mL/mmol) and is heated up till reflux. A solution of diazoacetoacetate **13** (1.00 equiv) and chlorobenzene (5 mL/mmol) is added to the catalyst solution under reflux *via* a syringe pump over 4 h. After complete conversion (monitored *via* TLC) all volatiles are removed under reduced pressure and the crude product is purified *via* flash column chromatography on silica gel.

1.6.3 Experimental details

1.6.3.1 Synthesis of annulene ester 15

1.6.3.1.1 1,4-Dihydronaphthalene (8)

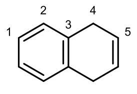

8
$C_{10}H_{10}$
M = 130.19 g mol^{-1}

According to a literature procedure[12], a Schlenk flask was charged with naphthalene (**9**, 12.8 g, 100 mmol, 1.00 equiv) and dry Et_2O (200 mL). Sodium (5.75 g, 250 mmol, 2.50 equiv) was added in portions to the reaction mixture and stirred at room temperature for 15 min. *tert*-Butanol (18.5 g, 250 mmol, 2.50 equiv) was added dropwise to the reaction mixture and was stirred at room temperature for 14 h. The mixture was filtered through a glass frit and the filter cake was washed with Et_2O (50 mL). The combined organic layers were washed with H_2O (2 × 100 mL) and brine (100 mL), dried over Na_2SO_4. Filtration and removal of all volatiles under reduced pressure afforded 1,4-dihydronaphthalene (**8**) as a colourless oil and was used without further purification (12.6 g, 96.6 mmol, 97%). **TLC** (SiO_2, cyclohexane/EtOAc = 9:1, v/v): $\mathbf{R}_f$ = 0.62; **^{1}H NMR** (400 MHz, CD_2Cl_2): δ 3.38 (d, $^3J_{4,5}$ = 1.3 Hz, 4H, H-4), 5.93 (m_c, 2H, H-5), 7.12 (m_c, 4H, H-1, H-2); **^{13}C NMR** (101 MHz, CD_2Cl_2): δ 30.1 (C-4), 125.2 (C-5), 126.2 (C-1), 128.7 (C-2), 134.7 (C-3); **HRMS** (m/z): [($M^{\bullet+}$)] calcd. for $C_{10}H_{10}^{\bullet+}$, 130.0777; found, 130.0771.

The data is in accordance with literature[13].

1.6.3.1.2 1-((1*r*,1a*R*,7a*S*)-1a,2,7,7a-Tetrahydro-1*H*-cyclopropa[b]naphthalen-1-yl)propan-1-on (10)

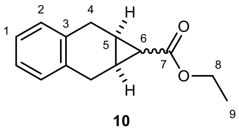

10
$C_{14}H_{16}O_2$
M = 216.28 g mol^{-1}

According to a literature procedure[14,15], a Schlenk flask was charged with $Rh_2(OAc)_4$ (22 mg, 50 µmol, 0.10 mol%) and 1,4-dihydronaphthalene (**8**, 6.51 g, 50.0 mmol, 1.00 equiv). Ethyl diazoacetate (15% in toluene, 57.2 g, 75.0 mmol, 1.50 equiv) was slowly added to the reaction mixture at room temperature over 16 h. The reaction mixture was filtered over a plug of Al_2O_3 (3 x 5 cm, eluent: CH_2Cl_2, 100 mL). After removal of all volatiles under reduced pressure the crude product was purified *via* flash column chromatography on silica gel (9 × 12 cm, cyclohexane/EtOAc = 20:1, 100 mL, #17–25) to afford ester **10** as a colourless oil (10.8 g, 50.0 mmol, 100%, *trans/cis* = 2:1).

Alternative procedure:

According to a literature procedure[14,15], a Schlenk flask was charged with $Rh_2(OAc)_4$ (84.9 mg, 192 µmol, 0.10 mol%) and 1,4-dihydronaphthalene (**8**, 25.0 g, 192 mmol, 1.00 equiv). Ethyl diazoacetate (15% in toluene, 245 mL, 288 mmol, 1.50 equiv) was slowly added to the reaction mixture at room temperature over 18 h. The reaction mixture was filtered over a plug of Al_2O_3 (3 × 5 cm, eluent: CH_2Cl_2, 200 mL). After removal of all volatiles under reduced pressure the

crude product was purified *via* distillation under reduced pressure (3.3×10^{-1} mbar), where the impurities were distilled out from the crude product mixture. The distillation was stopped when the head temperature exceeded 100 °C (oil bath temperature: 130 °C). Ester **10** was afforded as a yellow oil (39.3 g, 182 mmol, 95%, 2:1 dr). **TLC** (SiO_2, cyclohexane/EtOAc = 9:1, v/v): $\mathbf{R}_f$ = 0.47 (SiO_2, cyclohexane/EtOAc = 9:1); **^{1}H NMR** (400 MHz, CD_2Cl_2): δ 1.08 (t, $^3J_{9,8}$ = 7.1 Hz, 3H, H-9$_{cis}$), 1.19 (t, $^3J_{9,8}$ = 7.1 Hz, 3H, H-9$_{trans}$), 1.39 (t, $^3J_{6,5}$ = 3.9 Hz, 1H, H-6$_{trans}$), 1.69–1.73 (m, 3H, H-5$_{cis}$, H-6$_{trans}$), 1.91 (m$_c$, 2H, H-5$_{trans}$), 2.95–3.15 (m, 4H, H-4), 3.77 (q, $^3J_{8,9}$ = 7.1 Hz, 2H, H-8$_{cis}$), 4.04 (q, $^3J_{8,9}$ = 7.1 Hz, 2H, H-8$_{trans}$), 7.00–7.12 (m, 4H, H-1, H-2); **^{13}C NMR** (101 MHz, CD_2Cl_2): δ 14.3 (C-9$_{cis}$), 14.5 (C-9$_{trans}$), 17.3 (C-5$_{cis}$), 19.1 (C-6$_{trans}$), 21.3 (C-6$_{cis}$), 22.4 (C-5$_{trans}$), 25.1 (C-4$_{cis}$), 28.6 (C-4$_{trans}$), 60.2 (C-8$_{cis}$), 60.6 (C-8$_{trans}$), 125.9 (C-1$_{cis}$), 126.8 (C-1$_{trans}$), 128.6 (C-2$_{cis}$), 129.2 (C-2$_{trans}$), 134.2 (C-3$_{trans}$), 136.4 (C-3$_{cis}$), 174.4 (C-7$_{trans}$), 176.5 (C-7$_{cis}$); **HRMS** (m/z): [$(M+H)^+$] calcd. for $C_{14}H_{17}O_2^+$, 217.1223; found, 217.1230.

The data is in accordance with literature[14,15].

1.6.3.1.3 (1*r*,1a*R*,7a*S*)-1a,2,7,7a-Tetrahydro-1*H*-cyclopropa[b]naphthalene-1-carboxylic acid (*trans*-11) & ethyl (1*s*,1a*R*,7a*S*)-1a,2,7,7a-tetrahydro-1*H*-cyclopropa[b]naphthalene-1-carboxylate (*cis*-10)

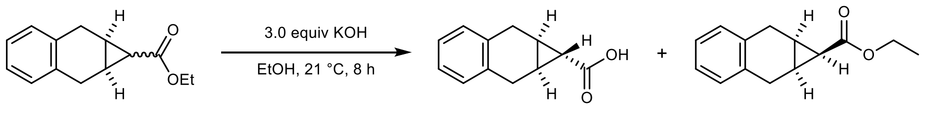

A round-bottom flask was charged with ester **10** (*trans/cis* = 67:33, 8.60 g, 39.8 mmol, 1.00 equiv) and EtOH (75 mL). Potassium hydroxide (6.68 g, 119 mmol, 3.00 equiv) was added and the reaction mixture was stirred at room temperature for 8 h. Then H_2O (300 mL) was added, the layers were separated.

1.6.3.1.3.1 For isolation of *trans*-carboxylic acid *trans*-11:

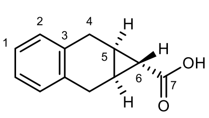

trans-**11**
$C_{12}H_{12}O_2$
M = 188.23 g/mol

The aqueous layer was washed with *tert*-butyl methyl ether (3 × 100 mL). Then conc. aqueous HCl-solution (32%) was added until pH ≤ 1 was set and the aqueous layer was extracted with *tert*-butyl methyl ether (3 × 100 mL). The combined organic layers were washed with brine and dried over Na_2SO_4. After filtration and removal of all volatiles under reduced pressure the crude product was purified *via* recrystallization in cyclohexane and EtOAc to afford acid *trans*-**11** as a colourless solid (2.20 g, 11.7 mmol, 29%). **TLC** (SiO_2, cyclohexane/EtOAc = 9:1, v/v): $\mathbf{R}_f$ = 0.16; **mp**: 156 °C (cyclohexane/EtOAc); **^{1}H NMR** (400 MHz, $CDCl_3$): δ 1.48 (t, $^3J_{6,5}$ = 3.83 Hz, 1H, H-6), 2.03 (m$_c$, 2H, H-5), 3.06 (d, $^2J_{4a,4b}$ = 15.9 Hz, 2H, H-4a), 3.14 (d, $^2J_{4b,4a}$ = 15.9 Hz, 2H, H-4b), 6.99–7.03 (m, 2H, H-2), 7.11–7.14 (m, 2H, H-1), 11.43 (br s, 1H,

O*H*); **^{13}C NMR** (101 MHz, $CDCl_3$): δ 18.7 (C-6), 23.3 (C-5), 28.3 (C-4), 126.7 (C-1), 129.1 (C-2), 133.4 (C-3), 188.5 (C-7); **HRMS** (m/z): [(M+H)$^+$] calcd. for $C_{12}H_{13}O_2^+$, 189.0910; found, 189.0909; **IR** (ATR): 3023 (w), 2920 (w), 2833 (w), 1666 (s), 1495 (w), 1438 (m), 1337 (w), 1274 (s), 1201 (s), 1103 (m), 1039 (m), 1006 (m), 922 (s), 848 (s), 749 (s), 678 cm^{-1} (s).

1.6.3.1.3.2 For isolation of *cis*-ester *cis*-10:

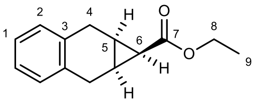

cis-**10**
$C_{14}H_{16}O_2$
M = 216.28 g mol^{-1}

The aqueous layer was extracted with *tert*-butyl methyl ether (3 × 100 mL). The combined organic layers were washed with brine and dried over Na_2SO_4. After filtration and removal of all volatiles under reduced pressure the crude product was purified *via* flash column chromatography on silica gel (2.5 × 12 cm, cyclohexane/EtOAc = 10:1, 20 mL, #2–7) to afford ester *cis*-**10** (2.28 g, 10.5 mmol, 26%) as a colourless oil. **TLC** (SiO_2, cyclohexane/EtOAc = 9:1, v/v): **R_f** = 0.47; **^{1}H NMR** (400 MHz, $CDCl_3$): δ 1.07 (t, $^3J_{9,8}$ = 7.1 Hz, 3H, H-9), 1.69–1.76 (m, 3H, H-5, H-6), 3.03–3.14 (m, 4H, H-4), 3.76 (q, $^3J_{8,9}$ = 7.1 Hz, 2H, H-8), 7.06 (m_c, 4H, H-1, H-2); **^{13}C NMR** (101 MHz, $CDCl_3$): δ 14.2 (C-9), 17.0 (C-5), 21.1 (C-6), 25.1 (C-4), 60.1 (C-8), 125.9 (C-1), 128.6 (C-2), 135.8 (C-3), 171.0 (C-7); **HRMS** (m/z): [(M+H)$^+$] calcd. for $C_{14}H_{17}O_2^+$, 217.1223; found, 217.1230; **IR** (ATR): 2979 (w), 2907 (w), 2340 (w), 1917 (w), 1715 (s), 1430 (m), 1369 (m), 1268 (m), 1148 (s), 1039 (m), 998 (m), 859 (m), 810 (m), 743 cm^{-1} (s).

The data is in accordance with literature[14,15].

1.6.3.1.4 (1s,1a*R*,7a*S*)-1a,2,7,7a-tetrahydro-1*H*-cyclopropa[b]naphthalene-1-carboxylic acid (*cis*-11)

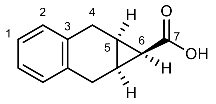

cis-**11**
$C_{12}H_{12}O_2$
M = 188.23 g mol^{-1}

A round-bottom flask was charged with ester *cis*-**10** (1.50 g, 6.94 mmol, 1.00 equiv) and EtOH (15 mL). Potassium hydroxide (1.17 g, 20.8 mmol, 3.00 equiv) was added and the reaction mixture was stirred under reflux for 11 h. After full conversion (monitored *via* TLC) H_2O (100 mL) was added, and the aqueous layer was washed with *tert*-butyl methyl ether (2 × 20 mL). Then the aqueous layer was acidified with conc. aqueous HCl solution and extracted with *tert*-butyl methyl ether (3 × 30 mL). The combined organic layer washed with brine and dried over Na_2SO_4. After filtration and removal of all volatiles under reduced pressure the crude product was purified *via* recrystallization in cyclohexane and EtOAc to afford acid *cis*-**11** as a colourless solid (1.25 g, 6.64 mmol, 96%). **TLC** (SiO_2, cyclohexane/EtOAc = 9:1, v/v): **R_f** = 0.14; **mp**: 150 °C; **^{1}H NMR** (400 MHz, $CDCl_3$): δ 1.74–1.78 (m, 1H, H-6), 1.80–1.85 (m, 2H, H-5), 2.92–3.07 (m, 4H, H-4), 7.11 (s, 4H, H-1, H-2), 10.9 (br s, O*H*); **^{13}C NMR** (101 MHz, $CDCl_3$): δ 19.3 (C-5), 21.0 (C-6), 24.2 (C-4), 125.9 (C-2), 128.4 (C-1), 136.6 (C-3) 177.4 (C-7). Carbon atom C-7 at

δ = 177.4 ppm was detected *via* HMBC; **HRMS** (m/z): [(M+H)$^+$] calcd. for $C_{12}H_{13}O_2^+$, 189.0910; found, 189.0909; **IR** (ATR): 3021 (w), 2930 (w), 2853 (w), 2694 (w), 2538 (w), 2342 (w), 2118 (w), 1908 (w), 1750 (w), 1680 (s), 1452 (s), 1346 (m), 1313 (w), 1232 (m), 1214 (s), 1194 (s), 1118 (m), 926 (s), 847 (m), 798 (m), 738 cm^{-1} (s).

1.6.3.1.5 Methyl 3-oxo-3-((1*s*,1a*R*,7a*S*)-1a,2,7,7a-tetrahydro-1*H*-cyclopropa[b]naphthalen-1-yl)propanoate (12a)

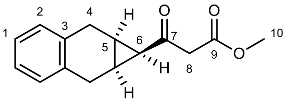

12a
$C_{15}H_{16}O_3$
M = 244.29 g mol^{-1}

Prepared according to **GP1** from carboxylic acid *cis*-**11** (1.00 g, 5.31 mmol, 1.00 equiv), *N*,*N*'-carbonyldiimidazole (862 mg, 5.31 mmol, 1.00 equiv), di-*iso*-propylamine (3.73 mL, 26.6 mmol, 5.00 equiv), *n*BuLi (2.91M in hexane, 9.12 mL, 26.6 mmol, 5.00 equiv) and methyl acetate (1.06 mL, 13.3 mmol, 2.50 equiv) in THF (100 mL). Purification *via* flash column chromatography on silica gel (4.5 × 13 cm, cyclohexane/EtOAc = 20:1, 20 mL, #76–140) afforded acetoacetate **12a** as a colourless oil (745 mg, 3.05 mmol, 57%). **TLC** (SiO_2, cyclohexane/EtOAc = 7:3, v/v): **R*f*** = 0.36; **^{1}H NMR** (400 MHz, $CDCl_3$): δ 1.96–2.00 (m, 2H, H-5), 2.13 (t, $^3J_{6,5}$ = 8.5 Hz, 1H, H-6), 2.86–3.03 (m, 4H, H-4), 3.52 (s, 2H, H-8), 3.72 (s, 3H, H-10) 7.09 (s, 4H, H-1, H-2); **^{13}C NMR** (101 MHz, $CDCl_3$): δ 22.6 (C-5), 23.9 (C-6), 28.5 (C-4), 51.8 (C-8), 52.3 (C-10), 125.9 (C-1), 128.4 (C-2), 136.5 (C-3) 168.0 (C-9), 200.7 (C-7); **HRMS** (m/z): [(M+H)$^+$] calcd. for $C_{15}H_{17}O_3^+$, 245.1172; found, 245.1170; **IR** (ATR): 3475 (w), 3357 (w), 3020 (w), 2919 (m), 2847 (w), 2318 (w), 2107 (w), 2082 (w), 1741 (s), 1678 (s), 1490 (m), 1398 (s), 1302 (s), 1258 (s), 1183 (m), 1140 (s), 1091 (s), 1036 (m), 1009 (s), 964 (s), 892 (m), 816 (s), 751 (s), 667 cm^{-1} (s).

1.6.3.1.6 *tert*-Butyl 3-oxo-3-((1*s*,1a*R*,7a*S*)-1a,2,7,7a-tetrahydro-1*H*-cyclopropa[b]naphthalen-1-yl)propanoate (12b)

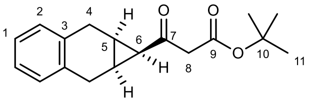

12b
$C_{18}H_{22}O_3$
M = 286.37 g mol^{-1}

Prepared according to **GP1** from carboxylic acid *cis*-**10** (1.00 g, 5.31 mmol, 1.00 equiv), *N*,*N*'-carbonyldiimidazole (947 mg, 5.84 mmol, 1.00 equiv), di-*iso*-propylamine (3.73 mL, 26.6 mmol, 5.00 equiv), *n*BuLi (2.61M in *n*-hexane, 10.2 mL, 26.6 mmol, 5.00 equiv) and *tert*-butyl acetate (1.79 mL, 13.3 mmol, 2.50 equiv) in THF (100 mL). Purification *via* flash column chromatography on silica gel (2.5 × 12 cm, cyclohexane/EtOAc = 20:1, 20 mL, #15–25) afforded acetoacetate **12c** as a colourless solid (1.06 g, 0.35 mmol, 70%). **TLC** (SiO_2, cyclohexane/EtOAc = 9:1, v/v): **R*f*** = 0.46; **mp**: 95–100 °C; **^{1}H NMR** (400 MHz, $CDCl_3$): δ 1.48 (s, 9H, H-11), 1.95–1.98 (m, 2H, H-5), 2.15 (t, $^3J_{6,5}$ = 8.2 Hz, 1H, H-6), 2.88–3.01 (m, 4H, H-4), 3.39 (s, 2H, H-8), 7.09 (s, 4H, H-1, H-2); **^{13}C NMR** (101 MHz, $CDCl_3$): δ 22.5 (C-5), 23.9 (C-4), 28.2 (C-11), 28.2 (C-6), 53.7 (C-8), 81.8 (C-10), 125.8 (C-1), 128.4 (C-2), 136.8 (C-3), 166.8 (C-9), 201.3 (C-7); **HRMS** (m/z): [(M–(C_4H_9)+2H)$^+$]: calcd. for $C_{14}H_{15}O_3^+$, 231.1016; found, 231.1016; **IR** (ATR): 2974 (m), 2927

(m), 2320 (w), 2107 (w), 1906 (w), 1719 (s), 1700 (s), 1452 (m), 1432 (m), 1393 (m), 1316 (s), 1271 (s), 1137 (s), 1086 (s), 1032 (s), 977 (s), 820 (s), 741 cm^{-1} (s).

1.6.3.1.7 Methyl-2-diazo-3-oxo-3-((*1s*,1a*R*,7a*S*)-1a,2,7,7a-tetrahydro-1*H*-cyclopropa[b]-naphthalen-1-yl)propanoate (13a)

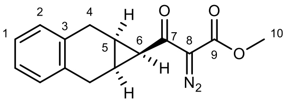

13a
$C_{15}H_{14}N_2O_3$
M = 270.29 g mol^{-1}

Prepared according to **GP2** from acetoacetate **12a** (740 mg, 3.03 mmol, 1.00 equiv), 4-acetamidobenzenesulfonyl azide (875 mg, 3.64 mmol, 1.2 equiv), triethylamine (0.51 mL, 3.64 mmol, 1.2 equiv) and MeCN (50 mL). Purification *via* flash column chromatography on silica gel (4 × 15 cm, cyclohexane/EtOAc = 30:1, 20 mL, #27–55) afforded diazoacetoacetate **13a** as a yellow oil (693 mg, 2.56 mmol, 85%). **TLC** (SiO_2, cyclohexane/EtOAc = 8:2, v/v): $\mathbf{R_f}$ = 0.50; **^{1}H NMR** (400 MHz, $CDCl_3$): δ 2.02 (m_c, 2H, H-5) 2.88 (t, $^3J_{6,5}$ = 8.4 Hz, 1H, H-6), 2.94–2.98 (m, 2H, H-4a), 3.02–3.08 (m, 2H, H-4b), 3.84 (s, 3H, H-10), 7.07 (m_c, 4H, H-1, H-2); **^{13}C NMR** (101 MHz, $CDCl_3$): δ 22.0 (C-5), 24.7 (C-4), 26.3 (C-6), 52.3 (C-10), 125.8 (C-1)*, 128.4 (C-2)*, 136.4 (C-3), 162.4 (C-9), 189.5 (C-7). Carbon atom C-8 was not detected; **HRMS** (m/z): [$(M+H)^+$] calcd. for $C_{15}H_{15}N_2O_3^+$, 271.1077; found, 271.1074; **IR** (ATR): 3421 (w), 3015 (w), 2912 (w), 2339 (w), 2125 (s), 1901 (w), 1704 (s), 1633 (s), 1491 (m), 1431 (s), 1300 (s), 1206 (s), 1126 (s), 1072 (s), 1006 (s), 894 (m), 802 (m), 739 cm^{-1} (s).

1.6.3.1.8 *tert*-Butyl 2-diazo-3-oxo-3-((1*s*,1a*R*,7a*S*)-1a,2,7,7a-tetrahydro-1*H*-cyclopropa[b]naphthalen-1-yl)propanoate (13b)

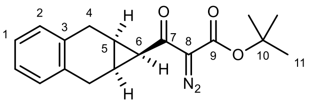

13b
$C_{18}H_{20}N_2O_3$
M = 312.377 g mol^{-1}

Prepared according to **GP2** from acetoacetate **12b** (1.00 g, 3.90 mmol, 1.00 equiv), 4-acetamidobenzenesulfonyl azide (1.12 g, 4.68 mmol, 1.20 equiv), triethylamine (0.65 mL, 4.68 mmol, 1.20 equiv) and MeCN (50 mL). Purification *via* flash column chromatography on silica gel (4.5 × 15 cm, cyclohexane/EtOAc = 30:1, 20 mL, #26–36) afforded diazoacetoacetate **13c** as a yellow solid (912 mg, 2.92 mmol, 75%). **TLC** (SiO_2, cyclohexane/EtOAc = 8:2, v/v): $\mathbf{R_f}$ = 0.49; **mp**: 120–125 °C; **^{1}H NMR** (400 MHz, $CDCl_3$): δ 1.55 (s, 9H, H-11), 1.99 (m_c, 2H, H-5), 2.86 (t, $^3J_{6,5}$ = 8.5 Hz, 1H, H-6), 2.95–3.08 (m, 4H, H-4), 7.07 (s, 4H, H-1, H-2); **^{13}C NMR** (101 MHz, $CDCl_3$): δ 21.9 (C-5), 24.8 (C-4), 26.2 (C-6), 28.5 (C-11), 83.0 (C-10), 125.7 (C-1), 128.4 (C-2), 136.5 (C-3), 161.3 (C-9), 190.1 (C-7). Carbon atom C-8 was not detected. **HRMS** (m/z): [$(M+H)^+$] calcd. for $C_{18}H_{21}N_2O_3^+$, 313.1547; found, 313.1548; **IR** (ATR): 2976 (w), 2918 (w), 2321 (w), 2129 (s), 1908 (w), 1697 (s), 1624 (s), 1454 (w), 1401 (m), 1302 (s), 1188 (s), 1128 (s), 1070 (s), 968 (s), 837 (m), 748 cm^{-1} (s).

1.6.3.1.9 Triasterane 14a

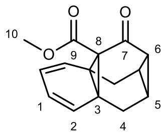

14a
$C_{15}H_{14}O_3$
M = 242.27 g mol^{-1}

Prepared according to **GP3** from diazoacetoacetate **13a** (270 mg, 1.00 mmol, 1.00 equiv), copper(II) hexafluoroacetylacetonate hydrate (49 mg, 0.10 mmol, 10 mol%) and CH_2Cl_2 (100 mL). Purification *via* flash column chromatography on silica gel (2.5 × 13 cm, cyclohexane/EtOAc = 4:1, 20 mL, #8–15) afforded triasterane **14a** as a colourless solid (195 mg, 805 µmol, 81%). **TLC** (SiO_2, cyclohexane/EtOAc = 8:2, v/v): **R*f*** = 0.24; **mp**: 160 °C; **^{1}H NMR** (400 MHz, $CDCl_3$): δ 1.86 (dd, $^3J_{5,6}$ = 8.2 Hz, $^3J_{5,4b}$ = 1.6 Hz, 2H, H-5), 2.06 (t, $^3J_{6,5}$ = 8.2 Hz, 1H, H-6), 2.34 (d, $^2J_{4a,4b}$ = 13.8 Hz, 2H, H-4a)*, 2.44 (dt, $^2J_{4b,4a}$ = 13.8 Hz, $^3J_{4b,5}$ = 1.6 Hz, 2H, H-4b)*, 3.56 (s, 3H, H-10), 5.90–5.97 (m_c, 4H, H-1, H-2); **^{13}C NMR** (101 MHz, $CDCl_3$): δ 22.7 (C-5), 23.2 (C-4), 26.5 (C-6), 41.3 (C-8), 45.9 (C-3), 52.1 (C-10), 122.8 (C-1), 128.9 (C-2), 165.3 (C-9), 202.7 (C-7); **HRMS** (m/z): [(M+H)$^+$] calcd. for $C_{15}H_{15}O_3^+$, 243.1016; found, 243.1015; **IR** (ATR): 2907 (w), 2107 (w), 1731 (s), 1639 (s), 1458 (m), 1359 (m), 1288 (m), 1246 (s), 1129 (m), 1080 (m), 1029 (s), 966 (m), 904 (m), 852 (m), 735 cm^{-1} (s).

1.6.3.1.10 Triasterane 14b

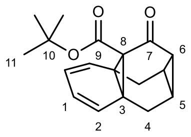

14b
$C_{18}H_{20}O_3$
M = 242.27 g mol^{-1}

Prepared according to **GP3** from diazoacetoacetate **13b** (500 mg, 1.60 mmol, 1.00 equiv), copper(II) hexafluoroacetylacetonate hydrate (76 mg, 0.10 mmol, 10 mol%) and CH_2Cl_2 (160 mL). Purification *via* flash column chromatography on silica gel (3 × 13 cm, cyclohexane/EtOAc = 7:3, 20 mL, #14–30) afforded triasterane **14b** as a colourless solid (250 mg, 879 µmol, 55%). **TLC** (SiO_2, cyclohexane/EtOAc = 8:2, v/v): **R*f*** = 0.11; **mp**: 168 °C; **^{1}H NMR** (400 MHz, $CDCl_3$): δ 1.34 (s, 9H, H-11), 1.83 (d, $^3J_{5,6}$ = 8.2 Hz, 2H, H-5), 2.03 (t, $^3J_{6,5}$ = 8.2 Hz, 1H, H-6), 2.31 (d, $^2J_{4a,4b}$ = 13.8 Hz, 2H, H-4a)*, 2.51 (d, $^2J_{4b,4a}$ = 13.8 Hz, 2H, H-4b)*, 5.87–5.90 (m, 2H, H-2), 5.94–5.97 (m, 2H, H-1); **^{13}C NMR** (101 MHz, $CDCl_3$): δ 22.6 (C-5), 23.2 (C-4), 26.6 (C-6), 28.0 (C-11), 41.3 (C-8), 45.6 (C-3), 81.1 (C-10), 122.8 (C-1), 128.9 (C-2), 164.0 (C-9), 203.3 (C-7). **HRMS** (m/z): [(M+H)$^+$] calcd. for $C_{18}H_{21}O_3^+$, 285.1485; found, 285.1485; **IR** (ATR): 3034 (w), 2970 (w), 2922 (w), 2257 (w), 2123 (w), 1731 (s), 1656 (s), 1354 (m), 1248 (s), 1158 (s), 1084 (s), 1029 (s), 920 (m), 880 (m), 820 (m), 732 cm^{-1} (s).

1.6.3.1.11 Methyl 1-oxo-1,2-dihydro-9a*H*-2,9-ethenobenzo[7]annulene-9a-carboxylate (15)

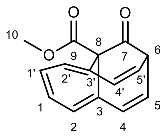

15
$C_{15}H_{12}O_3$
M = 240.26 g mol^{-1}

A pressure tube was charged with triasterane **14a** (200 mg, 826 µmol, 1.00 equiv) and chlorobenzene (17 mL). 2,3-Dichloro-5,6-dicyano-*p*-benzoquinone (375 mg, 1.65 mmol, 2.00 equiv) was added and the pressure tube was closed. The reaction mixture was stirred at 130 °C for 3 h. After full conversion (monitored *via* TLC) the reaction mixture was filtered over a plug of

silica (4 × 5 cm, eluent: CH_2Cl_2, 100 mL). After removal of all volatiles under reduced pressure the crude product was purified *via* flash column chromatography on silica gel (2.5 × 12 cm, cyclohexane/EtOAc = 20:1, 20 mL, #23–55) to afford annulene **15** as a yellow solid (63 mg, 262 mmol, 32%). **TLC** (SiO_2, cyclohexane/EtOAc = 6:4, v/v): $\mathbf{R_f}$ = 0.53, **mp**: 165 °C; **^{1}H NMR** (400 MHz, $CDCl_3$): δ 3.78 (s, 3H, H-10), 3.81 (pseudo t, $^3J_{6,5}$ = 6.5 Hz, $^3J_{6,5'}$ = 6.5 Hz, 1H, H-6), 5.31 (dd, $^3J_{5,4}$ = 9.0 Hz, $^3J_{5,6}$ = 6.5 Hz, 1H, H-5), 5.35 (dd, $^3J_{5',4'}$ = 8.9 Hz, $^3J_{5',6}$ = 6.5 Hz, 1H, H-5’), 6.70 (d, $^3J_{4',5'}$ = 8.9 Hz, 1H, H-4’), 6.71 (d, $^3J_{4,5}$ = 9.0 Hz, 1H, H-4), 6.75 (d, $^3J_{2,1}$ = 6.7 Hz, 1H, H-2)*, 6.83 (d, $^3J_{2',1'}$ = 6.7 Hz, 1H, H-2’)*, 6.96 (dd, $^3J_{1,1'}$ = 10.7 Hz, $^3J_{1,2}$ = 6.7 Hz, 1H, H-1)**, 7.06 (dd, $^3J_{1',1}$ = 10.7 Hz, $^3J_{1',2'}$ = 6.7 Hz, 1H, H-1’)**; **^{13}C NMR** (101 MHz, $CDCl_3$): δ 45.3 (C-6), 52.1 (C-10), 61.6 (C-8), 111.0 (C-5), 111.3 (C-5’), 121.6 (C-3), 121.8 (C-3’), 123.9 (C-2)*, 124.0 (C-2’)*, 127.4 (C-4’), 127.4 (C-1)*, 127.9 (C-4), 129.5 (C-1’)*, 166.5 (C-9), 203.0 (C-7); **HRMS** (m/z): [(M+H)$^+$] calcd. for $C_{15}H_{13}O_3^+$, 241.0859; found, 241.0858; **IR** (ATR): 3446 (w), 2947 (w), 2339 (w), 2339 (w), 2220 (w), 2088 (w), 1912 (w), 1724 (s), 1436 (m), 1353 (w), 1237 (s), 1113 (m), 1066 (s), 933 (s), 831 (s), 788 (s) 738 cm^{-1} (s).

Note: During the publication process it was found that DMF is the superior solvent compared to chlorobenzene in the oxidative dehydrogenation and should be used instead.

1.6.3.1.12 2-Diazo-1-((1*s*,1a*R*,7a*S*)-1a,2,7,7a-tetrahydro-1*H*-cyclopropa[b]naphthalen-1-yl)ethan-1-one (S10)

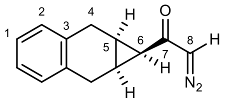

S10
$C_{13}H_{12}N_2O$
M = 212.25 g mol^{-1}

A Schlenk flask was charged with carboxylic acid *cis*-**11** (500 mg, 2.66 mmol, 1.00 equiv), dissolved in CH_2Cl_2 (20 mL) and cooled down to 0 °C. Oxalyl chloride (161 mg, 1.27 mmol, 1.20 equiv) and DMF (1 drop) were added, and the reaction mixture was stirred at 0 °C for 2 h. All volatiles were removed under reduced pressure and the residue was dissolved in MeCN (10 mL). A separate Schlenk flask was charged with trimethylsilyl diazomethane (2 M in hexane, 3.99 mL, 7.98 mmol, 3.00 equiv) and MeCN (10 mL) and cooled down to 0 °C. The acid chloride solution was added dropwise to the trimethylsilyl diazomethane solution and stirred at 0 °C for 1 h and at room temperature for 1 h. After removal of all volatiles under reduced pressure *via* the Schlenk line the crude product was purified *via* flash column chromatography on silica gel (3.5 × 15 cm, cyclohexane/EtOAc = 20:1 (#1–50) → 10:1 (#51-100), 20 mL, #59–73) to afford diazoketone **S10** as a yellow solid (382 mg, 1.80 mmol, 68%). **TLC** (SiO_2, cyclohexane/EtOAc = 8:2, v/v): $\mathbf{R_f}$ = 0.33; **mp**: 99 °C; **^{1}H NMR** (400 MHz, $CDCl_3$): δ 1.71–1.78 (m, 1H, H-6) 1.80–1.83 (m, 2H, H-5), 3.07–3.08 (m, 4H, H-4), 5.31 (br s, 1H, H-8), 7.05–7.10 (m, 4H, H-1, H-2); **^{13}C NMR** (101 MHz, $CDCl_3$): δ 20.0 (C-5), 24.9 (C-4), 27.2 (C-6), 56.2 (C-8), 125.9 (C-1)*, 128.4 (C-2)*, 136.3 (C-3), 192.2 (C-7); **HRMS** (m/z): [(M+H)$^+$] calcd. for $C_{13}H_{13}N_2O^+$, 213,1022; found, 213,1022; **IR** (ATR): 3213 (w), 3084 (m), 3003 (w), 2913 (w),

2623 (w), 2371 (w), 2263 (w), 2091 (s), 1918 (w), 1608 (s), 1491 (m), 1410 (s), 1314 (s), 1089 (s), 1089 (s), 1045 (s), 965 (s), 941 (m), 864 (m), 808 (m), 744 cm^{-1} (s).

1.6.3.1.13 Triasterane S11

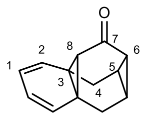

S11
$C_{13}H_{12}O$
M = 184.24 g mol^{-1}

A Schlenk tube was charged with $Rh_2(OAc)_4$ (22 mg, 50 µmol, 10 mol%) and dissolved in CH_2Cl_2 (20 mL). A solution of diazoketone **S10** (106 mg, 0.50 mmol, 1.00 equiv) and CH_2Cl_2 (5 mL) was slowly added to the catalyst solution *via* a syringe pump at room temperature over 5 h. After complete addition of the diazoketone solution all volatiles were removed under reduced pressure and the crude product was purified *via* flash column chromatography on silica gel (2 × 11 cm, cyclohexane/EtOAc = 8:1, 20 mL, #10–25) to afford ketone **S11** as a colourless solid (71 mg, 0.39 mmol, 77%). **TLC** (SiO_2, cyclohexane/EtOAc = 8:2, v/v): $\mathbf{R_f}$ = 0.13; **mp**: 135 °C; **^{1}H NMR** (400 MHz, $CDCl_3$): δ 0.87 (d, $^4J_{8,6}$ = 2.12 Hz, 1H, H-8), 1.81–1.83 (m, 2H, H-5), 1.92 (m_c, 1H, H-6), 2.32 (d, $^2J_{4a,4b}$ = 13.8 Hz, 2H, H-4a)*, 2.44 (d, $^2J_{4a,4b}$ = 13.5 Hz, 2H, H-4b)*, 5.86–5.90 (m, 2H, H-1), 5.94–5.98 (m_c, 2H, H-2); **^{13}C NMR** (101 MHz, $CDCl_3$): δ 23.0 (C-4), 23.5 (C-5), 27.0 (C-6), 34,7 (C-8), 43,8 (C-3), 121.6 (C-1), 130.8 (C-2), 208.2 (C-7); **HRMS** (m/z): [(M+H)$^+$] calcd. for $C_{13}H_{13}O^+$, 185.0961; found, 185.0960; **IR** (ATR): 3261 (w), 3035 (m), 2902 (m), 2846 (m), 2321 (w), 2111 (w), 1902 (w), 1845 (w), 1628 (s), 1426 (m), 1397 (m), 1348 (s), 1271 (m), 1199 (m), 1141 (m), 1030 (m), 989 (s), 899 (s), 830 (s), 720 cm^{-1} (s).

1.6.3.1.14 Methyl-2-(2a1,2b,3,7b-tetrahydrobenzo[g]cyclopropa[cd]benzofuran-2(2a*H*)-ylidene)acetate (tentative structure, S12)

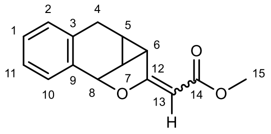

S12
$C_{15}H_{14}O_3$
M = 242.27 g mol^{-1}

A pressure tube was charged with triasterane **14a** (12.1 mg, 50.0 µmol, 1.00 equiv) and chlorobenzene (1 mL). Pd/C (10 wt%, 5.3 mg, 5.0 µmol, 10 mol%) was added and the pressure tube was closed. The reaction mixture was stirred at 150 °C for 35 h. After full conversion (monitored *via* TLC) the reaction mixture was filtered over a plug of silica (0.5 × 5 cm, eluent: CH_2Cl_2, 20 mL). After removal of all volatiles under reduced pressure ester **S12** was afforded a yellow oil (10.1 mg, 41.7 µmol, 83%). **TLC** (SiO_2, cyclohexane/EtOAc = 6:4, v/v): $\mathbf{R_f}$ = 0.68; **^{1}H NMR** (400 MHz, $CDCl_3$): δ 2.01 (m_c, 1H, H-5), 2.37 (dd, $^2J_{4a,4b}$ = 17.0 Hz, $^3J_{4a,5}$ = 3.3 Hz, 1H, H-4a), 2.52 (m_c, 1H, H-7), 3.17 (dd, $^2J_{4b,4a}$ = 17.0 Hz, $^3J_{4b,5}$ = 8.6 Hz, 1H, H-4b), 3.59 (dd, $^3J_{6,5}$ = 8.6 Hz, $^3J_{6,7}$ = 6.2 Hz, 1H, H-6), 3.70 (s, 3H, H-15), 5.32 (s, 1H, H-13), 5.60 (d, $^3J_{8,7}$ = 5.2 Hz, 1H, H-8), 7.18–7.21 (m, 2H, H-2, H-11), 7.26–7.30 (m, 2H, H-1, H-10); **^{13}C NMR** (101 MHz, $CDCl_3$): δ 19.9 (C-5), 24.3 (C-4), 26.5 (C-7), 29.9 (C-6), 50.9 (C-15), 81.7 (C-8), 89.7 (C-13), 126.3 (C-11), 128.2 (C-10), 128.5 (C-2), 129.3 (C-1), 134.4 (C-9), 138.1 (C-3), 169.5 (C-14), 174.6 (C-12). **HRMS** (m/z): [(M+H)$^+$] calcd. for $C_{15}H_{13}O_3^+$, 243.1016; found, 243.1014.

1.6.3.2 Follow-Up

1.6.3.2.1 Saponification

1.6.3.2.1.1 1-Oxo-1,9a-dihydro-2-2,9-ethenobenzo[7]annulene-2-carboxylic acid (16)

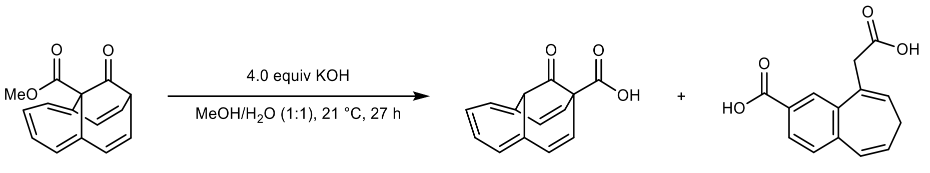

15 **16** 22% **S9**

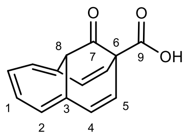

16
$C_{14}H_{10}O_3$
M = 226.23 g mol^{-1}

A Schlenk tube was charged with annulene **15** (30.0 mg, 125 µmol, 1.00 equiv) and MeOH/H_2O (1:1, 1 mL). Then potassium hydroxide (21 mg, 375 µmol, 3.00 equiv) was added and the reaction mixture was stirred for 24 h. Additional potassium hydroxide (7.00 mg, 125 µmol, 1.00 equiv) was added and the reaction was stirred for 3 h. After full conversion (monitored *via* TLC) H_2O (5 mL) was added and the aqueous layer was washed with *tert*-butyl methyl ether (3 × 2 mL). The aqueous layer was acidified with aqueous HCl-solution (2M) and then extracted with EtOAc (5 × 2 mL). The combined organic layer was washed with brine and dried over Na_2SO_4. After filtration and removal of all volatiles under reduced pressure the crude product purified *via* recrystallization from EtOAc to afford carboxylic acid **16** (6.0 mg, 27 µmol, 22%) as a colourless solid. X-ray crystal structure of side product **S9** was isolated (see Supporting Information Section 2.1.8). **TLC** (SiO_2, cyclohexane/EtOAc = 6:4, v/v): **R_f** = 0.06; **^{1}H NMR** (400 MHz, $CDCl_3$): δ 0.86 (s, 1H, H-8), 5.61 (d, $^3J_{5,4}$ = 8.9 Hz, 2H, H-5), 6.75 (m_c, 2H, H-2), 6.79 (d, $^3J_{4,5}$ = 8.9 Hz, 2H, H-4), 7.17 (m_c, 2H, H-1). The ^{1}H NMR spectrum contains signals of *tert*-butyl methyl ether; **^{13}C NMR** (101 MHz, $CDCl_3$): δ 48.7 (C-8), 58.0 (C-6), 115.7 (C-5), 121.1 (C-3), 123.1 (C-2), 126.8 (C-4), 129.1 (C-1), 176.4 (C-9), 205.6 (C-7). Carbon atom C-9 and C-7 were detected *via* 1H, ^{13}C HMBC NMR. The ^{13}C NMR spectrum contains signals of *tert*-butyl methyl ether; **HRMS** (m/z): [(M+H)$^+$] calcd. for $C_{14}H_{11}O_3^+$, 227.0703; found, 227.0707.

1.6.3.2.2 Irradiation experiments

1.6.3.2.2.1 Methyl 10-oxo-9,10-dihydro-10a*H*-1,9-(metheno)heptalene-10a-carboxylate (18)

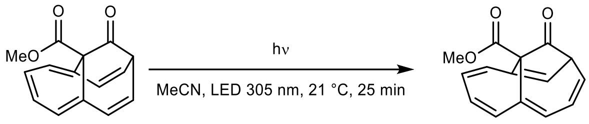

15 **18**

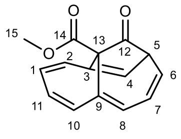

18
$C_{15}H_{12}O_3$
M = 240.26 g mol^{-1}

A quartz cuvette was charged with 2.5 mL of a prior prepared degassed solution of annulene **15** (4.97 mg, 20.7 µmol in 5 mL MeCN). The cuvette (2.5 mL) was then irradiated with a 305 nm LED (LG Innotek, FWHM = 13 nm) for 25 min. This procedure was repeated and the resulting solutions were combined. The crude product was purified *via* preparative HPLC. After removal of all volatiles under reduced pressure the ester **18** was afforded as a yellow solid (3.2 mg, 13 µmol, 64%). The product is sensitive to air and light. **^{1}H NMR** (500 MHz, CD_2Cl_2): δ 3.56 (s, 3H, H-15), 3.70 (dd, $^3J_{5,6}$ = 8.2 Hz, $^3J_{5,4}$ = 2.3 Hz, 1H, H-5), 4.48 (d, $^3J_{4,5}$ = 2.3 Hz, 1H, H-4), 5.53 (dd, $^3J_{6,7}$ = 10.8 Hz, $^3J_{6,5}$ = 8.2 Hz, 1H, H-6), 6.04 (dd, $^3J_{11,10}$ = 11.6 Hz, $J_{11,1}$ = 7.8 Hz, 1H, H-11), 6.10 (d, $^3J_{8,7}$ = 8.2 Hz, 1H, H-8), 6.18 (dd, $^3J_{1,2}$ = 11.4 Hz, $^3J_{1,11}$ = 7.8 Hz, 1H, H-1), 6.19 (dd, $^3J_{7,6}$ = 10.8 Hz, $^3J_{7,8}$ = 8.2 Hz, 1H, H-7), 6.72 (d, $^3J_{2,1}$ = 11.4 Hz, 1H, H-2), 6.97 (d, $^3J_{10,11}$ = 11.6 Hz, 1H, H-10); **^{13}C NMR** (125 MHz, CD_2Cl_2): δ 52.1 (C-5), 52.6 (C-15), 68.0 (C-13), 93.6 (C-4), 120.1 (C-6), 121.0 (C-8), 122.5 (C-11), 124.0 (C-7), 127.4 (C-1), 129.2 (C-2), 133.7 (C-3), 134.4 (C-9), 135.3 (C-10), 167.9 (C-14), 205.8 (C-12); **^{1}H NMR** (600 MHz, $CDCl_3$): δ 3.62 (s, 3H, H-15), 3.73 (dd, $^3J_{5,6}$ = 8.2 Hz, $^3J_{5,4}$ = 2.3 Hz, 1H, H-5), 4.47 (d, $^3J_{4,5}$ = 2.3 Hz, 1H, H-4), 5.52 (dd, $^3J_{6,7}$ = 11.7 Hz, $^3J_{6,5}$ = 7.9 Hz, 1H, H-6), 6.06 (dd, $^3J_{11,10}$ = 11.6 Hz, $J_{11,1}$ = 7.8 Hz, 1H, H-11), 6.11 (dd, $^3J_{8,7}$ = 8.2 Hz, $^3J_{8,10}$ = 1.0 Hz, 1H, H-8), 6.19 (m_c, 2H, H-1, H-7), 6.74 (d, $^3J_{2,1}$ = 11.4 Hz, 1H, H-2), 6.99 (d, $^3J_{10,11}$ = 11.7 Hz, 1H, H-10); **^{13}C NMR** (125 MHz, $CDCl_3$): δ 51.7 (C-5), 52.7 (C-15), 67.7 (C-13), 93.2 (C-4), 119.8 (C-6), 121.0 (C-8), 122.3 (C-11), 123.9 (C-7), 127.3 (C-1), 128.9 (C-2), 133.3 (C-3), 134.0 (C-9), 135.0 (C-10), 167.8 (C-14), 205.5 (C-12). **HRMS** (m/z): [(M+H)$^+$] calcd. for $C_{15}H_{12}O_3^+$, 241.0859; found, 241.0861.

1.7 UV/vis Absorption Experiments

In the following, a full irradiation cycle (forth and back irradiation) refers to the irradiation of compound **15** using a 305 nm LED until the photostationary state of **15** and **18** was reached. Afterwards, this solution was irradiated with a 455 nm LED until no **18** could be observed in the UV/vis absorption trace of the HPLC chromatogram. All UV/vis experiments were conducted at 25 °C if not stated otherwise.

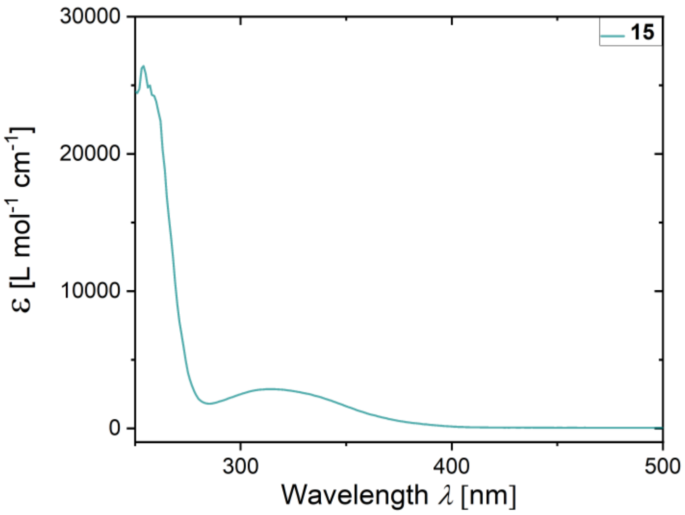

Supplementary Figure 7. UV/vis spectrum (molar extinction coefficient ε) of compound **15** in MeCN (c(**15**) = 1.1 × 10^{-4} mol L^{-1}).

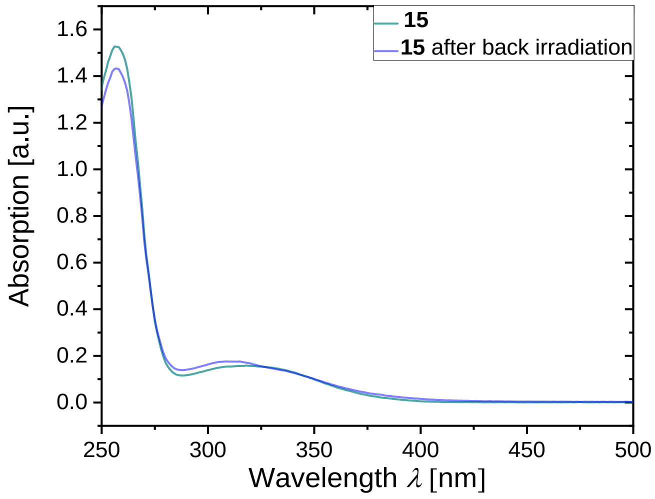

Supplementary Figure 8. UV/vis-absorption of compound **15** in cyclohexane (c(**15**) = 3.7 × 10^{-5} mol L^{-1}) before irradiation and after one full irradiation cycle.

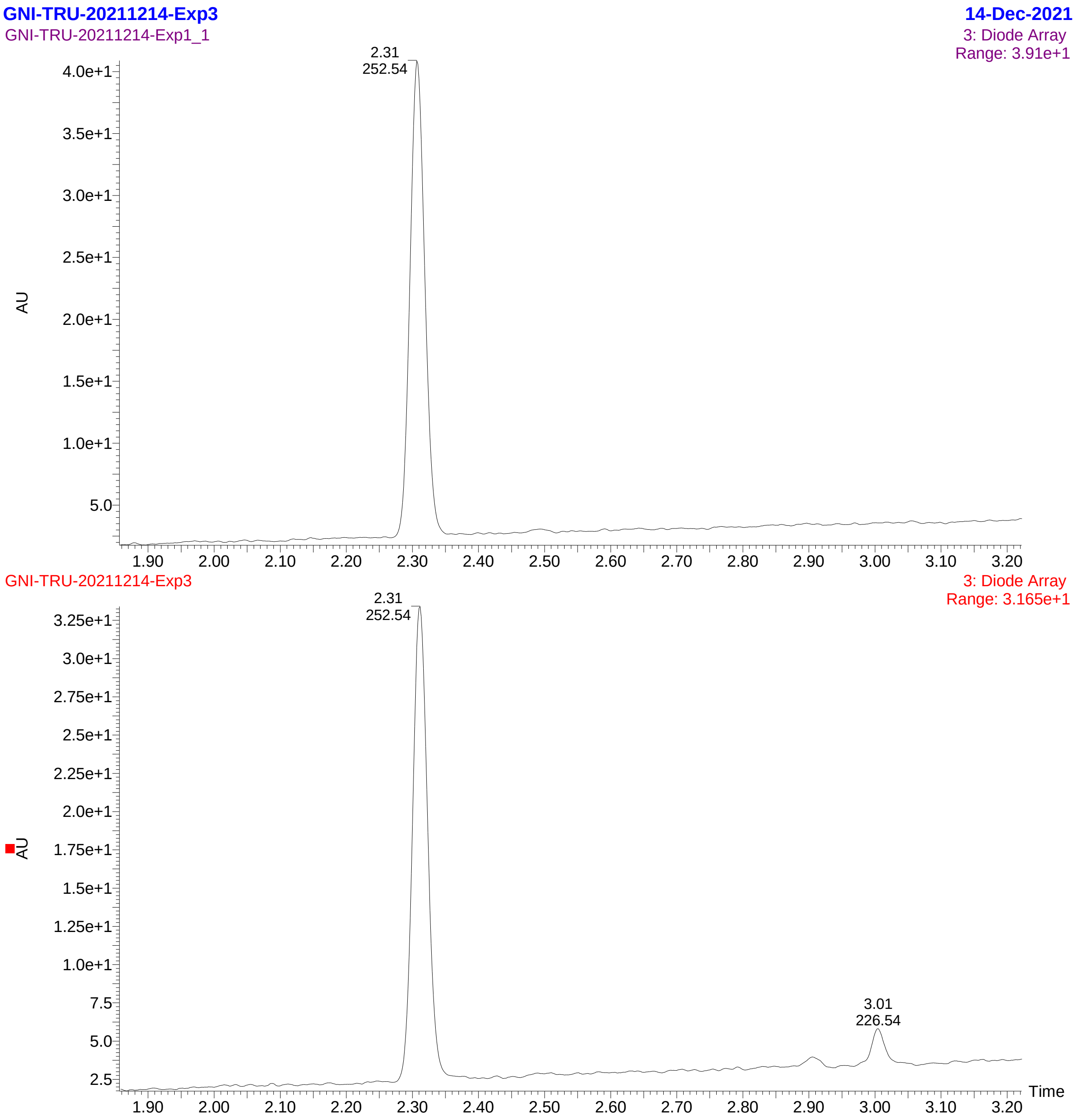

Supplementary Figure 9. UV/vis absorption (300 nm – 650 nm) trace of the HPLC chromatogram obtained from the spectroscopy cyclohexane solutions of Supplementary Figure 8. Before irradiation (top) and after one irradiation cycle (bottom). t_R = 3.01 min: minor unknown impurity from the irradiation cycle.

1.7.1 Switching in polar medium: MeCN

The photoswitching experiments were further conducted in MeCN as an alternative solvent with increased polarity as compared to the initially studied cyclohexane medium (see main text). This control experiment further indicated a non-polar transition state as is expected for a pericyclic [1,11] sigmatropic shift of a hydrocarbon scaffold. The reaction kinetics are approximately identical to the irradiation experiments in cyclohexane. (Supplementary Figure 8–9)

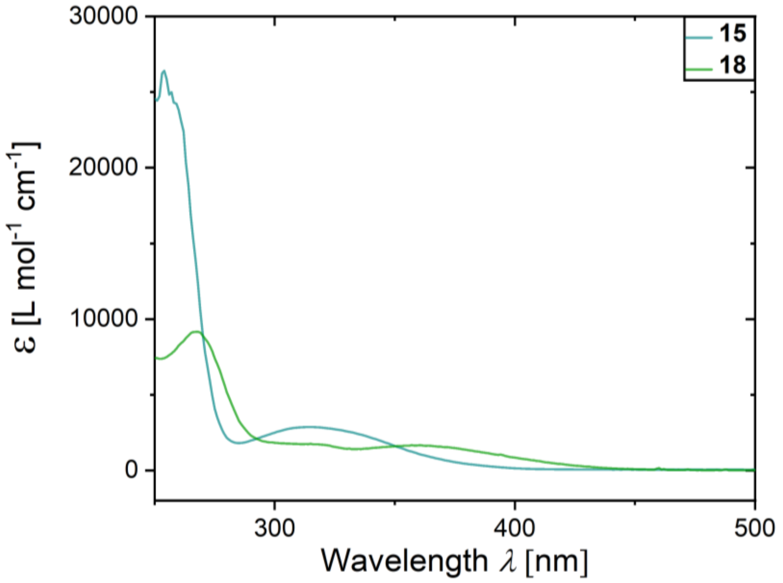

Supplementary Figure 10. UV/vis absorption of **15** in MeCN ($c = 7.8\times10^{-6}$ mol L^{-1}) before irradiation (cyan graph) and of isolated **18** (green graph) after irradiation ($c = 1.1\times10^{-4}$ mol L^{-1}).The molar absorptivity is equal at 350 nm for **15** and **18**, which is the analytical wavelengths of choice for further analysis of the UV/vis traces in the HPLC analysis.

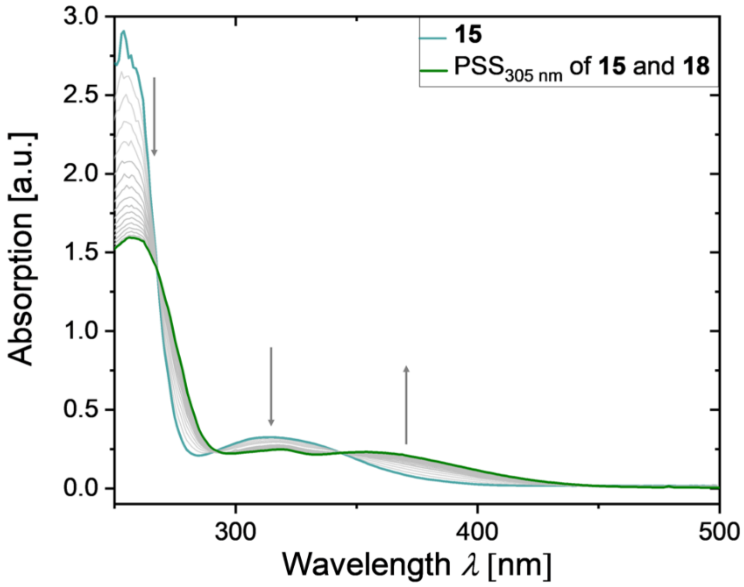

Supplementary Figure 11. UV/vis absorption of **15** in MeCN (c(**15**) = 1.1×10^{-4} mol L^{-1}) before irradiation (cyan graph), upon irradiation with a 305 nm LED with 5 s time intervalls (grey graphs) and at the PSS consisting of **15** and **18** (green graph) after total irradiation time of 80 s.

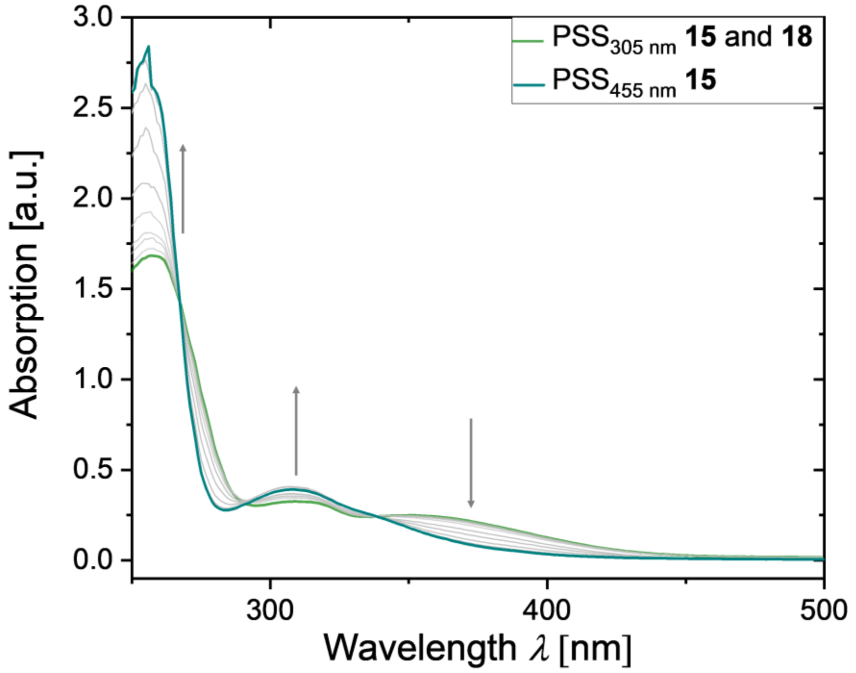

Supplementary Figure 12. UV/vis absorption of **15** and **18** in MeCN (c_{start}(**15**) = 1.1×10^{-4} mol L^{-1}) at the PSS$_{305\ nm}$ (green graph), upon irradiation with a 455 nm LED for 2 s, 12 s, 22 s, 42 s, 102 s, 322 s, 564 s 1044 s (17.4 min) (grey graphs) and at the PSS$_{455\ nm}$ consisting only of of **15** according to the HPLC analysis (cyan graph).

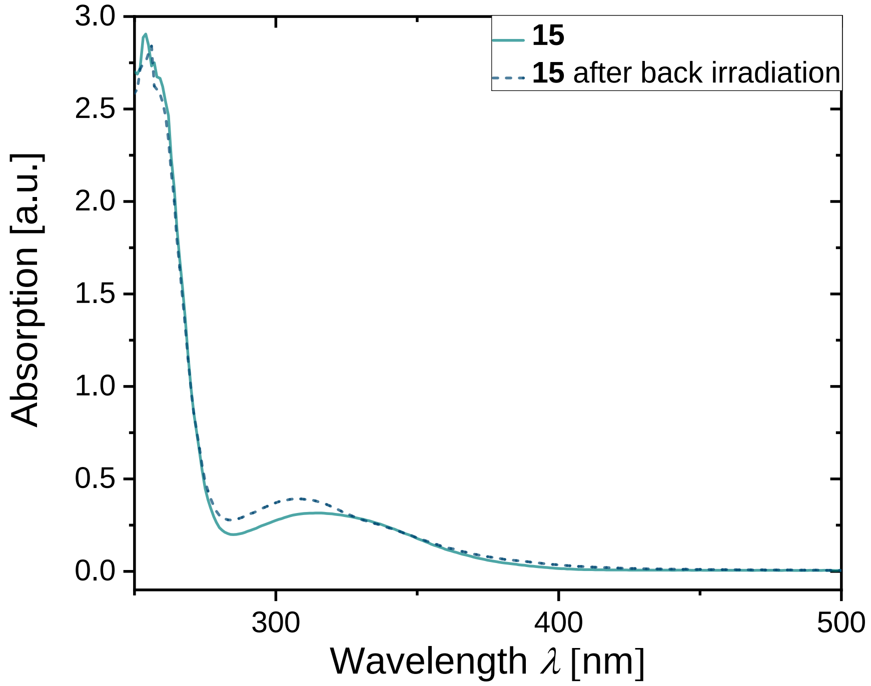

Supplementary Figure 13. UV/vis absorption of **15** in MeCN (c_{start}(**15**) = 1.1×10^{-4} mol L^{-1}) before irradiation (cyan graph) and after one full irradiation cycle (dashed cyan graph).

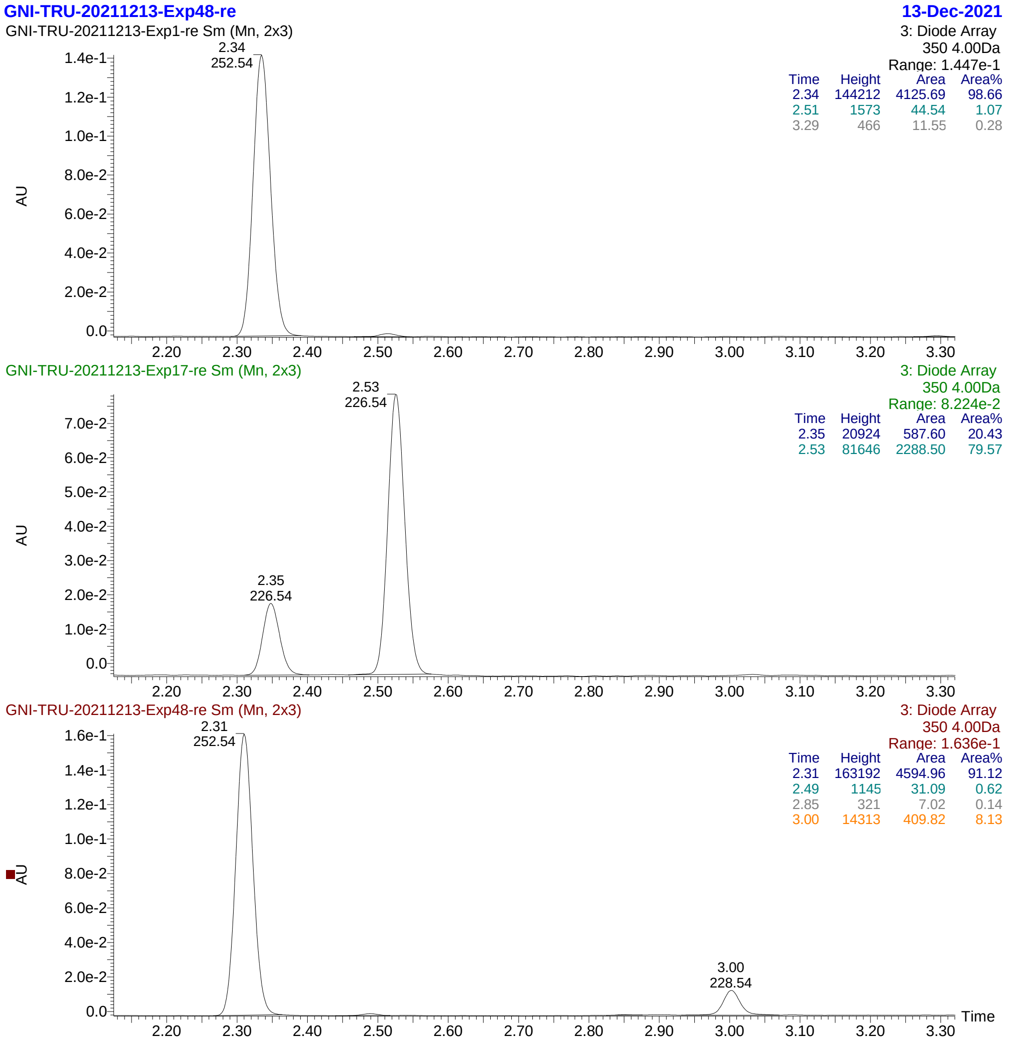

Supplementary Figure 14. UV/vis absorption trace (at 350 nm) of the HPLC chromatogram obtained from the spectroscopy MeCN solutions of Supplementary Figure 11 & 12. Before irradiation (top), at the $PSS_{305\ nm}$ and after one irradiation cycle ($PSS_{455\ nm}$ bottom). The composition at the $PSS_{305\ nm}$ in MeCN is **15**/**18** 20:80 based on the integration of the HPLC 350nm-UV/vis trace (middle graph).

1.7.2 Photoswitch-Cycling monitored by HPLC (in MeCN)

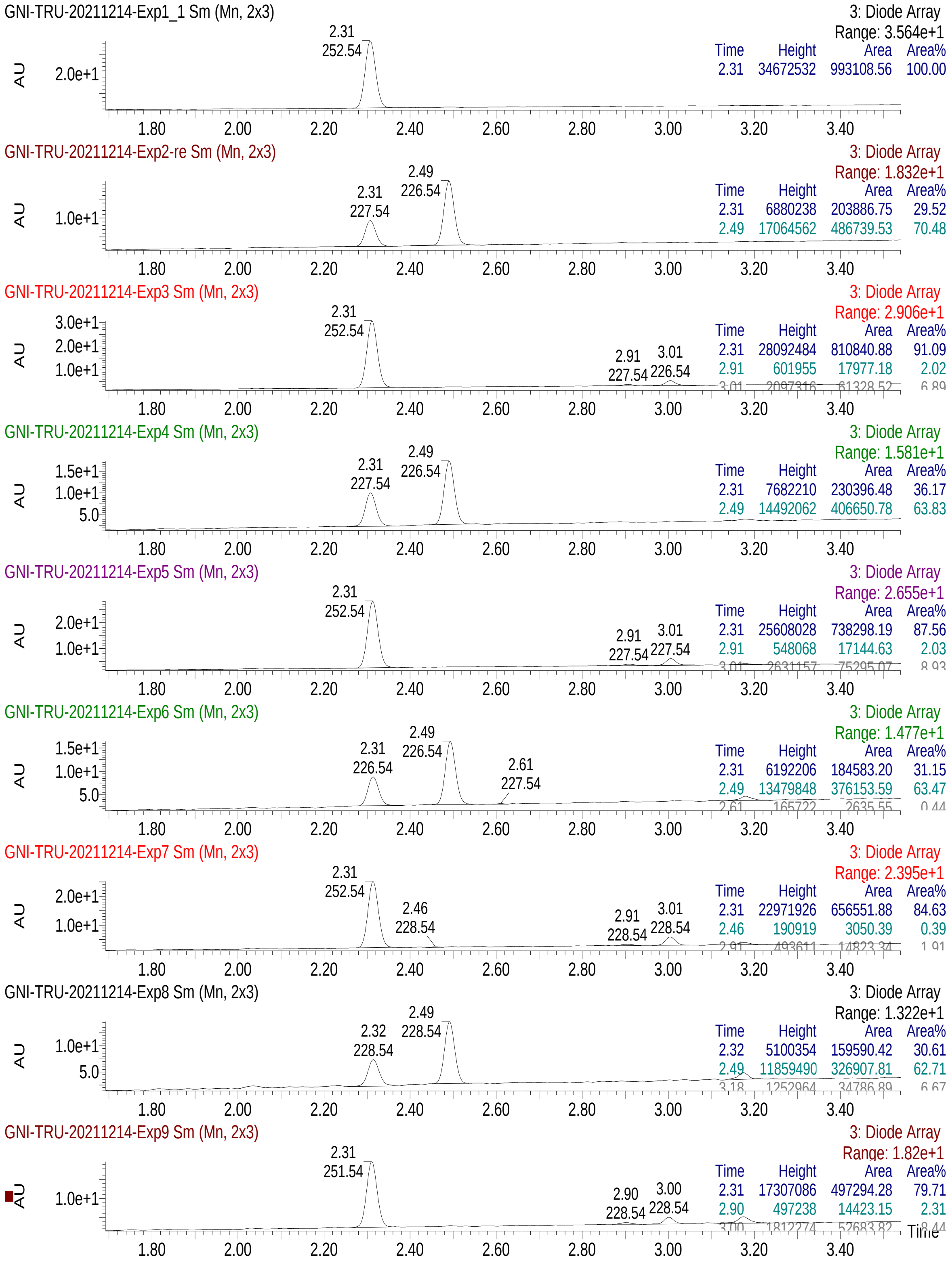

Supplementary Figure 15. UV/vis absorption trace of the HPLC chromatogram obtained from the cycling experiments conducted with **15** in MeCN. Top chromatogram before irradiation, second chromatogram after irradiation with a 305 nm LED, third chromatogram after irradiation with 455 nm continuing till the last chromatogram presenting the end of the fourth cycle. The sum of absorption (300–650 nm) at the UV/vis absorption diode detector was used as analytical probe (ordinate).

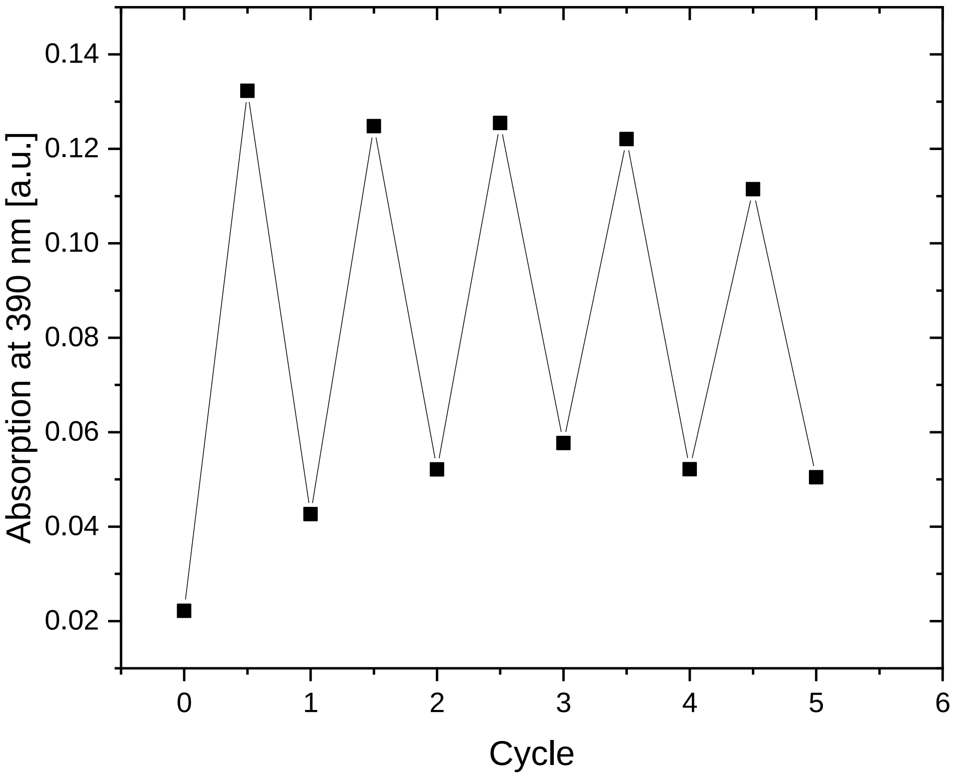

Supplementary Figure 16. UV/vis absorption at 390 nm of the spectroscopy (MeCN) solutions of the photo-cycling experiments of **15**/**18**.

1.7.3 Thermal Stability Test

Testing the thermal stability at the reached PSS leads to the classification of a “type *p*” photoswitch that undergoes photochemical backreaction exclusively (no thermal backreaction observed, Supplementary Figure 17 & 18).

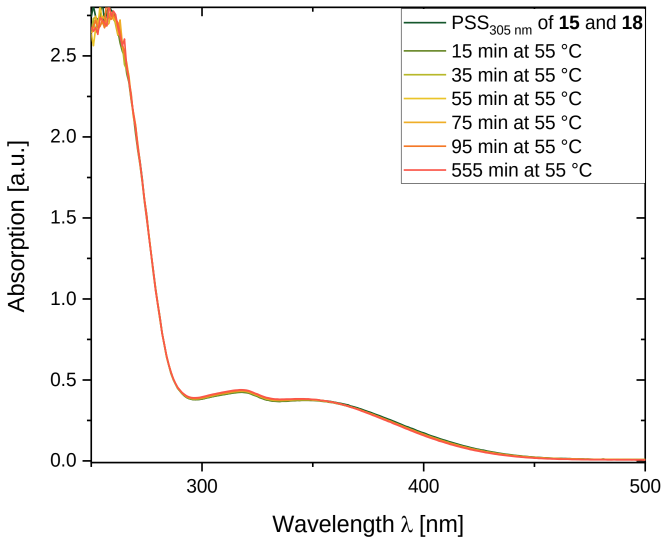

Supplementary Figure 17. UV/vis absorption of **15/18** at the $PSS_{305\ nm}$ in MeCN (c_{start}(**15** = 2.1×10^{-4} mol L^{-1}) upon heating the solution to 55 °C for 555 min.

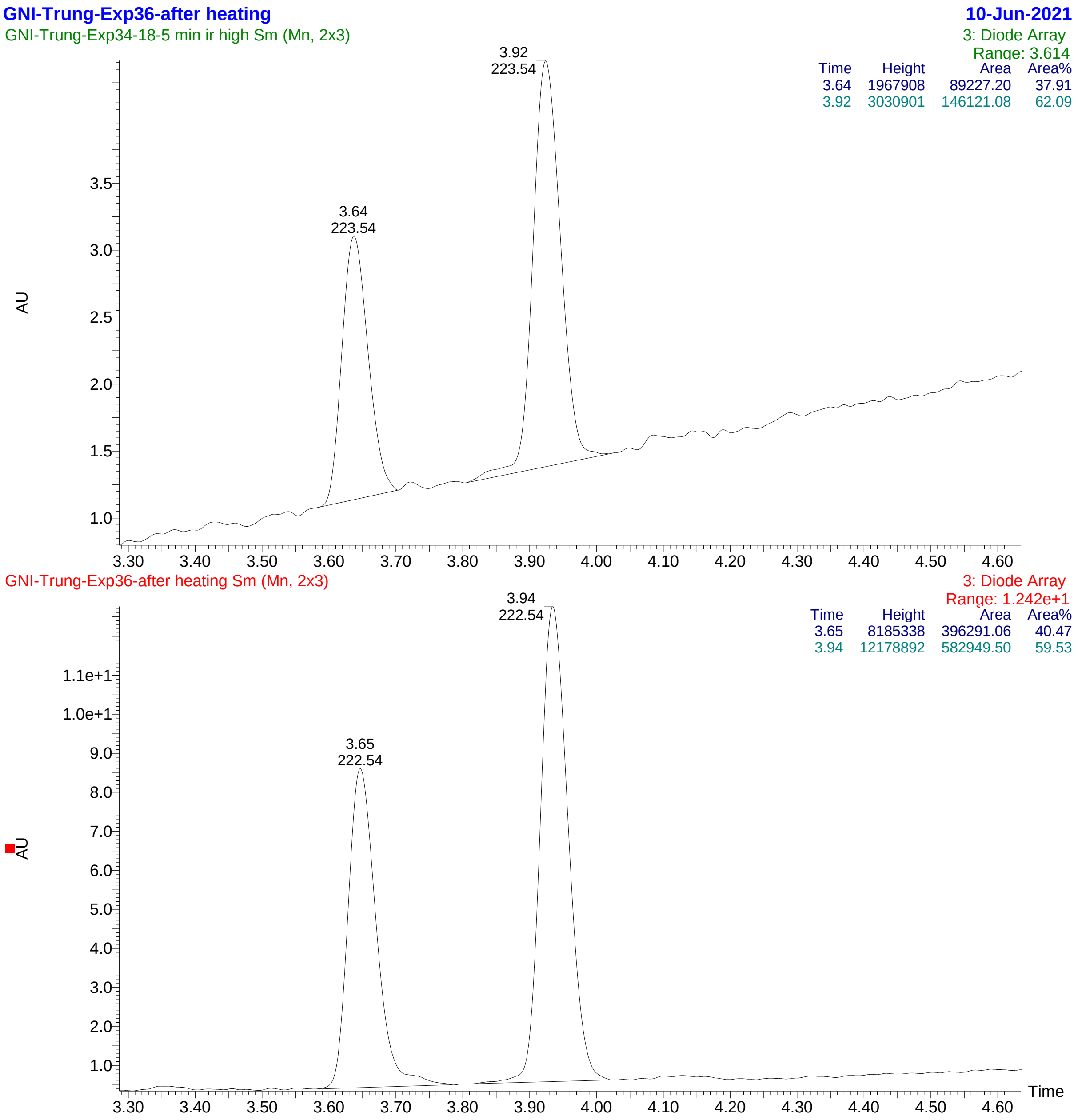

Supplementary Figure 18. UV/vis absorption trace of the HPLC chromatogram obtained from the thermal stability tests (Supplementary Figure 17) conducted with a solution of **15**/**18** in MeCN at the $PSS_{305\ nm}$. Top before heating, bottom after heating for 555 min at 55 °C. The sum of absorption (300–650 nm) at the UV/vis absorption diode detector was used as analytical probe (ordinate) to detect any sort of new forming byproduct.

1.7.4 UV/vis absorption spectroscopy and irradiation of purified (isolated) 18

The irradiation control experiments of HPLC-purified **18** exclude (photo-)side reactions from other species in the PSS, such as **15** (Supplementary Figure 19–20). The formed trace impurity was found to arise with 455 nm irradiation of prior isolated **18** at t_R = 3.02 min, see Supplementary Figure 21, bottom chromatogram).

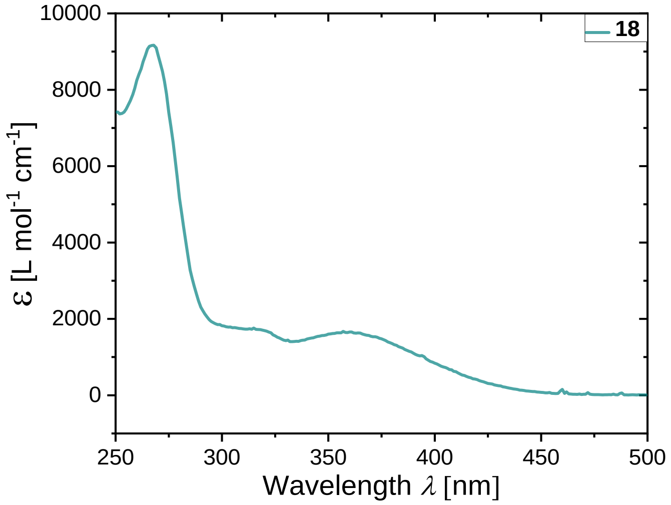

Supplementary Figure 19. UV/vis absorption of isolated **18** (c(**18**) = 8.6×10^{-5} mol L^{-1}) in MeCN.

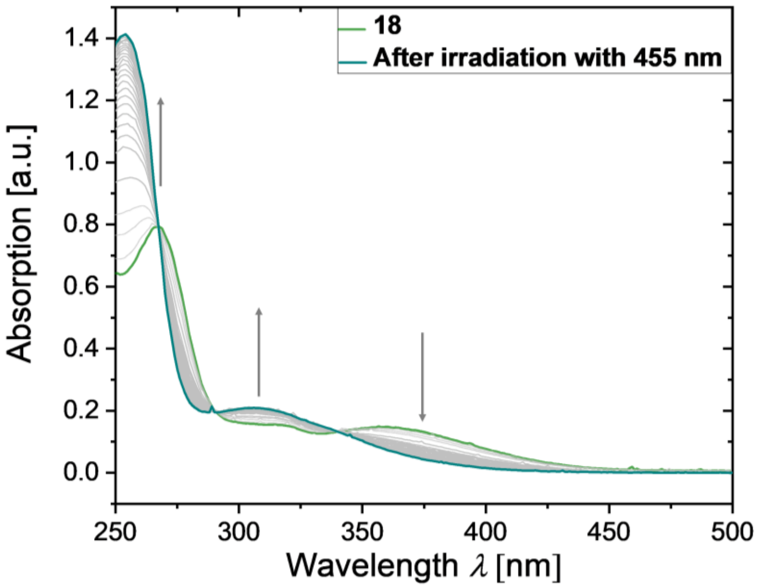

Supplementary Figure 20. UV/vis absorption of **18** in MeCN (c_{start}(**18**) = 8.6×10^{-5} mol L^{-1}) upon irradiation with a 455 nm LED.

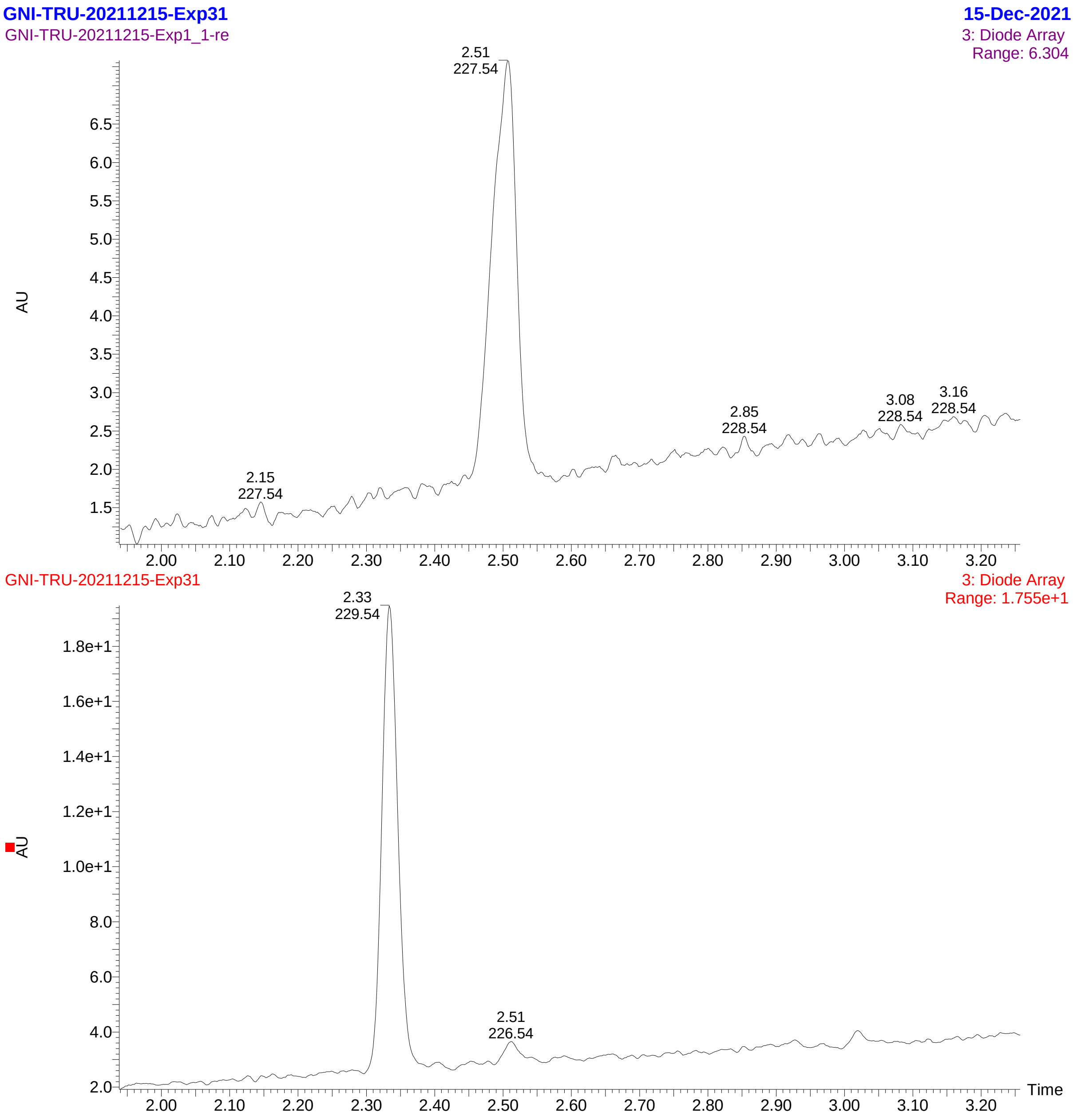

Supplementary Figure 21. UV/vis absorption trace of the HPLC chromatogram obtained from the irradiation of **18** shown in Supplementary Figure 20. Top HPLC chromatogram of isolated **18**, bottom spectroscopy solution after irradtion with a 455 nm LED. The sum of absorption (300–650 nm) at the UV/vis absorption diode detector was used as analytical probe (ordinate) to detect any sort of new forming byproduct.

1.7.5 Long-time irradiation / Fatigue Experiment

We intentionally promoted the fatigue-causing side reaction by constant irradiation of a solution of **15** and **18** (at the PSS) with 340 nm for 30 min (Supplementary Figure 22). Analytical HPLC revealed the formation two trace side products (at 1.30 min and 2.06 min) as well as one minor side product (3.02 min, molecular structure not determined), thus rationally explaining the observed fatigue resistance.

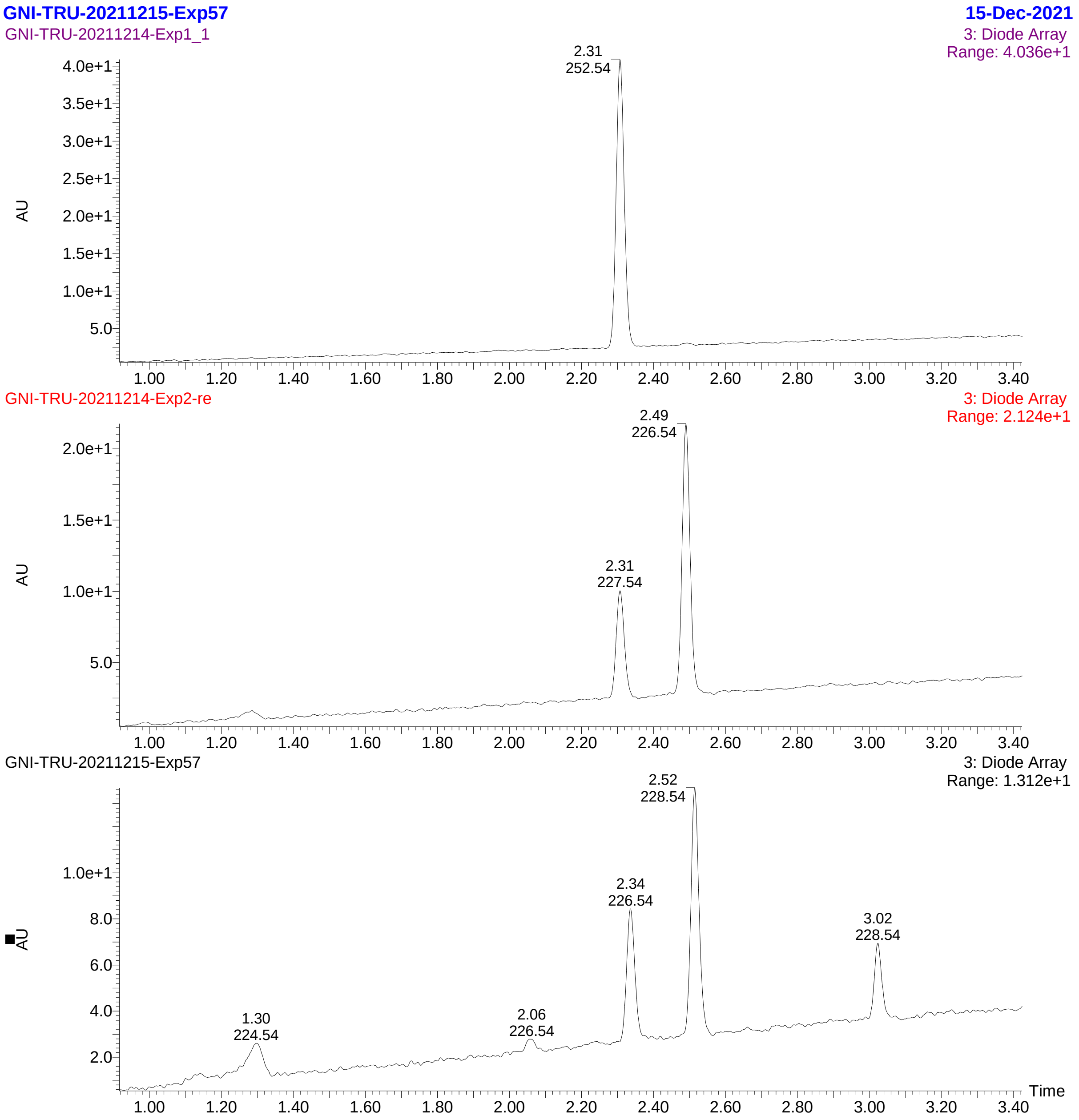

Supplementary Figure 22. UV/vis absorption trace of the HPLC chromatogram obtained from pure **15**, after the irradiation with a 305 LED (PSS_{305} **15**/**18**) (middle) and after irradiating the spectroscopy solution represented in the middle for 30 min with a 340 nm LED to investigate the possible degradation products. The sum of absorption (300–650 nm) at the UV/vis absorption diode detector was used as analytical probe (ordinate) to detect any sort of new forming byproduct.

1.8 Computational Analysis

All calculations were conducted with Gaussian 16 Revision A.03[16] on the chccs-cluster of the Department of Chemistry of Humboldt University of Berlin starting from pre-optimized MM2 force field models. The structures are confirmed ground-state minima according to the analysis of their analytical frequencies computed at the same level, which show no imaginary frequencies.

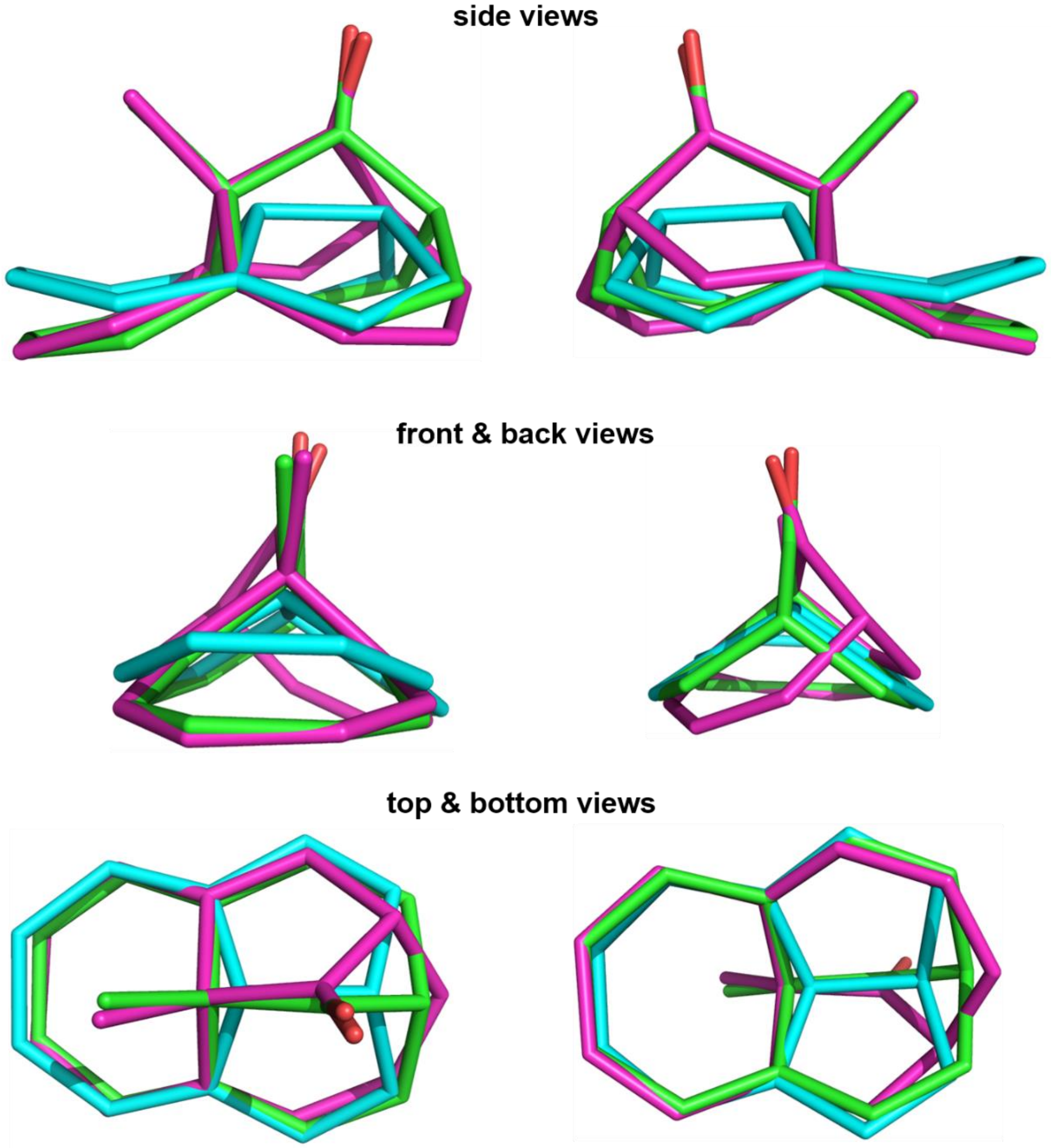

Supplementary Figure 23. Overlap of the geometry-optimized structures of elassovalene **3** (in cyan), Me-**15** (in green), and Me-**18** (in pink) at the B3LYP-D3/def2TZVP level of theory.

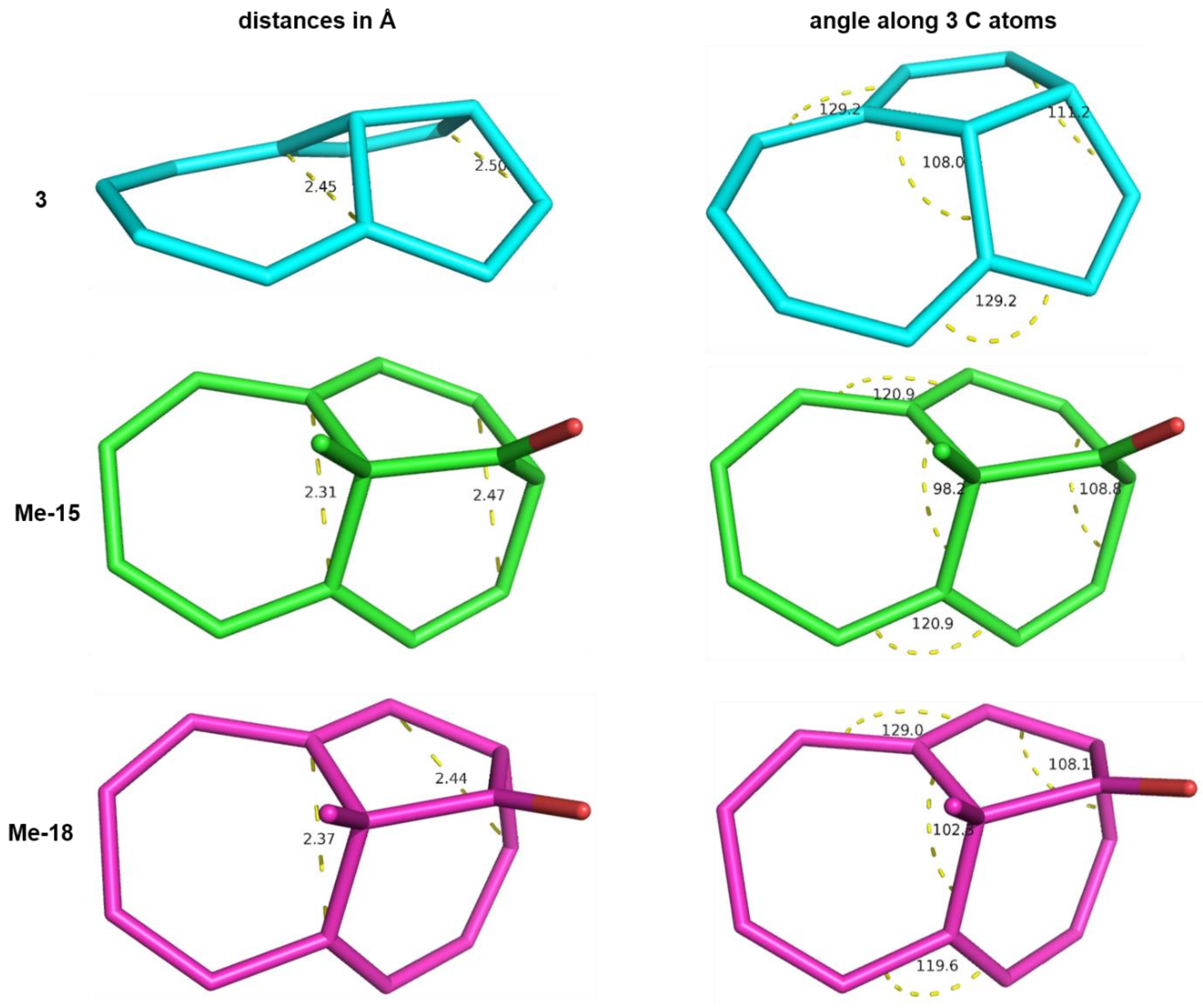

Supplementary Figure 24. Overview of selected C—C distances and C–C–C bond angles of geometry-optimized structures of elassovalene **3** (in cyan), Me-**15** (in green), and Me-**18** (in pink) at the B3LYP-D3/def2TZVP level of theory.

1.8.1 NICS Scans[17–21]

NICS values were calculated using GIAO methods at the B3LYP/6-311G(d,p) level of theory on previously geometry-optimized MM2 force field structures followed by a DFT geometry optimization at the B3LYP-D3/def2TZVP level of theory. Performing NICS scans on a system as **15** and **18** bears several challenges. The ring-system is not planar and in the case of **18** not symmetric. Therefore we decided to calculate the $NICS_{ZZ}$ metric surfaces at various heigths above the ring planes additionally to the widely known NICS-XY scans[20]. According to a detailed analysis by Gershoni-Poranne and Stanger, this is the recommended approach for non-planar molecules, in which it is not feasible to separate the π and σ contributions to the NICS value[21]. A calculated reference of benzene is given at the beginning of this section.

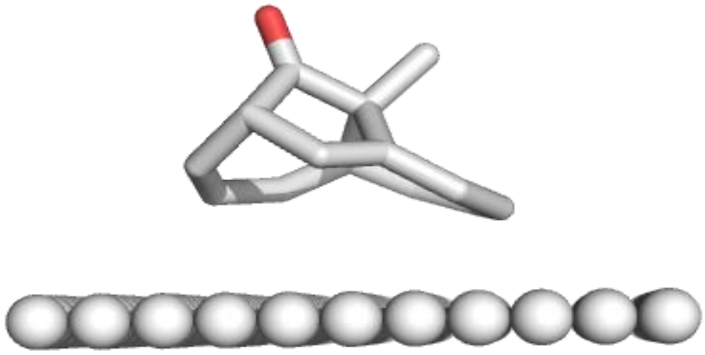

Supplementary Figure 25. Schematic representation of the becquerel surface (10 Å × 10 Å grid with 0.5 Å distance intervals between the probes) placed under the homoaromatic ring-system centroid of **Me-18**. The distance *d* is selected as either 1.8 Å, 2.0 Å, or 2.2 Å to minimize the influence of the σ–bond-electrons on the NICS value as recommended by Gershoni-Poranne and Stanger[21].

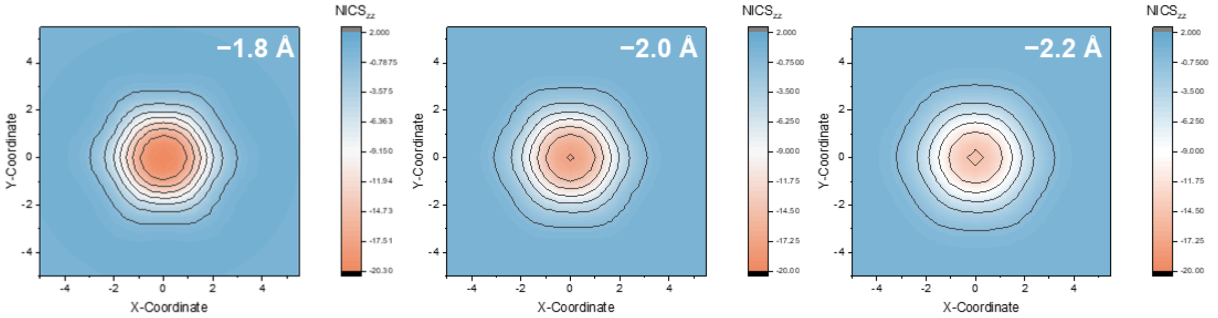

Supplementary Figure 26. $NICS_{ZZ}$-XY heat maps of benzene showing the plane below the molecule at the distances –2.2 Å, –2.0 Å, or –1.8 Å.. Note the slightly different scale of this NICS-2D map at 1.8 Å (from –20.30 to 2.000 ppm) compared to the other NICS-2D plots (from –20.00 to 2.000 ppm).

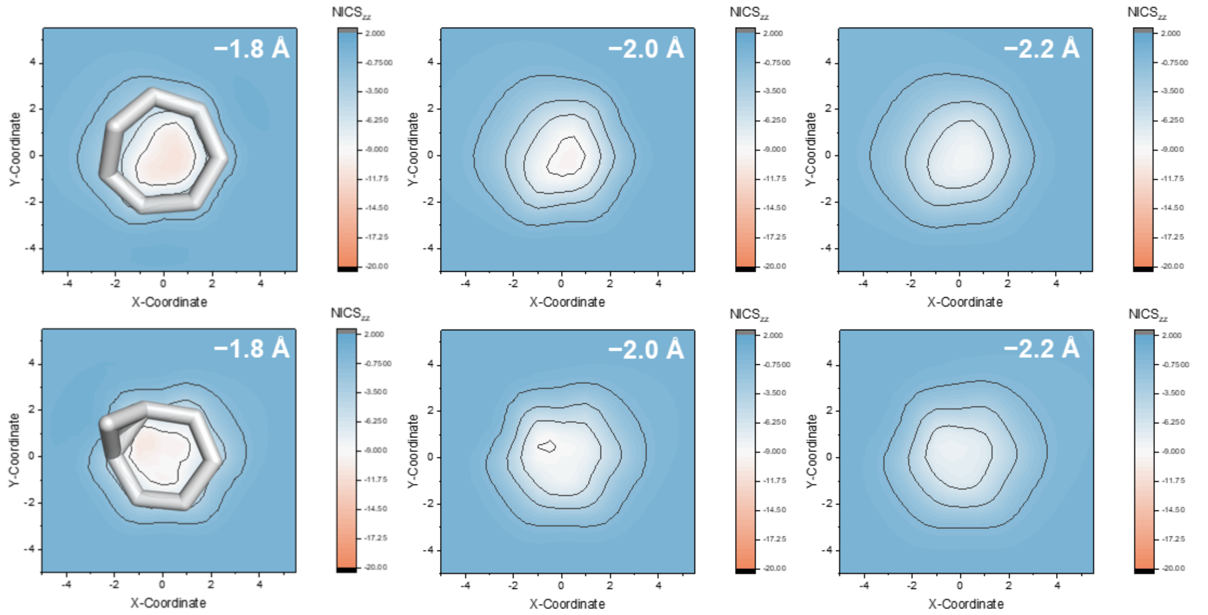

Supplementary Figure 27. NICS$_{ZZ}$-XY heat maps of cycloheptatriene (top row) and norcaradiene (bottom row) at the plane below the molecule with the distances –2.2 Å, –2.0 Å, or –1.8 Å.

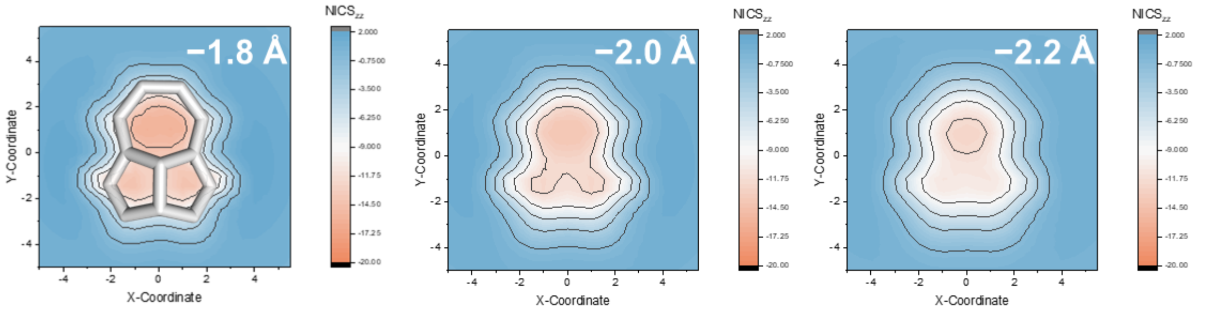

Supplementary Figure 28. NICS$_{ZZ}$-XY heat maps of elassovalene showing the plane below the molecule at the distances –2.2 Å, –2.0 Å, or –1.8 Å.

Supplementary Table 4. Calculated $NICS_{ZZ}$ values of the central becquerel, extracted from the GIAO calculations

Molecule	$NICS_{ZZ}$ (ppm)	Distance (Å)
Cycloheptatriene	–10.7	–1.8
	–9.5	–2.0
	–8.3	–2.2
Norcaradiene	–9.3	–1.8
	–8.5	–2.0
	–7.6	–2.2
Elassovalene (7 membered ring)	–15.6	–1.8
	–14.0	–2.0
	–12.5	–2.2
Elassovalene (below bridge)	–12.5	–1.8
	–11.6	–2.0
	–10.7	–2.2
Benzene	–20.2	–1.8
	–17.4	–2.0
	–14.9	–2.2

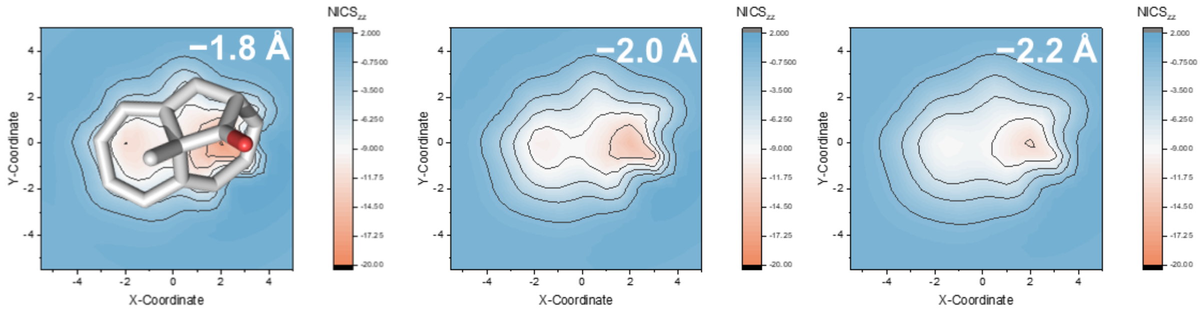

Supplementary Figure 29. NICS$_{zz}$-XY heat maps of compound **Me-18** showing the plane below the molecule at the distances –2.2 Å, –2.0 Å, or –1.8 Å. Without (Top) and with an overlayed DFT-optimized model of **Me-18** (bottom).

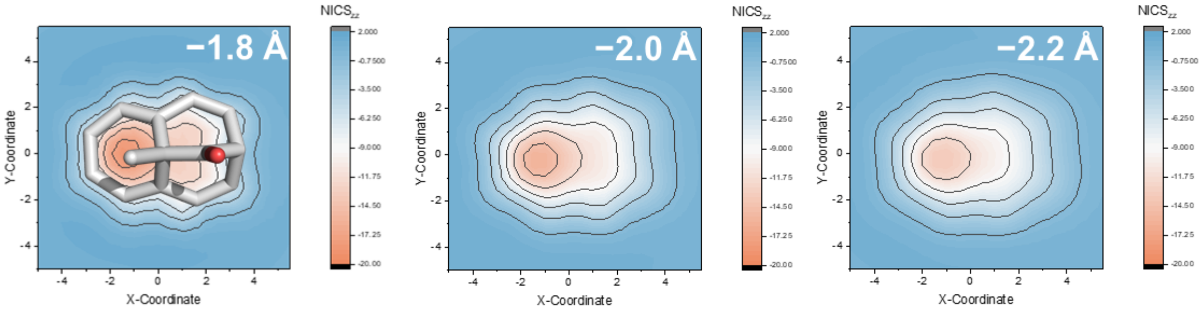

Supplementary Figure 30. NICS$_{zz}$-XY heat maps of compound **Me-15** showing the plane below the molecule at the distances –2.2 Å, –2.0 Å, or –1.8 Å. Without (Top) and with an overlayed computer modelof **Me-15** (bottom).

1.8.2 ACID Plots

ACID plots[22,23] were generated for cycloheptatriene, **15** and **18**, which were previously geometry-optimized using an MM2 force field followed by a DFT geometry optimization at the B3LYP-D3/def2TZVP level of theory. We calculated the ACID plots at the HF/6-311G(d,p) and the B3LYP-D3/def2TZVP level of theory, as was earlier compared by Herges and co-workers in their initial report[22,23]. We prioritize the hybrid functional B3LYP method with Grimme's D3 dispersion for our conclusion although both methods give the same qualitative results. The critical isosurface value (CIV) (the lowest ACID value in space between two interacting units) is defined as a measure of the strength of a conjugation[22,23]. The values for cycloheptatriene are shown for comparison, as its homoaromatic character has been discussed previously[24].

ACID Plots of Cycloheptatriene

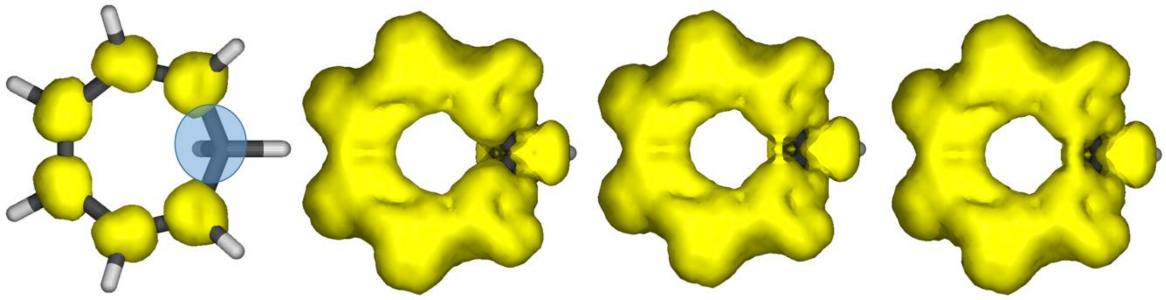

Supplementary Figure 31. ACID plots of cycloheptatriene at iso-surface(is)-values of 0.1, 0.028, 0.027, 0.026 showing that the topology of the ACID boundary surface changes at an is-value of 0.027(CIV). The calculations gave the same CIV using either HF/6-311G(d,p) and B3LYP-D3/def2TZVP.

ACID Plots of Norcaradiene

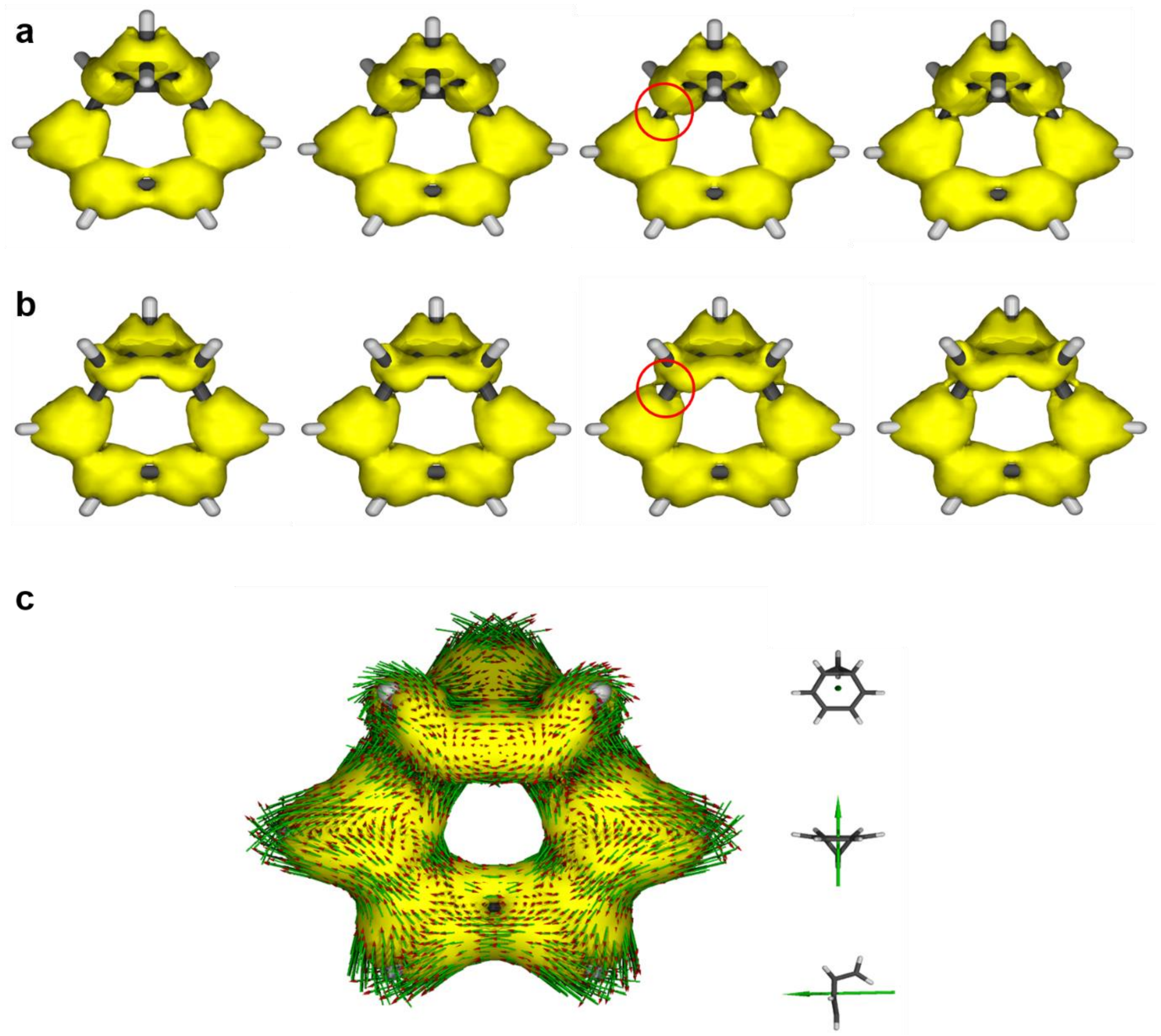

Supplementary Figure 32. (a) top-view on ACID plots of norcaradiene at is-values of 0.059, 0.058, 0.057, 0.056 and 0.055 showing the changes of topology of the ACID boundary surface at an is-value of 0.057 (encircled in red), level of theory: B3LYP-D3/def2TZVP; (b) bottom-view on ACID plots of norcaradiene as in panel a; (c) ACID plot of norcaradiene with the vector field on the 0.04 is-value (the alignment of the external magnetic field as green arrow at the right). The clockwise alignment –accounting for a diatropic ring current– is significantly disturbed.

ACID Plots of Elassovalene

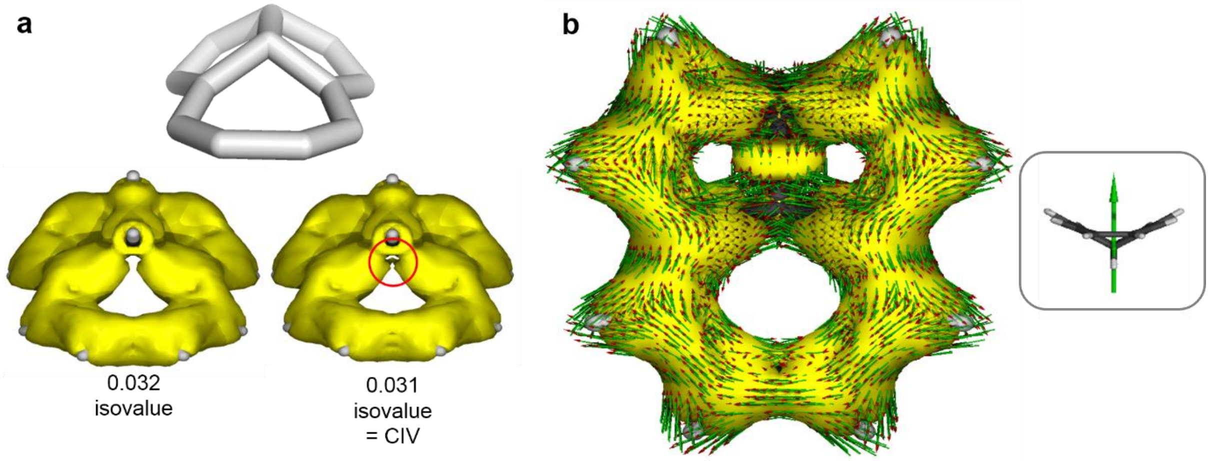

Supplementary Figure 33. (a) ACID plots of elassovalene at is-values of 0.032 and 0.031 showing the changes of topology of the ACID boundary surface at an is-value of 0.031 (encircled in red), but a second change does not even occur at an is-value of 0.01, calculated at the B3LYP-D3/def2TZVP level of theory; (b) ACID plot of elassovalene with the vector field (the alignment of the external magnetic field as green arrow at the right).

ACID Plots of 15

For the ACID plots at the HF/6-311G(d,p) the topology of the ACID boundary surface changes first between the central bridged C-atoms C(4) and C(9) (encircled in red, see above) with a CIV of 0.033. In comparison, using B3LYP-D3/def2TZVP the topology of the ACID boundary surface changes first between the upper bridged C-atoms C(2) and C(11) (encircled in red, see below) with a CIV of 0.034

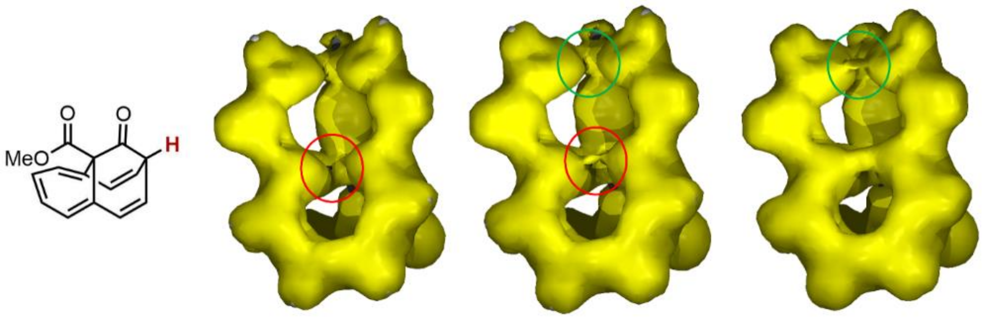

Supplementary Figure 34. ACID plots of **15** at is-values of 0.034, 0.033 and 0.027 showing that the topology of the ACID boundary surface changes at an is-value (= CIV) of 0.033 (encircled in red) and a second occurs at an is-value of 0.027 (encircled in green), calculated at the HF/6-311G(d,p) level of theory.

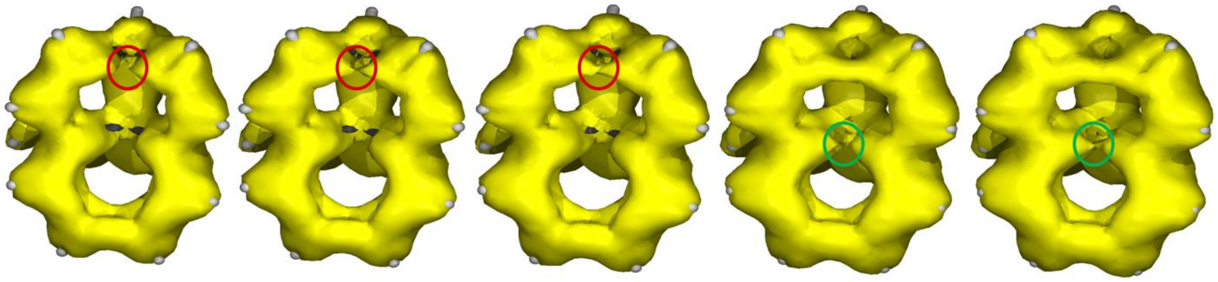

Supplementary Figure 35. ACID plots of **15** at is-values of 0.035, 0.034, 0.033, 0.027 and 0.026 showing that the topology of the ACID boundary surface changes at an is-value of 0.034 (encircled in red) and a second change occurs at an is-value of 0.026 (encircled in green), calculated at the B3LYP-D3/def2TZVP level of theory.

ACID Plots of 18

For the ACID plots at the HF/6-311G(d,p) the topology of the ACID boundary surface changes first between the upper bridged C-atoms C(1) and C(10) (encircled in red, see above) with a CIV of 0.033. In comparison using B3LYP-D3/def2TZVP the topology of the ACID boundary surface changes between the upper bridged C-atoms C(1) and C(10) as well (encircled in red, see below), but with a higher CIV of 0.041.

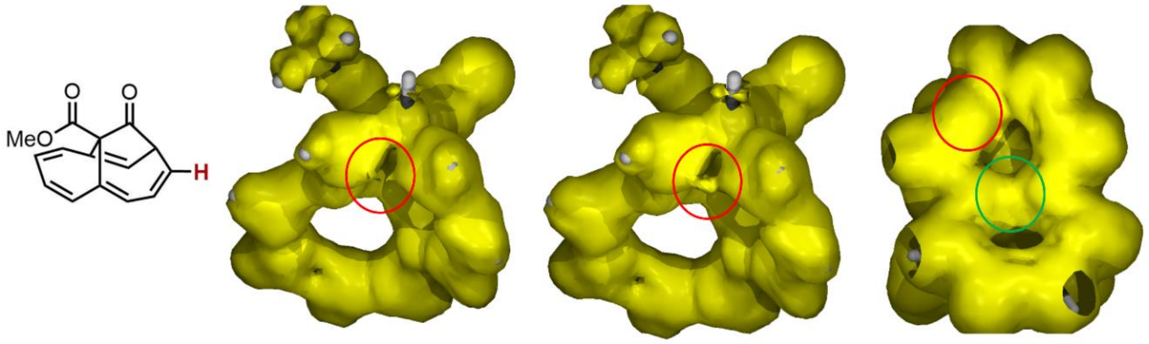

Supplementary Figure 36. ACID plots of **18** at is-values of 0.034, 0.033 and 0.01 showing that the topology of the ACID boundary surface changes at an is-value of 0.033 (encircled in red), but a second change does not even occur at an is-value of 0.01 (circled in green), calculated at the HF/6-311G(d,p) level of theory

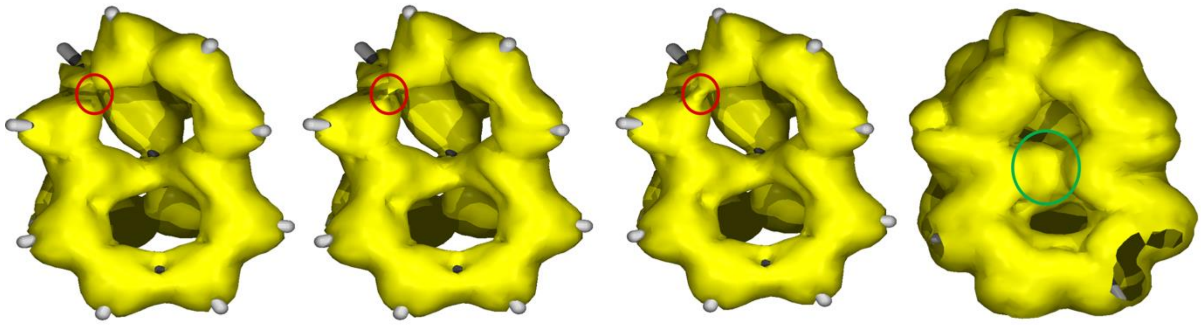

Supplementary Figure 37. ACID plots of **18** at is-values of 0.041, 0.040, 0.039 and 0.01 showing that the topology of the ACID boundary surface changes at an is-value of 0.040 (encircled in red), but a second topology change (at the central C(4) and C(9)) does not occur even at an is-value of 0.01 (the absence is circled in green), calculated at the B3LYP-D3/def2TZVP level of theory.

1.8.3 Calculated ^{1}H NMR chemical shifts of 15, 18, Me-15, Me18, norcaradiene, cycloheptatriene, elassovalene and benzene

^{1}H-NMR chemical shifts were calculated using GIAO methods at the B3LYP/6-311G(d,p) level of theory on optimized geometries obtained at the DFT:B3LYP-D3/def2TZVP level of theory. A reference of TMS was used for calibration purposes. The results are summarized in the following table. In agreement with the experimental values, the hydrogen atoms above the ring currents in **15** and **18** show significant up-field shifts, while in contrast the hydrogen atoms H(20) in ellasovalene shows only a moderate up-field shift.

Supplementary Table 5. Calculated isotropic magnetic shielding vectors of selected compounds at the DFT:B3LYP/6-311G(d,p) (GIAO) level of theory.

**Compound**	**Isotropic magnetic shielding vector (ppm)**	**Isotropic magnetic shielding vector (ppm), relative to TMS**	**Experimental chem. shifts (ppm)**
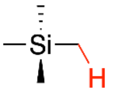 **TMS**	Average: 32.07257	0	0
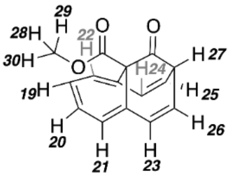 **Compound 15**	H19 24.9336<br>H20 24.7207<br>H21 24.8360<br>H22 25.0872<br>H23 25.1480<br>H24 25.2668<br>H25 27.0042<br>H26 27.0680<br>H27 28.5223<br>H28 28.6249<br>H29 29.0227<br>H30 28.4697	H19 7.13897<br>H20 7.35187<br>H21 7.23657<br>H22 6.98537<br>H23 6.92457<br>H24 6.80577<br>H25 5.06837<br>H26 5.00457<br>H27 3.55027<br>H28 3.44767<br>H29 3.04987<br>H30 3.60287	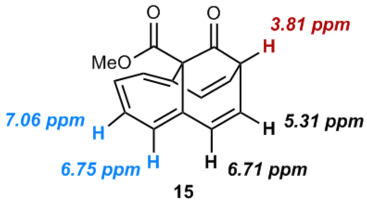
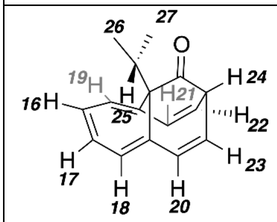 **Compound-Me-15**	H16 24.7137<br>H17 24.7142<br>H18 24.9980<br>H19 24.9987<br>H20 25.2481<br>H21 25.2463	H16 7.35887<br>H17 7.35837<br>H18 7.07457<br>H19 7.07387<br>H20 6.82447<br>H21 6.82627	N/A

	H22 27.1827<br>H23 27.1823<br>H24 28.3686<br>H25 32.7787<br>H26 32.5125<br>H27 32.5076	H22 4.88987<br>H23 4.89027<br>H24 3.70397<br>H25 –0.70613<br>H26 –0.43993<br>H27 –0.43503	
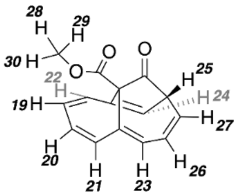<br>**Compound 18**	H19 25.5282<br>H20 25.8499<br>H21 24.3894<br>H22 24.9935<br>H23 25.7629<br>H24 28.6321<br>H25 28.4925<br>H26 25.6646<br>H27 26.6699<br>H28 28.4591<br>H29 28.8230<br>H30 28.4618	H19 6.54437<br>H20 6.22267<br>H21 7.68317<br>H22 7.07907<br>H23 6.30967<br>H24 3.44047<br>H25 3.58007<br>H26 6.40797<br>H27 5.40267<br>H28 3.61347<br>H29 3.24957<br>H30 3.61077	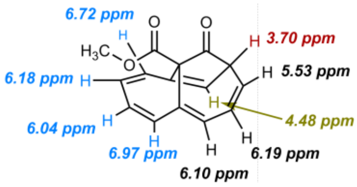
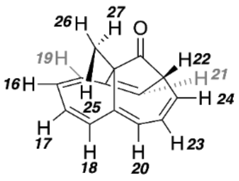<br>**Compound-Me-18**	H16 25.6014<br>H17 25.5930<br>H18 24.7790<br>H19 25.0241<br>H20 25.7816<br>H21 28.4733<br>H22 28.5443<br>H23 25.7005<br>H24 26.6724<br>H25 32.2398<br>H26 31.3448<br>H27 31.3750	H16 6.47117<br>H17 6.47957<br>H18 7.29357<br>H19 7.04847<br>H20 6.29097<br>H21 3.59927<br>H22 3.52827<br>H23 6.37207<br>H24 5.40017<br>H25 –0.16723<br>H26 0.72777<br>H27 0.69757	N/A
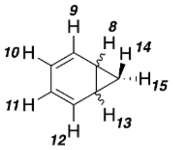<br>**Norcaradiene**	H8 30.1817<br>H9 25.6852<br>H10 26.0246<br>H11 26.0253<br>H12 25.6847<br>H13 30.1810<br>H14 33.0605<br>H15 30.5118	H8 1.89087<br>H9 6.38737<br>H10 6.04797<br>H11 6.04727<br>H12 6.38787<br>H13 1.89157<br>H14 –0.98793<br>H15 1.56077	N/A
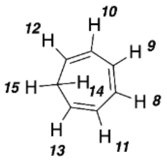<br>**Cycloheptatriene**	H8 25.1746<br>H9 25.1742<br>H10 25.5414<br>H11 25.5412<br>H12 26.7523<br>H13 26.7518<br>H14 30.4726<br>H15 29.1132	H8 6.89797<br>H9 6.89837<br>H10 6.53117<br>H11 6.53137<br>H12 5.32027<br>H13 5.32077<br>H14 1.59997<br>H15 2.95937	H8/9: 6.58<br>H10/11:6.18<br>H12/13:5.36<br>H14/15:2.24<br><br>Source: SDBS database<br>https://sdbs.db.aist.go.jp/<br>(SDBS No. 3886HSP-49-492)

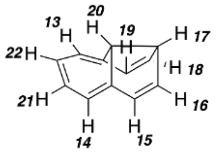<br>**Ellassovalene**	H13 25.1788<br>H14 25.1837<br>H15 25.3234<br>H16 25.9241<br>H17 28.3329<br>H18 25.9225<br>H19 25.3253<br>H20 30.8431<br>H21 24.9640<br>H22 24.9641	H13 6.89377<br>H14 6.88887<br>H15 6.74917<br>H16 6.14847<br>H17 3.73967<br>H18 6.15007<br>H19 6.74727<br>H20 1.22947<br>H21 7.10857<br>H22 7.10847	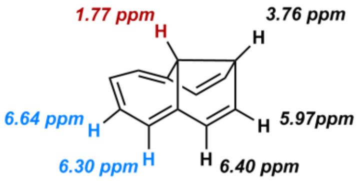<br>3
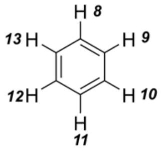<br>**Benzene**	H8 24.5547<br>H9 24.5518<br>H10 24.5519<br>H11 24.5549<br>H12 24.5520<br>H13 24.5519	H8 7.51787<br>H9 7.52077<br>H10 7.52067<br>H11 7.51767<br>H12 7.52057<br>H13 7.52067	7.36

Comparison of chemical shifts of selected hydrogen atoms shows that the level of theory is appropriate for evaluation of the magnetic shielding analysis, such as our NICS calculations. The deviation is in the range of $\Delta\delta_{calc-obs}$ ca. –0.3 ppm, depending on solvent effects.

1.8.4 TD-DFT Results, Orbital Energy Levels of 15 and 18 and Orbital Plots

Vertical transitions were calculated for **15** and **18** on the geometry obtained from B3LYP-D3/def2-TZVP optimizations for the first 50 transitions at the TD-DFT-D3(BJ)/UCAM-B3LYP/def2-TZVP level of theory using MeCN as SMD solvent model. Die original dataset of the first 10 transitions can be found in the appendix, section S2.3. The obtained transitions were simulated to obtain a spectral trace using the SpecDis software, developed by Bringmann and co-workers.[25]

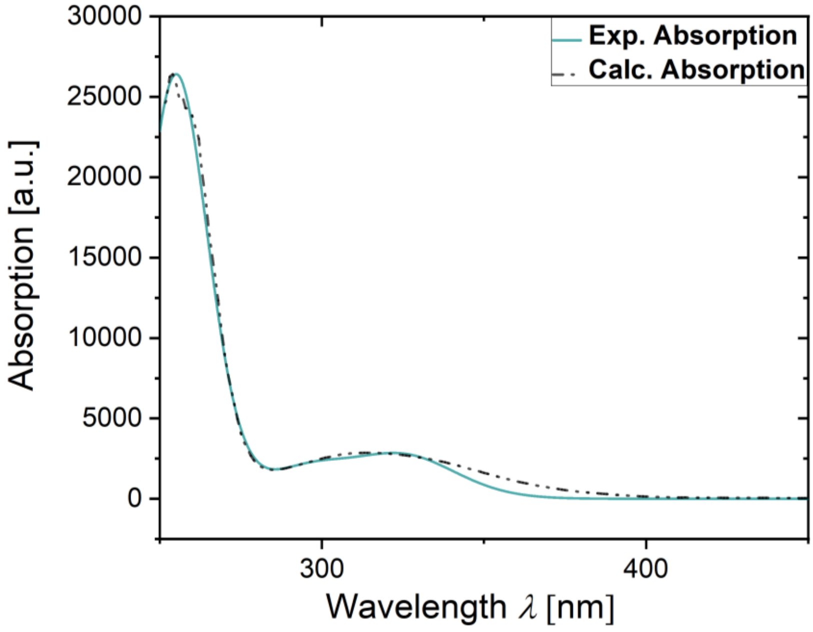

Supplementary Figure 38. Comparison of the UV/Vis spectra of **15** in MeCN at 295 K (cyan solid graph, $c = 1.1 \cdot 10^{-4}$ M) and the TD-DFT-calculated spectra (black dotted graph, level of theory: TD-DFT-D3(BJ)/UCAM-B3LYP/def2-tzvp/SMD(MeCN). The calculated spectrum is shown with a UV correction of −13 nm, a scaling factor of 0.2648, and a standard deviation (graph width fitting parameter σ) of σ = 0.24 eV. The comparison was performed using the SpecDis software, which computed a similarity factor of 99.8%.[25]

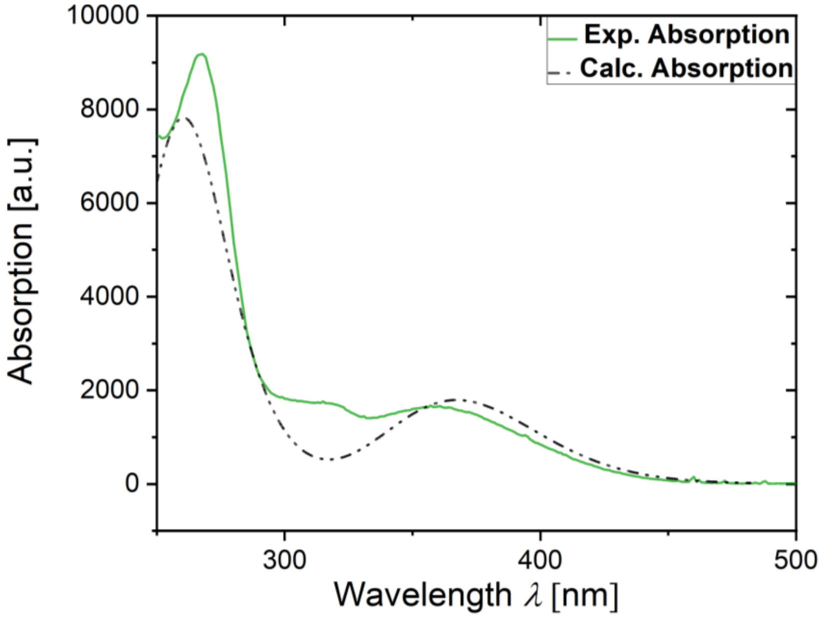

Supplementary Figure 39. Comparison of the UV/Vis spectra of **18** in MeCN at 295 K (gren solid graph, $c = 8.6 \cdot 10^{-5}$ M) and the TD-DFT-calculated spectra (black dotted graph, level of theory: TD-DFT-D3(BJ)/UCAM-B3LYP/def2-tzvp/SMD(MeCN). The calculated spectrum is shown with a UV correction of −18 nm, a scaling factor of 0.2111, and a standard deviation (graph width fitting parameter σ) of σ = 0.29 eV. The comparison was performed using the SpecDis software, which computed a similarity factor of 96.0%.[25]

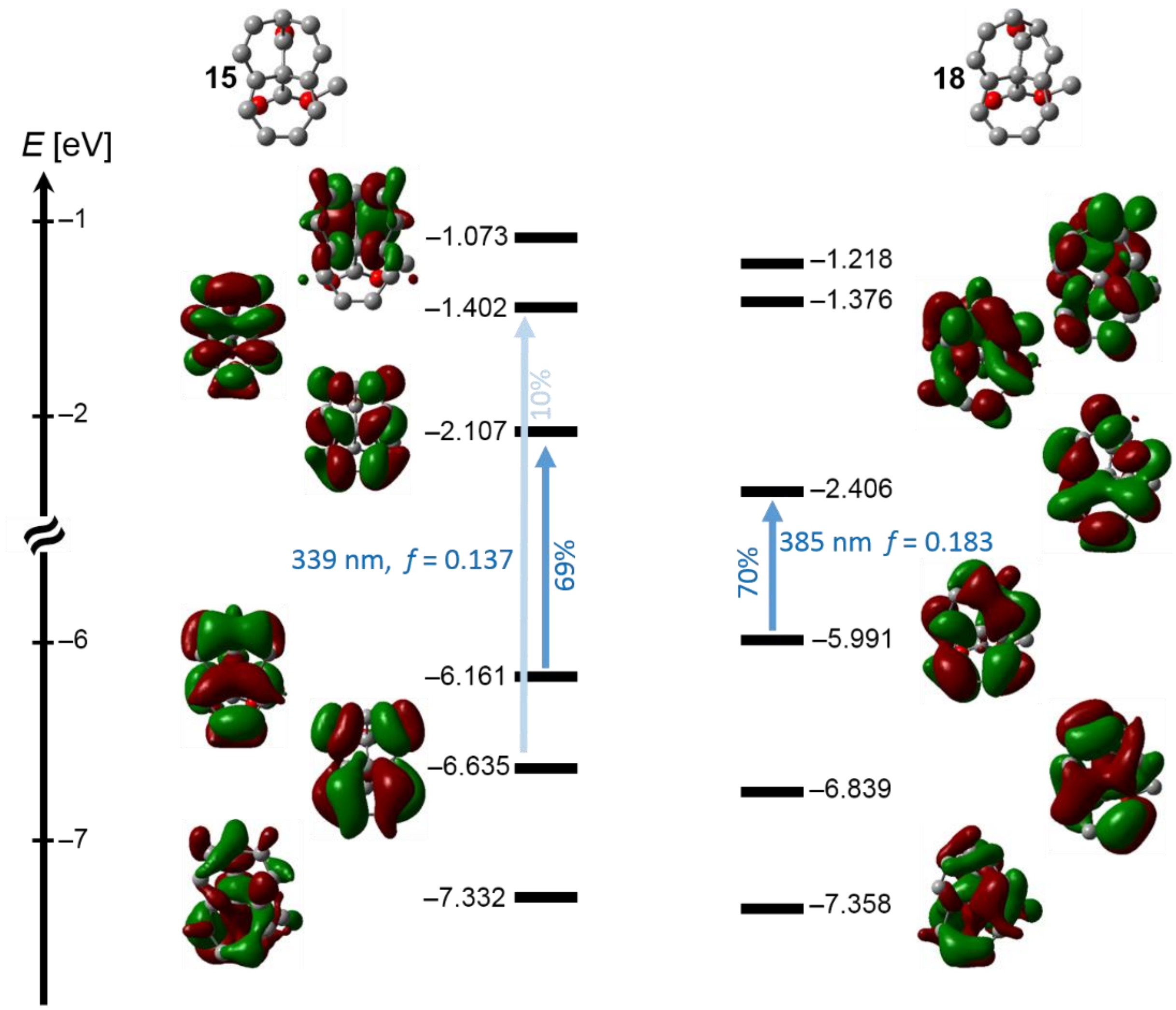

Supplementary Figure 40. Orbital energy diagram of **15** and **18** at the B3LYP-D3/def2-TZVP level of energy (black values) and the lowest vertical transition calculated at the TD-DFT-D3(BJ)/UCAM-B3LYP/def2-TZVP/SMD(MeCN) level of theory (blue transitions). In **15**, this transition is composed of a 69% HOMO→LUMO and a 10% HOMO–1→LUMO+1 combination (energy in nm, *f* is the calculated oscillator strengths). In **18**, this transition is purely composed of a HOMO→LUMO transition. The orbital depictions are plotted at the 0.02 *e* Å^{-3} isosurface at the B3LYP/def2-TZVP level of theory.

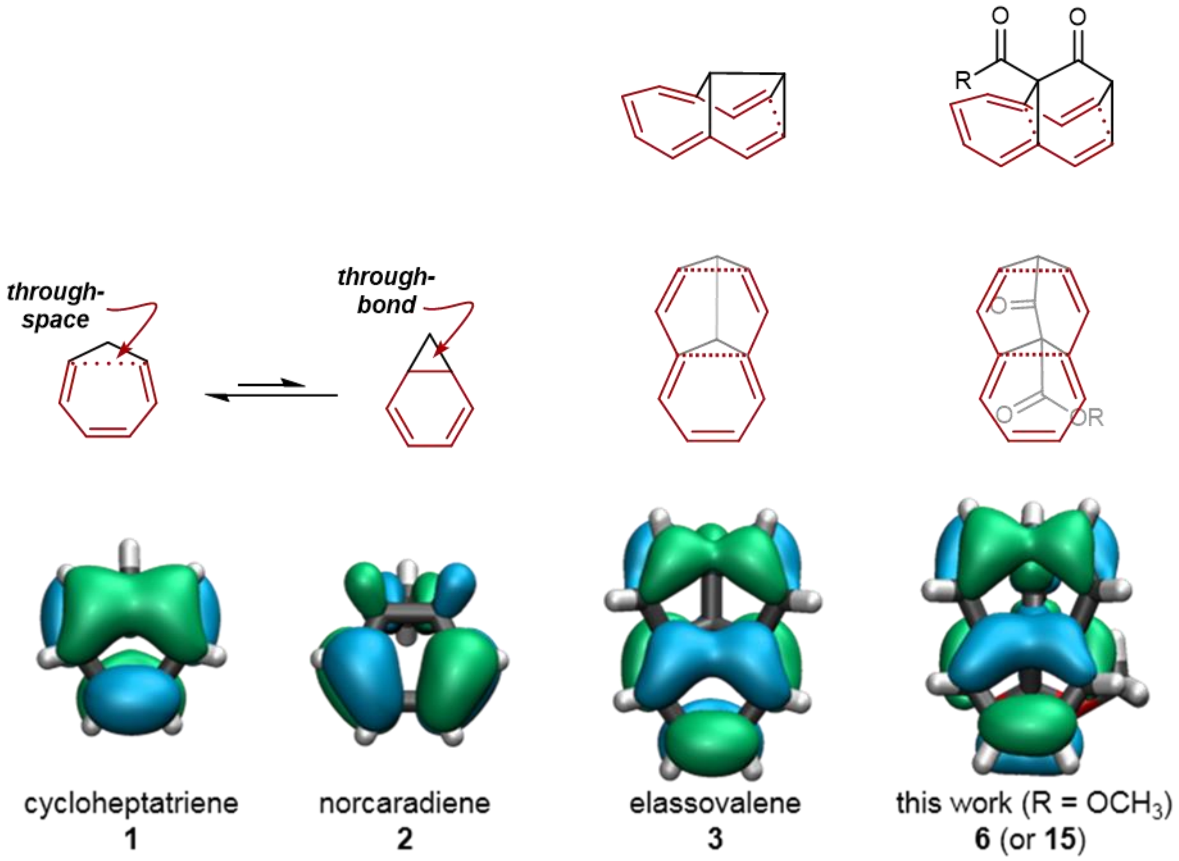

Supplementary Figure 41. HOMO orbital plots for **1**–**3** and **6** at the 0.03 *e* Å^{-3} isosurface at the B3LYP/def2-TZVP level of theory. Note that compound **6** of the introduction was later introduced as **15**.

1.8.5 Strain Analysis on H-15, H-18, Elassovalene

The strain energy of an organic compound can usually be derived from its heat of formation of the elements. The difference in the heat of formation of the strained isomer vs the unstrained isomer can then be regarded as the strain energy. As this method is not feasible for the investigated molecules due to limited synthetic acces to strain free version of **15**, **18**, elassovalene. We therefor decided to choose a recently developed programm to investigate the strain energies theoretically. As the absolute values cannot be compared to experimental results the consistent method used allows internal comparison.[26] StrainViz[27] is based on the analysis of DFT calculations performed with Gaussian. While it has not been benchmarked for tricyclic molecular scaffolds and especially not for homoaromatic compounds, where disruption of the diatropic ring currents is necessary throughout the analysis, the obtained results visualize and support our findings. We adopt the nomenclature for strain energy terms as in the cited work of Jasti and co.workers.[27] This strain energy differs from the strain energy as defined by A. von Baeyer.[28] All calculations were performed on the geometry optimized structures of elassovalene, and on the H-subsituted parent scaffolds of **15** (**H-15**) and **18** (**H-18**) that were obtained at the DFT:B3LYP/def2TZVP level of theory. The absolute energies obtained depend on the selected fragments used in the calculations and are therefore given as well in the Appendix. All StrainViz calculations were performed at B3LYP/6-311G(d).

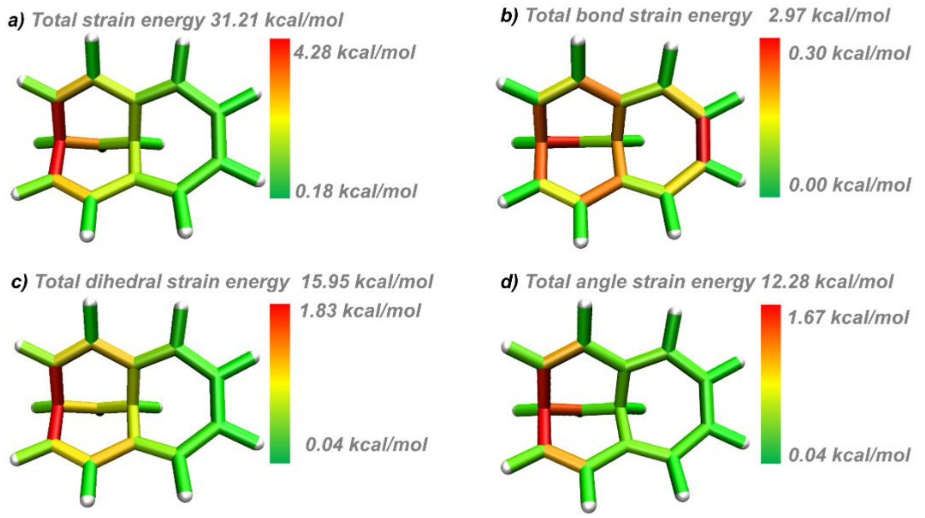

Supplementary Figure 42. The calculated total strain energy, bond strain energy, dihedral strain energy, and angle strain energy of **H-15**.

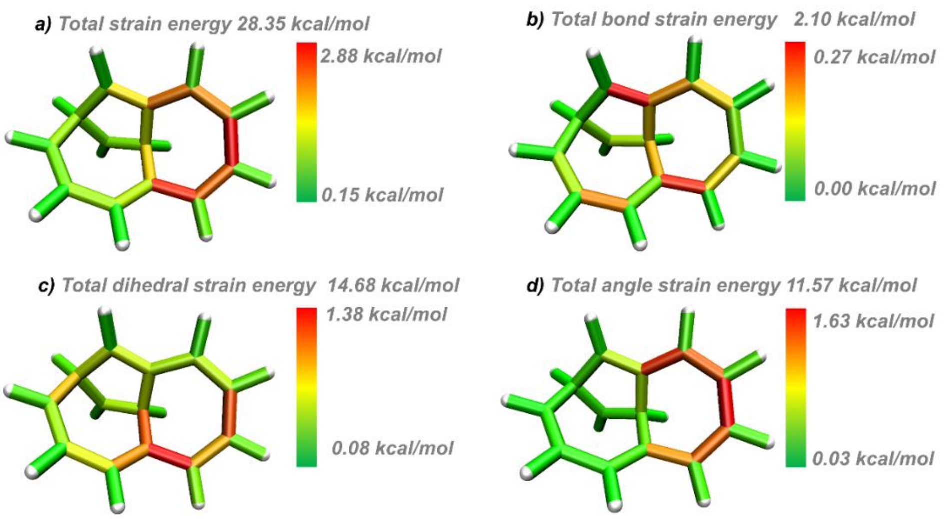

Supplementary Figure 43. The calculated total strain energy, bond strain energy, dihedral strain energy, and angle strain energy of **H-18**.

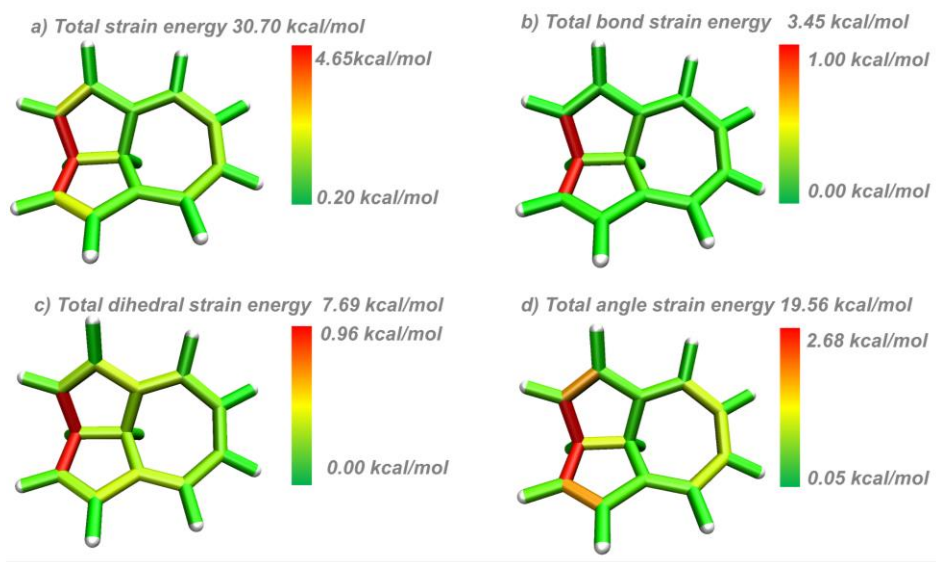

Supplementary Figure 44. The calculated total strain energy, bond strain energy, dihedral strain energy, and angle strain energy of elassovalene (**3**).

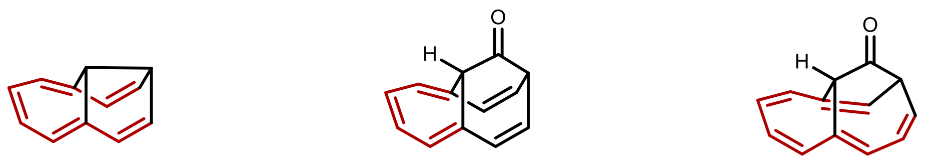

3
elassovalene

H-15

H-18

The total strain energies are in a similar range from 28.35 kcal mol^{-1} to 31.21 kcal mol^{-1} for ellasovalene, **H-15** and **H-18**. It is distributed among several bonds in the molecular scaffold of **H-15**, **H-18**. In contrast the strain energy in elassovalene is concentrated at the two (red) bonds bridging the two cyclopentenylen fragments. This difference in strain distribution might cause different reactivity and stability within the studied series of molecules.

2 Appendix

2.1 Crystallographic Data[29]

2.1.1 (1*s*,1a*R*,7a*S*)-1a,2,7,7a-tetrahydro-1*H*-cyclopropa[b]naphthalene-1-carboxylic acid (*cis*-11)

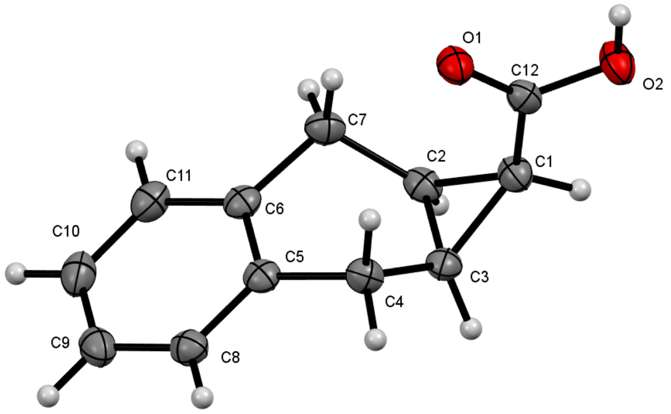

Supplementary Table 6. Crystal data and structure refinement for *cis*-**11**.

CCDC number	2128305	
Empirical formula	$C_{12}\ H_{12}\ O_2$	
Formula weight	188.22	
Temperature	150.00(10) K	
Wavelength	1.54184 Å	
Crystal system	Triclinic	
Space group	*P*-1	
Unit cell dimensions	*a* = 7.5990(5) Å	α = 65.320(6)°
	b = 8.0638(5) Å	β = 79.717(5)°
	c = 8.5252(5) Å	γ = 84.980(6)°
Volume	467.01(5) $Å^3$	
Z	2	
Density (calculated)	1.338 Mg/m^3	
Absorption coefficient	0.726 mm^{-1}	
F(000)	200	
Crystal size	0.37 x 0.22 x 0.17 mm^3	
Theta range for data collection	5.78 to 67.45°.	
Index ranges	$-8 \le h \le 9$, $-9 \le k \le 9$, $-6 \le l \le 10$	
Reflections collected	2725	
Independent reflections	1677 [*R*(int) = 0.0118]	
Completeness to θ = 67.45°	99.4%	
Absorption correction	Semi-empirical from equivalents	

Max. and min. transmission	0.8853 and 0.7755
Refinement method	Full-matrix least-squares on F^2
Data / restraints / parameters	1677 / 0 / 131
Goodness-of-fit on F^2	1.028
Final *R* indices [I>2σ(I)]	*R*1 = 0.0346, w*R*2 = 0.0888
R indices (all data)	*R*1 = 0.0366, w*R*2 = 0.0903
Largest diff. peak and hole	0.195 and –0.195 e^- Å^{-3}

Supplementary Table 7. Bond lengths [Å] and angles [°] for *cis*-**11**.

O(1)-C(12)	1.2265(15)
O(2)-C(12)	1.3250(14)
C(1)-C(12)	1.4667(16)
C(1)-C(3)	1.5337(15)
C(1)-C(2)	1.5347(16)
C(2)-C(3)	1.4917(16)
C(2)-C(7)	1.5213(17)
C(3)-C(4)	1.5200(16)
C(4)-C(5)	1.5149(16)
C(5)-C(8)	1.3888(17)
C(5)-C(6)	1.3997(16)
C(6)-C(11)	1.3904(17)
C(6)-C(7)	1.5123(16)
C(8)-C(9)	1.3906(18)
C(9)-C(10)	1.3832(19)
C(10)-C(11)	1.3887(18)
C(12)-C(1)-C(3)	123.27(10)
C(12)-C(1)-C(2)	123.40(10)
C(3)-C(1)-C(2)	58.18(7)
C(3)-C(2)-C(7)	115.47(10)
C(3)-C(2)-C(1)	60.88(7)
C(7)-C(2)-C(1)	124.44(10)
C(2)-C(3)-C(4)	115.22(10)
C(2)-C(3)-C(1)	60.95(7)
C(4)-C(3)-C(1)	124.12(10)
C(5)-C(4)-C(3)	109.07(9)
C(8)-C(5)-C(6)	119.67(11)
C(8)-C(5)-C(4)	122.97(10)

C(6)-C(5)-C(4)	117.32(10)
C(11)-C(6)-C(5)	119.40(11)
C(11)-C(6)-C(7)	123.05(11)
C(5)-C(6)-C(7)	117.51(10)
C(6)-C(7)-C(2)	108.94(9)
C(5)-C(8)-C(9)	120.55(11)
C(10)-C(9)-C(8)	119.76(11)
C(9)-C(10)-C(11)	120.08(11)
C(10)-C(11)-C(6)	120.54(11)
O(1)-C(12)-O(2)	122.14(11)
O(1)-C(12)-C(1)	125.45(11)
O(2)-C(12)-C(1)	112.41(10)

Symmetry *trans*formations used to generate equivalent atoms:

2.1.2 *tert*-Butyl 3-oxo-3-((1s,1a*R*,7a*S*)-1a,2,7,7a-tetrahydro-1*H*-cyclopropa[b]naphthalen-1-yl)propanoate (12b)

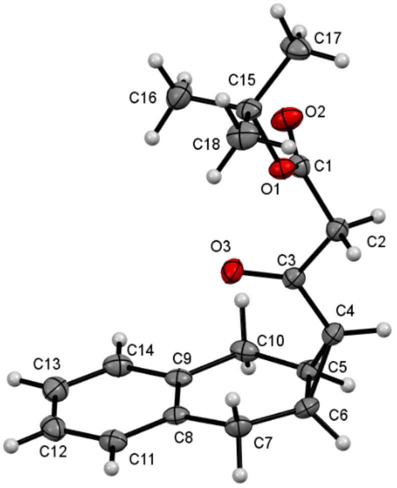

Supplementary Table 8. Crystal data and structure refinement for **12b**.

CCDC number	2128302	
Empirical formula	C18 H22 O3	
Formula weight	286.36	
Temperature	150.01(10) K	
Wavelength	1.54184 Å	
Crystal system	Monoclinic	
Space group	$P2_1/c$	
Unit cell dimensions	a = 5.73381(13) Å	α = 90°
	b = 10.6147(2) Å	β = 91.0213(17)°
	c = 24.6993(5) Å	γ = 90°
Volume	1503.02(5) Å^3	
Z	4	
Density (calculated)	1.265 Mg/m^3	
Absorption coefficient	0.677 mm^{-1}	
F(000)	616	
Crystal size	0.38 x 0.21 x 0.20 mm^3	
Theta range for data collection	3.58 to 67.47°.	
Index ranges	$-6 \le h \le 6$, $-12 \le k \le 12$, $-23 \le l \le 29$	
Reflections collected	5408	
Independent reflections	2705 [*R*(int) = 0.0144]	
Completeness to θ = 67.47°	99.9 %	
Absorption correction	Semi-empirical from equivalents	
Max. and min. transmission	0.8787 and 0.7810	
Refinement method	Full-matrix least-squares on F^2	
Data / restraints / parameters	2705 / 0 / 193	

Goodness-of-fit on F^2	1.057
Final *R* indices [I>2σ(I)]	*R*1 = 0.0343, w*R*2 = 0.0866
R indices (all data)	*R*1 = 0.0378, w*R*2 = 0.0900
Largest diff. peak and hole	0.174 and –0.263 e^- $Å^{-3}$

Supplementary Table 9. Bond lengths [Å] and angles [°] for **12b**.

O(1)-C(1)	1.3349(14)
O(1)-C(15)	1.4878(13)
O(2)-C(1)	1.2060(14)
O(3)-C(3)	1.2131(14)
C(1)-C(2)	1.5100(16)
C(2)-C(3)	1.5181(16)
C(3)-C(4)	1.4748(16)
C(4)-C(5)	1.5296(17)
C(4)-C(6)	1.5307(16)
C(5)-C(6)	1.4910(17)
C(5)-C(10)	1.5084(18)
C(6)-C(7)	1.5122(17)
C(7)-C(8)	1.5098(17)
C(8)-C(9)	1.3959(16)
C(8)-C(11)	1.3980(17)
C(9)-C(14)	1.3957(17)
C(9)-C(10)	1.5123(17)
C(11)-C(12)	1.3813(18)
C(12)-C(13)	1.3895(19)
C(13)-C(14)	1.3841(19)
C(15)-C(16)	1.5138(18)
C(15)-C(18)	1.5185(17)
C(15)-C(17)	1.5191(17)
C(1)-O(1)-C(15)	121.51(9)
O(2)-C(1)-O(1)	125.79(11)
O(2)-C(1)-C(2)	123.17(11)
O(1)-C(1)-C(2)	111.03(9)
C(1)-C(2)-C(3)	112.77(10)
O(3)-C(3)-C(4)	124.65(11)
O(3)-C(3)-C(2)	121.68(10)
C(4)-C(3)-C(2)	113.66(10)

C(3)-C(4)-C(5)	122.80(10)
C(3)-C(4)-C(6)	120.80(10)
C(5)-C(4)-C(6)	58.32(8)
C(6)-C(5)-C(10)	120.38(10)
C(6)-C(5)-C(4)	60.88(8)
C(10)-C(5)-C(4)	122.40(10)
C(5)-C(6)-C(7)	120.80(11)
C(5)-C(6)-C(4)	60.81(8)
C(7)-C(6)-C(4)	122.22(10)
C(8)-C(7)-C(6)	115.94(10)
C(9)-C(8)-C(11)	118.64(11)
C(9)-C(8)-C(7)	122.60(11)
C(11)-C(8)-C(7)	118.72(10)
C(14)-C(9)-C(8)	119.12(11)
C(14)-C(9)-C(10)	118.21(10)
C(8)-C(9)-C(10)	122.67(11)
C(5)-C(10)-C(9)	116.46(10)
C(12)-C(11)-C(8)	121.79(11)
C(11)-C(12)-C(13)	119.46(12)
C(14)-C(13)-C(12)	119.27(12)
C(13)-C(14)-C(9)	121.63(11)
O(1)-C(15)-C(16)	110.49(10)
O(1)-C(15)-C(18)	101.95(9)
C(16)-C(15)-C(18)	111.15(11)
O(1)-C(15)-C(17)	109.22(9)
C(16)-C(15)-C(17)	112.62(11)
C(18)-C(15)-C(17)	110.89(11)

Symmetry *trans*formations used to generate equivalent atoms:

2.1.3 *tert*-Butyl 2-diazo-3-oxo-3-((1*s*,1a*R*,7a*S*)-1a,2,7,7a-tetrahydro-1*H*-cyclopropa[b]naphthalen-1-yl)propanoate (13b)

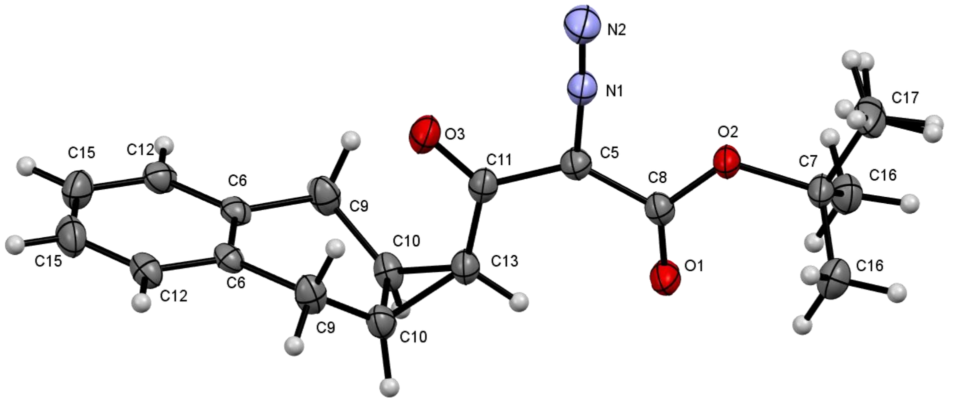

Supplementary Table 10. Crystal data and structure refinement for **13b**.

CCDC number	2128309
Empirical formula	C18 H20 N2 O3
Formula weight	312.36
Temperature	150.01(10) K
Wavelength	1.54184 Å
Crystal system	Orthorhombic
Space group	Pnma
Unit cell dimensions	a = 15.0492 (4) Å
	b = 9.0381 (2) Å
	c = 11.6981 (3) Å
Volume	1591.13 (7) Å^3
Z	4
Density (calculated)	1.304 Mg m^{-3}
Absorption coefficient	0.73 mm^{-1}
F(000)	664
Crystal size	0.55 × 0.44 × 0.33 mm^3
Theta range for data collection	5.9 to 72.5°
Reflections collected	2657
$R[F^2 > 2\sigma(F^2)] = 0.035$	H-atom parameters constrained
Largest diff. peak and hole	−0.21 and –0.16 e$^-$ Å^{-3}

Supplementary Table 11. Bond lengths [Å] and angles [°] for **13b**.

O(1)-C(8)	1.209 (2)
O(2)-C(8)	1.340 (2)
O(2)-C(7)	1.496 (2)
O(3)-C(11)	1.233 (2)
N(1)-N(2)	1.116 (2)
N(1)-C(5)	1.340 (2)
C(5)-C(8)	1.468 (2)
C(5)-C(11)	1.475 (2)
C(6)-C(12)	1.3916 (17)
C(6)-C(6i)	1.394 (2)
C(6)-C(9)	1.5093 (17)
C(7)-C(16)	1.5107 (17)
C(7)-C(16i)	1.5107 (17)
C(7)-C(17)	1.520 (3)
C(9)-C(10)	1.5175 (18)
C(10)-C(10i)	1.490 (2)
C(10)-C(13)	1.5424 (18)
C(11)-C(13)	1.469 (3)
C(12)-C(15)	1.3830 (19)
C(15)-C(15i)	1.380 (3)
C(8)-O(2)-C(7)	122.01 (13)
N(2)-N(1)-C(5)	176.02 (17)
N(1)-C(5)-C(8)	116.02 (15)
N(1)-C(5)-C(11)	111.48 (15)
C(8)-C(5)-C(11)	132.50 (16)
C(12)-C(6)-C(6i)	119.36 (7)
C(12)-C(6)-C(9)	120.21 (11)
C(6i)-C(6)-C(9)	120.42 (7)
O(2)-C(7)-C(16)	109.70 (9)
O(2)-C(7)-C(17)	109.70 (9)

C(16)-C7-C(17)	112.44 (15)
O2-C7-C(17)	101.84 (14)
C(16)-C7-C(17)	111.32 (10)
C(17)-C7-C(17)	111.32 (10)
O(1)-C(8)-O(2)	126.53 (16)
O(1)-C(8)-C(5)	124.10 (16)
O(2)-C(8)-C(5)	109.38 (14)
C(6)-C(9)-C(10)	112.62 (10)
C(10i)-C(10)-C(9)	118.18 (7)
C(10i)-C(10)-C(13)	61.12 (5)
C(9)-C(10)-C(13)	122.96 (12)
O(3)-C(11)-C(13)	123.51 (16)
O(3)-C(11)-C(5)	118.48 (16)
C(13)-C(11)-C(5)	118.01 (15)
C(15)-C(12)-C(6)	120.74 (13)
C(11)-C(13)-C(10)	120.99 (13)
C(11)-C(13)-C(10i)	120.99 (13)
C(10)-C(13)-C(10i)	57.77 (11)
C(15i)-C(15)-C(12)	119.90 (8)

Symmetry code: (i) x, −y+1/2, z.

2.1.4 Triasterane 14a

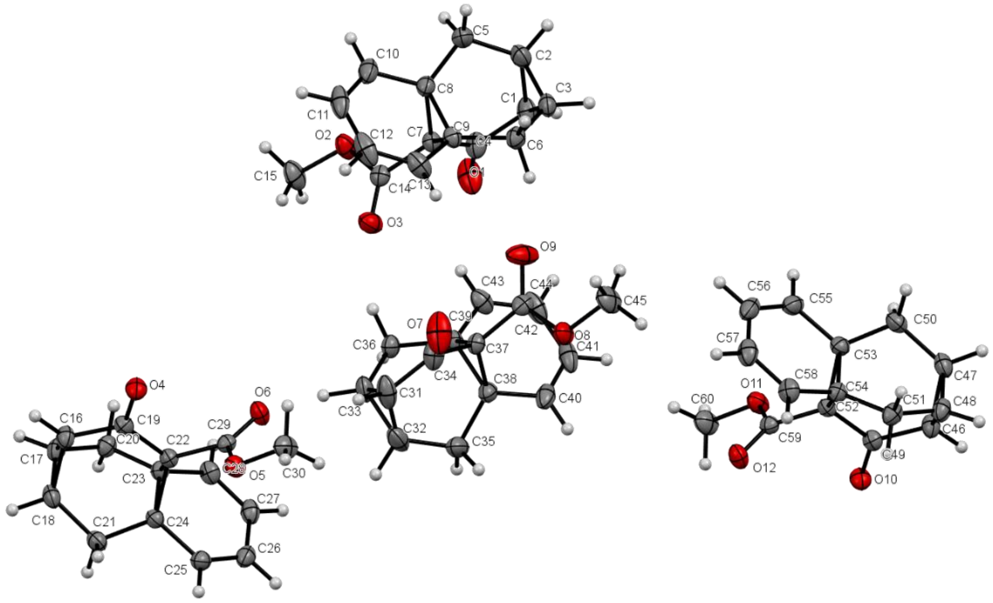

Supplementary Table 12. Crystal data and structure refinement for **14a**.

CCDC number	2128304	
Empirical formula	C15 H14 O3	
Formula weight	242.26	
Temperature	150.01(10) K	
Wavelength	1.54184 Å	
Crystal system	Triclinic	
Space group	P-1	
Unit cell dimensions	a = 11.5389(4) Å	α = 82.438(3)°
	b = 14.7506(4) Å	β = 69.164(3)°
	c = 15.0755(5) Å	γ = 84.945(3)°
Volume	2374.90(14) $Å^3$	
Z	8	
Density (calculated)	1.355 Mg/m^3	
Absorption coefficient	0.765 mm^{-1}	
F(000)	1024	
Crystal size	0.38 x 0.27 x 0.09 mm^3	
Theta range for data collection	3.03 to 67.49°.	
Index ranges	$-11 \le h \le 13$, $-14 \le k \le 17$, $-18 \le l \le 18$	
Reflections collected	16013	
Independent reflections	8552 [*R*(int) = 0.0179]	
Completeness to θ = 67.49°	99.9 %	
Absorption correction	Semi-empirical from equivalents	
Max. and min. transmission	0.9330 and 0.7599	

Refinement method	Full-matrix least-squares on F^2
Data / restraints / parameters	8552 / 0 / 652
Goodness-of-fit on F^2	1.041
Final *R* indices [I>2σ(I)]	*R*1 = 0.0393, w*R*2 = 0.1103
R indices (all data)	*R*1 = 0.0441, w*R*2 = 0.1151
Largest diff. peak and hole	0.326 and –0.326 e^{-} Å^{-3}

Supplementary Table 13. Bond lengths [Å] and angles [°] for **14a**.

O(1)-C(4)	1.2249(17)
O(2)-C(14)	1.3329(15)
O(2)-C(15)	1.4424(16)
O(3)-C(14)	1.1994(16)
O(4)-C(19)	1.2252(15)
O(5)-C(29)	1.3415(15)
O(5)-C(30)	1.4442(15)
O(6)-C(29)	1.1961(15)
O(7)-C(34)	1.2211(18)
O(8)-C(44)	1.3286(15)
O(8)-C(45)	1.4419(16)
O(9)-C(44)	1.1953(16)
O(10)-C(49)	1.2241(15)
O(11)-C(59)	1.3404(15)
O(11)-C(60)	1.4450(15)
O(12)-C(59)	1.1988(15)
C(1)-C(4)	1.4605(19)
C(1)-C(2)	1.529(2)
C(1)-C(3)	1.5331(19)
C(2)-C(3)	1.4756(19)
C(2)-C(5)	1.5071(19)
C(3)-C(6)	1.5052(18)
C(4)-C(7)	1.4845(17)
C(5)-C(8)	1.5109(18)
C(6)-C(9)	1.5176(17)
C(7)-C(14)	1.5023(16)
C(7)-C(8)	1.5426(17)
C(7)-C(9)	1.5437(16)
C(8)-C(10)	1.4747(17)
C(8)-C(9)	1.5254(17)

C(9)-C(13)	1.4762(17)
C(10)-C(11)	1.335(2)
C(11)-C(12)	1.441(2)
C(12)-C(13)	1.339(2)
C(16)-C(19)	1.4642(17)
C(16)-C(18)	1.5304(18)
C(16)-C(17)	1.5353(17)
C(17)-C(18)	1.4869(19)
C(17)-C(20)	1.5080(17)
C(18)-C(21)	1.5057(18)
C(19)-C(22)	1.4847(16)
C(20)-C(23)	1.5156(16)
C(21)-C(24)	1.5128(16)
C(22)-C(29)	1.5016(16)
C(22)-C(24)	1.5471(16)
C(22)-C(23)	1.5482(16)
C(23)-C(28)	1.4817(16)
C(23)-C(24)	1.5267(16)
C(24)-C(25)	1.4768(17)
C(25)-C(26)	1.3398(19)
C(26)-C(27)	1.4486(19)
C(27)-C(28)	1.3376(19)
C(31)-C(34)	1.463(2)
C(31)-C(32)	1.522(2)
C(31)-C(33)	1.5273(19)
C(32)-C(33)	1.483(2)
C(32)-C(35)	1.503(2)
C(33)-C(36)	1.5038(18)
C(34)-C(37)	1.4856(17)
C(35)-C(38)	1.5090(18)
C(36)-C(39)	1.5161(16)
C(37)-C(44)	1.5020(17)
C(37)-C(39)	1.5402(16)
C(37)-C(38)	1.5447(17)
C(38)-C(40)	1.4761(18)
C(38)-C(39)	1.5285(17)
C(39)-C(43)	1.4743(17)
C(40)-C(41)	1.337(2)
C(41)-C(42)	1.442(2)

C(42)-C(43)	1.335(2)
C(46)-C(49)	1.4632(17)
C(46)-C(47)	1.5299(18)
C(46)-C(48)	1.5358(17)
C(47)-C(48)	1.486(2)
C(47)-C(50)	1.5038(19)
C(48)-C(51)	1.5054(18)
C(49)-C(52)	1.4874(16)
C(50)-C(53)	1.5124(17)
C(51)-C(54)	1.5142(17)
C(52)-C(59)	1.5003(16)
C(52)-C(53)	1.5473(16)
C(52)-C(54)	1.5485(16)
C(53)-C(55)	1.4769(17)
C(53)-C(54)	1.5262(17)
C(54)-C(58)	1.4792(17)
C(55)-C(56)	1.339(2)
C(56)-C(57)	1.447(2)
C(57)-C(58)	1.338(2)
C(14)-O(2)-C(15)	115.75(10)
C(29)-O(5)-C(30)	114.48(10)
C(44)-O(8)-C(45)	115.75(11)
C(59)-O(11)-C(60)	114.74(10)
C(4)-C(1)-C(2)	117.82(11)
C(4)-C(1)-C(3)	116.60(11)
C(2)-C(1)-C(3)	57.61(9)
C(3)-C(2)-C(5)	119.85(12)
C(3)-C(2)-C(1)	61.33(9)
C(5)-C(2)-C(1)	118.49(11)
C(2)-C(3)-C(6)	120.66(11)
C(2)-C(3)-C(1)	61.06(9)
C(6)-C(3)-C(1)	117.09(11)
O(1)-C(4)-C(1)	122.70(12)
O(1)-C(4)-C(7)	120.44(12)
C(1)-C(4)-C(7)	116.85(11)
C(2)-C(5)-C(8)	112.53(11)
C(3)-C(6)-C(9)	112.98(10)
C(4)-C(7)-C(14)	112.76(10)

C(4)-C(7)-C(8)	118.30(10)
C(14)-C(7)-C(8)	119.85(10)
C(4)-C(7)-C(9)	116.30(10)
C(14)-C(7)-C(9)	120.61(10)
C(8)-C(7)-C(9)	59.24(8)
C(10)-C(8)-C(5)	116.00(11)
C(10)-C(8)-C(9)	116.26(11)
C(5)-C(8)-C(9)	119.43(10)
C(10)-C(8)-C(7)	116.39(10)
C(5)-C(8)-C(7)	116.89(11)
C(9)-C(8)-C(7)	60.42(8)
C(13)-C(9)-C(6)	115.47(11)
C(13)-C(9)-C(8)	116.66(11)
C(6)-C(9)-C(8)	118.58(10)
C(13)-C(9)-C(7)	118.41(10)
C(6)-C(9)-C(7)	116.31(10)
C(8)-C(9)-C(7)	60.34(8)
C(11)-C(10)-C(8)	122.13(14)
C(10)-C(11)-C(12)	121.33(13)
C(13)-C(12)-C(11)	121.78(13)
C(12)-C(13)-C(9)	121.51(14)
O(3)-C(14)-O(2)	123.67(11)
O(3)-C(14)-C(7)	125.51(11)
O(2)-C(14)-C(7)	110.81(10)
C(19)-C(16)-C(18)	117.84(11)
C(19)-C(16)-C(17)	115.75(10)
C(18)-C(16)-C(17)	58.03(8)
C(18)-C(17)-C(20)	120.22(11)
C(18)-C(17)-C(16)	60.82(8)
C(20)-C(17)-C(16)	117.07(10)
C(17)-C(18)-C(21)	119.98(11)
C(17)-C(18)-C(16)	61.15(8)
C(21)-C(18)-C(16)	118.03(10)
O(4)-C(19)-C(16)	122.48(11)
O(4)-C(19)-C(22)	120.23(11)
C(16)-C(19)-C(22)	117.26(10)
C(17)-C(20)-C(23)	113.47(10)
C(18)-C(21)-C(24)	113.21(10)
C(19)-C(22)-C(29)	111.86(10)

C(19)-C(22)-C(24)	118.17(10)
C(29)-C(22)-C(24)	121.33(10)
C(19)-C(22)-C(23)	116.48(10)
C(29)-C(22)-C(23)	120.58(10)
C(24)-C(22)-C(23)	59.11(7)
C(28)-C(23)-C(20)	115.75(10)
C(28)-C(23)-C(24)	116.32(10)
C(20)-C(23)-C(24)	119.28(10)
C(28)-C(23)-C(22)	118.54(10)
C(20)-C(23)-C(22)	115.29(10)
C(24)-C(23)-C(22)	60.41(7)
C(25)-C(24)-C(21)	115.68(10)
C(25)-C(24)-C(23)	116.57(10)
C(21)-C(24)-C(23)	118.86(10)
C(25)-C(24)-C(22)	117.54(10)
C(21)-C(24)-C(22)	116.53(10)
C(23)-C(24)-C(22)	60.49(7)
C(26)-C(25)-C(24)	122.04(12)
C(25)-C(26)-C(27)	121.42(12)
C(28)-C(27)-C(26)	121.43(12)
C(27)-C(28)-C(23)	122.05(12)
O(6)-C(29)-O(5)	123.87(11)
O(6)-C(29)-C(22)	125.24(11)
O(5)-C(29)-C(22)	110.80(10)
C(34)-C(31)-C(32)	117.45(12)
C(34)-C(31)-C(33)	116.63(12)
C(32)-C(31)-C(33)	58.20(10)
C(33)-C(32)-C(35)	120.36(12)
C(33)-C(32)-C(31)	61.06(10)
C(35)-C(32)-C(31)	118.04(12)
C(32)-C(33)-C(36)	119.94(11)
C(32)-C(33)-C(31)	60.74(11)
C(36)-C(33)-C(31)	118.00(11)
O(7)-C(34)-C(31)	122.61(13)
O(7)-C(34)-C(37)	120.28(13)
C(31)-C(34)-C(37)	117.10(12)
C(32)-C(35)-C(38)	112.82(12)
C(33)-C(36)-C(39)	112.91(10)
C(34)-C(37)-C(44)	112.45(10)

C(34)-C(37)-C(39)	116.86(10)
C(44)-C(37)-C(39)	120.49(10)
C(34)-C(37)-C(38)	116.97(11)
C(44)-C(37)-C(38)	121.14(10)
C(39)-C(37)-C(38)	59.40(8)
C(40)-C(38)-C(35)	116.21(11)
C(40)-C(38)-C(39)	115.50(12)
C(35)-C(38)-C(39)	118.99(10)
C(40)-C(38)-C(37)	117.27(10)
C(35)-C(38)-C(37)	117.07(11)
C(39)-C(38)-C(37)	60.15(8)
C(43)-C(39)-C(36)	115.08(11)
C(43)-C(39)-C(38)	116.86(11)
C(36)-C(39)-C(38)	118.93(10)
C(43)-C(39)-C(37)	118.17(10)
C(36)-C(39)-C(37)	116.56(10)
C(38)-C(39)-C(37)	60.44(8)
C(41)-C(40)-C(38)	122.64(14)
C(40)-C(41)-C(42)	121.34(13)
C(43)-C(42)-C(41)	121.26(13)
C(42)-C(43)-C(39)	122.05(14)
O(9)-C(44)-O(8)	123.78(12)
O(9)-C(44)-C(37)	124.74(12)
O(8)-C(44)-C(37)	111.44(10)
C(49)-C(46)-C(47)	117.87(11)
C(49)-C(46)-C(48)	115.84(10)
C(47)-C(46)-C(48)	58.00(9)
C(48)-C(47)-C(50)	119.96(11)
C(48)-C(47)-C(46)	61.20(9)
C(50)-C(47)-C(46)	118.21(11)
C(47)-C(48)-C(51)	120.44(11)
C(47)-C(48)-C(46)	60.80(9)
C(51)-C(48)-C(46)	116.93(10)
O(10)-C(49)-C(46)	122.58(11)
O(10)-C(49)-C(52)	120.16(11)
C(46)-C(49)-C(52)	117.22(10)
C(47)-C(50)-C(53)	113.07(11)
C(48)-C(51)-C(54)	113.35(10)
C(49)-C(52)-C(59)	112.03(10)

C(49)-C(52)-C(53)	118.16(10)
C(59)-C(52)-C(53)	121.34(10)
C(49)-C(52)-C(54)	116.03(10)
C(59)-C(52)-C(54)	120.76(10)
C(53)-C(52)-C(54)	59.08(7)
C(55)-C(53)-C(50)	115.32(11)
C(55)-C(53)-C(54)	116.38(11)
C(50)-C(53)-C(54)	119.00(11)
C(55)-C(53)-C(52)	118.15(10)
C(50)-C(53)-C(52)	116.57(10)
C(54)-C(53)-C(52)	60.50(7)
C(58)-C(54)-C(51)	115.50(10)
C(58)-C(54)-C(53)	116.48(11)
C(51)-C(54)-C(53)	119.28(10)
C(58)-C(54)-C(52)	118.45(10)
C(51)-C(54)-C(52)	115.62(10)
C(53)-C(54)-C(52)	60.42(7)
C(56)-C(55)-C(53)	122.18(13)
C(55)-C(56)-C(57)	121.37(12)
C(58)-C(57)-C(56)	121.48(13)
C(57)-C(58)-C(54)	122.02(12)
O(12)-C(59)-O(11)	123.97(11)
O(12)-C(59)-C(52)	125.09(11)
O(11)-C(59)-C(52)	110.87(10)

Symmetry *trans*formations used to generate equivalent atoms:

2.1.5 Triasterane 14b

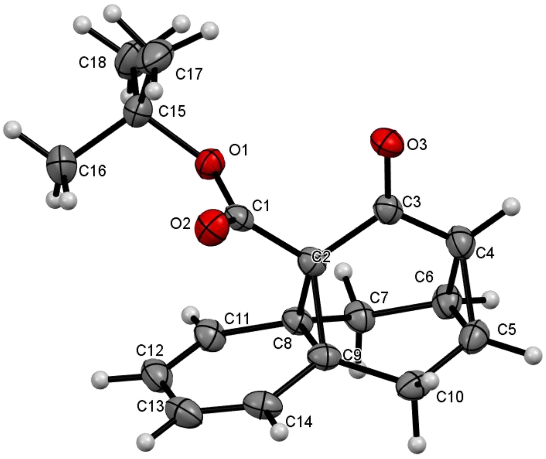

Supplementary Table 14. Crystal data and structure refinement for **14b**.

CCDC number	2128306	
Empirical formula	C18 H20 O3	
Formula weight	284.34	
Temperature	150.01(10) K	
Wavelength	1.54184 Å	
Crystal system	Monoclinic	
Space group	$P2_1/n$	
Unit cell dimensions	a = 6.04138(18) Å	α = 90°
	b = 14.0715(5) Å	β = 93.120(3)°
	C = 17.1606(5) Å	γ = 90°
Volume	1456.69(8) Å^3	
Z	4	
Density (calculated)	1.297 Mg/m^3	
Absorption coefficient	0.698 mm^{-1}	
F(000)	608	
Crystal size	0.28 x 0.16 x 0.12 mm^3	
Theta range for data collection	4.07 to 67.48°.	
Index ranges	$-5 \le h \le 7$, $-16 \le k \le 14$, $-20 \le l \le 20$	
Reflections collected	5300	
Independent reflections	2627 [*R*(int) = 0.0275]	
Completeness to θ = 67.48°	99.7 %	
Absorption correction	Semi-empirical from equivalents	
Max. and min. transmission	0.9221 and 0.8285	
Refinement method	Full-matrix least-squares on F^2	
Data / restraints / parameters	2627 / 0 / 193	

Goodness-of-fit on F^2	1.120
Final *R* indices [$I>2\sigma(I)$]	*R*1 = 0.0628, w*R*2 = 0.1658
R indices (all data)	*R*1 = 0.0688, w*R*2 = 0.1688
Largest diff. peak and hole	0.283 and –0.272 e$^-$ Å^{-3}

Supplementary Table 15. Bond lengths [Å] and angles [°] for **14b**.

O(1)-C(1)	1.344(3)
O(1)-C(15)	1.469(3)
O(2)-C(1)	1.203(3)
O(3)-C(3)	1.227(3)
C(1)-C(2)	1.496(4)
C(2)-C(3)	1.489(4)
C(2)-C(8)	1.547(4)
C(2)-C(9)	1.550(4)
C(3)-C(4)	1.453(4)
C(4)-C(6)	1.531(4)
C(4)-C(5)	1.543(4)
C(5)-C(6)	1.492(4)
C(5)-C(10)	1.502(4)
C(6)-C(7)	1.507(4)
C(7)-C(8)	1.507(4)
C(8)-C(11)	1.477(4)
C(8)-C(9)	1.527(4)
C(9)-C(14)	1.464(4)
C(9)-C(10)	1.519(4)
C(11)-C(12)	1.335(4)
C(12)-C(13)	1.453(4)
C(13)-C(14)	1.338(5)
C(15)-C(18)	1.516(5)
C(15)-C(16)	1.517(4)
C(15)-C(17)	1.518(4)
C(1)-O(1)-C(15)	121.7(2)
O(2)-C(1)-O(1)	125.8(3)
O(2)-C(1)-C(2)	126.0(3)
O(1)-C(1)-C(2)	108.2(2)
C(3)-C(2)-C(1)	112.2(2)
C(3)-C(2)-C(8)	118.6(2)

C(1)-C(2)-C(8)	118.6(2)
C(3)-C(2)-C(9)	117.0(2)
C(1)-C(2)-C(9)	121.9(2)
C(8)-C(2)-C(9)	59.08(17)
O(3)-C(3)-C(4)	123.6(3)
O(3)-C(3)-C(2)	119.5(3)
C(4)-C(3)-C(2)	116.9(2)
C(3)-C(4)-C(6)	118.1(2)
C(3)-C(4)-C(5)	115.9(3)
C(6)-C(4)-C(5)	58.09(19)
C(6)-C(5)-C(10)	119.6(3)
C(6)-C(5)-C(4)	60.55(19)
C(10)-C(5)-C(4)	117.9(2)
C(5)-C(6)-C(7)	119.2(3)
C(5)-C(6)-C(4)	61.4(2)
C(7)-C(6)-C(4)	118.6(2)
C(8)-C(7)-C(6)	113.1(2)
C(11)-C(8)-C(7)	117.2(2)
C(11)-C(8)-C(9)	115.3(2)
C(7)-C(8)-C(9)	119.3(2)
C(11)-C(8)-C(2)	115.5(2)
C(7)-C(8)-C(2)	116.7(2)
C(9)-C(8)-C(2)	60.57(17)
C(14)-C(9)-C(10)	115.7(2)
C(14)-C(9)-C(8)	117.2(2)
C(10)-C(9)-C(8)	117.6(2)
C(14)-C(9)-C(2)	119.3(2)
C(10)-C(9)-C(2)	115.5(2)
C(8)-C(9)-C(2)	60.35(17)
C(5)-C(10)-C(9)	114.0(2)
C(12)-C(11)-C(8)	122.9(3)
C(11)-C(12)-C(13)	121.2(3)
C(14)-C(13)-C(12)	120.8(3)
C(13)-C(14)-C(9)	122.4(3)
O(1)-C(15)-C(18)	102.1(2)
O(1)-C(15)-C(16)	108.9(2)
C(18)-C(15)-C(16)	110.3(3)
O(1)-C(15)-C(17)	110.0(2)
C(18)-C(15)-C(17)	111.5(3)

C(16)-C(15)-C(17)	113.4(3)

Symmetry *trans*formations used to generate equivalent atoms:

2.1.6 Methyl 1-oxo-1,2-dihydro-9a*H*-2,9-ethenobenzo[7]annulene-9a-carboxylate (15)

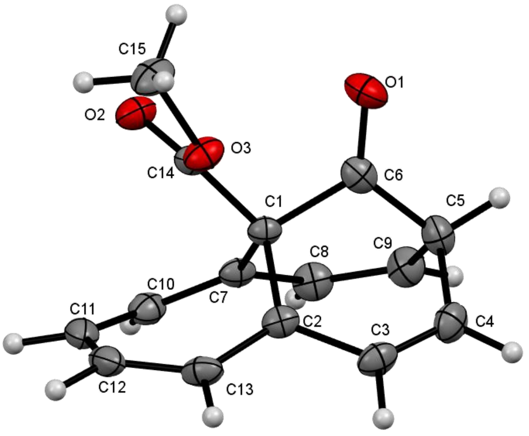

Supplementary Table 16. Crystal data and structure refinement for **15**.

CCDC number	2128303	
Empirical formula	C15 H12 O3	
Formula weight	240.25	
Temperature	150.01(10) K	
Wavelength	1.54184 Å	
Crystal system	Orthorhombic	
Space group	$Pna2_1$	
Unit cell dimensions	a = 12.5081(2) Å	α = 90°
	b = 13.8918(2) Å	β = 90°
	c = 6.63025(10) Å	γ = 90°
Volume	1152.07(3) $Å^3$	
Z	4	
Density (calculated)	1.385 Mg/m^3	
Absorption coefficient	0.788 mm^{-1}	
F(000)	504	
Crystal size	0.27 x 0.12 x 0.02 mm^3	
Theta range for data collection	4.76 to 67.42°.	
Index ranges	$-14 \le h \le 14$, $-16 \le k \le 16$, $-6 \le l \le 7$	
Reflections collected	7091	
Independent reflections	1779 [*R*(int) = 0.0181]	
Completeness to θ = 67.42°	100.0 %	
Absorption correction	Semi-empirical from equivalents	
Max. and min. transmission	0.9860 and 0.8167	
Refinement method	Full-matrix least-squares on F^2	

Data / restraints / parameters	1779 / 1 / 176
Goodness-of-fit on F^2	1.083
Final *R* indices [*I*>2σ(I)]	*R*1 = 0.0269, w*R*2 = 0.0691
R indices (all data)	*R*1 = 0.0278, w*R*2 = 0.0697
Absolute structure parameter	0.17(19)
Largest diff. peak and hole	0.251 and –0.161 e^{-} Å^{-3}

Supplementary Table 17. Bond lengths [Å] and angles [°] for **15**.

O(1)-C(6)	1.2088(19)
O(2)-C(14)	1.200(2)
O(3)-C(14)	1.3301(18)
O(3)-C(15)	1.4494(18)
C(1)-C(2)	1.512(2)
C(1)-C(7)	1.5187(18)
C(1)-C(6)	1.5323(18)
C(1)-C(14)	1.534(2)
C(2)-C(13)	1.370(2)
C(2)-C(3)	1.447(2)
C(3)-C(4)	1.342(2)
C(3)-H(3A)	1.03(2)
C(4)-C(5)	1.510(2)
C(4)-H(4A)	0.9500
C(5)-C(6)	1.494(2)
C(5)-C(9)	1.517(2)
C(5)-H(5A)	1.0000
C(7)-C(10)	1.364(2)
C(7)-C(8)	1.4495(19)
C(8)-C(9)	1.341(2)
C(8)-H(8A)	0.9500
C(9)-H(9A)	0.9500
C(10)-C(11)	1.420(2)
C(10)-H(10A)	0.946(19)
C(11)-C(12)	1.376(3)
C(11)-H(11A)	0.998(19)
C(12)-C(13)	1.416(2)
C(12)-H(12A)	1.002(19)
C(13)-H(13A)	0.9500
C(15)-H(15A)	0.9800

C(15)-H(15B)	0.9800
C(15)-H(15C)	0.9800
C(14)-O(3)-C(15)	116.34(13)
C(2)-C(1)-C(7)	100.66(11)
C(2)-C(1)-C(6)	110.12(13)
C(7)-C(1)-C(6)	109.36(11)
C(2)-C(1)-C(14)	115.33(12)
C(7)-C(1)-C(14)	115.16(12)
C(6)-C(1)-C(14)	106.14(12)
C(13)-C(2)-C(3)	122.29(15)
C(13)-C(2)-C(1)	118.80(14)
C(3)-C(2)-C(1)	118.42(13)
C(4)-C(3)-C(2)	122.19(16)
C(4)-C(3)-H(3A)	123.8(10)
C(2)-C(3)-H(3A)	113.9(10)
C(3)-C(4)-C(5)	121.70(15)
C(3)-C(4)-H(4A)	119.2
C(5)-C(4)-H(4A)	119.2
C(6)-C(5)-C(4)	108.67(12)
C(6)-C(5)-C(9)	106.60(13)
C(4)-C(5)-C(9)	109.49(13)
C(6)-C(5)-H(5A)	110.7
C(4)-C(5)-H(5A)	110.7
C(9)-C(5)-H(5A)	110.7
O(1)-C(6)-C(5)	125.06(13)
O(1)-C(6)-C(1)	122.69(14)
C(5)-C(6)-C(1)	112.23(12)
C(10)-C(7)-C(8)	123.13(13)
C(10)-C(7)-C(1)	119.14(13)
C(8)-C(7)-C(1)	117.39(12)
C(9)-C(8)-C(7)	123.44(14)
C(9)-C(8)-H(8A)	118.3
C(7)-C(8)-H(8A)	118.3
C(8)-C(9)-C(5)	120.73(13)
C(8)-C(9)-H(9A)	119.6
C(5)-C(9)-H(9A)	119.6
C(7)-C(10)-C(11)	125.69(15)
C(7)-C(10)-H(10A)	115.9(12)

C(11)-C(10)-H(10A)	118.1(12)
C(12)-C(11)-C(10)	125.31(15)
C(12)-C(11)-H(11A)	117.2(12)
C(10)-C(11)-H(11A)	116.4(11)
C(11)-C(12)-C(13)	125.39(14)
C(11)-C(12)-H(12A)	117.4(12)
C(13)-C(12)-H(12A)	115.8(13)
C(2)-C(13)-C(12)	125.60(16)
C(2)-C(13)-H(13A)	117.2
C(12)-C(13)-H(13A)	117.2
O(2)-C(14)-O(3)	125.13(14)
O(2)-C(14)-C(1)	124.75(14)
O(3)-C(14)-C(1)	109.94(13)
O(3)-C(15)-H(15A)	109.5
O(3)-C(15)-H(15B)	109.5
H(15A)-C(15)-H(15B)	109.5
O(3)-C(15)-H(15C)	109.5
H(15A)-C(15)-H(15C)	109.5
H(15B)-C(15)-H(15C)	109.5

Symmetry *trans*formations used to generate equivalent atoms:

2.1.7 1-Oxo-1,9a-dihydro-2H-2,9-ethenobenzo[7]annulene-2-carboxylic acid (16)

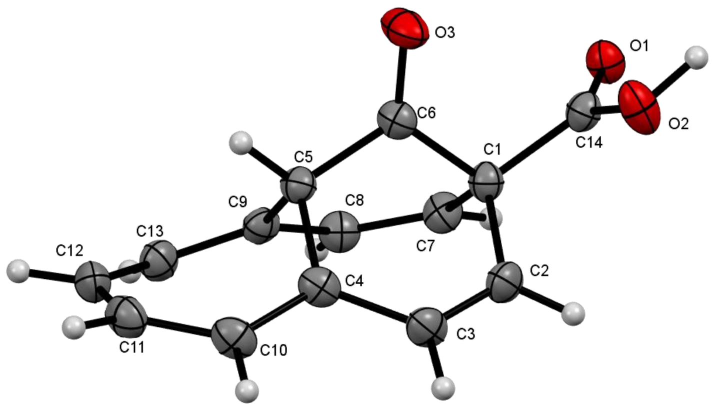

Supplementary Table 18. Crystal data and structure refinement for **16**.

CCDC number	2128308	
Empirical formula	C14 H10 O3	
Formula weight	226.22	
Temperature	150.01(10) K	
Wavelength	1.54184 Å	
Crystal system	Monoclinic	
Space group	$P2_1/n$	
Unit cell dimensions	a = 6.6629(2) Å	α = 90°
	b = 13.3174(4) Å	β = 104.574(3)°
	c = 12.3559(4) Å	γ = 90°
Volume	1061.10(6) $Å^3$	
Z	4	
Density (calculated)	1.416 Mg/m^3	
Absorption coefficient	0.821 mm^{-1}	
F(000)	472	
Crystal size	0.31 x 0.30 x 0.07 mm^3	
Theta range for data collection	4.97 to 67.46°.	
Index ranges	$-5 \le h \le 7$, $-15 \le k \le 15$, $-14 \le l \le 14$	
Reflections collected	3719	
Independent reflections	1901 [*R*(int) = 0.0131]	
Completeness to θ = 67.46°	99.9 %	
Absorption correction	Semi-empirical from equivalents	
Max. and min. transmission	0.9433 and 0.7850	
Refinement method	Full-matrix least-squares on F^2	
Data / restraints / parameters	1901 / 0 / 158	

Goodness-of-fit on F^2	1.087
Final *R* indices [$I>2\sigma(I)$]	*R*1 = 0.0371, w*R*2 = 0.0924
R indices (all data)	*R*1 = 0.0400, w*R*2 = 0.0957
Largest diff. peak and hole	0.228 and –0.201 e^{-} Å^{-3}

Supplementary Table 19. Bond lengths [Å] and angles [°] for **16**.

O(1)-C(14)	1.2222(17)
O(2)-C(14)	1.3096(17)
O(3)-C(6)	1.2054(17)
C(1)-C(14)	1.5136(19)
C(1)-C(7)	1.5239(18)
C(1)-C(6)	1.5255(18)
C(1)-C(2)	1.5256(19)
C(2)-C(3)	1.342(2)
C(3)-C(4)	1.440(2)
C(4)-C(10)	1.364(2)
C(4)-C(5)	1.5115(18)
C(5)-C(6)	1.4947(18)
C(5)-C(9)	1.5085(19)
C(7)-C(8)	1.337(2)
C(8)-C(9)	1.4443(19)
C(9)-C(13)	1.362(2)
C(10)-C(11)	1.424(2)
C(11)-C(12)	1.370(2)
C(12)-C(13)	1.428(2)
C(14)-C(1)-C(7)	112.52(11)
C(14)-C(1)-C(6)	108.72(11)
C(7)-C(1)-C(6)	105.66(11)
C(14)-C(1)-C(2)	113.15(11)
C(7)-C(1)-C(2)	109.29(11)
C(6)-C(1)-C(2)	107.07(11)
C(3)-C(2)-C(1)	121.97(13)
C(2)-C(3)-C(4)	122.92(13)
C(10)-C(4)-C(3)	125.38(13)
C(10)-C(4)-C(5)	117.24(13)
C(3)-C(4)-C(5)	117.05(12)
C(6)-C(5)-C(9)	111.35(11)

C(6)-C(5)-C(4)	111.36(11)
C(9)-C(5)-C(4)	102.09(10)
O(3)-C(6)-C(5)	124.33(12)
O(3)-C(6)-C(1)	123.97(13)
C(5)-C(6)-C(1)	111.67(11)
C(8)-C(7)-C(1)	121.98(12)
C(7)-C(8)-C(9)	122.85(13)
C(13)-C(9)-C(8)	125.73(13)
C(13)-C(9)-C(5)	117.16(12)
C(8)-C(9)-C(5)	116.94(12)
C(4)-C(10)-C(11)	124.18(13)
C(12)-C(11)-C(10)	125.39(13)
C(11)-C(12)-C(13)	125.99(13)
C(9)-C(13)-C(12)	124.29(14)
O(1)-C(14)-O(2)	123.85(13)
O(1)-C(14)-C(1)	122.24(12)
O(2)-C(14)-C(1)	113.89(12)

Symmetry *trans*formations used to generate equivalent atoms:

2.1.8 9-(Carboxymethyl)-7*H*-benzo[7]annulene-2-carboxylic acid (S9)

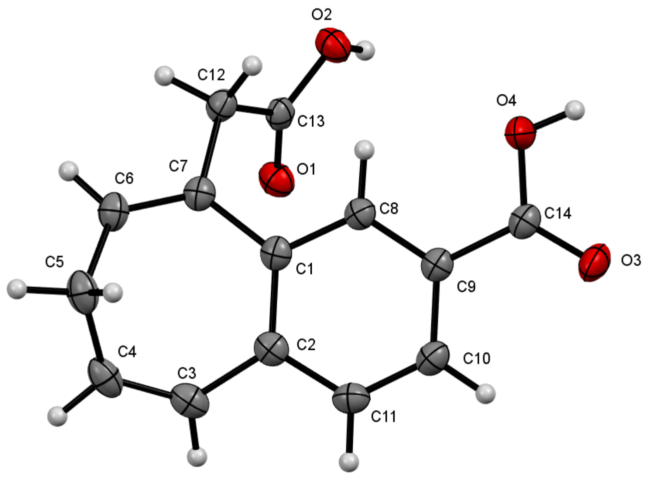

Supplementary Table 20. Crystal data and structure refinement for **S9**.

CCDC number	2128307	
Empirical formula	C14 H12 O4	
Formula weight	244.24	
Temperature	150.00(10) K	
Wavelength	1.54184 Å	
Crystal system	Monoclinic	
Space group	$P2_1/c$	
Unit cell dimensions	a = 13.4532(5) Å	α = 90°
	b = 6.5900(2) Å	β = 110.134(4)°
	c = 13.8727(5) Å	γ = 90°
Volume	1154.74(7) $Å^3$	
Z	4	
Density (calculated)	1.405 Mg/m^3	
Absorption coefficient	0.860 mm^{-1}	
F(000)	512	
Crystal size	0.26 x 0.13 x 0.06 mm^3	
Theta range for data collection	3.50 to 67.48°.	
Index ranges	$-16 \le h \le 15$, $-7 \le k \le 7$, $-16 \le l \le 13$	
Reflections collected	3941	
Independent reflections	2082 [*R*(int) = 0.0155]	
Completeness to θ = 67.48°	99.9 %	
Absorption correction	Semi-empirical from equivalents	
Max. and min. transmission	0.9518 and 0.8099	
Refinement method	Full-matrix least-squares on F^2	
Data / restraints / parameters	2082 / 0 / 171	
Goodness-of-fit on F^2	1.090	

Final *R* indices [*I*>2σ(I)]	*R*1 = 0.0332, w*R*2 = 0.0819
R indices (all data)	*R*1 = 0.0368, w*R*2 = 0.0848
Largest diff. peak and hole	0.152 and –0.194 $e^{–}$ $Å^{–3}$

Supplementary Table 21. Bond lengths [Å] and angles [°] for **S9**.

O(1)-C(13)	1.2333(15)
O(2)-C(13)	1.3033(14)
O(3)-C(14)	1.2170(14)
O(4)-C(14)	1.3173(15)
C(1)-C(8)	1.4018(16)
C(1)-C(2)	1.4207(17)
C(1)-C(7)	1.4838(16)
C(2)-C(11)	1.4044(17)
C(2)-C(3)	1.4645(17)
C(3)-C(4)	1.3349(18)
C(4)-C(5)	1.495(2)
C(5)-C(6)	1.5045(18)
C(6)-C(7)	1.3363(16)
C(7)-C(12)	1.5131(16)
C(8)-C(9)	1.3908(16)
C(9)-C(10)	1.3930(17)
C(9)-C(14)	1.4815(16)
C(10)-C(11)	1.3766(17)
C(12)-C(13)	1.5021(16)
C(8)-C(1)-C(2)	117.87(10)
C(8)-C(1)-C(7)	118.44(11)
C(2)-C(1)-C(7)	123.43(10)
C(11)-C(2)-C(1)	118.55(11)
C(11)-C(2)-C(3)	116.44(11)
C(1)-C(2)-C(3)	124.94(11)
C(4)-C(3)-C(2)	126.61(12)
C(3)-C(4)-C(5)	122.91(11)
C(4)-C(5)-C(6)	110.33(10)
C(7)-C(6)-C(5)	124.24(11)
C(6)-C(7)-C(1)	124.15(11)
C(6)-C(7)-C(12)	118.74(11)
C(1)-C(7)-C(12)	117.08(10)

C(9)-C(8)-C(1)	122.30(11)
C(8)-C(9)-C(10)	119.47(11)
C(8)-C(9)-C(14)	121.24(11)
C(10)-C(9)-C(14)	119.27(10)
C(11)-C(10)-C(9)	119.14(11)
C(10)-C(11)-C(2)	122.53(12)
C(13)-C(12)-C(7)	114.97(10)
O(1)-C(13)-O(2)	123.53(11)
O(1)-C(13)-C(12)	122.33(11)
O(2)-C(13)-C(12)	114.12(11)
O(3)-C(14)-O(4)	122.07(11)
O(3)-C(14)-C(9)	124.02(11)
O(4)-C(14)-C(9)	113.90(10)

Symmetry *trans*formations used to generate equivalent atoms:

2.1.9 Triasterane S11

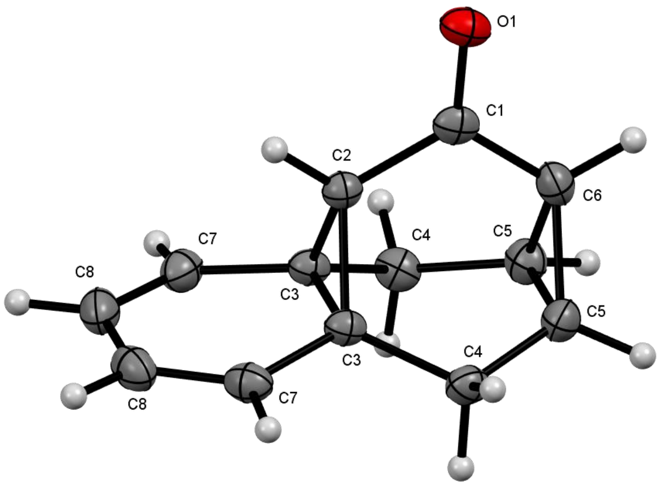

Supplementary Table 22. Crystal data and structure refinement for **S11**.

CCDC number	2133482	
Empirical formula	C13 H12 O	
Formula weight	184.23	
Temperature	150.01(10) K	
Wavelength	1.54184 Å	
Crystal system	Orthorhombic	
Space group	$Cmc2_1$	
Unit cell dimensions	a = 9.2806(3) Å	α = 90°.
	b = 12.9060(4) Å	β = 90°.
	c = 7.4518(3) Å	γ = 90°.
Volume	892.54(5) $Å^3$	
Z	4	
Density (calculated)	1.371 Mg/m^3	
Absorption coefficient	0.664 mm^{-1}	
F(000)	392	
Crystal size	0.21 x 0.10 x 0.07 mm^3	
Theta range for data collection	5.87 to 67.42°	
Index ranges	$-11 \le h \le 10$, $-15 \le k \le 15$, $-8 \le l \le 8$	
Reflections collected	2849	
Independent reflections	813 [*R*(int) = 0.0177]	
Completeness to θ = 67.42°	100.0 %	
Absorption correction	Semi-empirical from equivalents	
Max. and min. transmission	0.9525 and 0.8732	
Refinement method	Full-matrix least-squares on F^2	
Data / restraints / parameters	813 / 1 / 70	
Goodness-of-fit on F^2	1.103	

Final *R* indices [I>2σ(I)]	*R*1 = 0.0272, w*R*2 = 0.0715
R indices (all data)	*R*1 = 0.0274, w*R*2 = 0.0717
Absolute structure parameter	0.2(3)
Largest diff. peak and hole	0.097 and –0.170 e^{-} $Å^{-3}$

Supplementary Table 23. Bond lengths [Å] and angles [°] for **S11**.

O(1)-C(1)	1.228(2)
C(1)-C(6)	1.461(3)
C(1)-C(2)	1.469(2)
C(2)-C(3)#1	1.5473(18)
C(2)-C(3)	1.5473(18)
C(3)-C(7)	1.4728(15)
C(3)-C(4)	1.5116(16)
C(3)-C(3)#1	1.531(2)
C(4)-C(5)	1.5062(16)
C(5)-C(5)#1	1.490(3)
C(5)-C(6)	1.537(2)
C(6)-C(5)#1	1.537(2)
C(7)-C(8)	1.3373(18)
C(8)-C(8)#1	1.452(3)
O(1)-C(1)-C(6)	122.81(16)
O(1)-C(1)-C(2)	120.83(16)
C(6)-C(1)-C(2)	116.35(16)
C(1)-C(2)-C(3)#1	118.50(12)
C(1)-C(2)-C(3)	118.50(12)
C(3)#1-C(2)-C(3)	59.32(10)
C(7)-C(3)-C(4)	117.55(10)
C(7)-C(3)-C(3)#1	116.43(7)
C(4)-C(3)-C(3)#1	117.73(6)
C(7)-C(3)-C(2)	115.60(11)
C(4)-C(3)-C(2)	116.71(10)
C(3)#1-C(3)-C(2)	60.34(5)
C(5)-C(4)-C(3)	113.60(10)
C(5)#1-C(5)-C(4)	118.73(6)
C(5)#1-C(5)-C(6)	61.02(6)
C(4)-C(5)-C(6)	118.39(12)
C(1)-C(6)-C(5)#1	117.49(12)

C(1)-C(6)-C(5)	117.49(12)
C(5)#1-C(6)-C(5)	57.96(12)
C(8)-C(7)-C(3)	121.76(12)
C(7)-C(8)-C(8)#1	121.32(7)

Symmetry *trans*formations used to generate equivalent atoms:

#1 -x,y,z

2.2 Spectral Data

2.2.1 1,4-Dihydronaphthalene (8)

^{1}H NMR

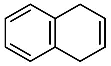

8

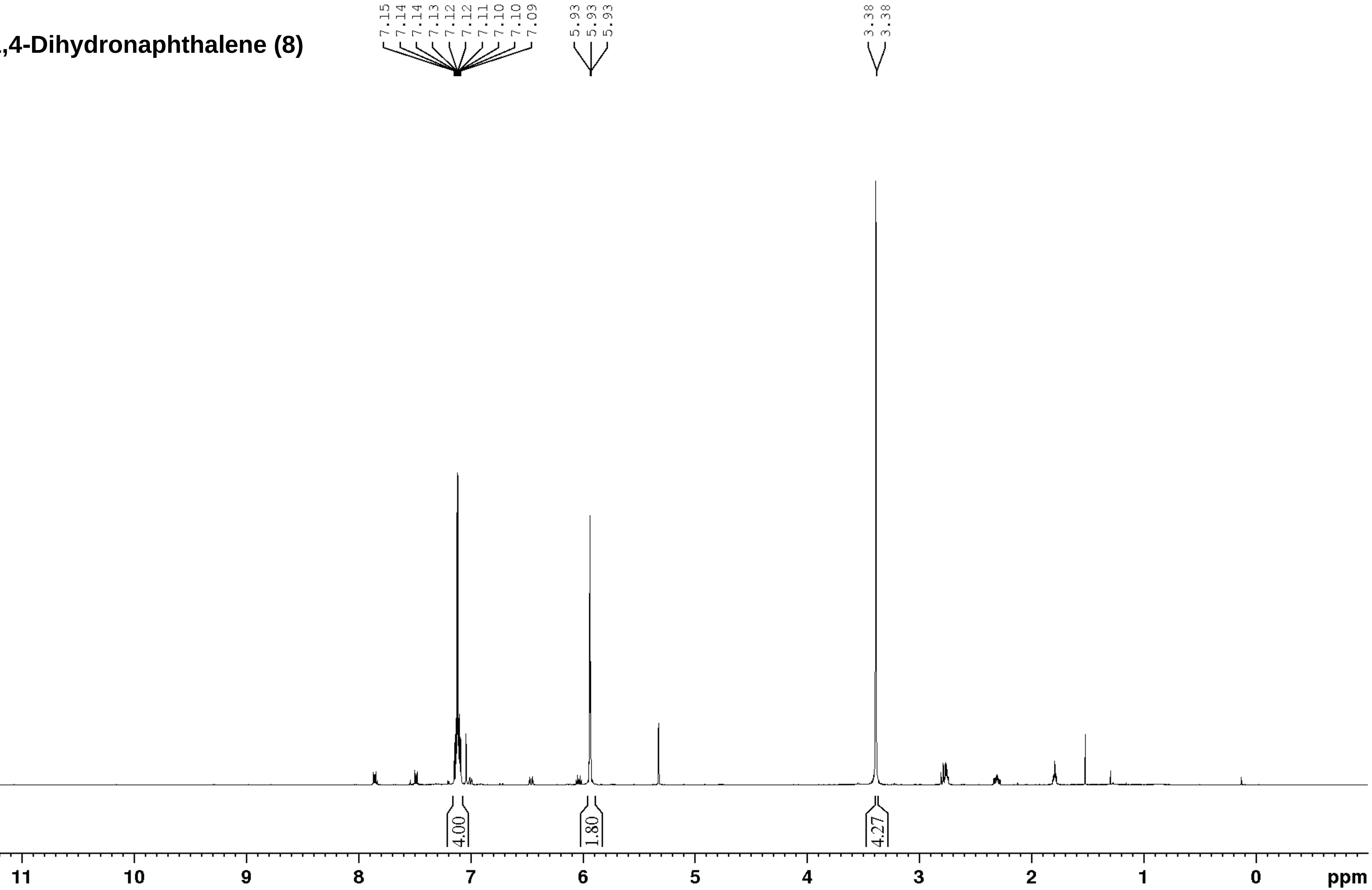

^{13}C NMR

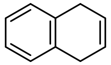

8

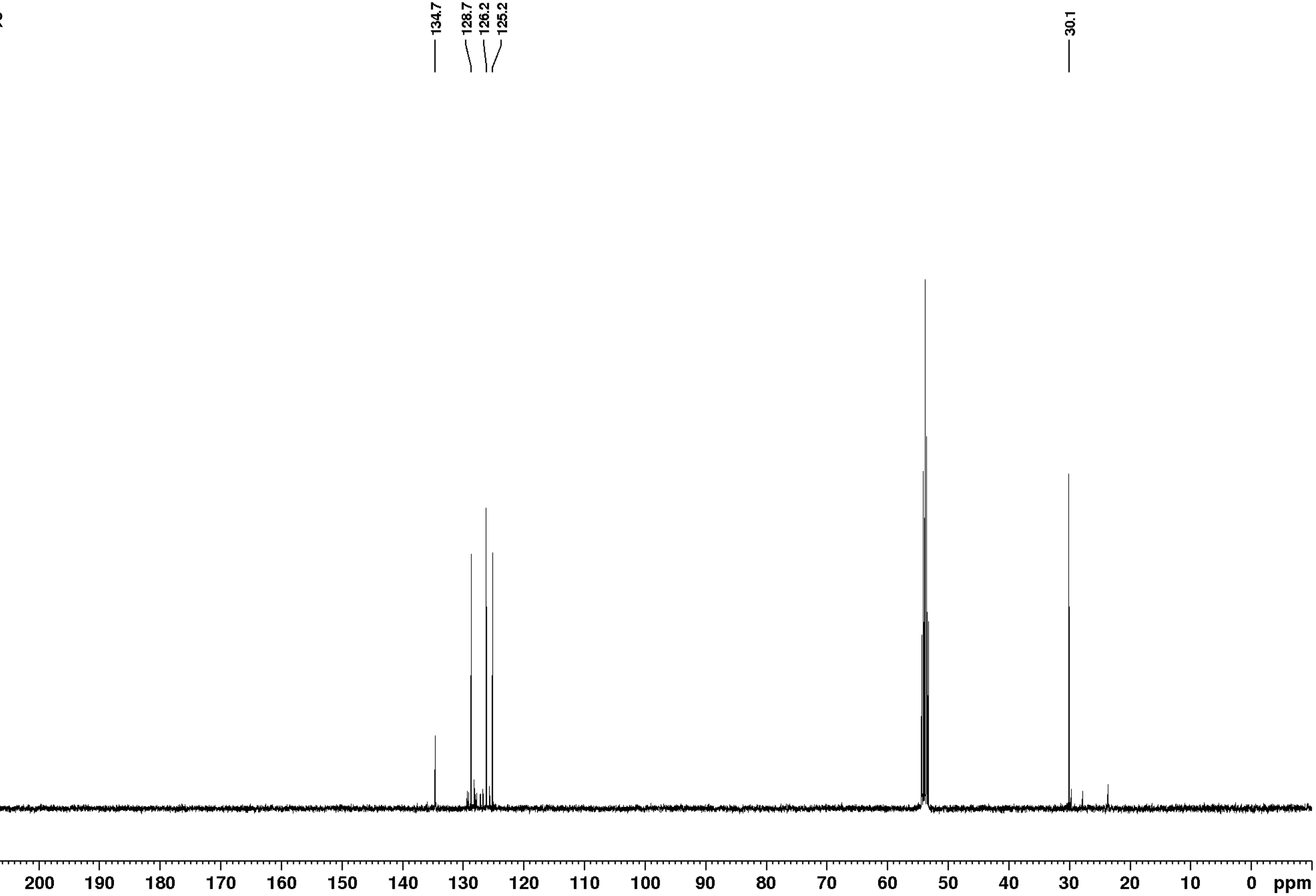
134.7
128.7
126.2
125.2
30.1
210 200 190 180 170 160 150 140 130 120 110 100 90 80 70 60 50 40 30 20 10 0 ppm

^1H, ^1H COSY

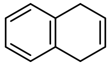

8

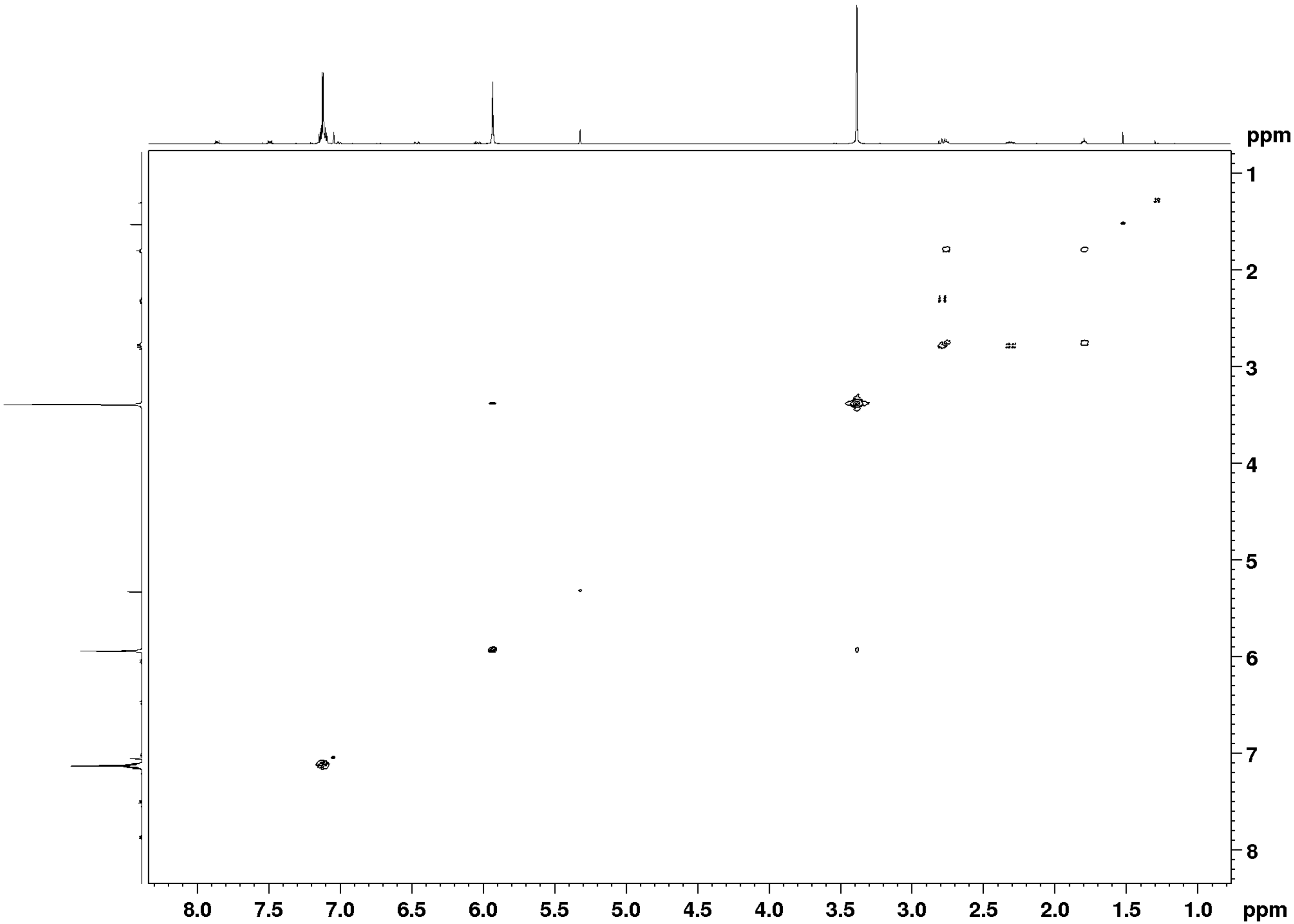

^{1}H, ^{13}C HSQC

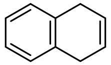

8

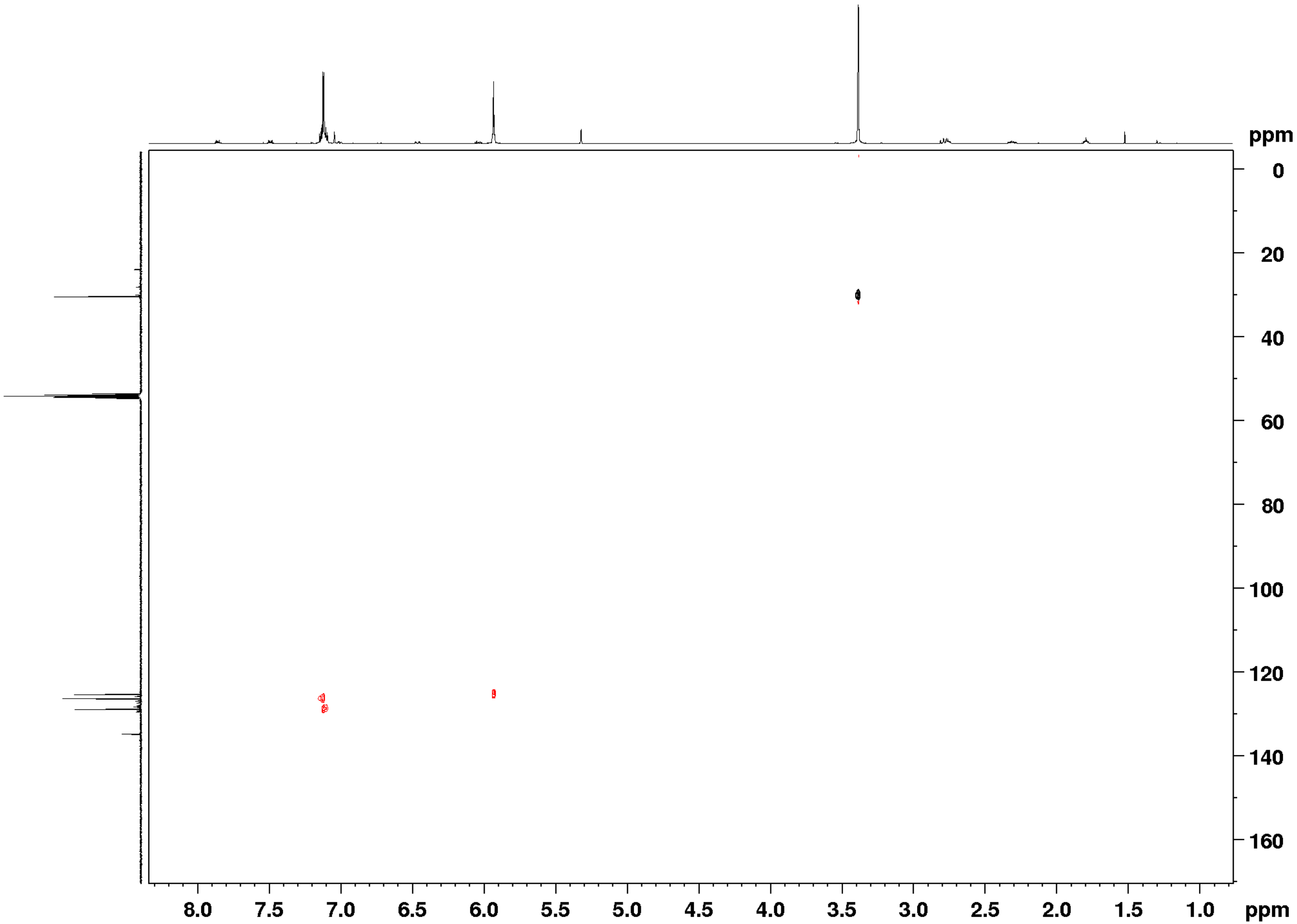

ppm
0
20
40
60
80
100
120
140
160
8.0
7.5
7.0
6.5
6.0
5.5
5.0
4.5
4.0
3.5
3.0
2.5
2.0
1.5
1.0
ppm

^{1}H, ^{13}C HMBC

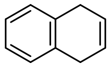

8

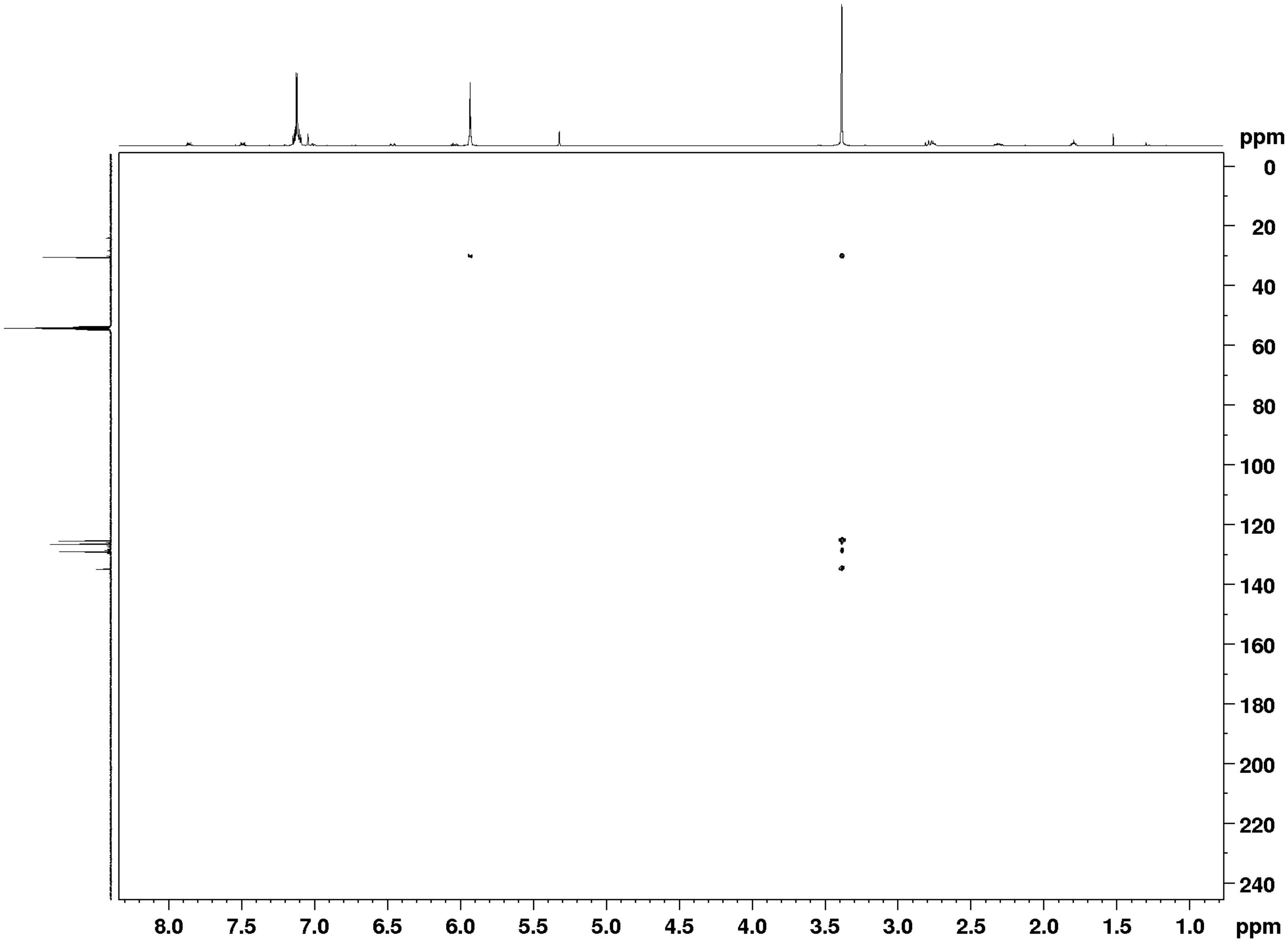
ppm
0
20
40
60
80
100
120
140
160
180
200
220
240
8.0
7.5
7.0
6.5
6.0
5.5
5.0
4.5
4.0
3.5
3.0
2.5
2.0
1.5
1.0
ppm

2.2.2 1-((1*r*,1a*R*,7a*S*)-1a,2,7,7a-Tetrahydro-1*H*-cyclopropa[b]naphthalen-1-yl)propan-1-on (10)

^{1}H NMR

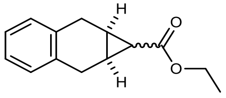

10

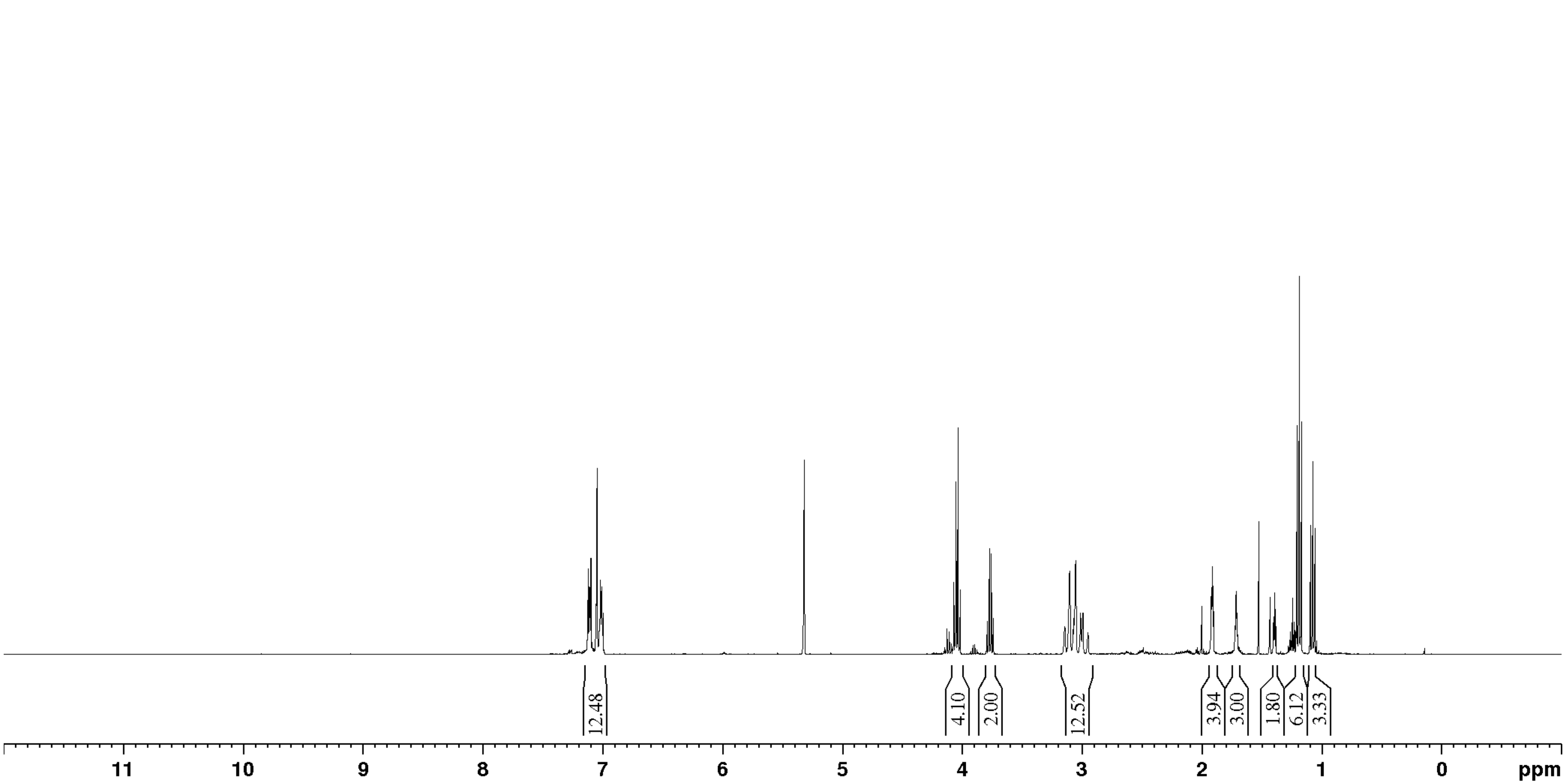

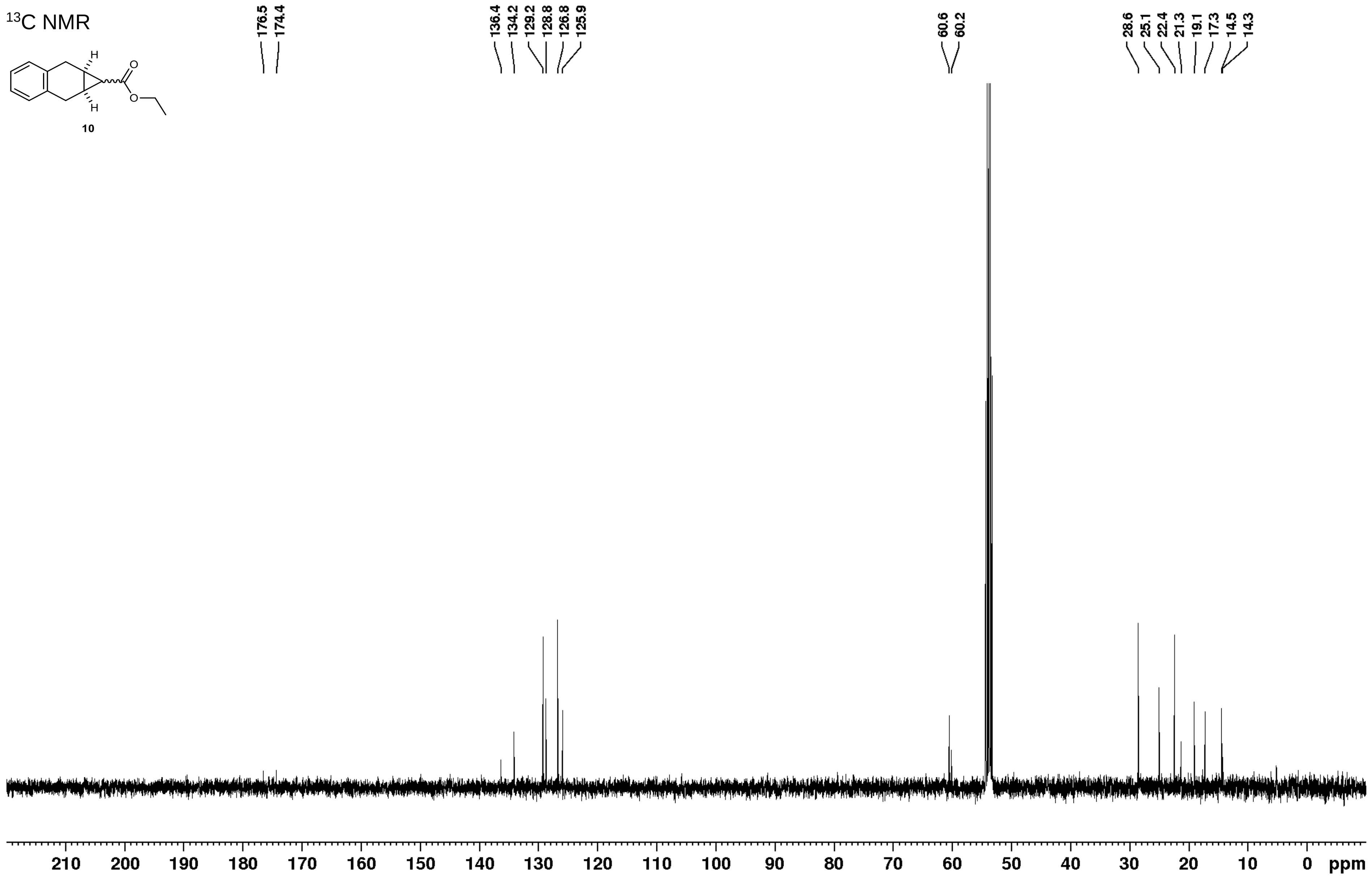

13C NMR
10
176.5
174.4
136.4
134.2
129.2
128.8
126.8
125.9
60.6
60.2
28.6
25.1
22.4
21.3
19.1
17.3
14.5
14.3
210
200
190
180
170
160
150
140
130
120
110
100
90
80
70
60
50
40
30
20
10
0
ppm

^{1}H, ^{1}H COSY

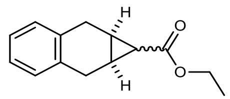

10

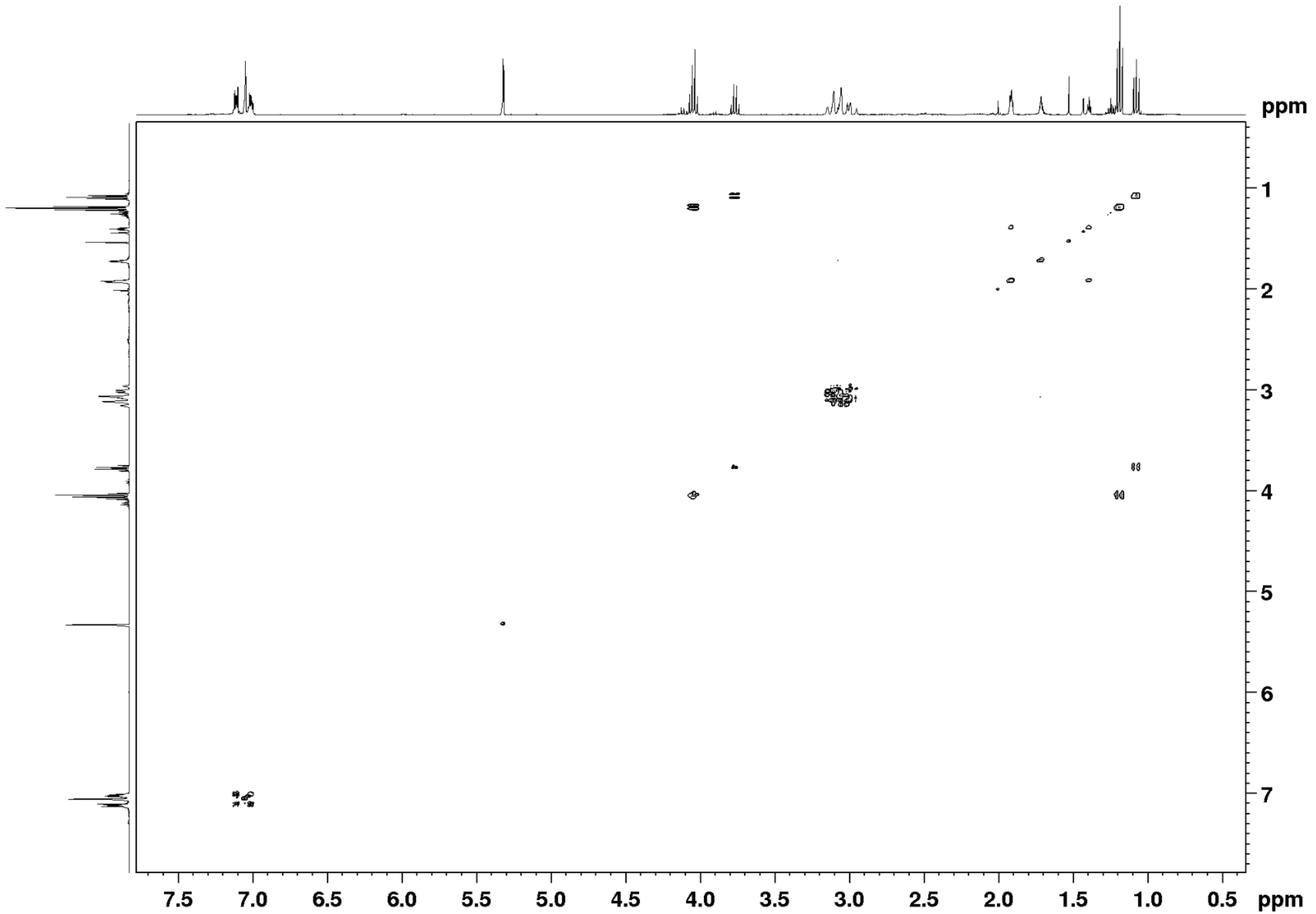

^{1}H, ^{13}C HSQC

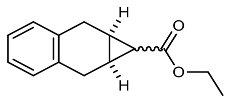

10

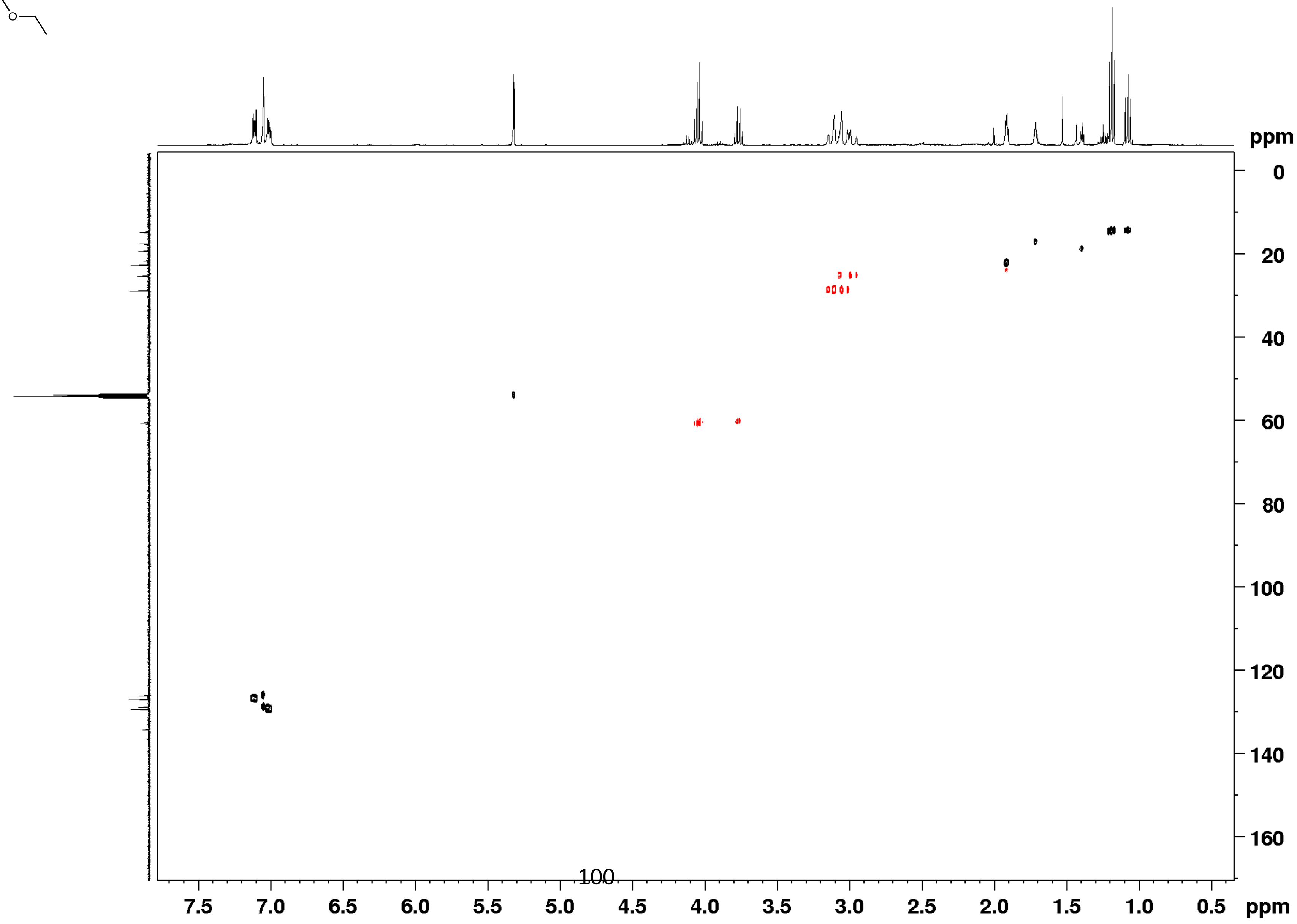

100

^{1}H, ^{13}C HMBC

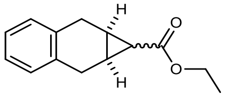

10

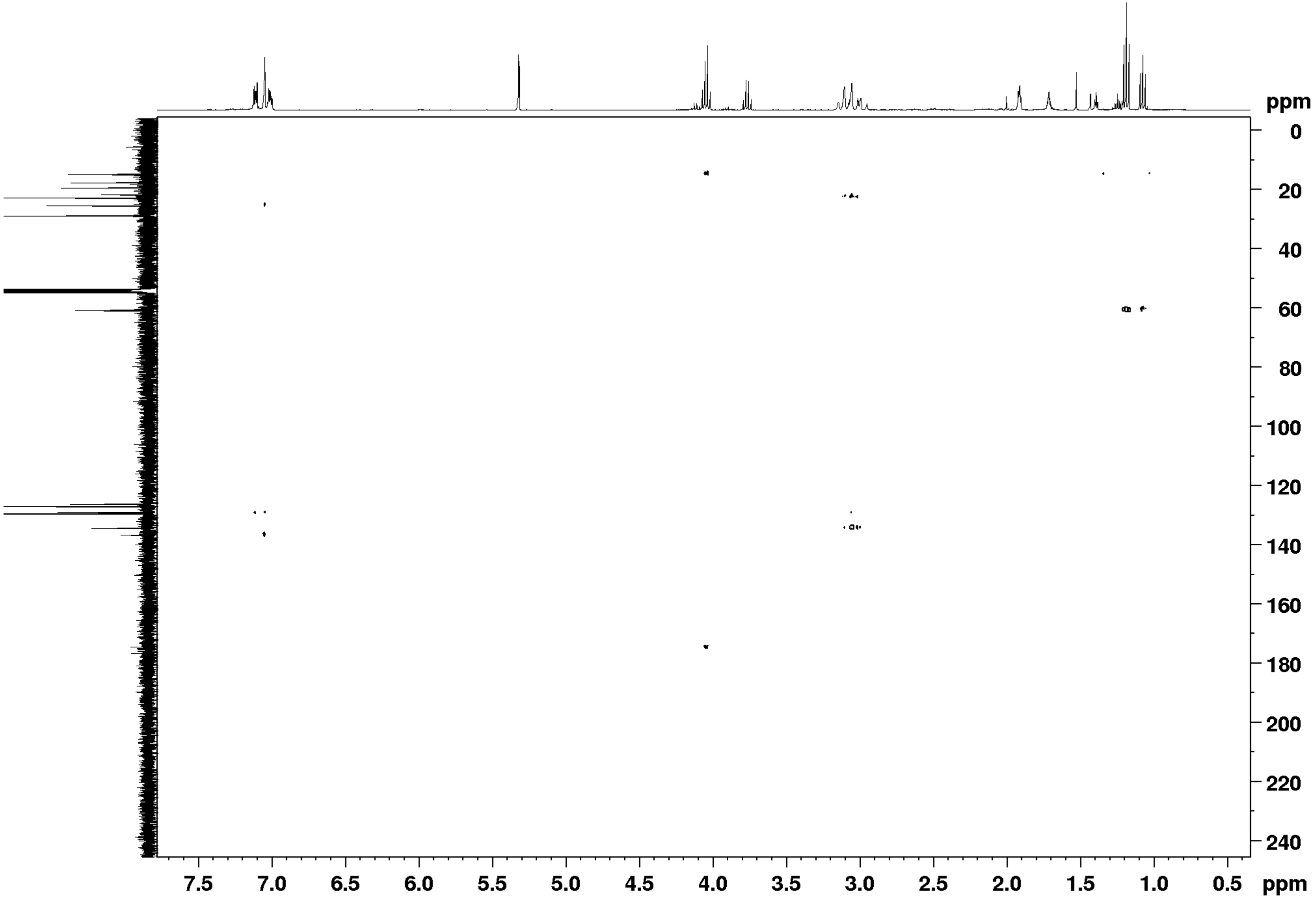

^1H, ^1H NOESY

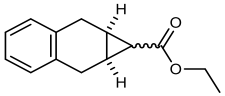

10

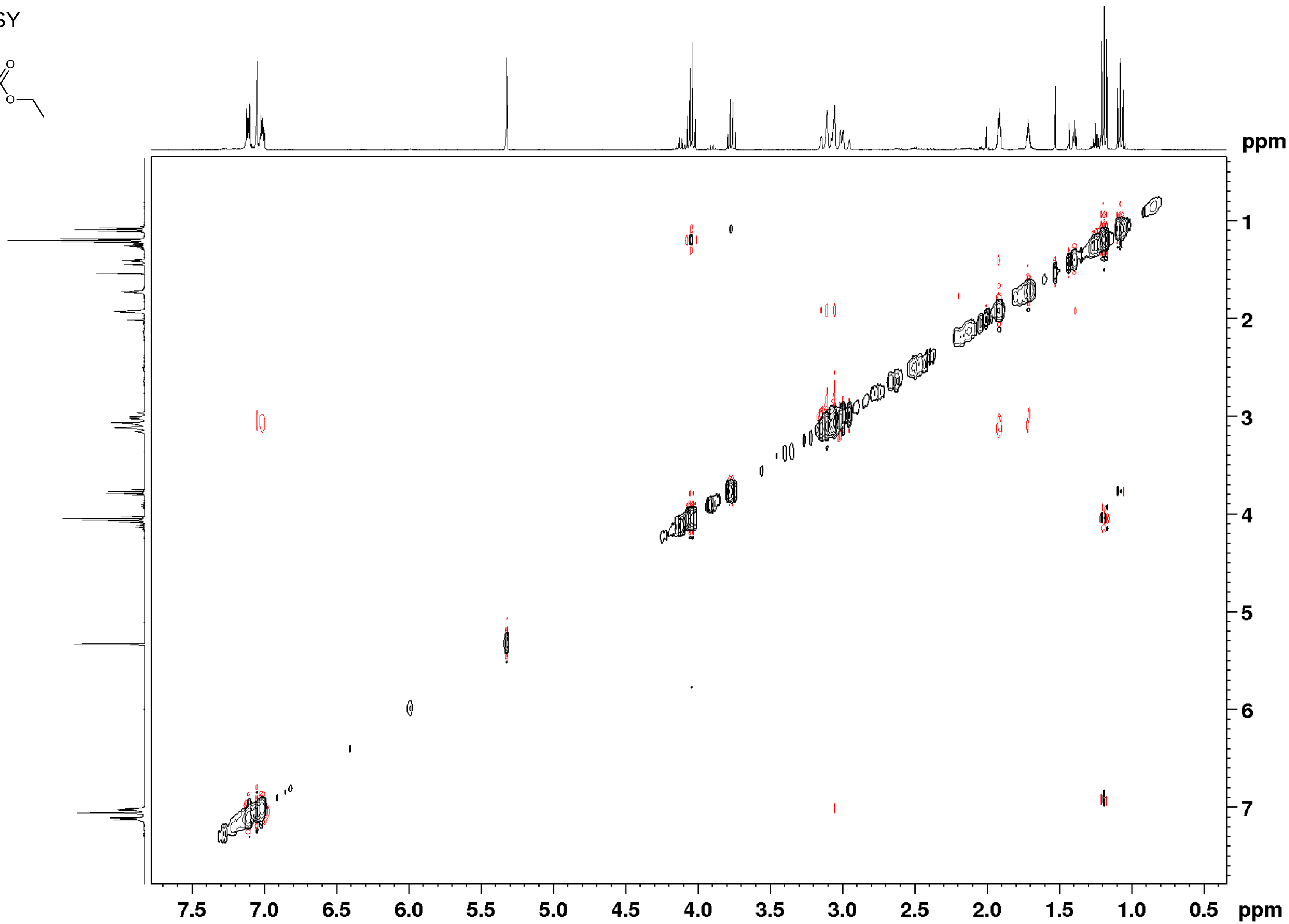

HRMS

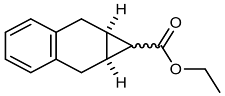

10

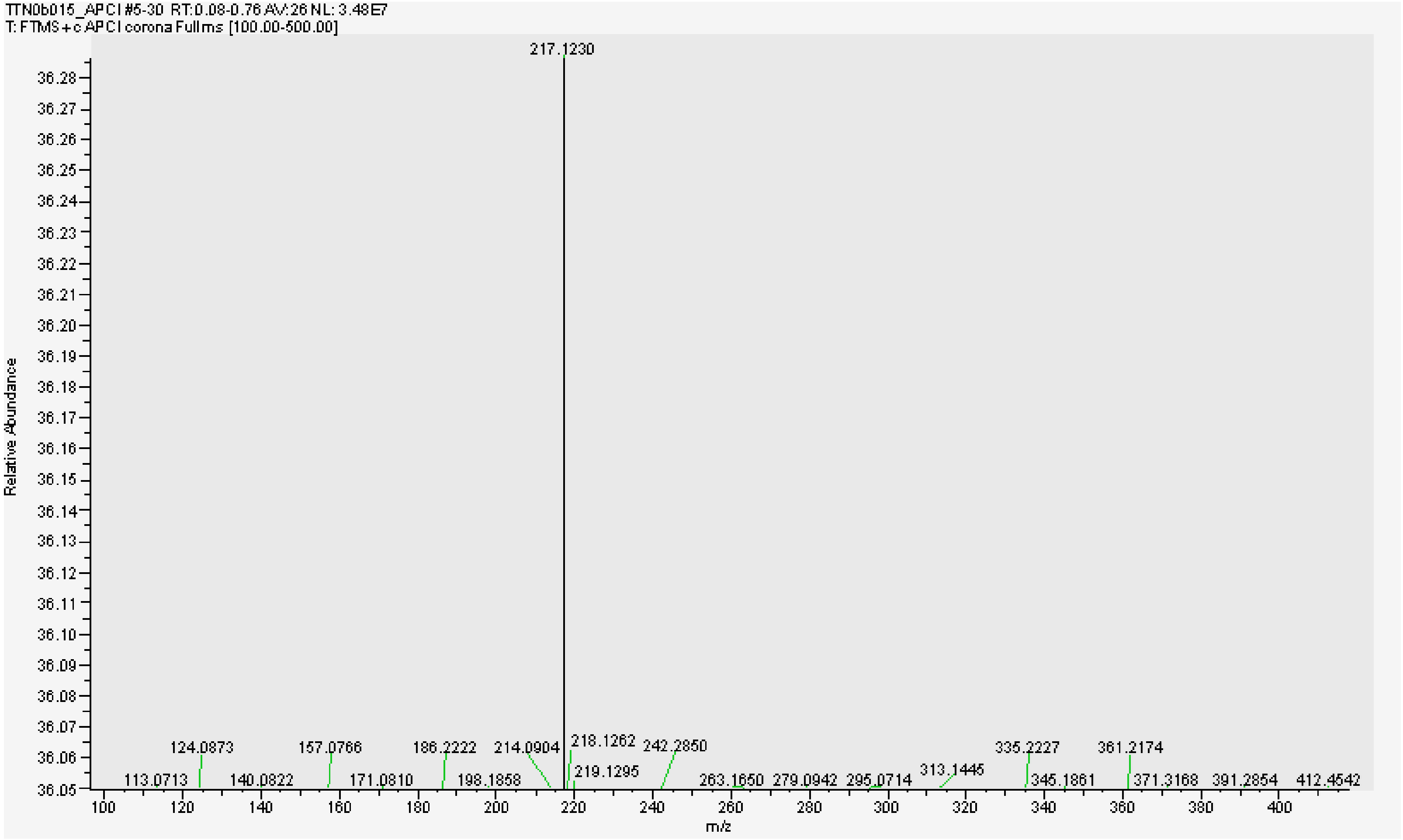
TTN0b015_APCI #5-30 RT:0.08-0.76 AV:26 NL: 3.48E7
T: FTMS + c APCI corona Full ms [100.00-500.00]
217.1230
124.0873
157.0766
186.2222
214.0904
218.1262
242.2850
335.2227
361.2174
113.0713
140.0822
171.0810
198.1858
219.1295
263.1650
279.0942
295.0714
313.1445
345.1861
371.3168
391.2854
412.4542
Relative Abundance
m/z

2.2.3 (1*r*,1a*R*,7a*S*)-1a,2,7,7a-Tetrahydro-1*H*-cyclopropa[b]naphthalene-1-carboxylic acid (*trans*-11)

^{1}H NMR

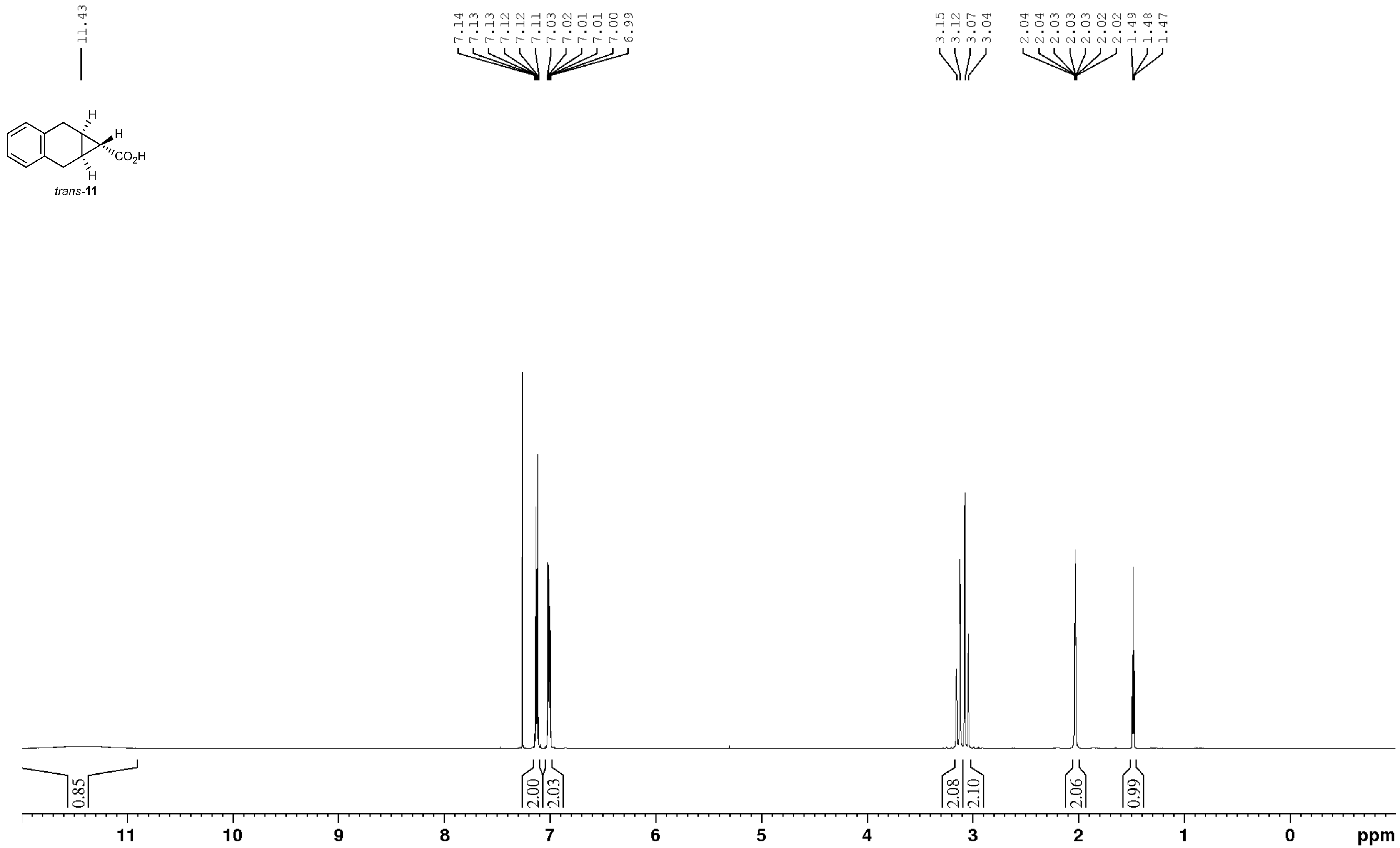

^{13}C NMR

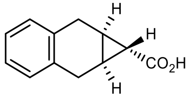

trans-**11**

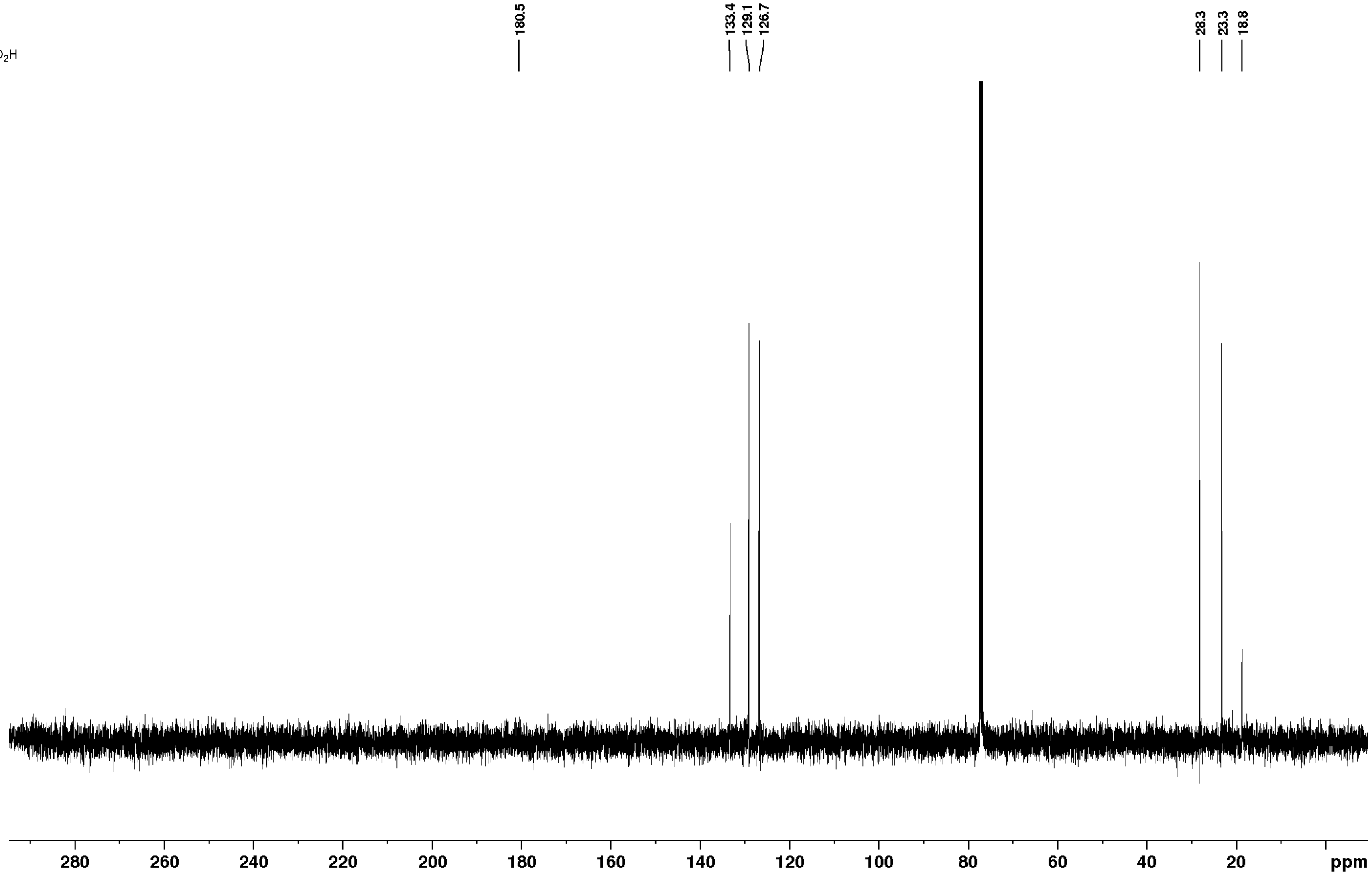

^{1}H, ^{1}H COSY

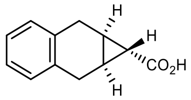

trans-**11**

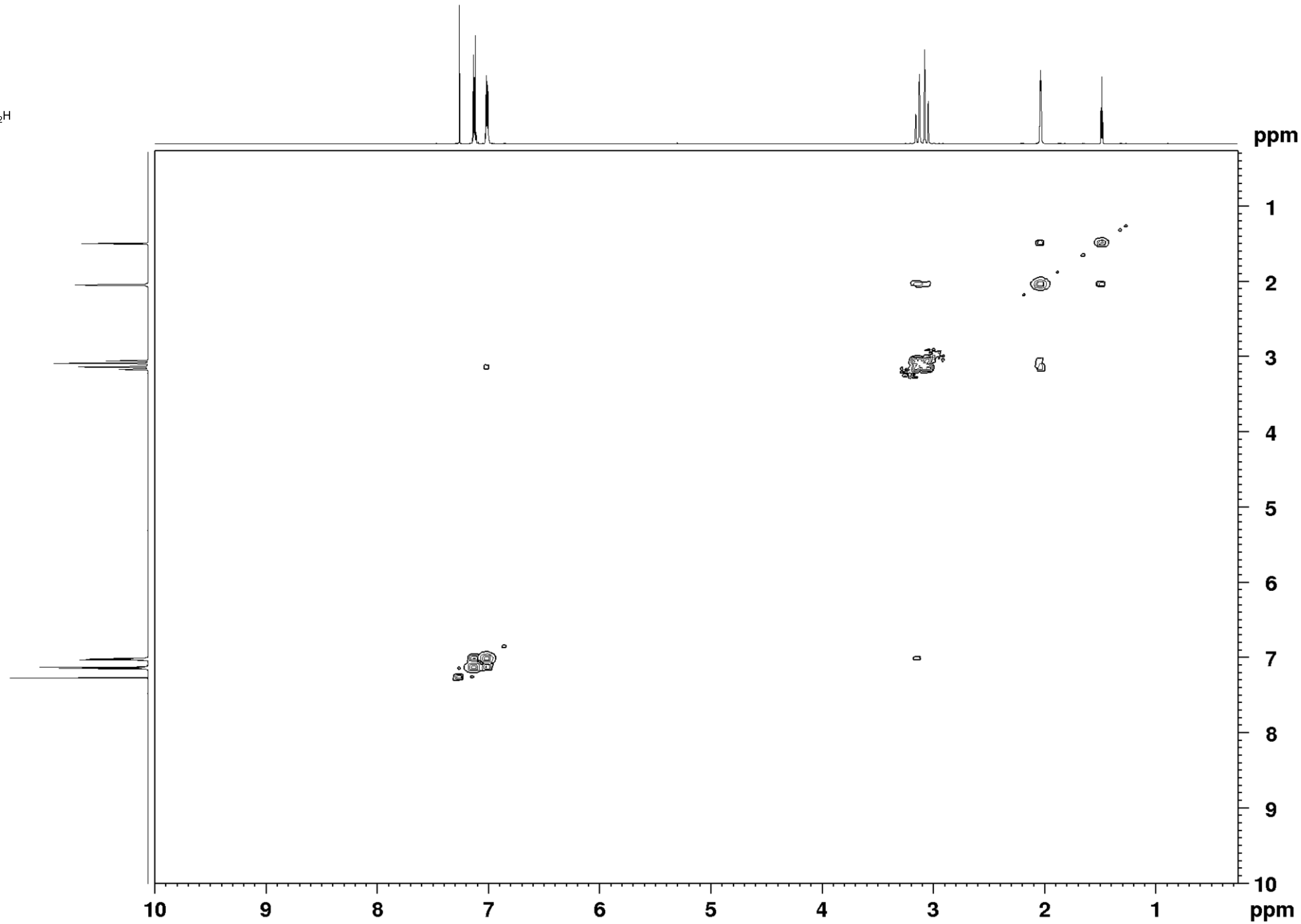

^{1}H, ^{13}C HSQC

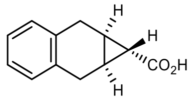
trans-**11**

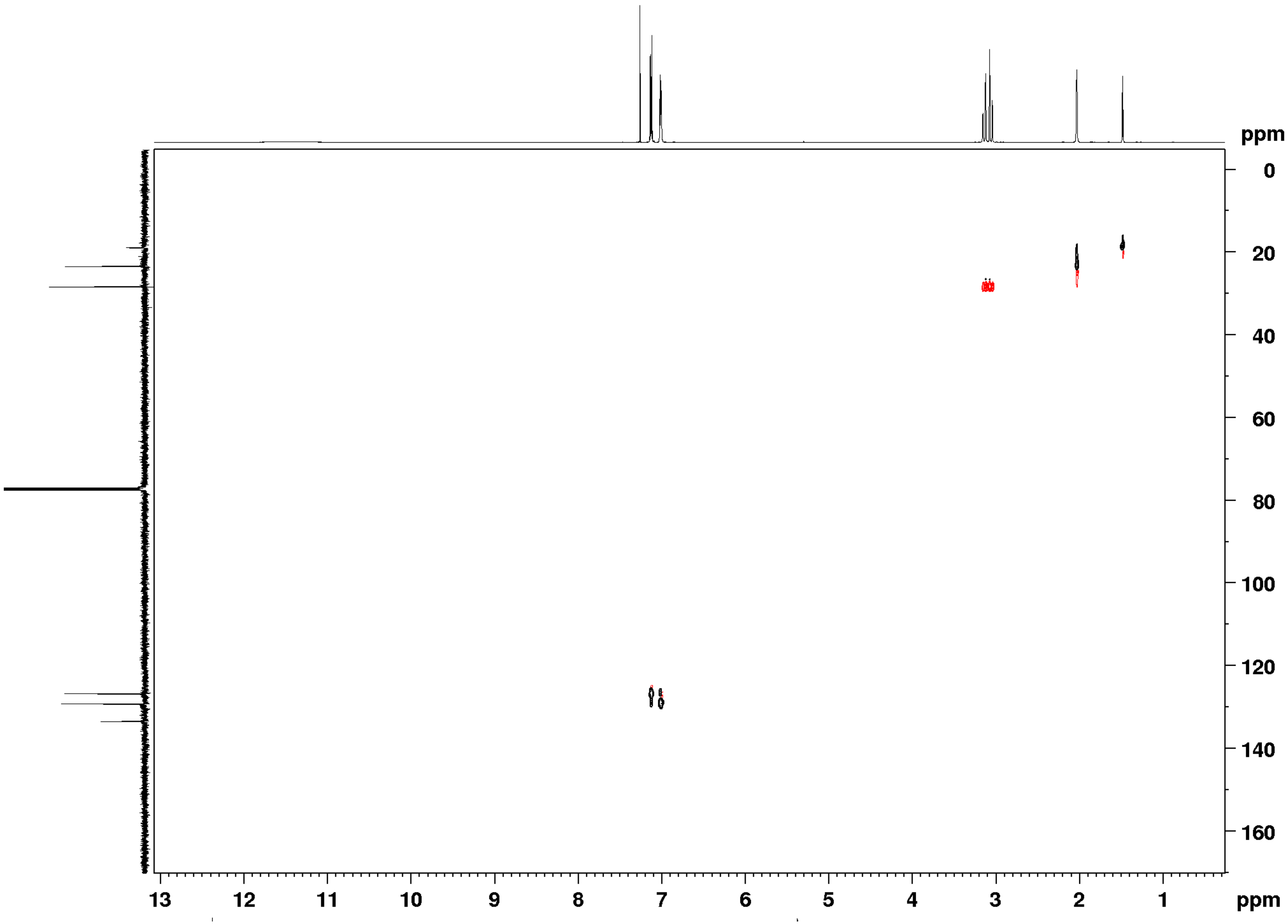

^{1}H, ^{13}C HMBC

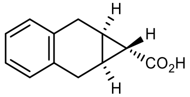

trans-**11**

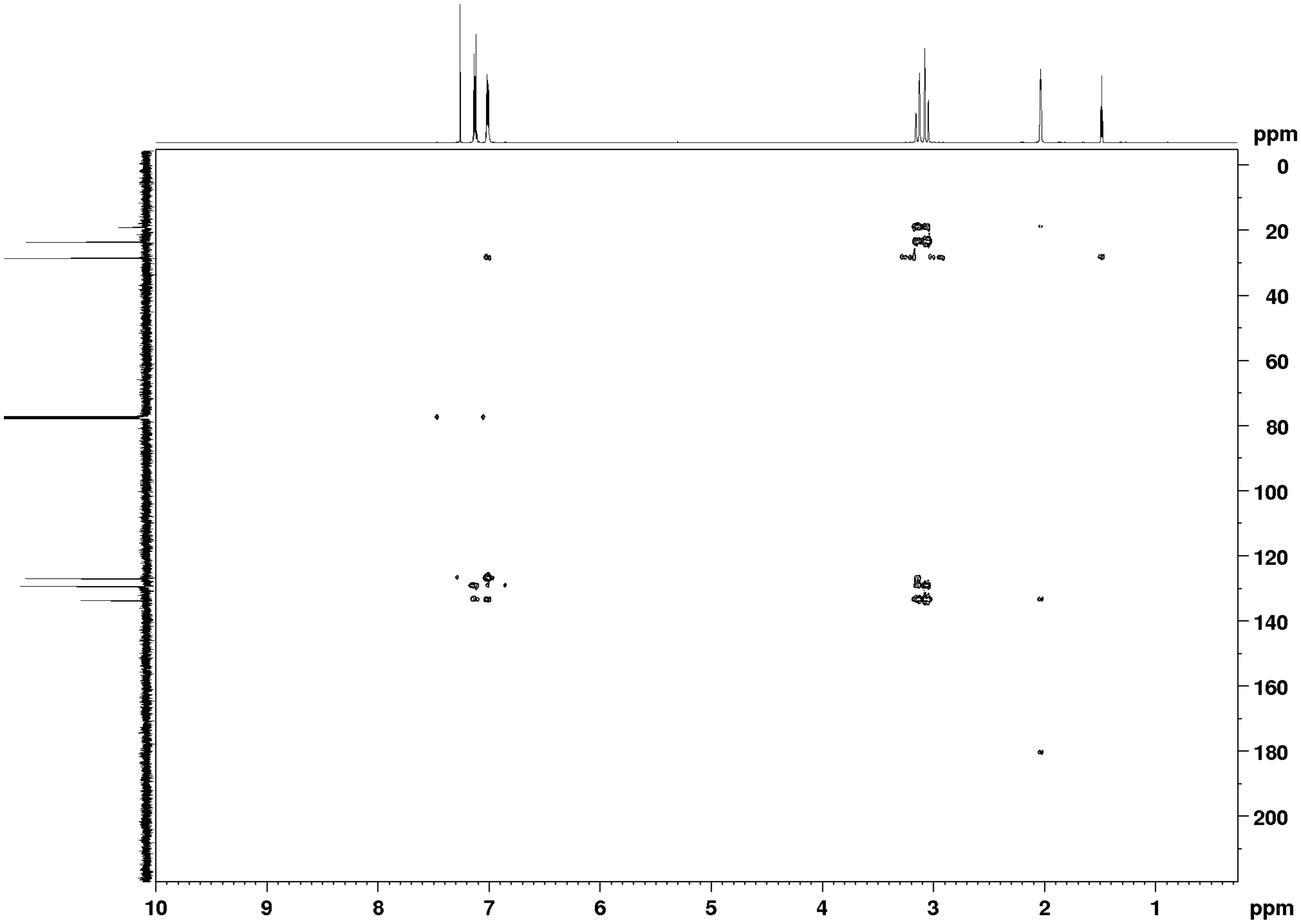

^1H, ^1H NOESY

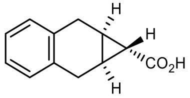

trans-**11**

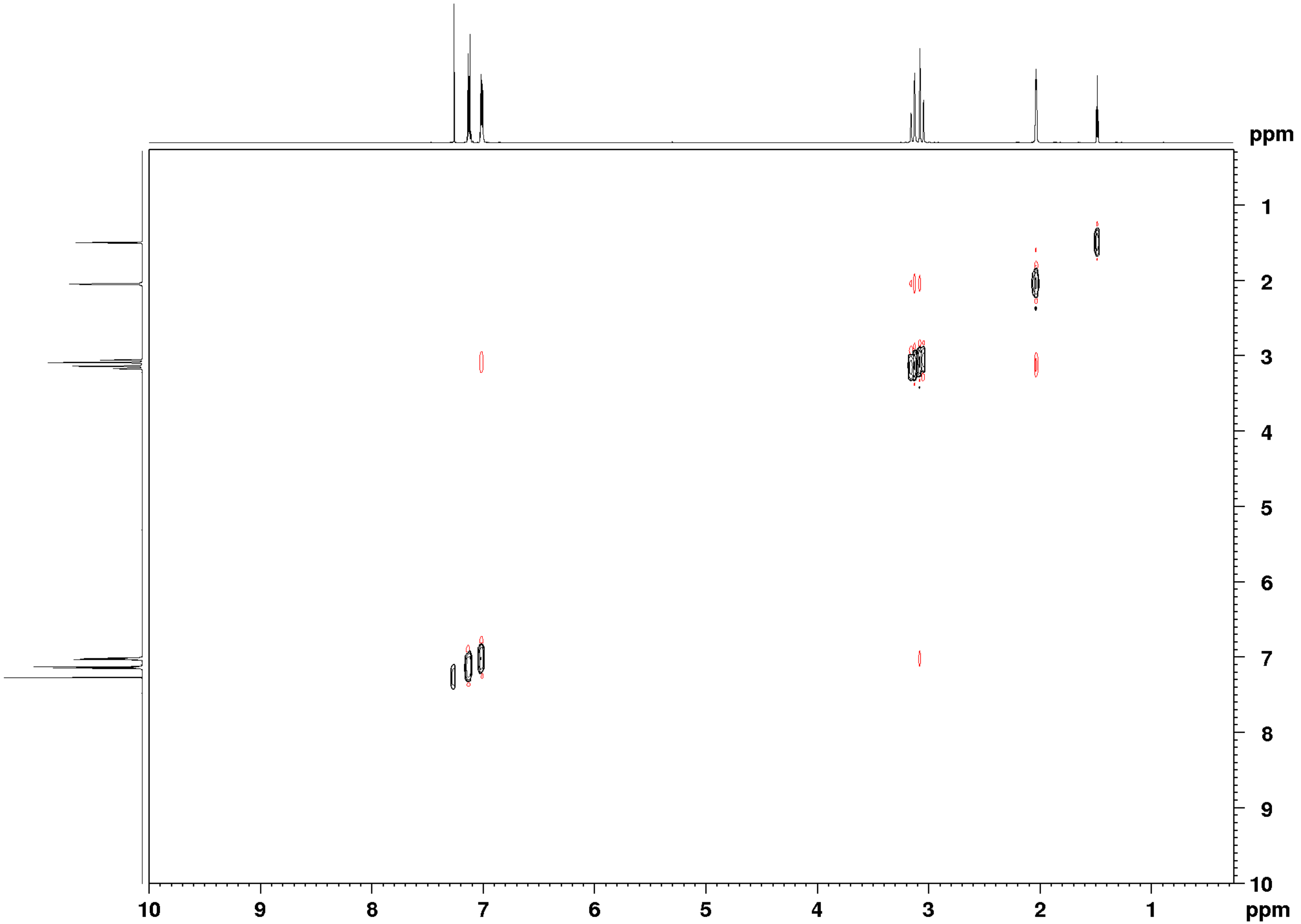

HRMS

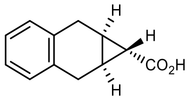

trans-**11**

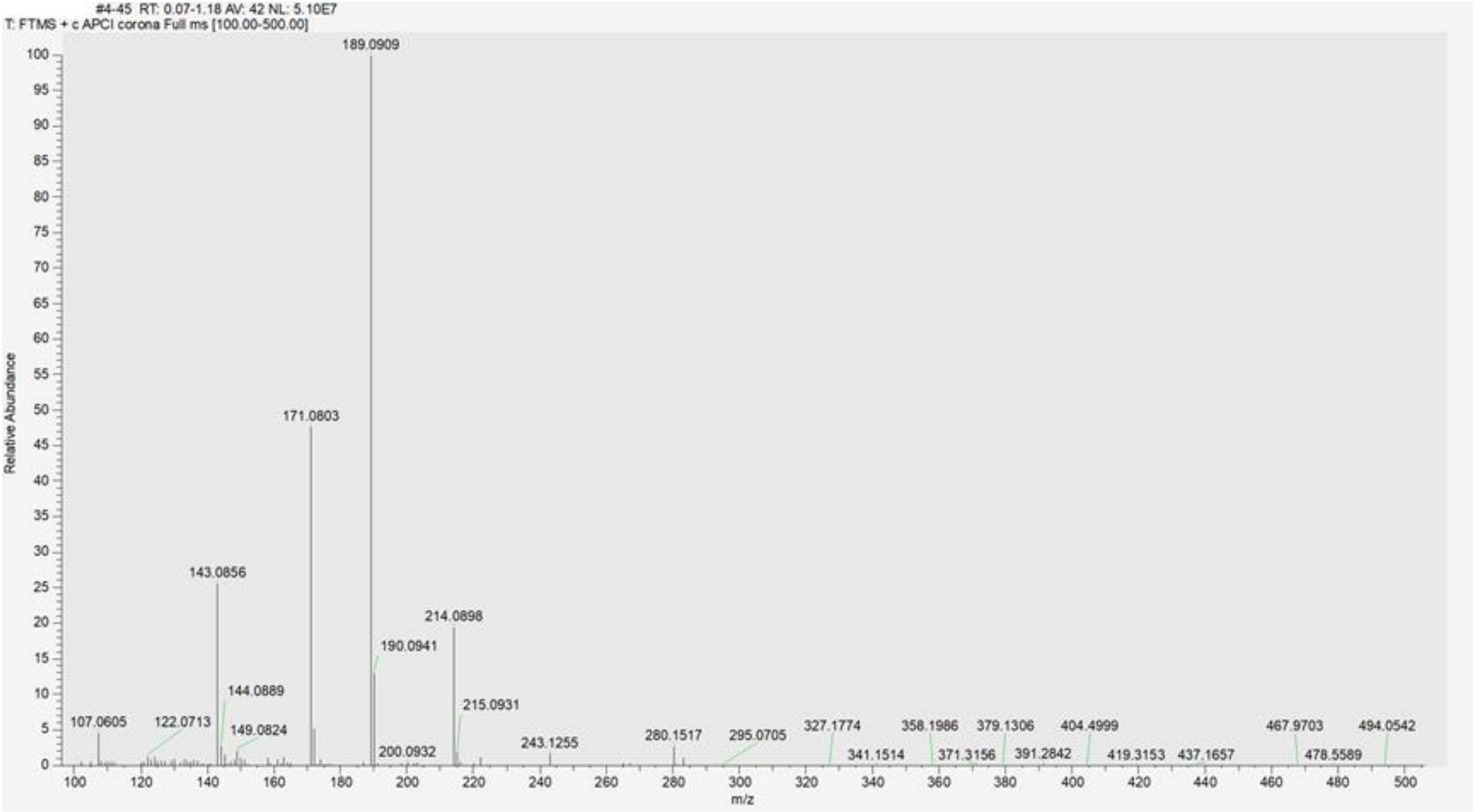

IR spectra

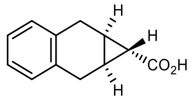

trans-**11**

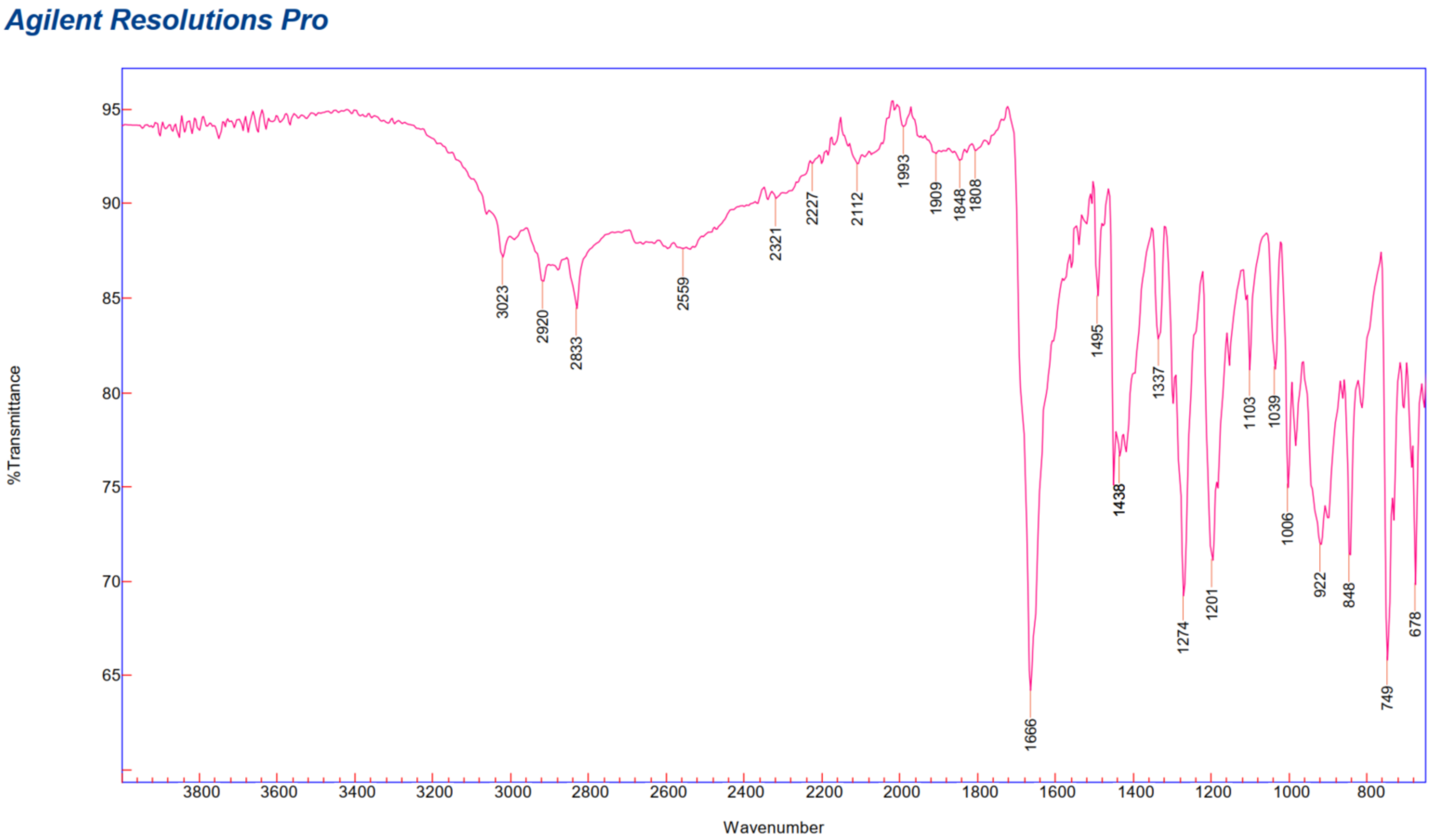

2.2.4 Ethyl (1*s*,1a*R*,7a*S*)-1a,2,7,7a-tetrahydro-1*H*-cyclopropa[b]naphthalene-1-carboxylate (*cis*-10)

^{1}H NMR

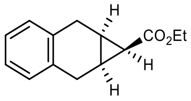

cis-**10**

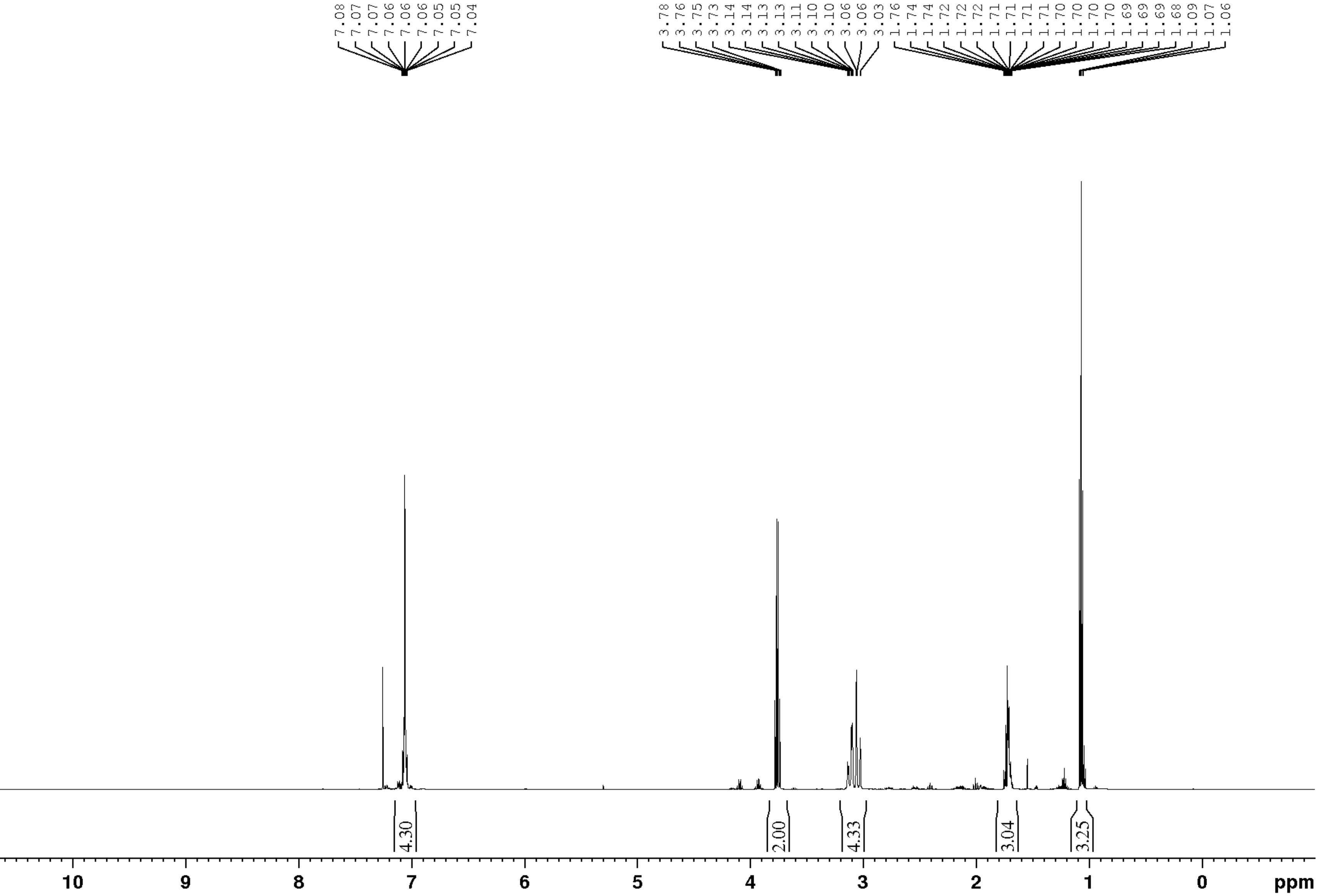

^{13}C NMR

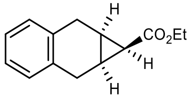

cis-**10**

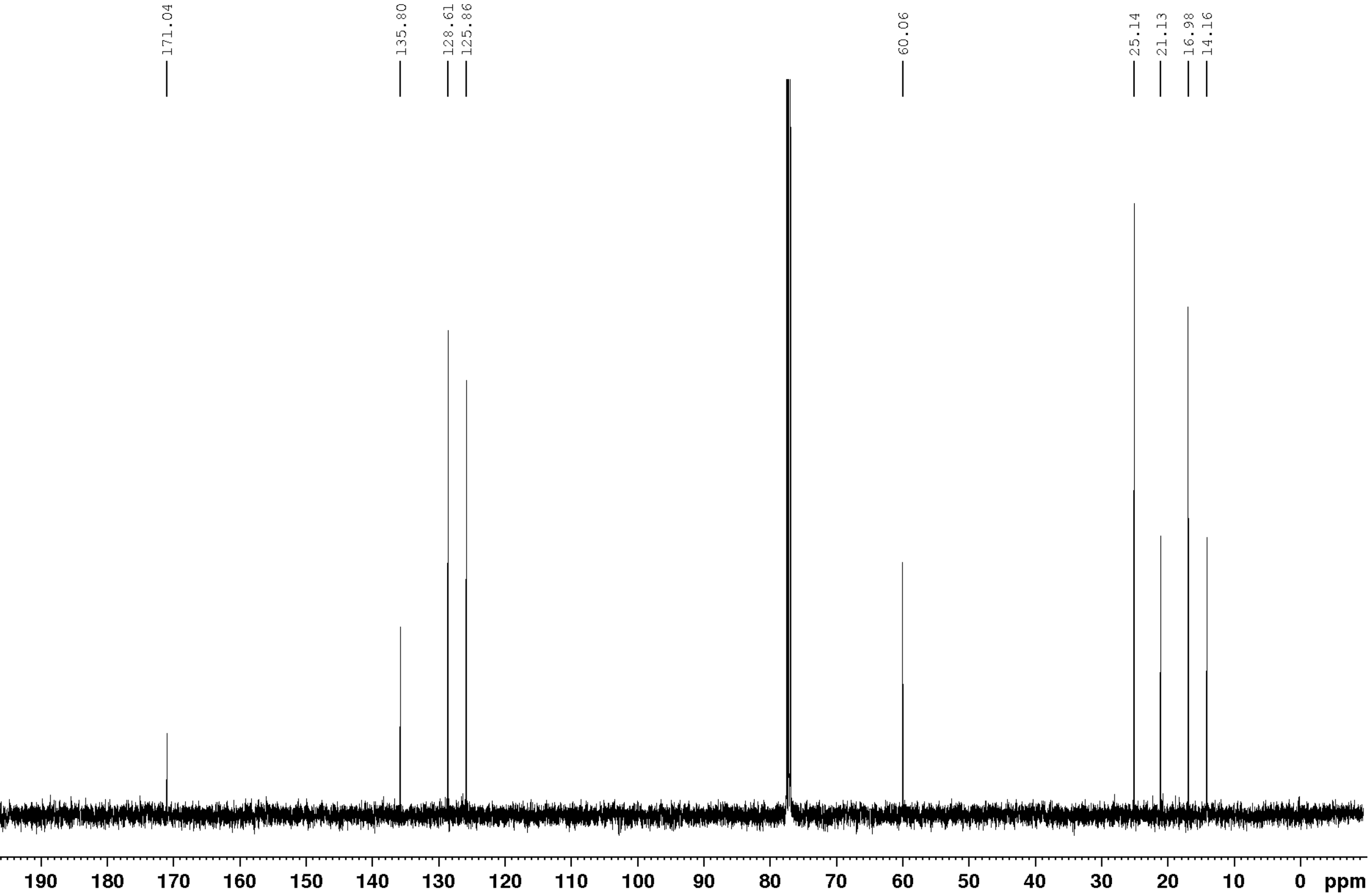

^1H, ^1H COSY

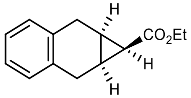

cis-**10**

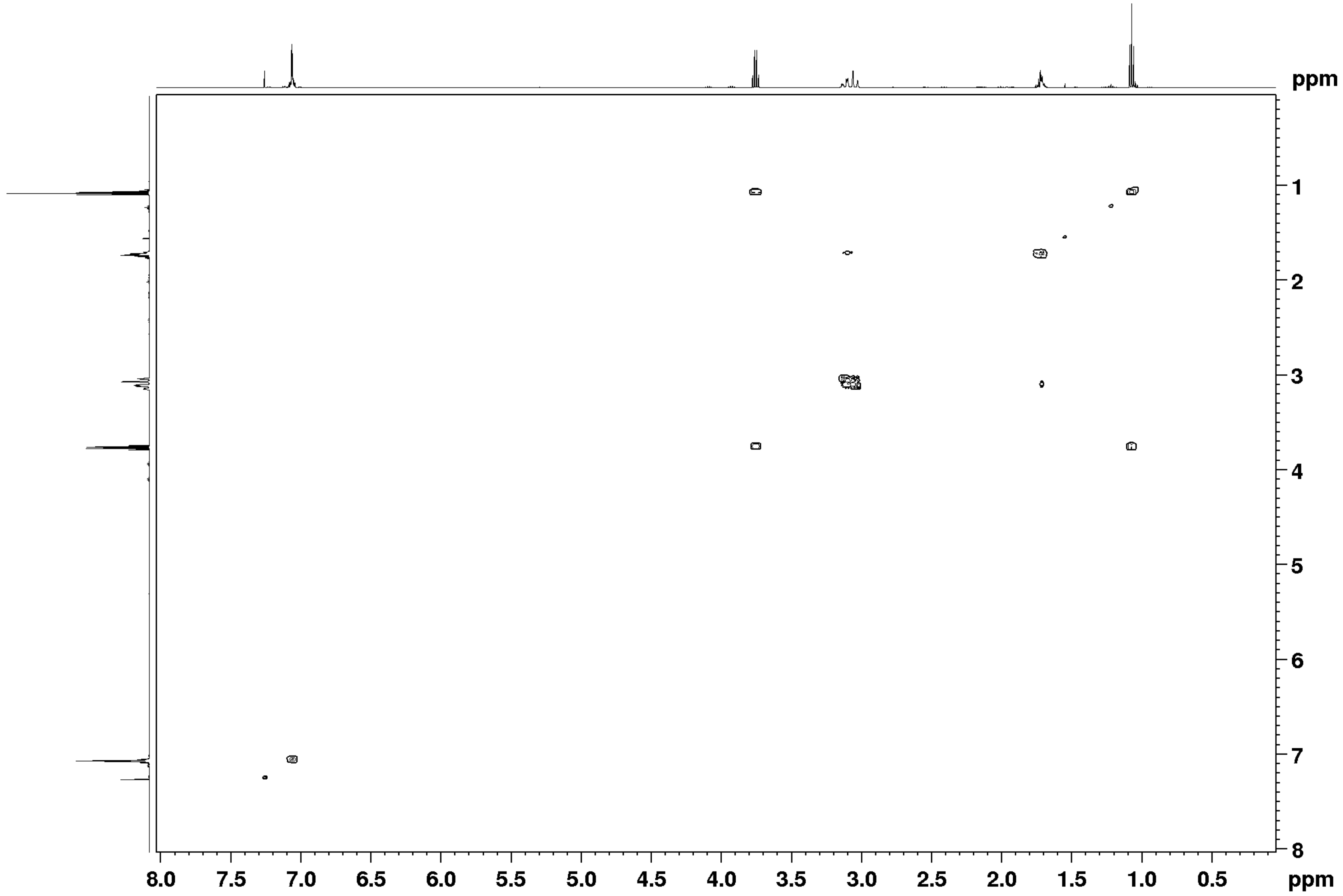

^{1}H, ^{13}C HSQC

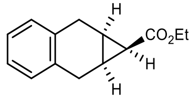

cis-**10**

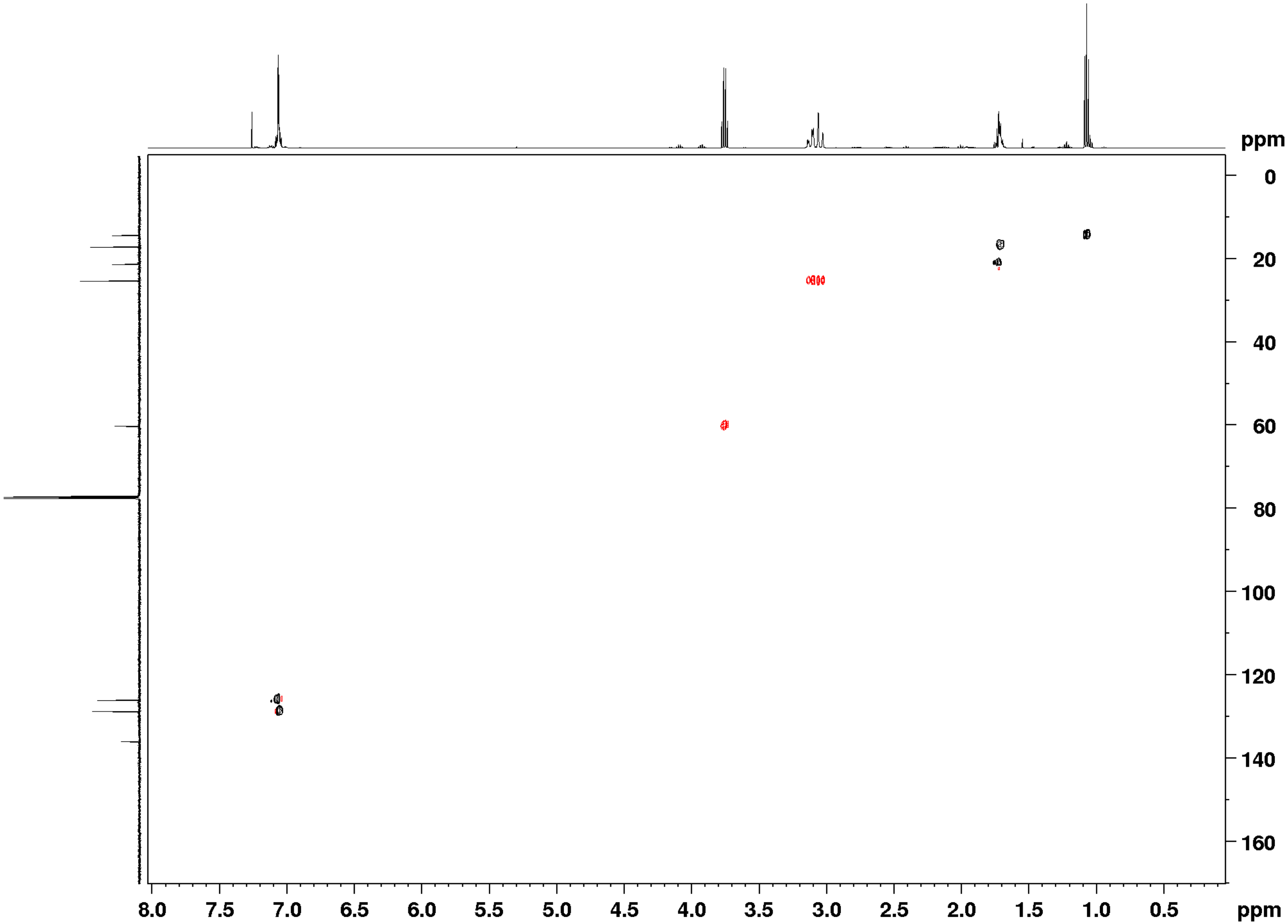

^1H, ^{13}C HMBC

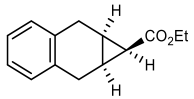

cis-**10**

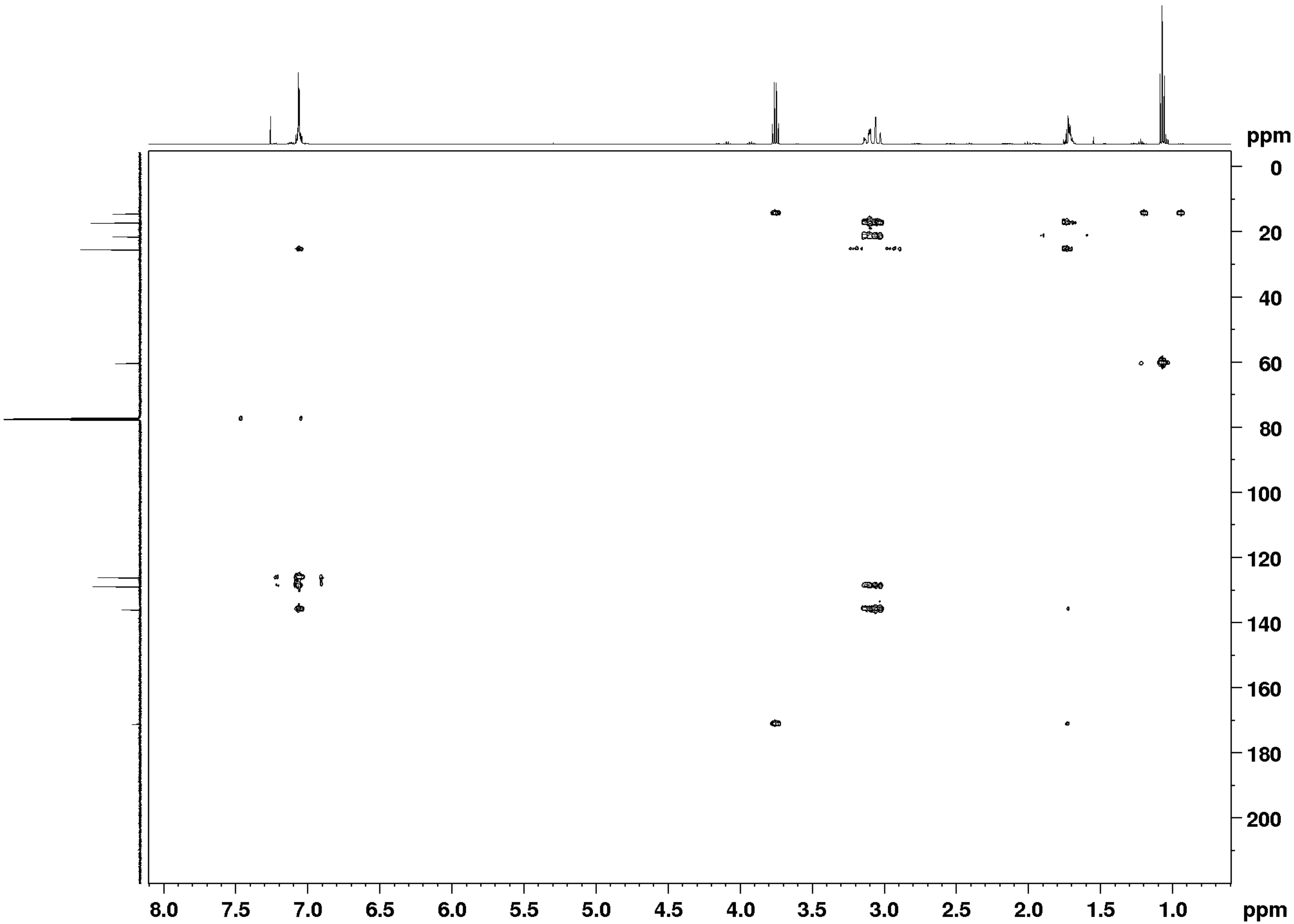

^1H, ^1H NOESY

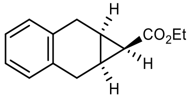

cis-**10**

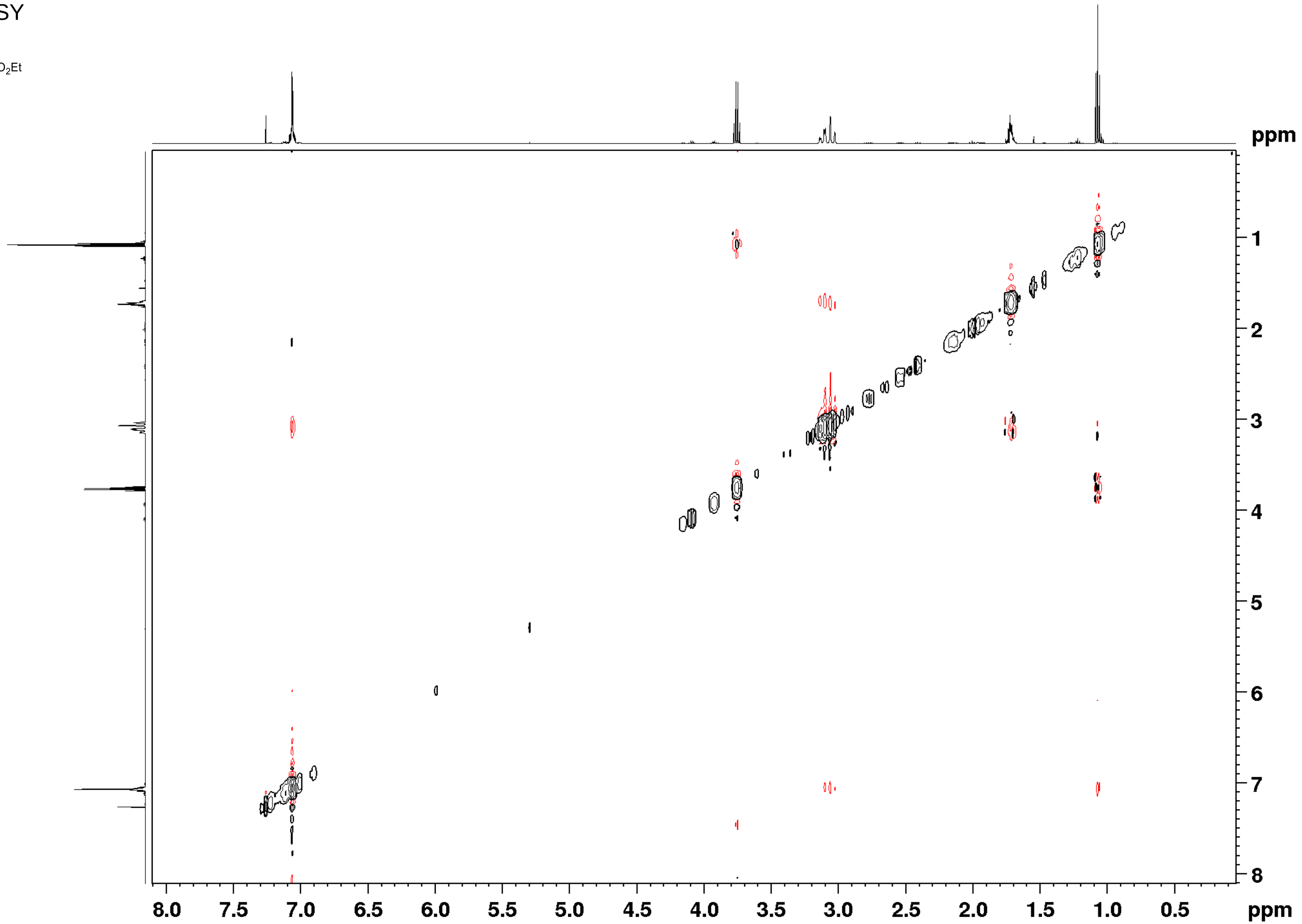

HRMS

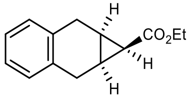

H
CO2Et
H
H

cis-**10**

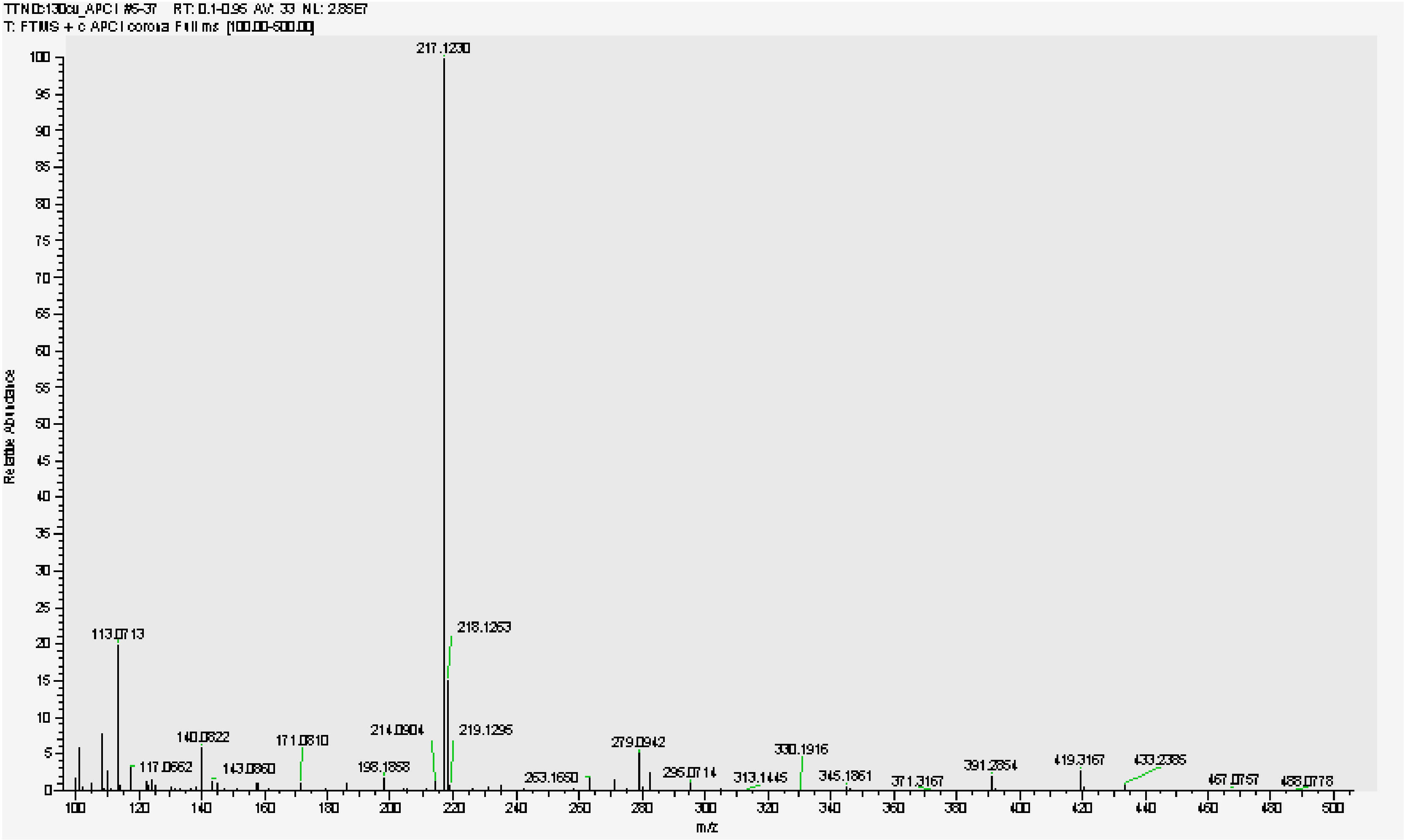

TTN0:130cu_APCI #5-37 RT: 0.1-0.96 AV: 33 NL: 2.85E7
T: FTMS + c APCI corona Full ms [100.00-500.00]
217.1230
218.1263
219.1295
113.0713
140.0822
171.0810
214.0904
279.0942
330.1916
117.0662
143.0860
198.1858
263.1650
295.0714
313.1445
345.1861
371.3167
391.2854
419.3167
433.2385
467.0757
488.0718
Relative Abundance
m/z

IR spectra

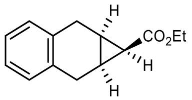

cis-**10**

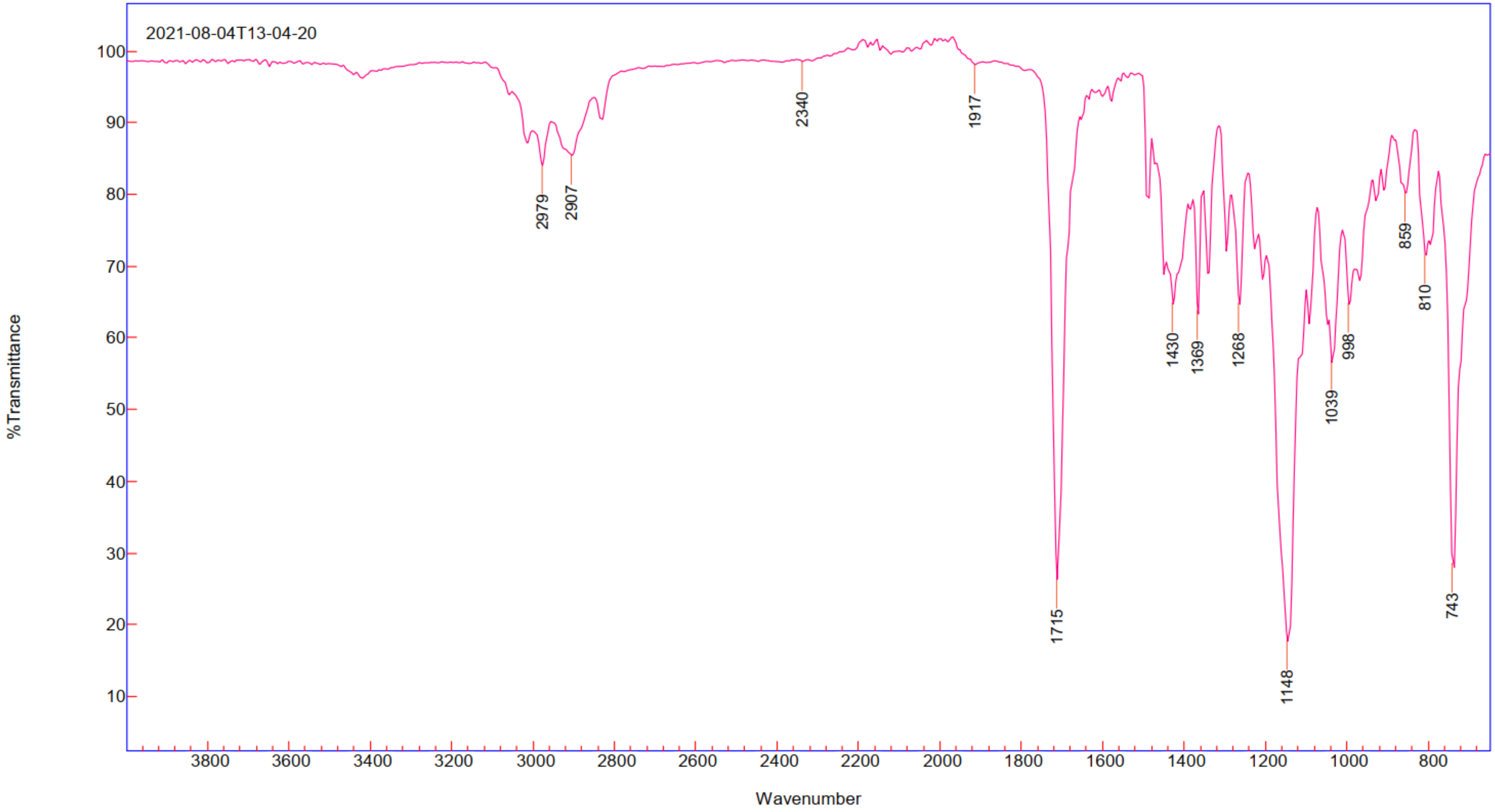

2.2.5 (1*s*,1a*R*,7a*S*)-1a,2,7,7a-tetrahydro-1*H*-cyclopropa[b]naphthalene-1-carboxylic acid (*cis*-11)

^{1}H NMR

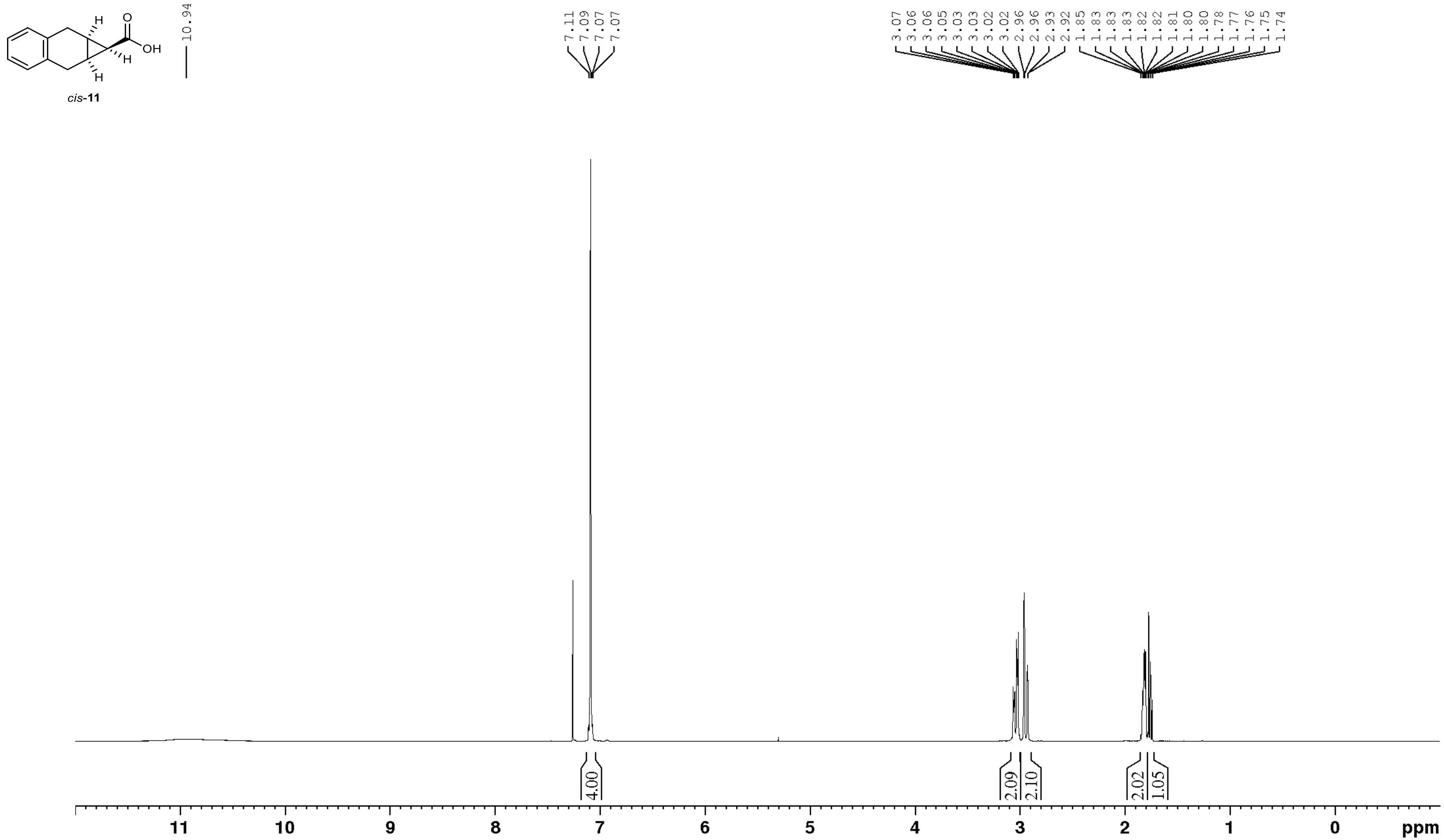

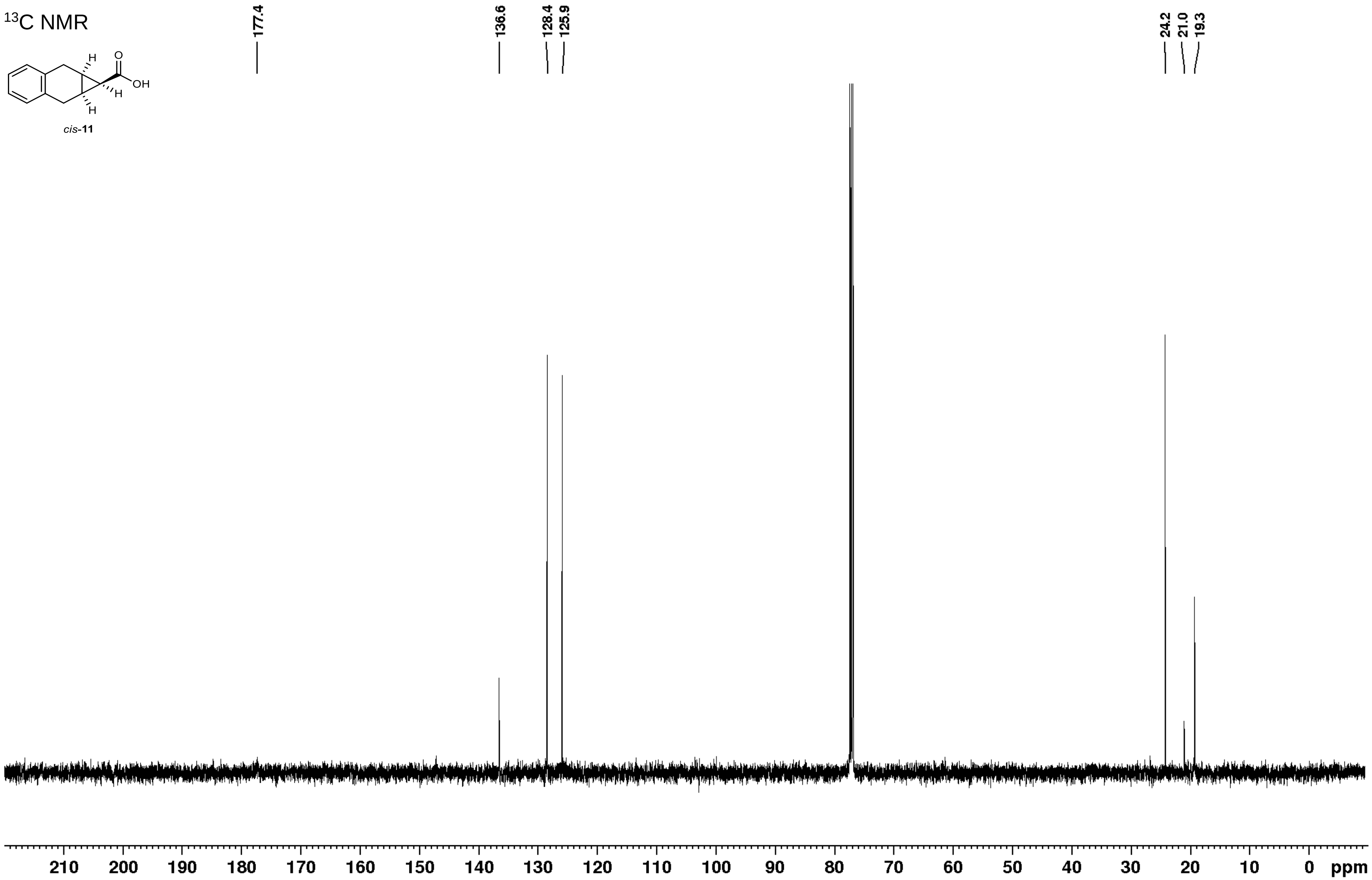

^{13}C NMR
cis-11
O
OH
H
177.4
136.6
128.4
125.9
24.2
21.0
19.3
210 200 190 180 170 160 150 140 130 120 110 100 90 80 70 60 50 40 30 20 10 0 ppm

^{1}H, ^{1}H COSY

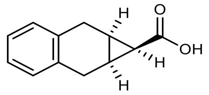

cis-**11**

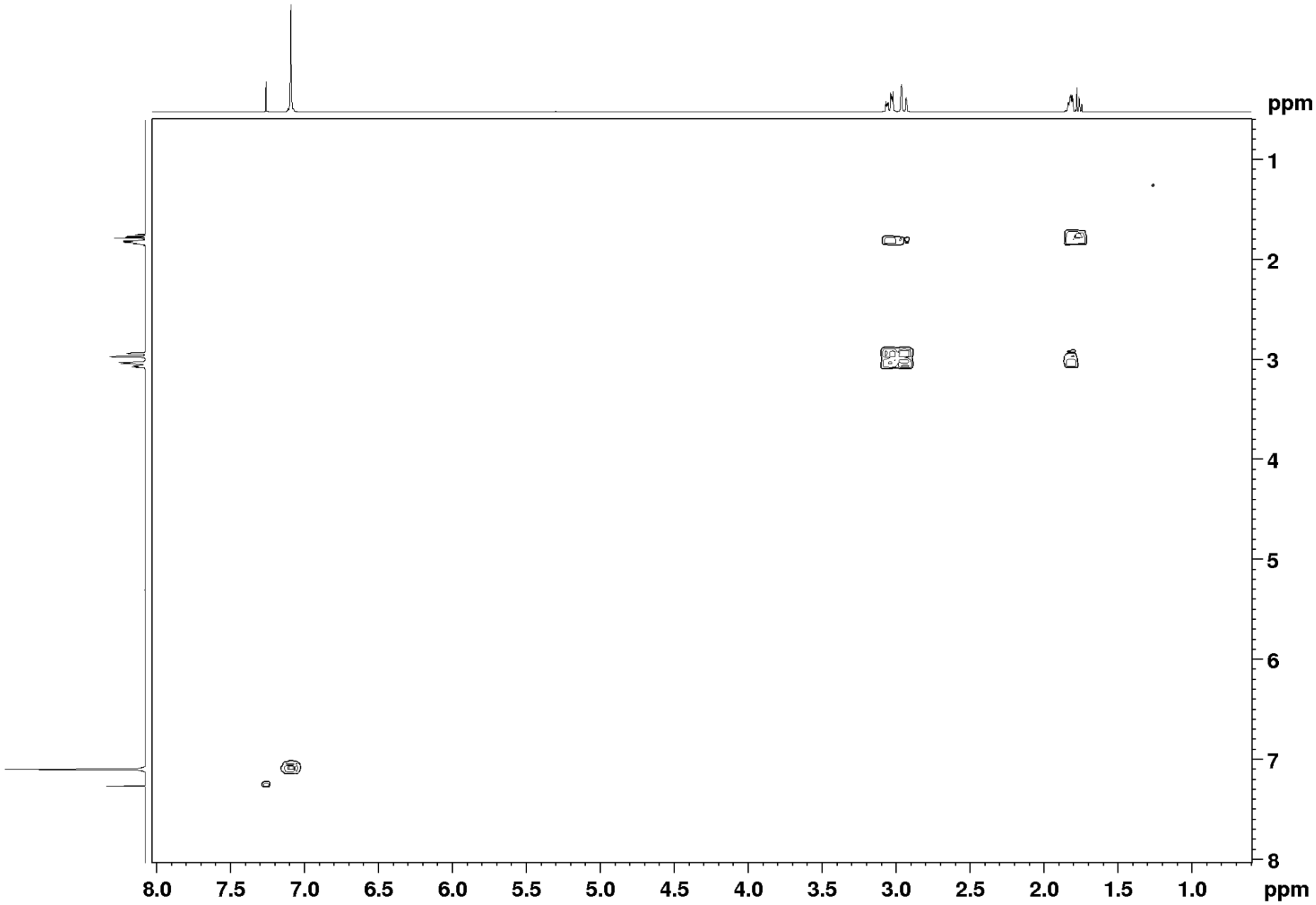

^{1}H, ^{13}C HSQC

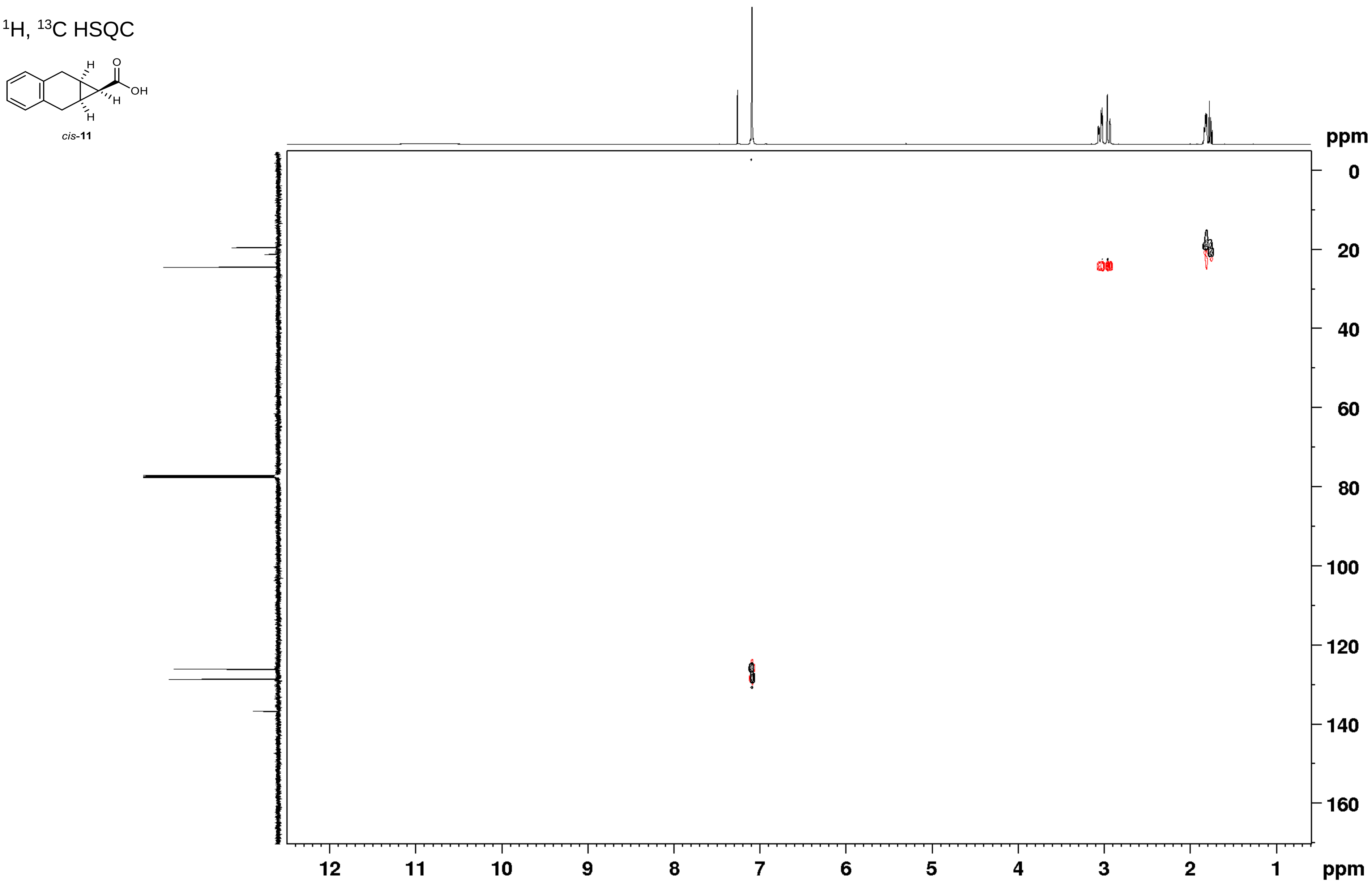
H
O
OH
H
H
cis-11
ppm
0
20
40
60
80
100
120
140
160
12
11
10
9
8
7
6
5
4
3
2
1
ppm

^{1}H, ^{13}C HMBC

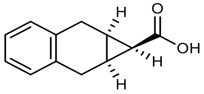

cis-**11**

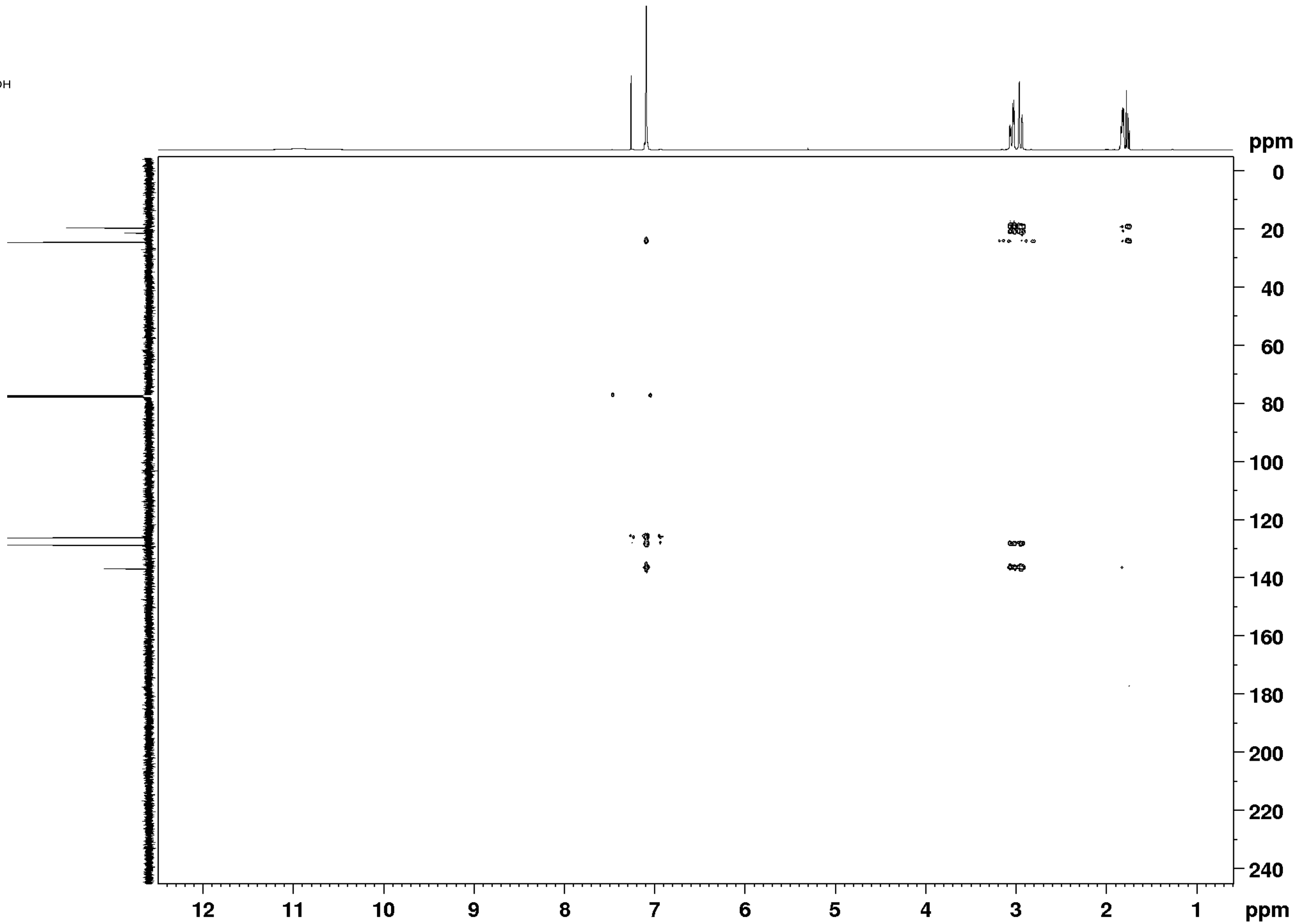

$^{1}H, ^{1}H$ NOESY

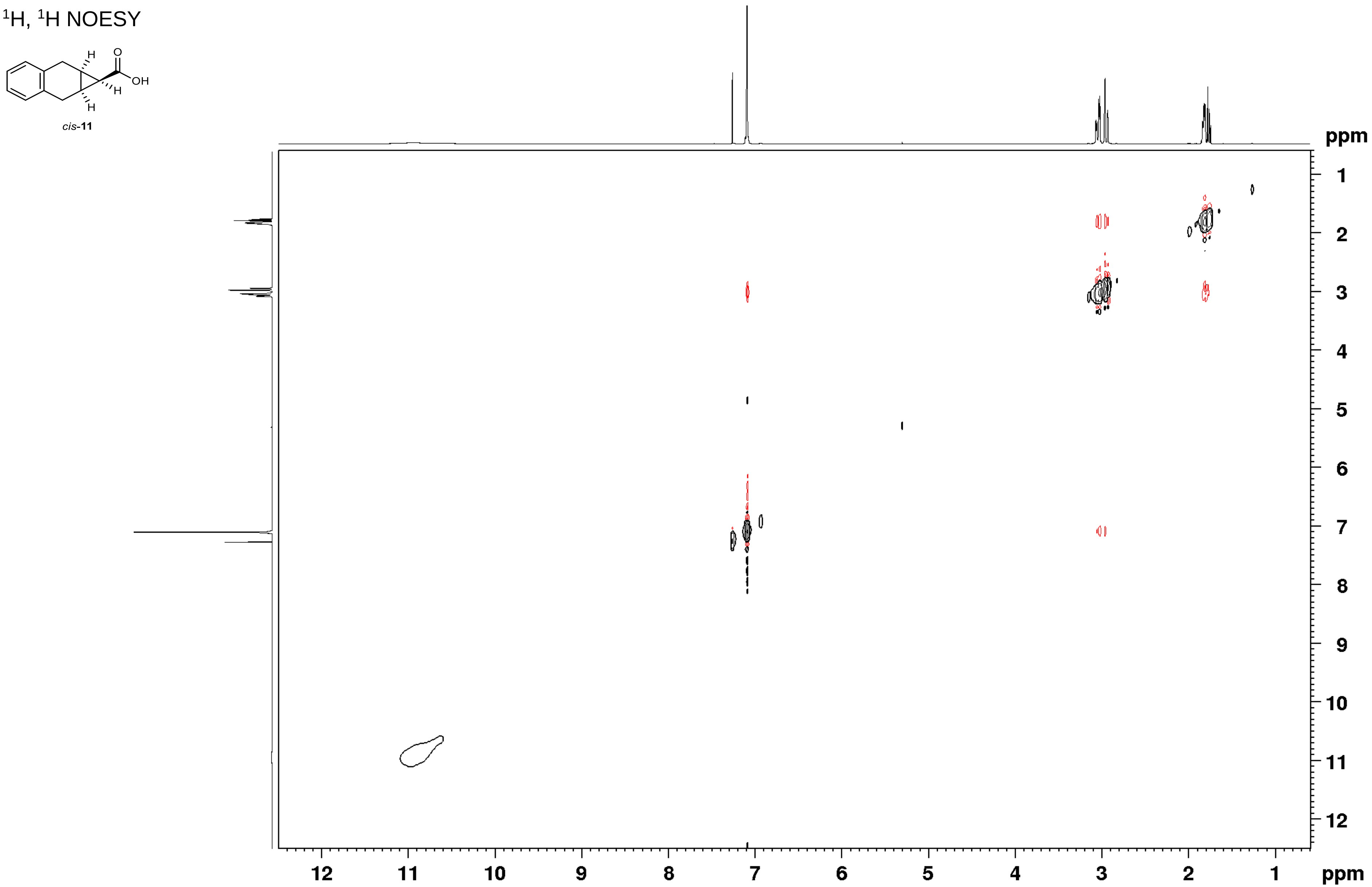

cis-**11**

HRMS

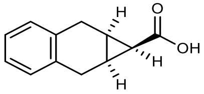

cis-**11**

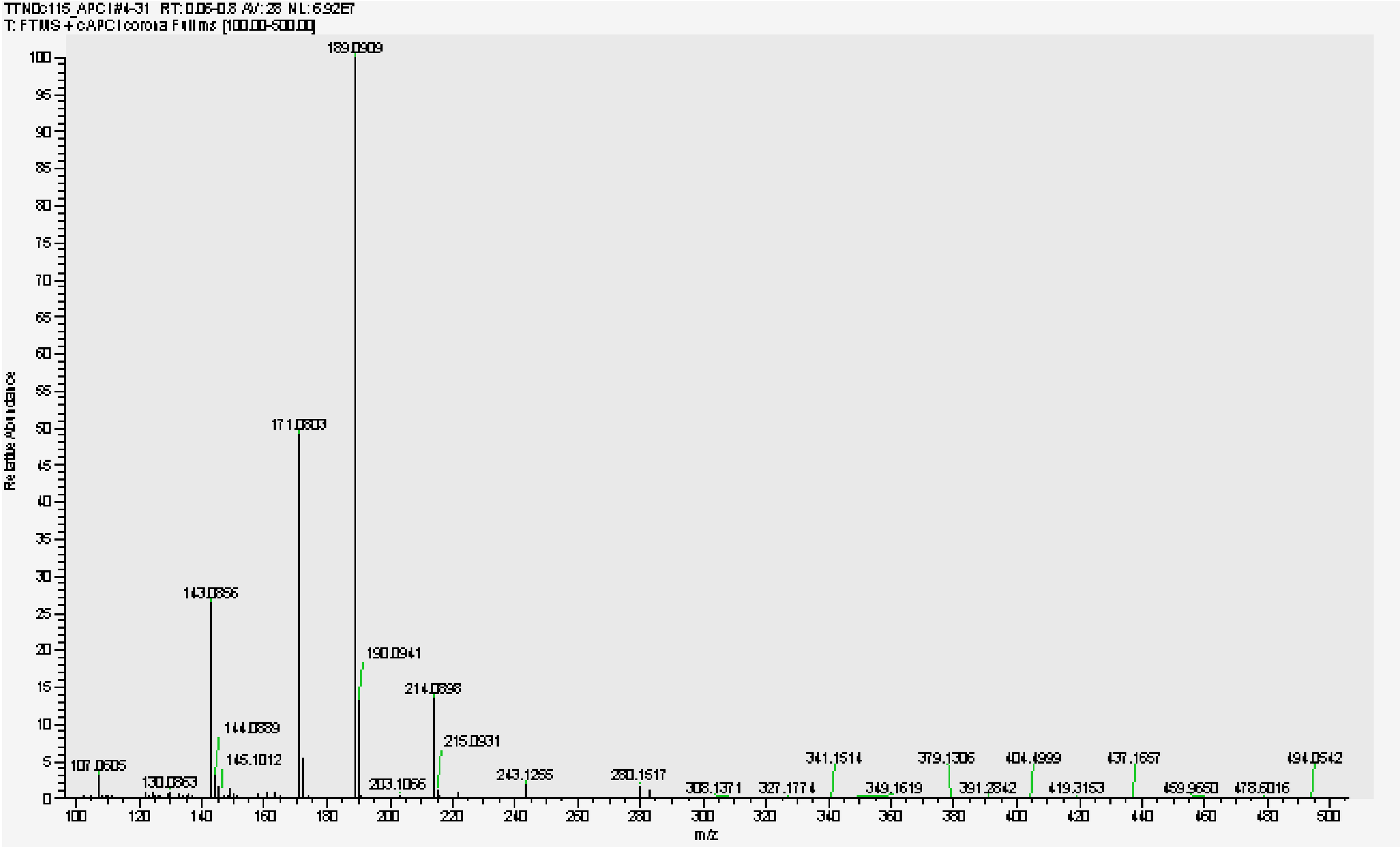

TTNDc115_APCI#4-31 RT: 0.06-0.8 AV: 28 NL: 6.92E7
T: FTMS + c APCI corona Full ms [100.00-500.00]
189.0909
171.0803
143.0856
190.0941
214.0898
144.0889
215.0931
145.1012
107.0605
130.0863
203.1066
243.1255
280.1517
308.1371
327.1774
341.1514
349.1619
379.1306
391.2842
404.4999
419.3153
437.1657
459.9650
478.6016
494.0542
Relative Abundance
m/z

IR spectra

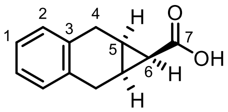

XX

$C_{12}H_{12}O_2$

M = 188.23 g/mol

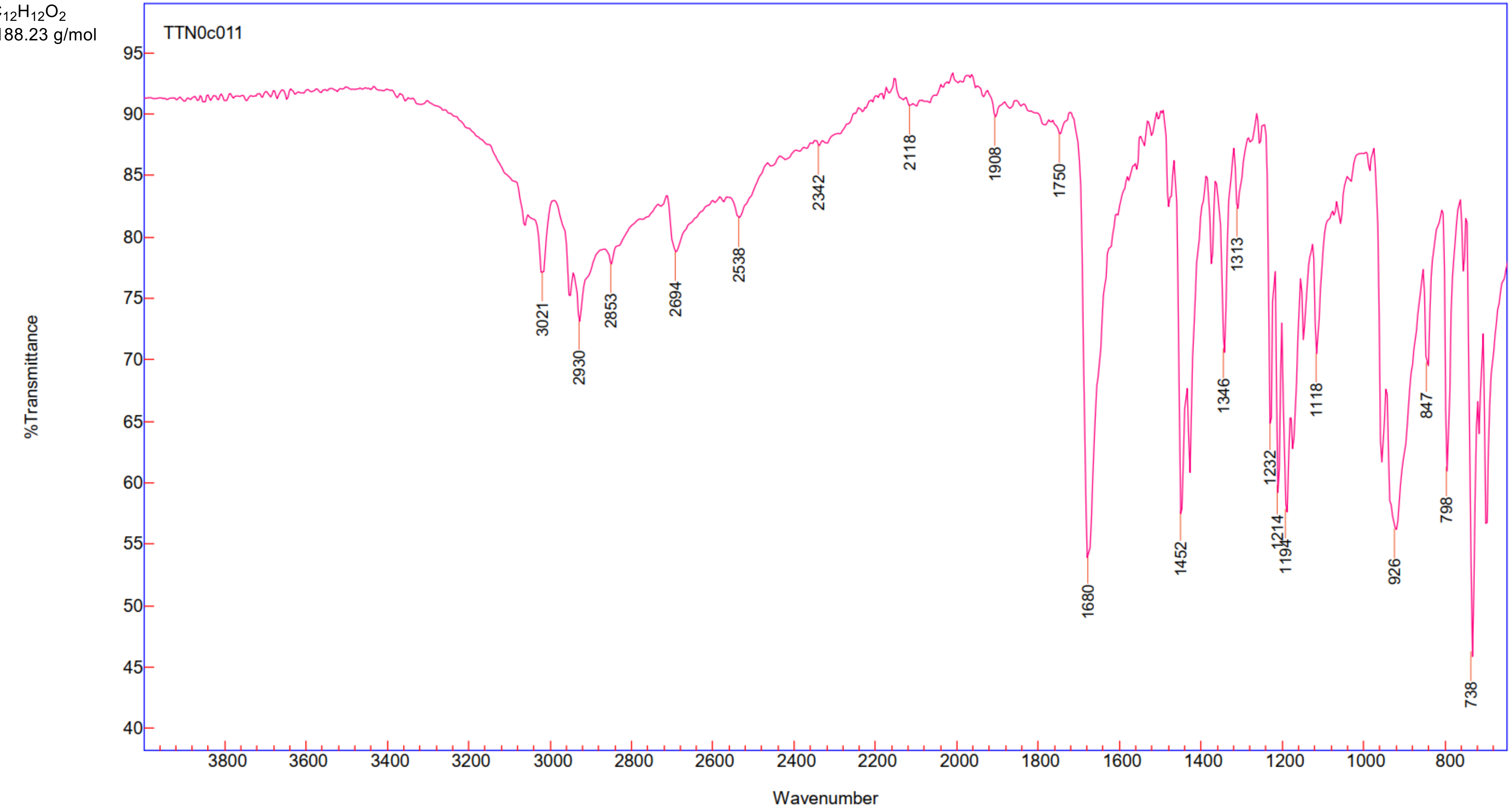

2.2.6 Methyl 3-oxo-3-((1*s*,1a*R*,7a*S*)-1a,2,7,7a-tetrahydro-1*H*-cyclopropa[b]naphthalen-1-yl)propanoate (12a)

^{1}H NMR

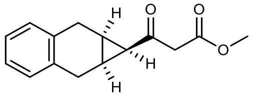

12a

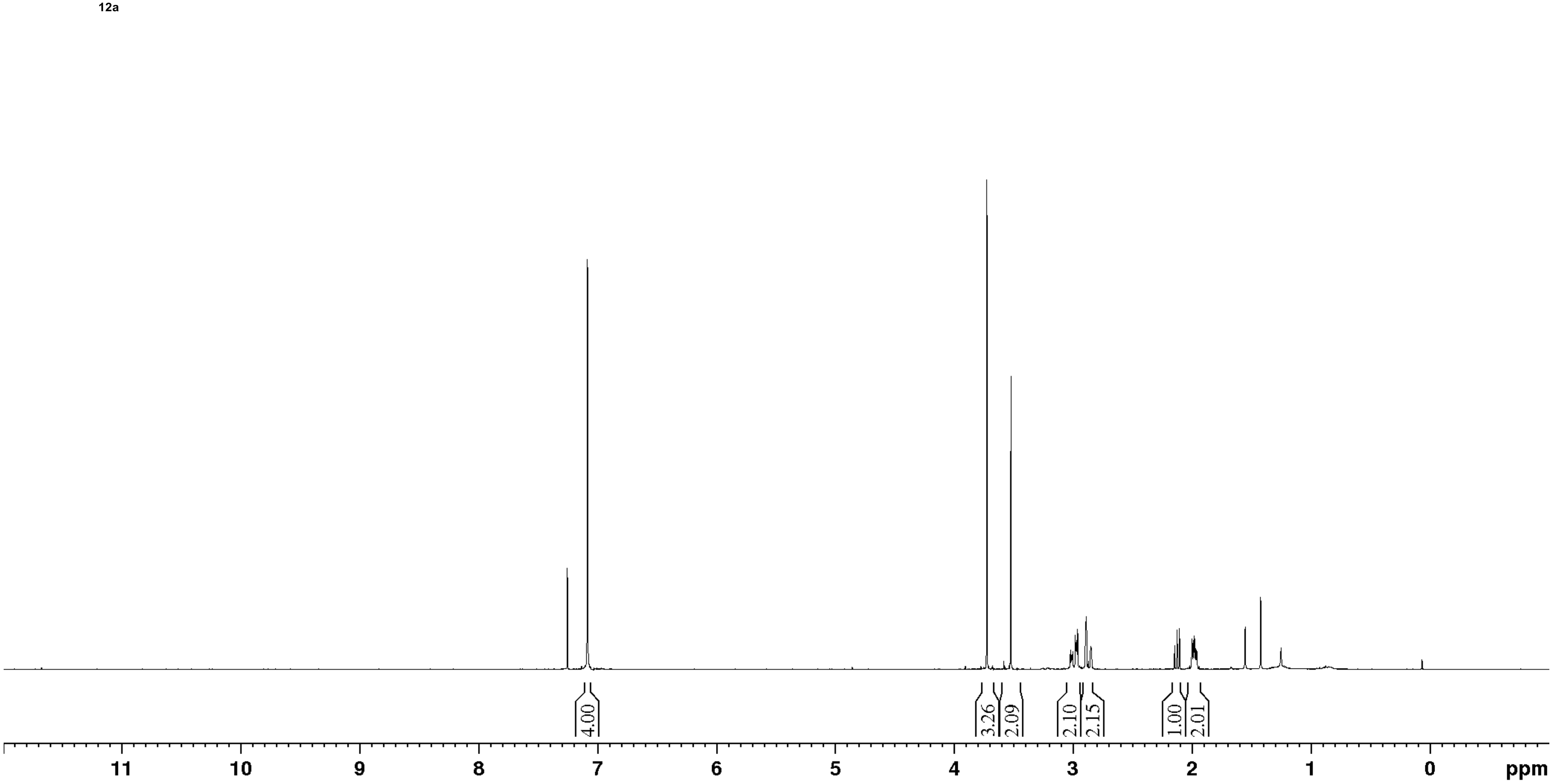

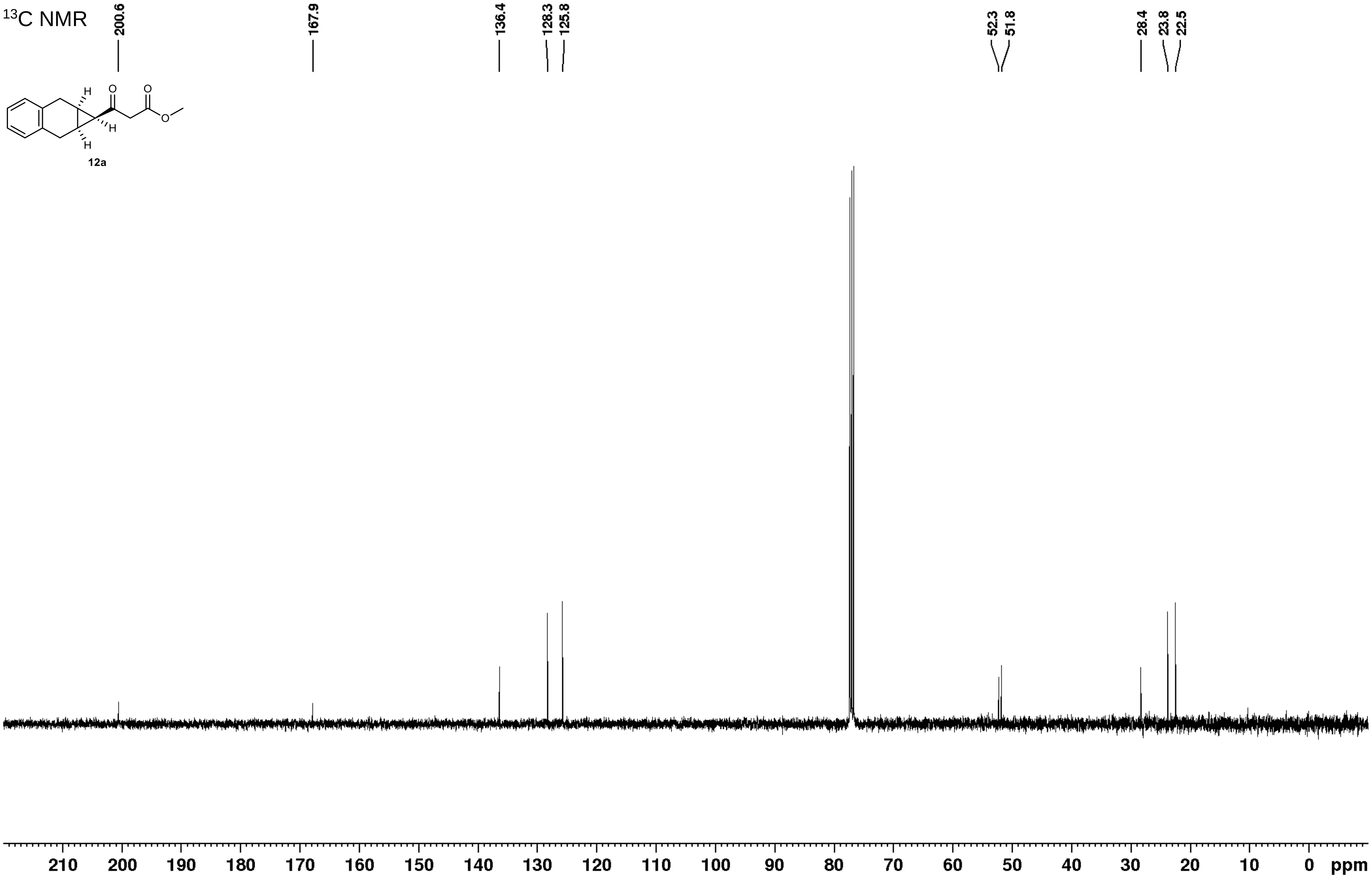

13C NMR
200.6
167.9
136.4
128.3
125.8
52.3
51.8
28.4
23.8
22.5
12a
210 200 190 180 170 160 150 140 130 120 110 100 90 80 70 60 50 40 30 20 10 0 ppm

^{1}H, ^{1}H COSY

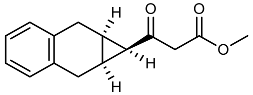

12a

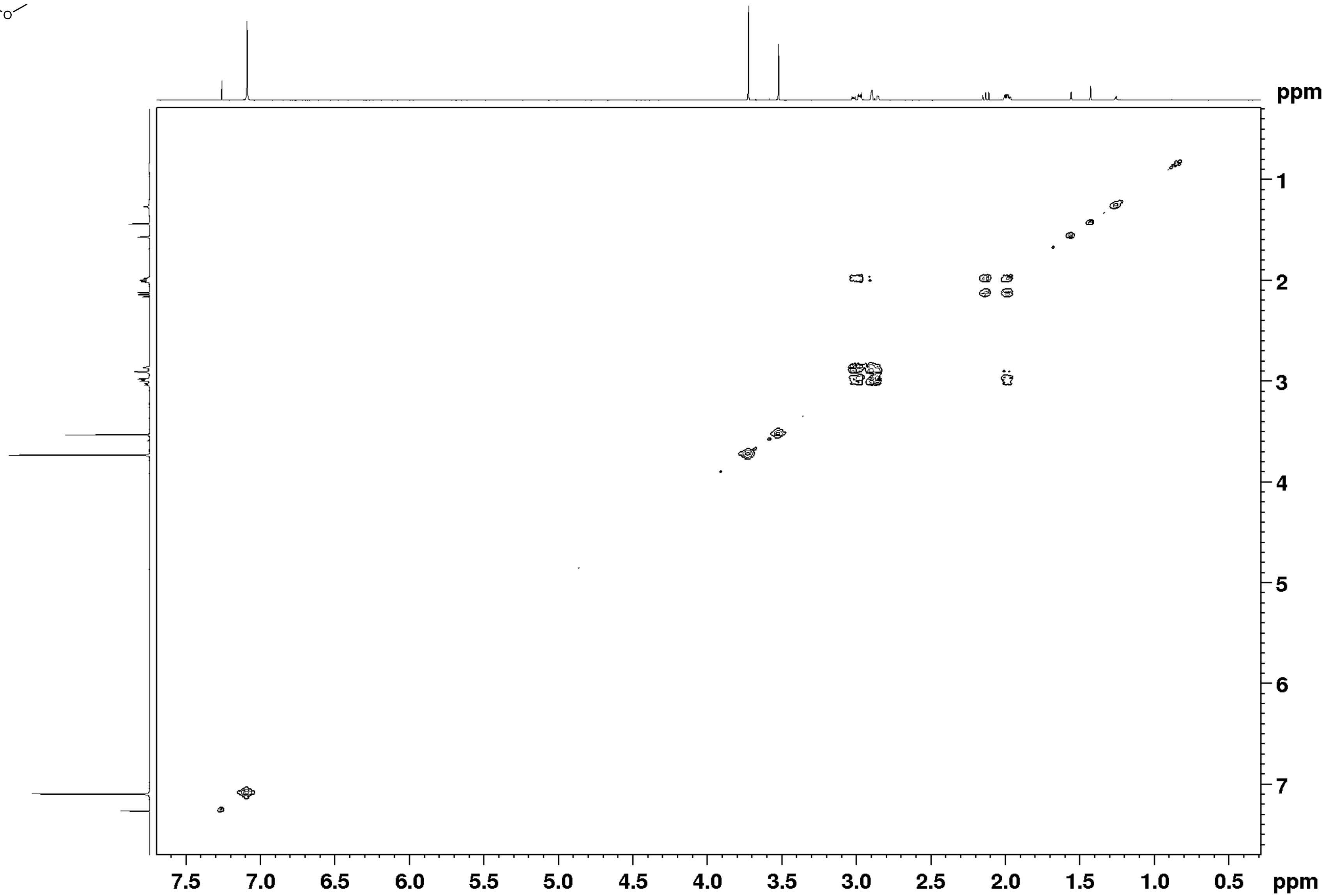

^{1}H, ^{13}C HSQC

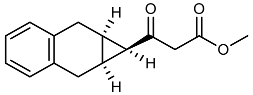

12a

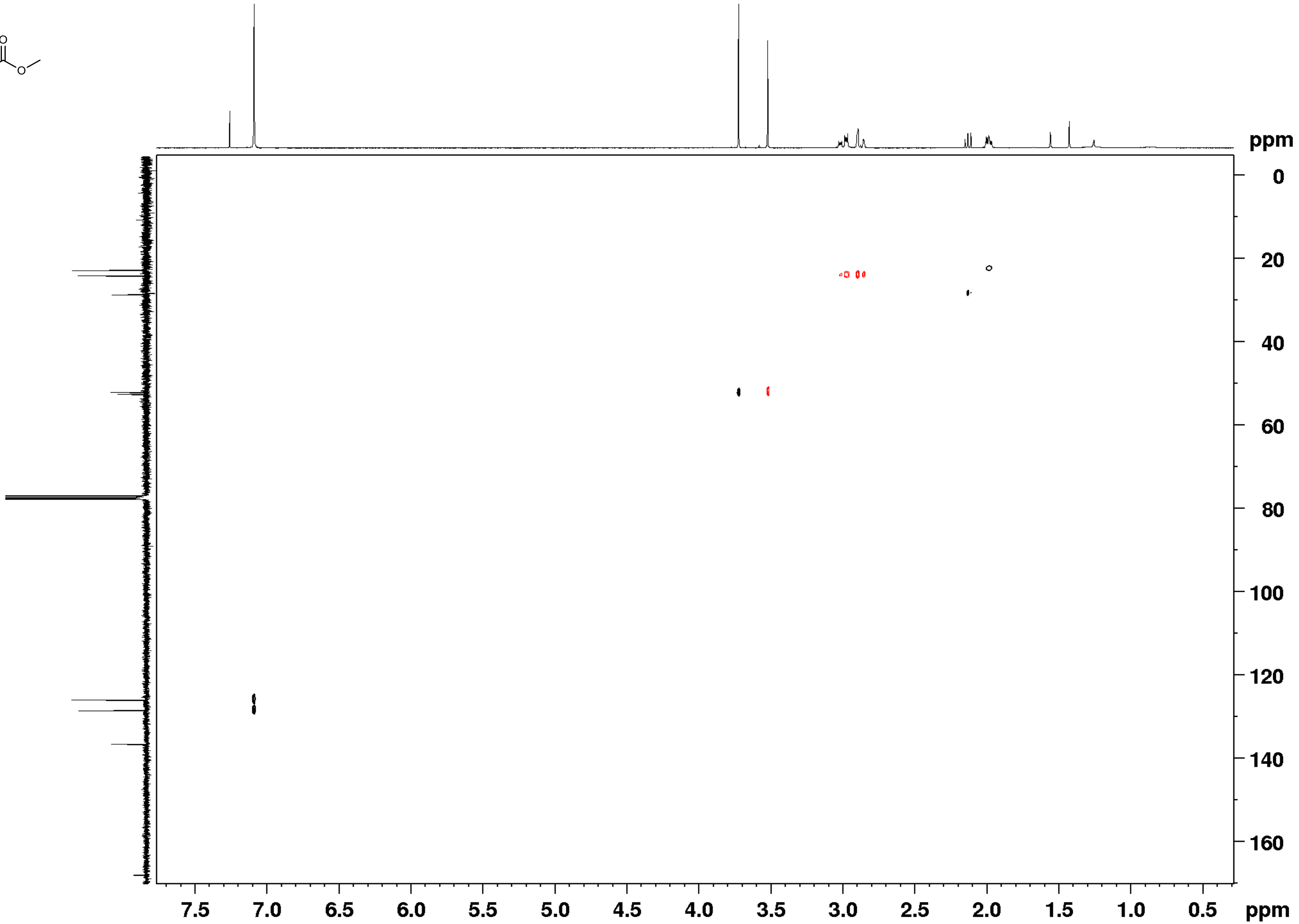

^1H, ^{13}C HMBC

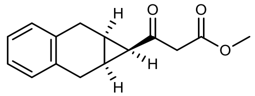

12a

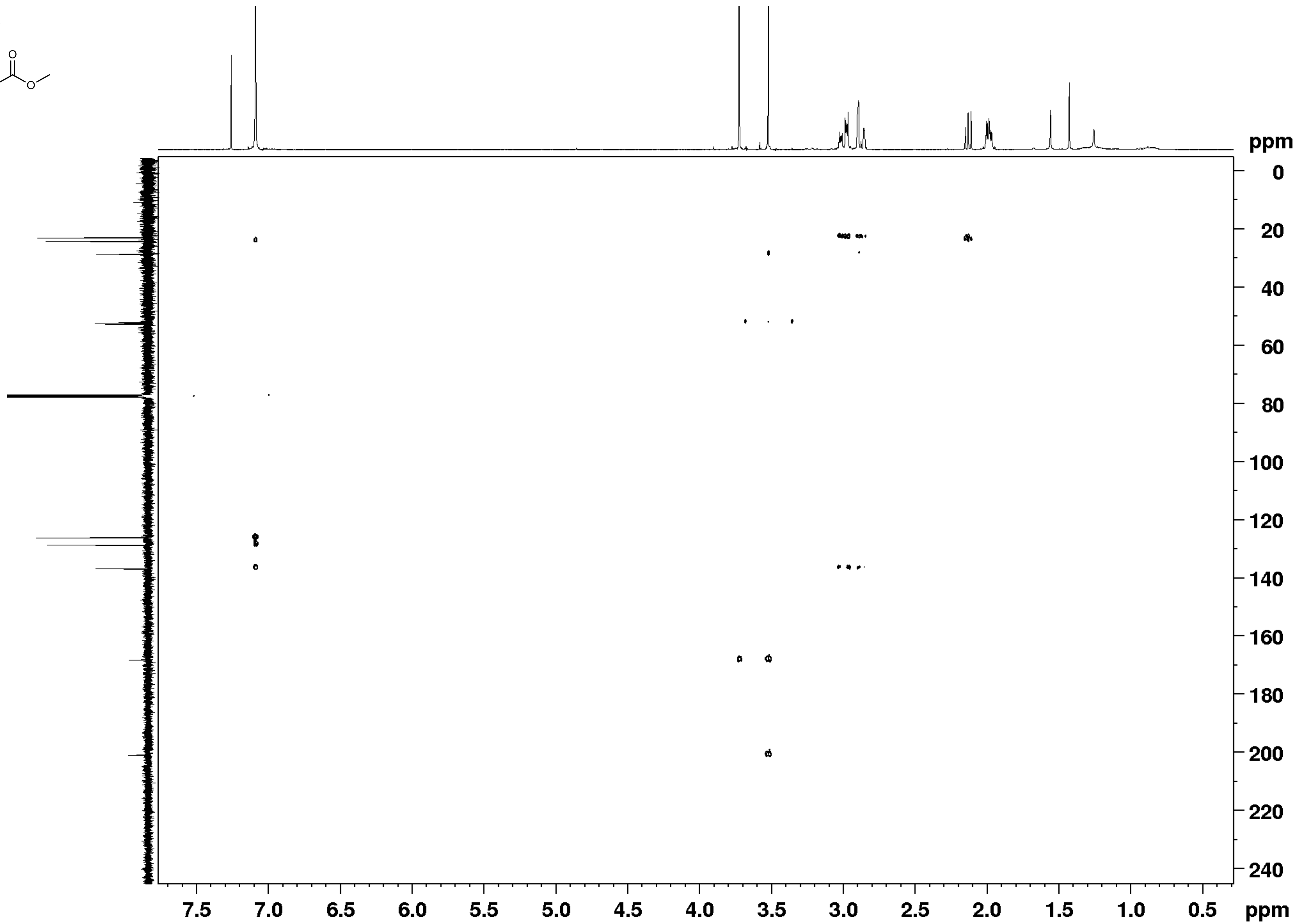

^1H, ^1H NOESY

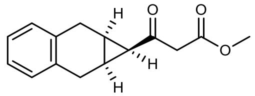

12a

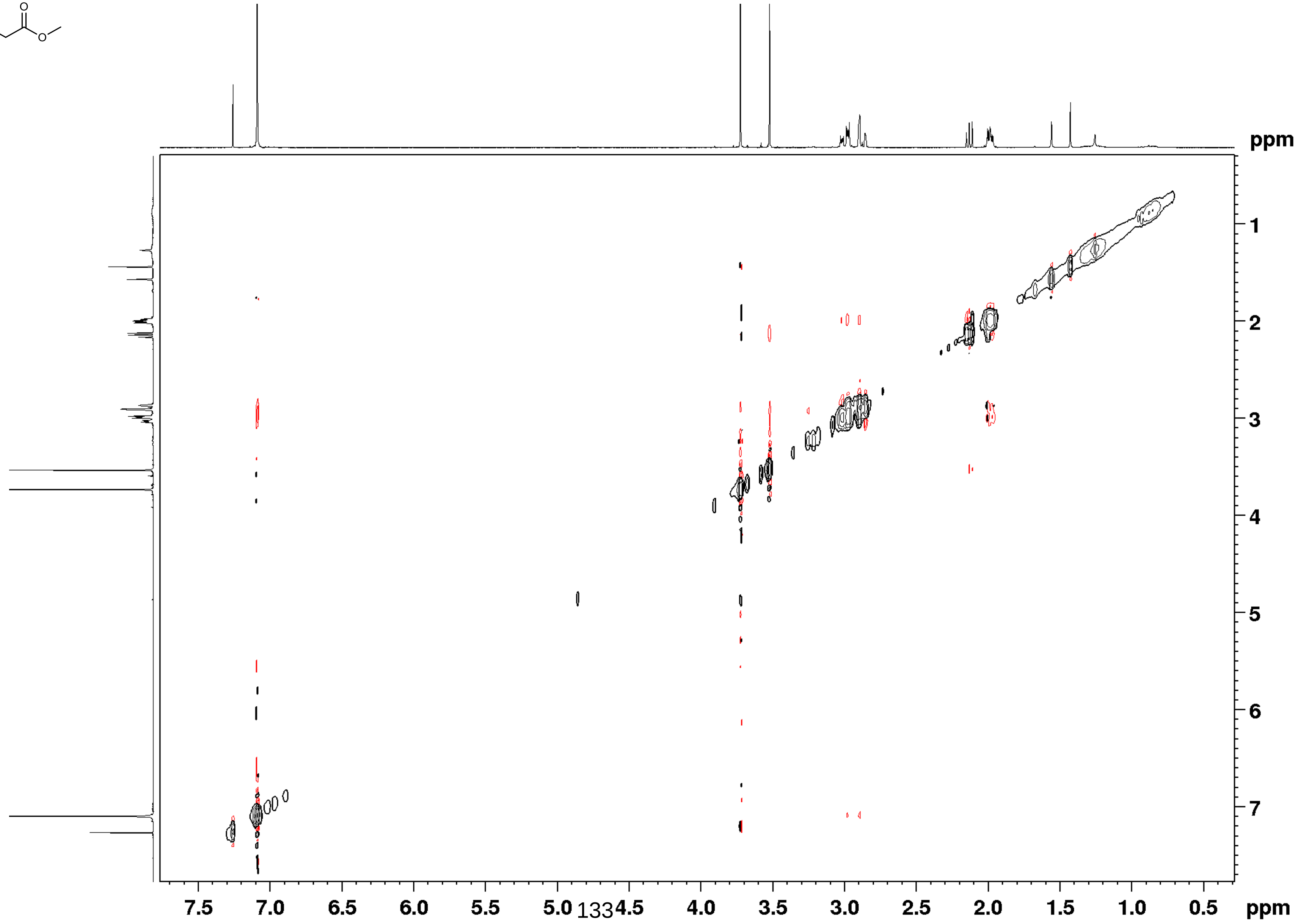

HRMS

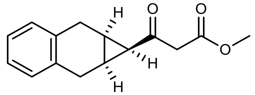

12a

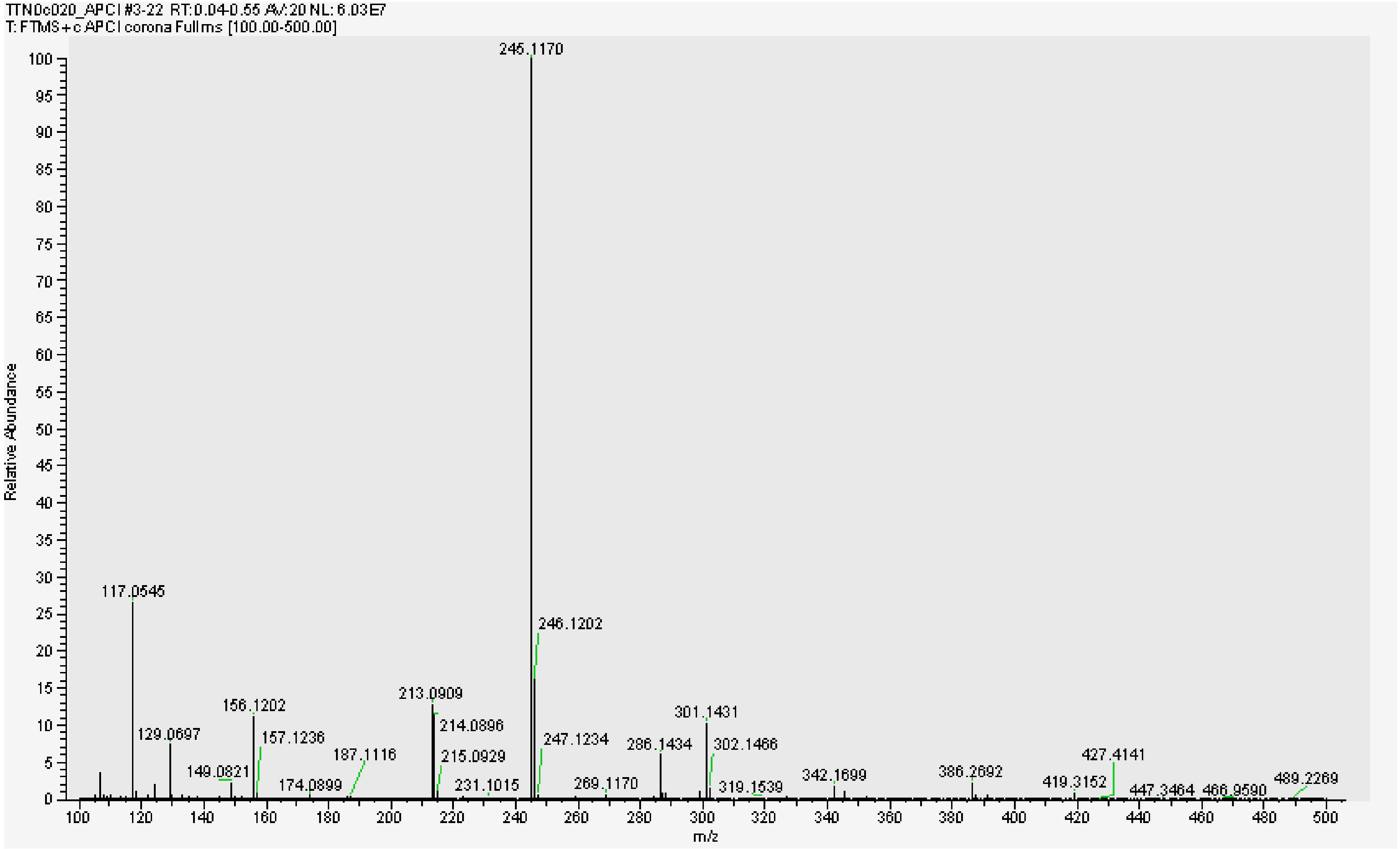
TTN0c020_APCI #3-22 RT: 0.04-0.55 AV: 20 NL: 6.03E7
T: FTMS + c APCI corona Full ms [100.00-500.00]
Relative Abundance
m/z
245.1170
117.0545
129.0697
156.1202
157.1236
149.0821
174.0899
187.1116
213.0909
214.0896
215.0929
231.1015
246.1202
247.1234
269.1170
286.1434
301.1431
302.1466
319.1539
342.1699
386.2692
419.3152
427.4141
447.3464
466.9590
489.2269

IR

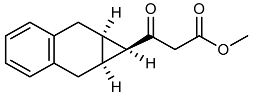

12a

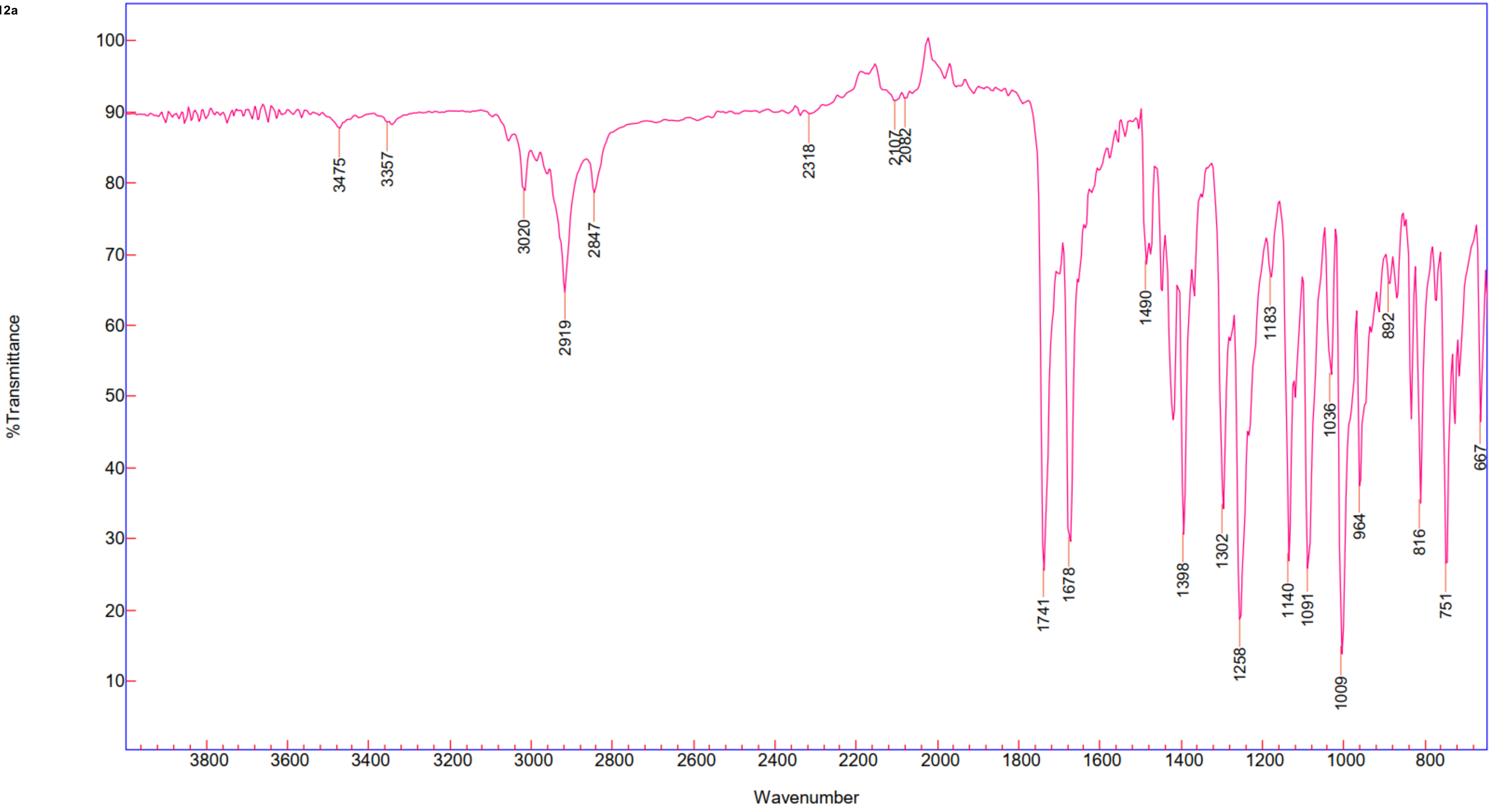

%Transmittance
Wavenumber
100
90
80
70
60
50
40
30
20
10
3800
3600
3400
3200
3000
2800
2600
2400
2200
2000
1800
1600
1400
1200
1000
800
3475
3357
3020
2919
2847
2318
2107
2082
1741
1678
1490
1398
1302
1258
1183
1140
1091
1036
1009
964
892
816
751
667

2.2.7 *tert*-Butyl 3-oxo-3-((1*s*,1a*R*,7a*S*)-1a,2,7,7a-tetrahydro-1*H*-cyclopropa[b]naphthalen-1-yl)propanoate (12b)

^{1}H NMR

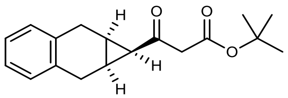

12b

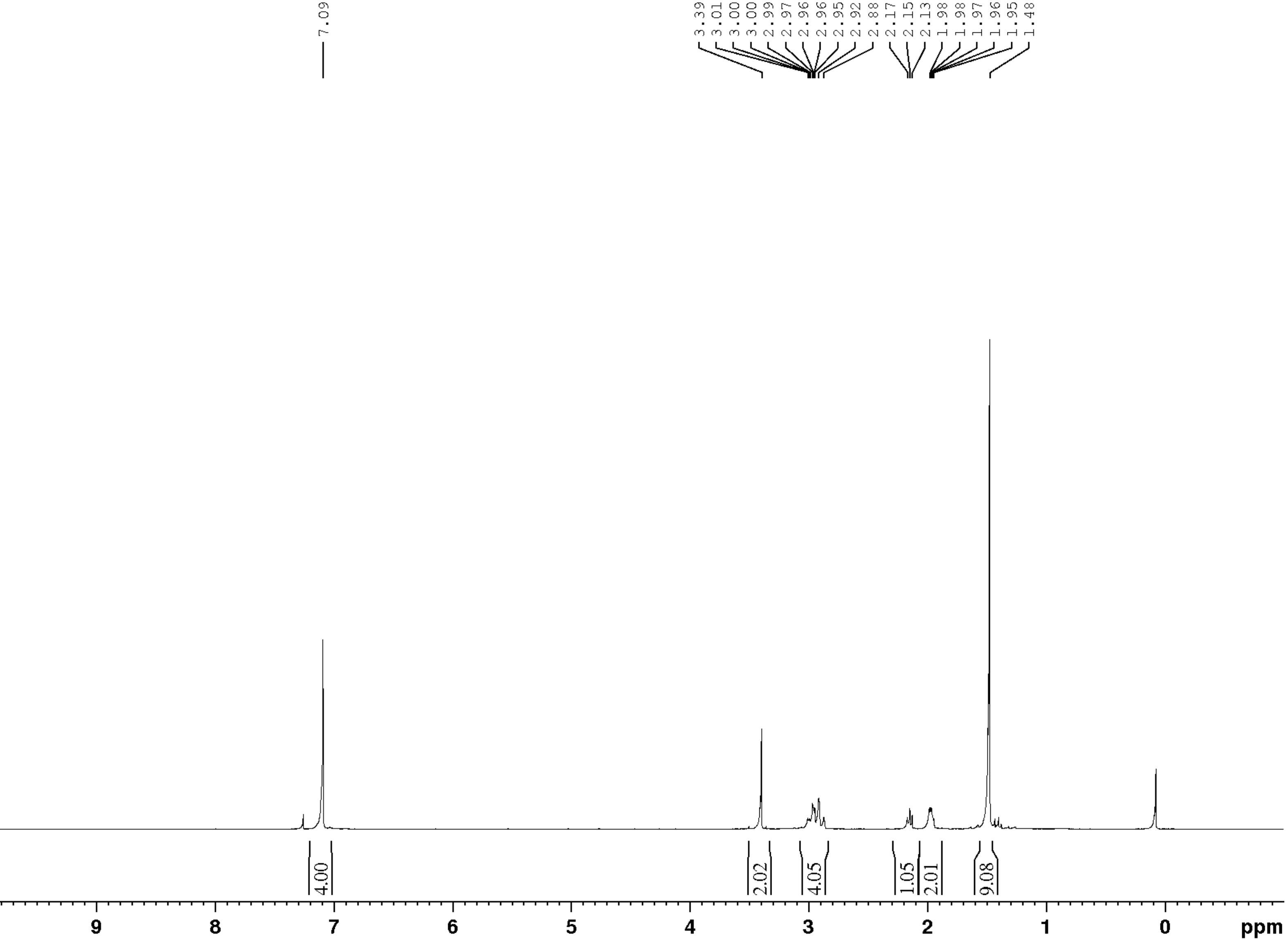

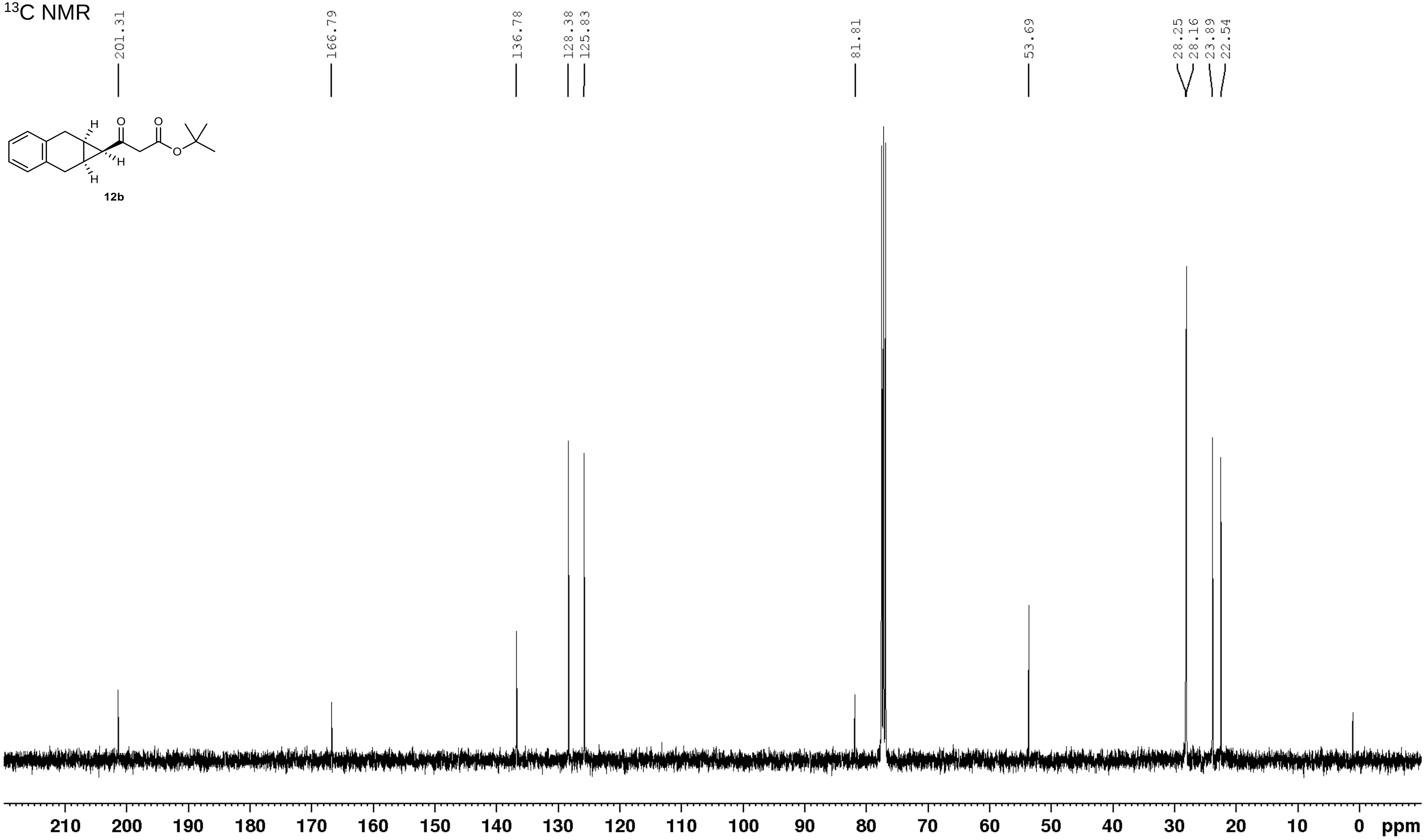
^{13}C NMR
201.31
166.79
136.78
128.38
125.83
81.81
53.69
28.25
28.16
23.89
22.54
12b
210 200 190 180 170 160 150 140 130 120 110 100 90 80 70 60 50 40 30 20 10 0 ppm

^{1}H, ^{1}H COSY

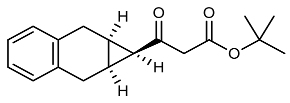

12b

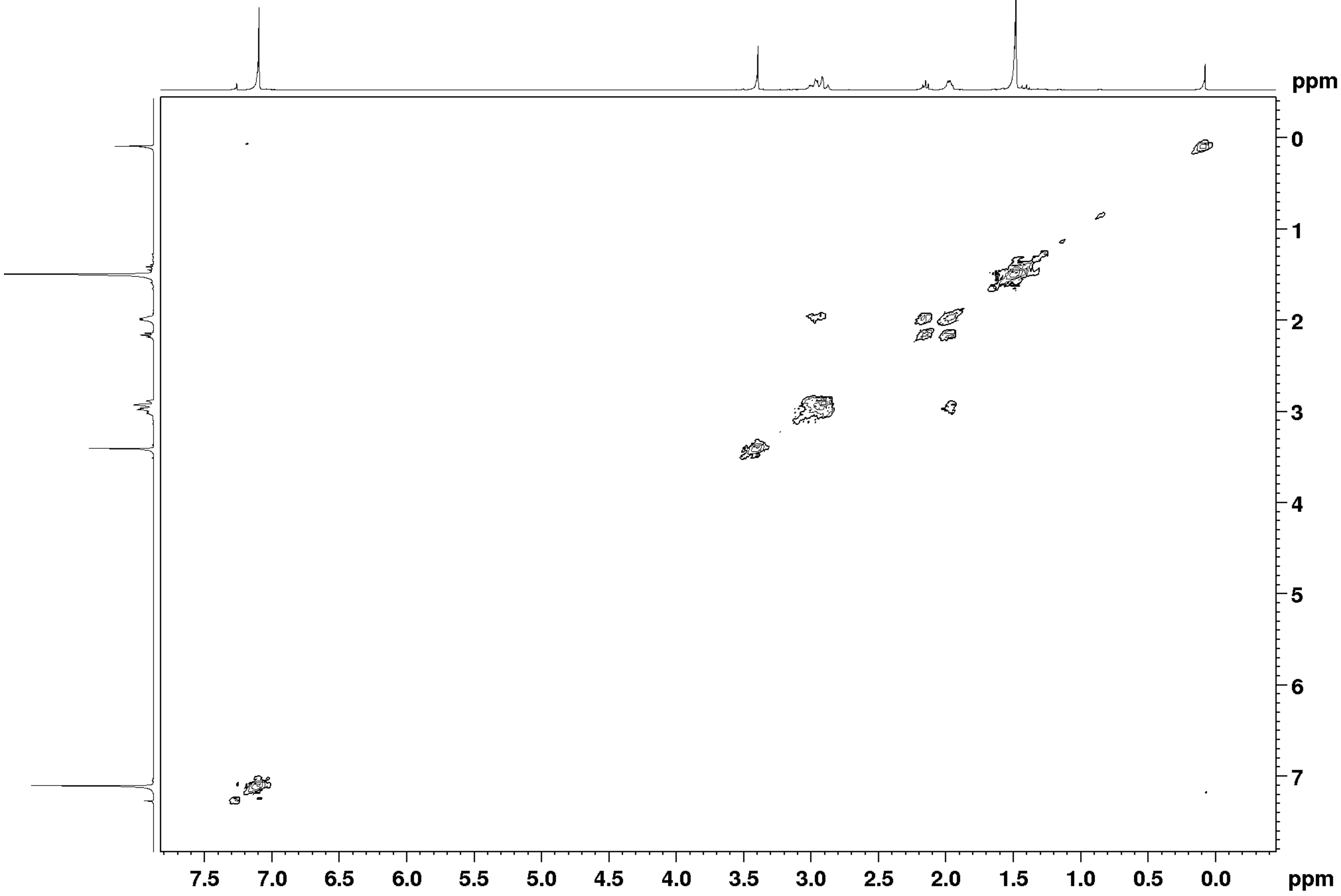

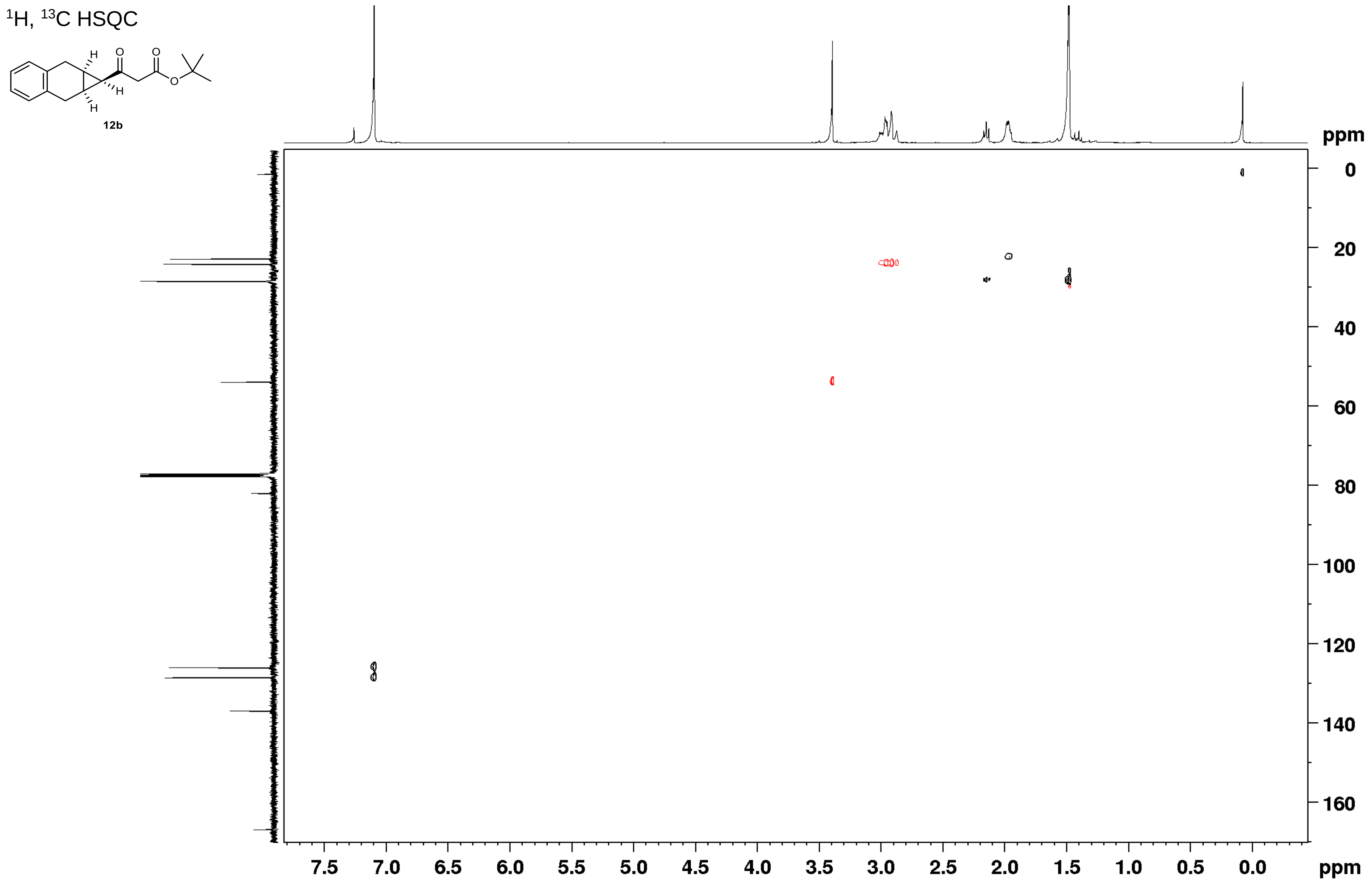

^{1}H, ^{13}C HSQC
12b
ppm
0
20
40
60
80
100
120
140
160
7.5
7.0
6.5
6.0
5.5
5.0
4.5
4.0
3.5
3.0
2.5
2.0
1.5
1.0
0.5
0.0
ppm

^{1}H, ^{13}C HMBC

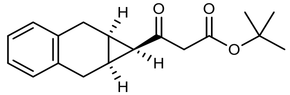

12b

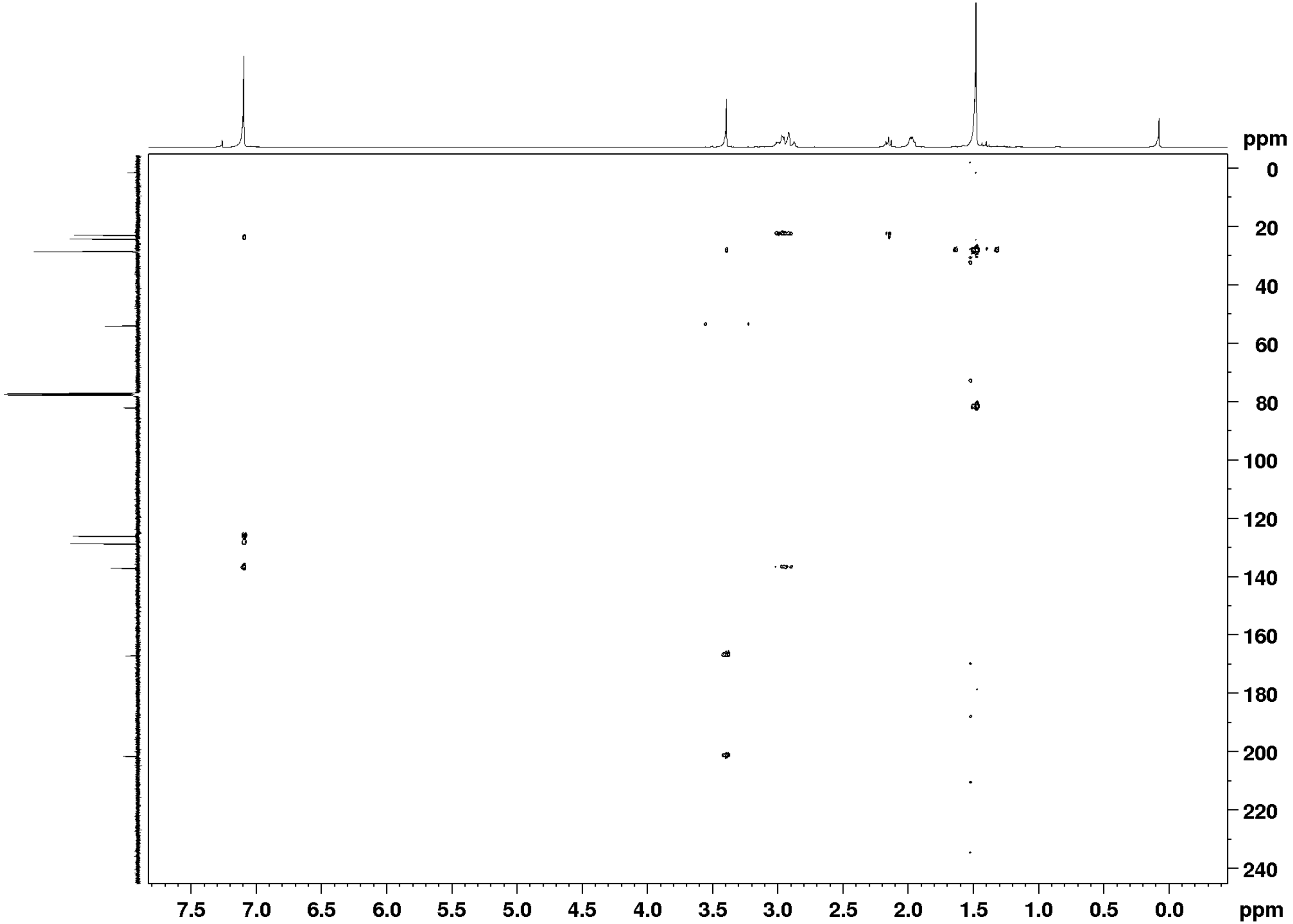

HRMS

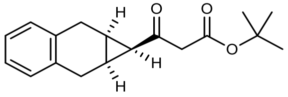

12b

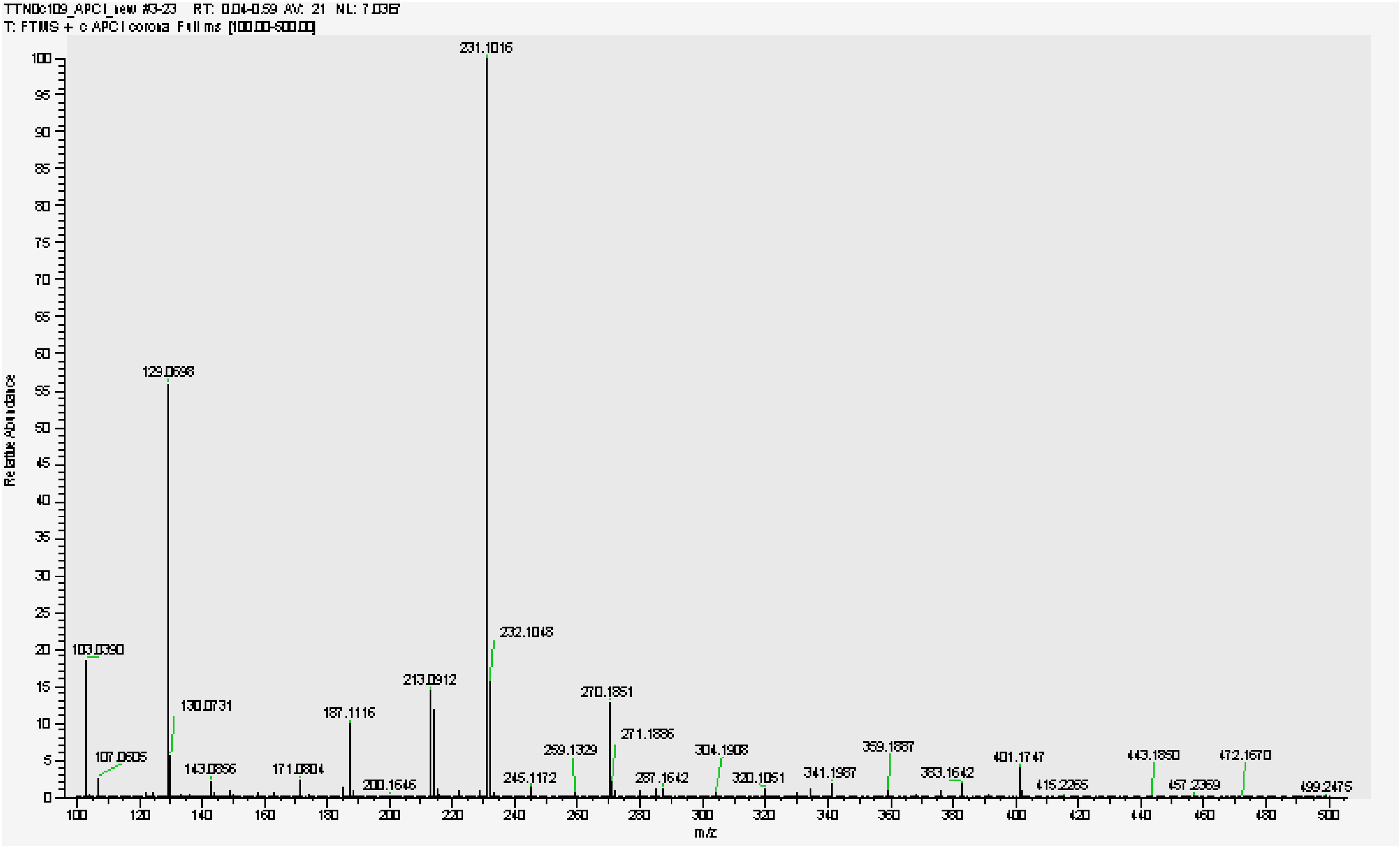
TTNDc109_APCI_new #3-23 RT: 0.04-0.59 AV: 21 NL: 7.03E7
T: FTMS + c APCI corona Full ms [100.00-500.00]
231.1016
129.0698
232.1048
103.0390
213.0912
270.1851
130.0731
187.1116
271.1886
107.0605
143.0866
171.0804
200.1646
245.1172
259.1329
287.1642
304.1908
320.1051
341.1987
359.1887
383.1642
401.1747
415.2265
443.1850
457.2369
472.1670
499.2475
Relative Abundance
m/z

IR

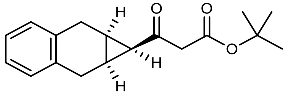

H
O
O
O
H
H

12b

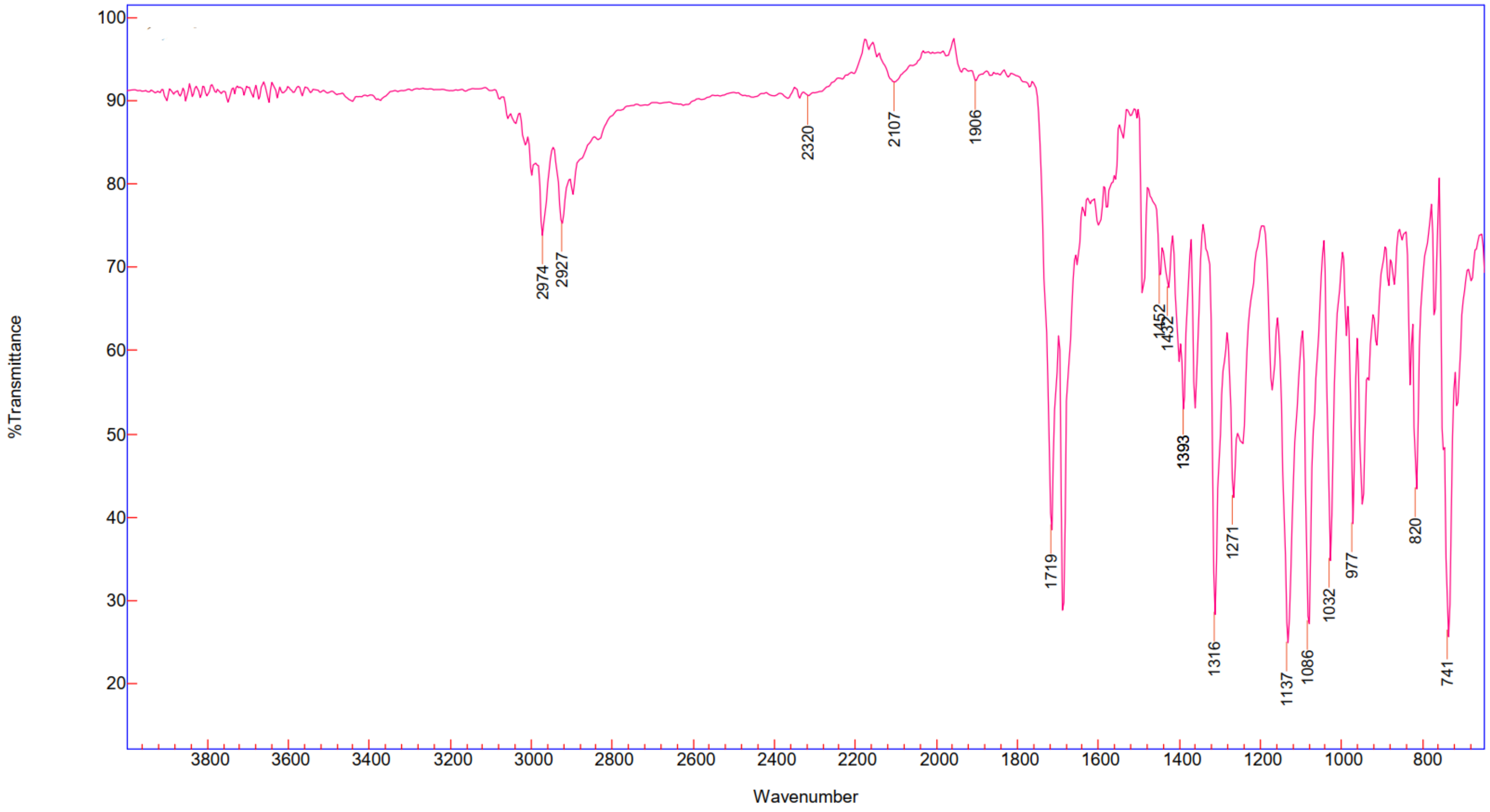

%Transmittance
Wavenumber
100
90
80
70
60
50
40
30
20
3800
3600
3400
3200
3000
2800
2600
2400
2200
2000
1800
1600
1400
1200
1000
800
2974
2927
2320
2107
1906
1719
1452
1432
1393
1316
1271
1137
1086
1032
977
820
741

2.2.8 Methyl-2-diazo-3-oxo-3-((*1s*,1a*R*,7a*S*)-1a,2,7,7a-tetrahydro-1*H*-cyclopropa[b]naphthalen-1-yl)propanoate (13a)

^{1}H NMR

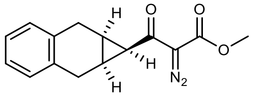

13a

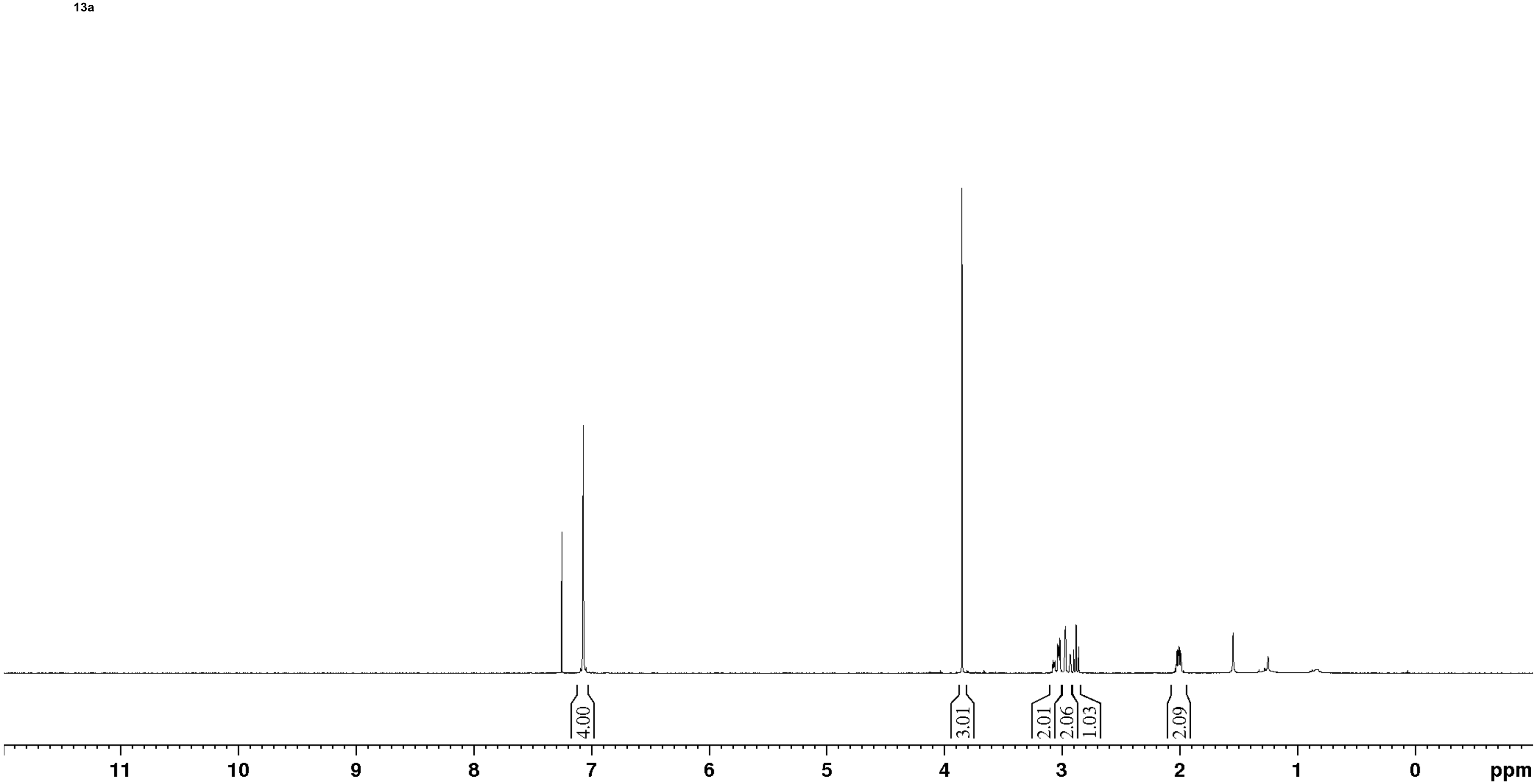

^{13}C NMR

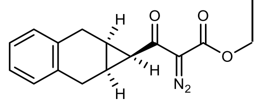

13a

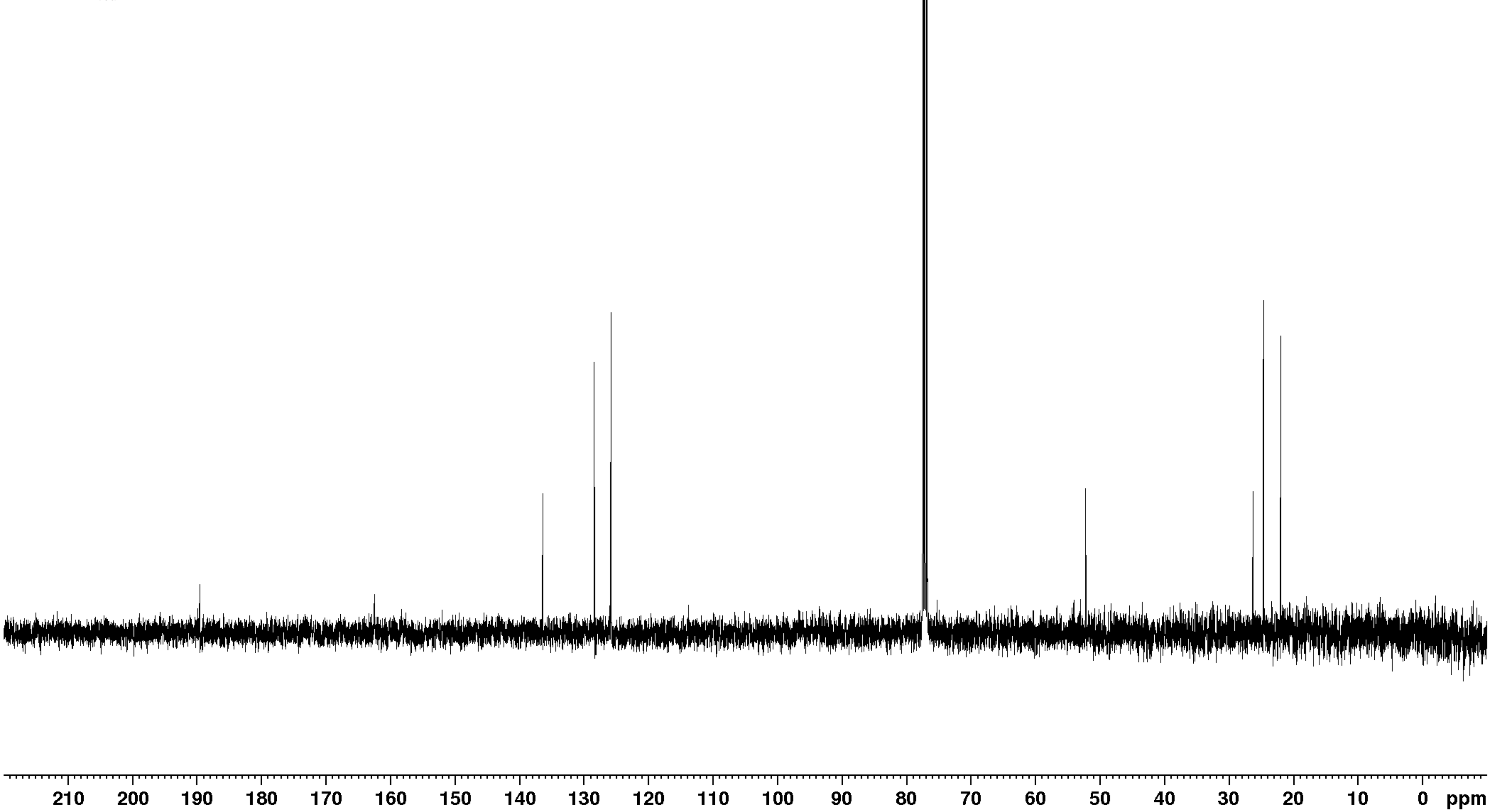

189.5
162.5
136.4
128.4
125.8
52.3
26.3
24.7
22.0
210 200 190 180 170 160 150 140 130 120 110 100 90 80 70 60 50 40 30 20 10 0 ppm

^1H, ^1H COSY

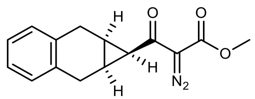

13a

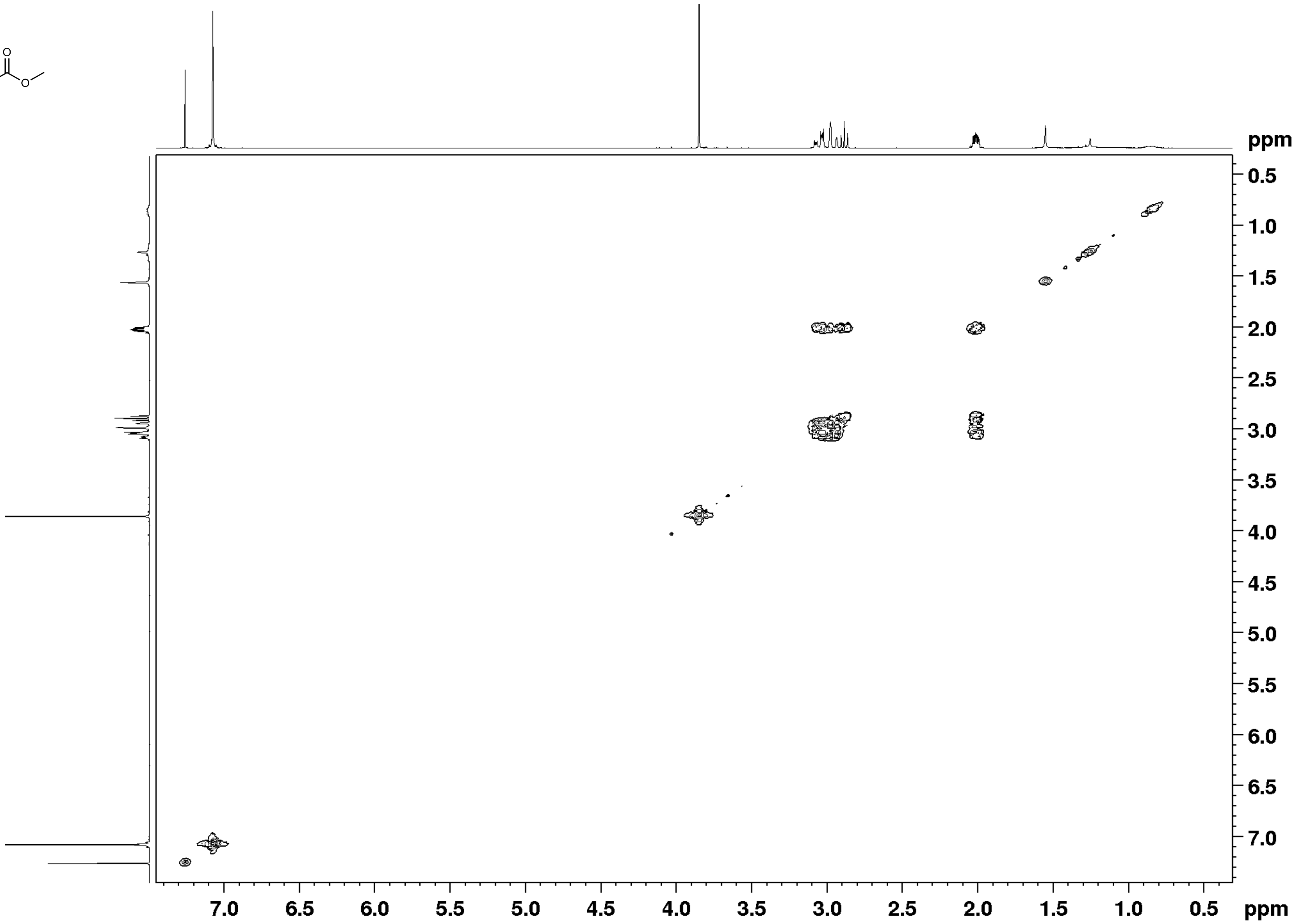

^1H, ^{13}C HSQC

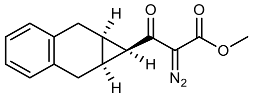

13a

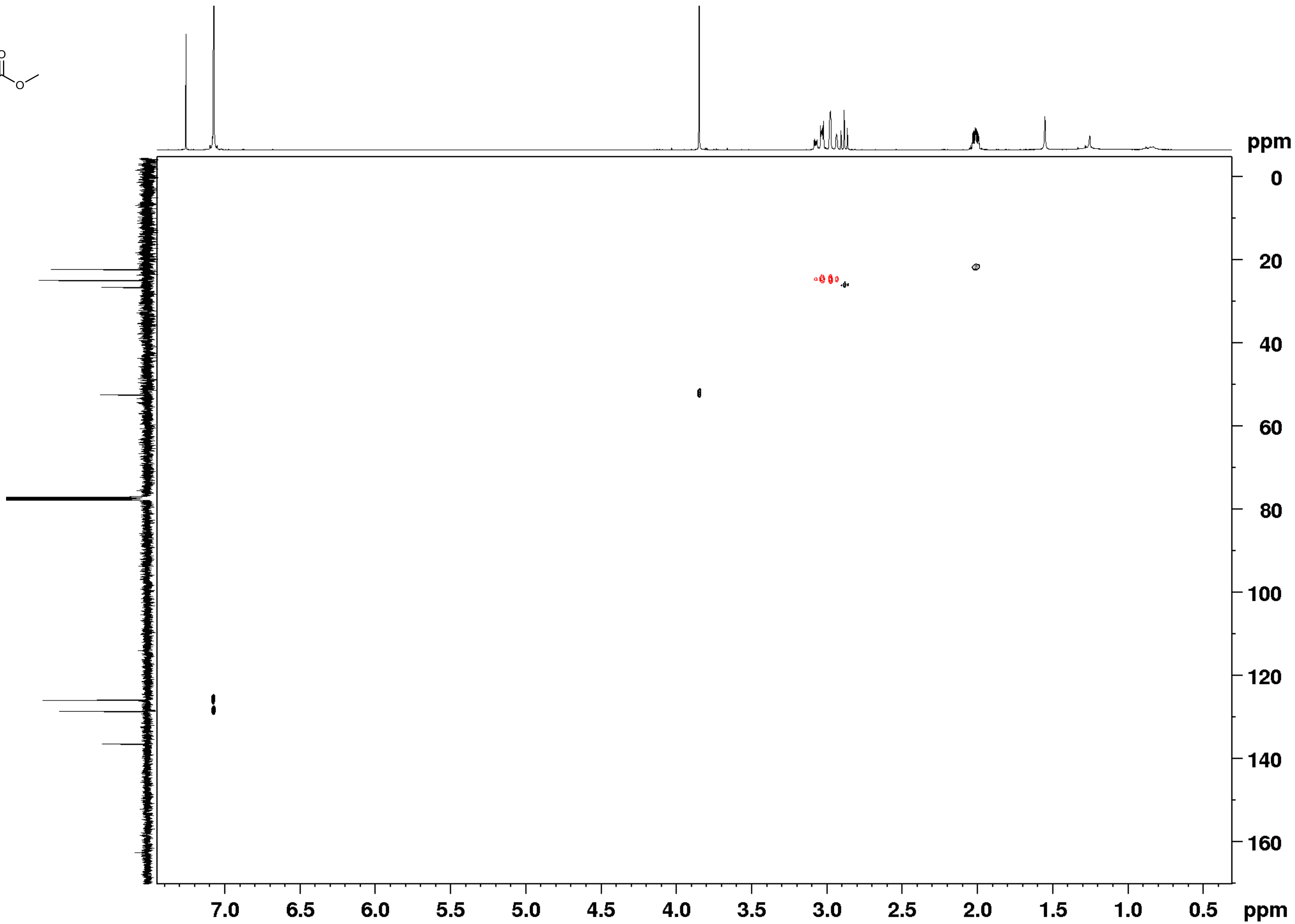

^{1}H, ^{13}C HMBC

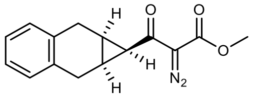

13a

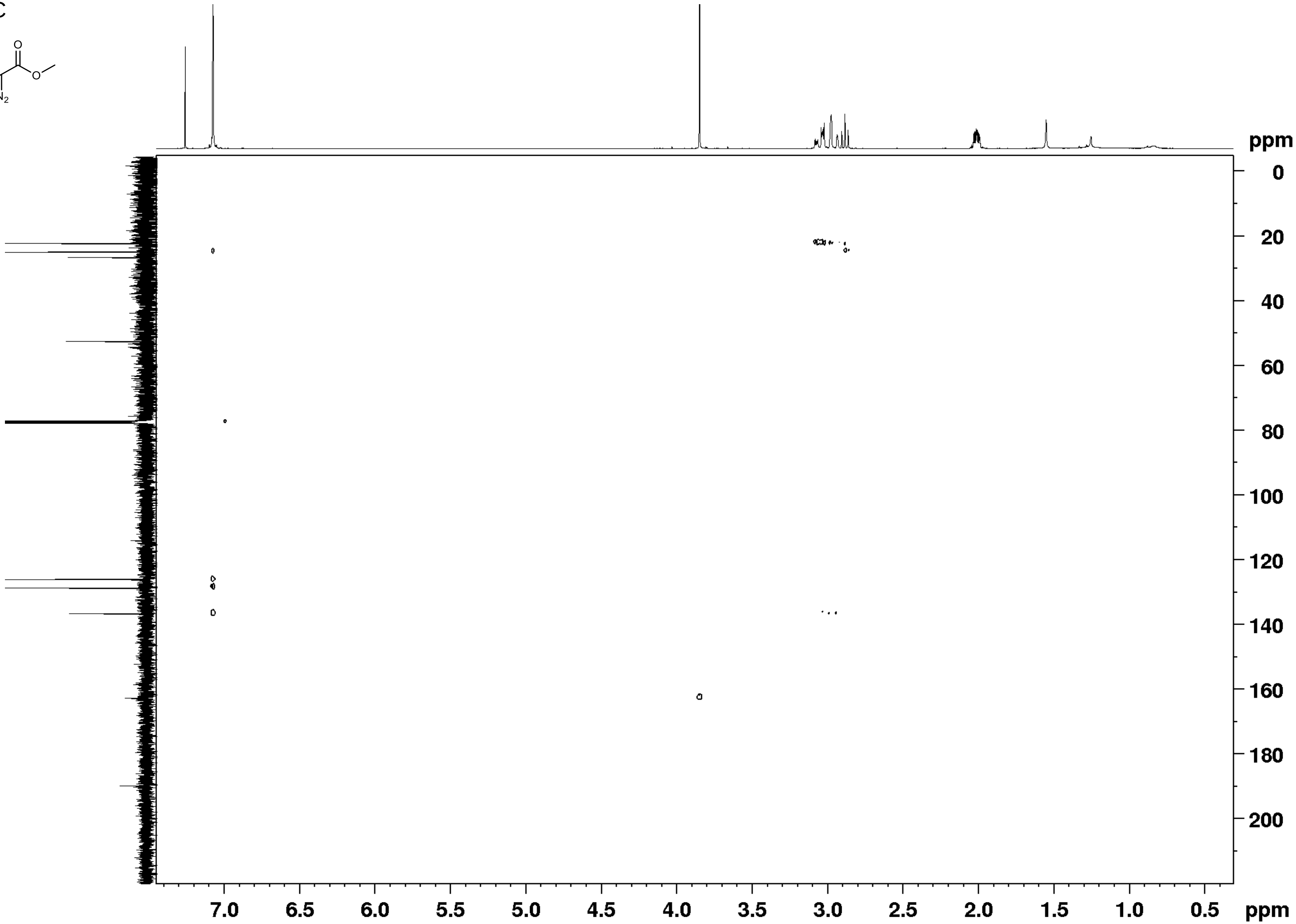

^1H, ^1H NOESY

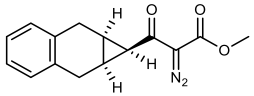

13a

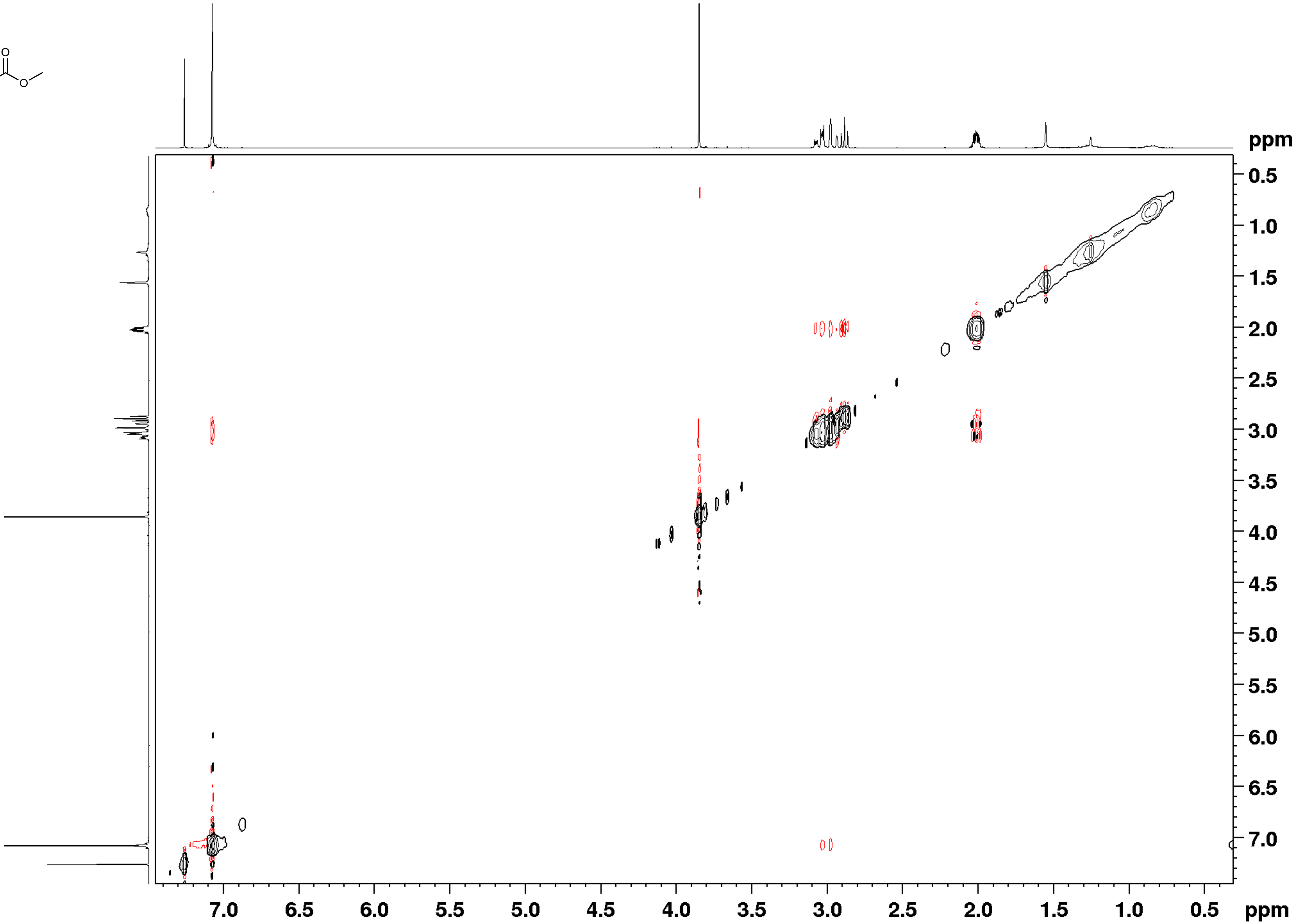

HRMS

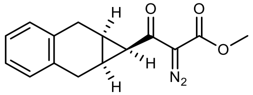

13a

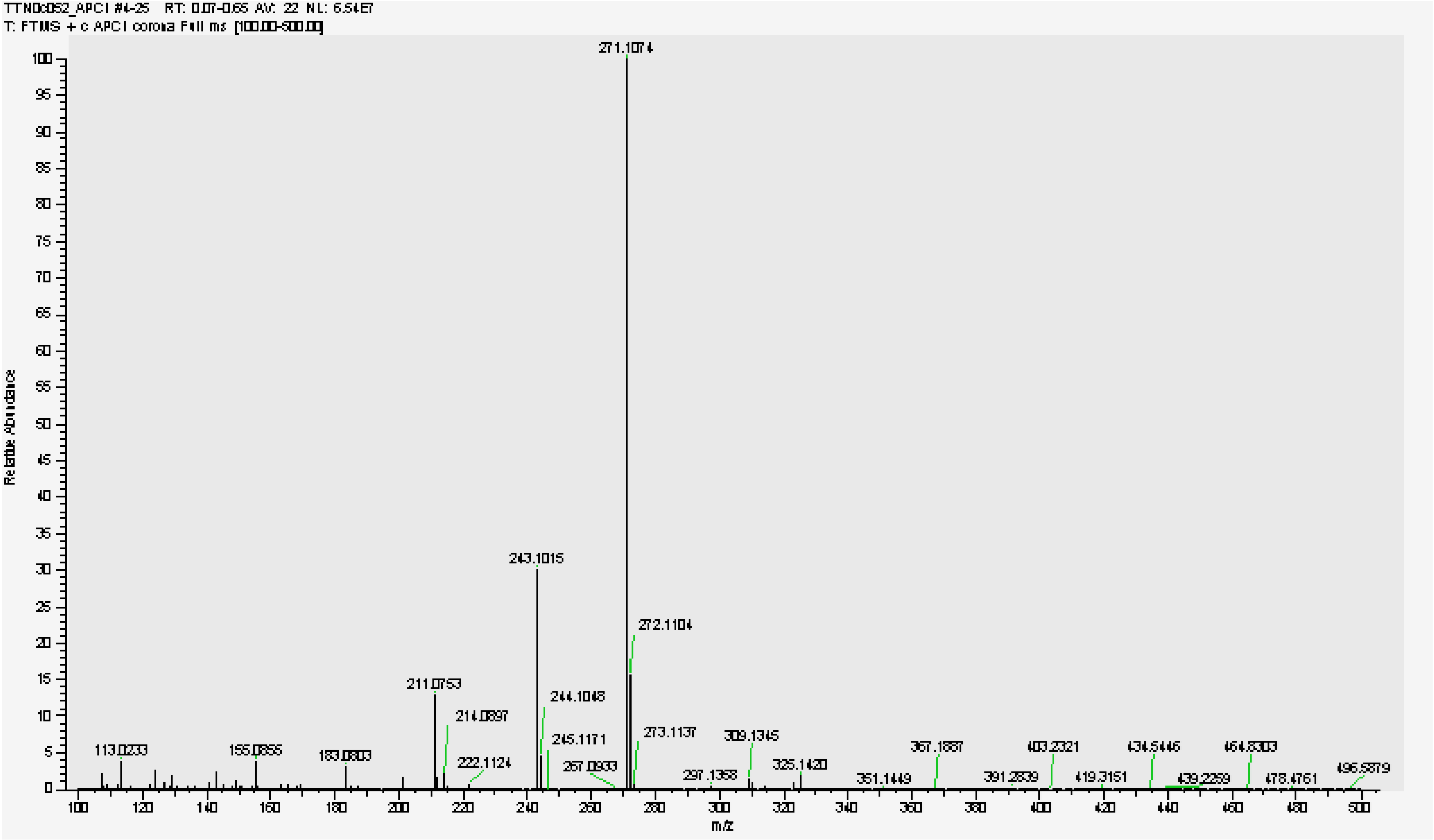

TTN0c052_APCI #4-25 RT: 0.07-0.65 AV: 22 NL: 6.54E7
T: FTMS + c APCI corona Full ms [100.00-500.00]
271.1074
243.1015
272.1104
211.0753
244.1048
214.0897
245.1171
273.1137
309.1345
113.0233
155.0855
183.0803
222.1124
267.0933
297.1358
325.1420
351.1449
367.1887
391.2839
403.2321
419.3151
434.5446
439.2259
464.8303
478.4761
496.5879
Relative Abundance
m/z

IR

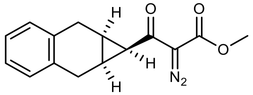
H
O
O
O
H
H
N2

13a

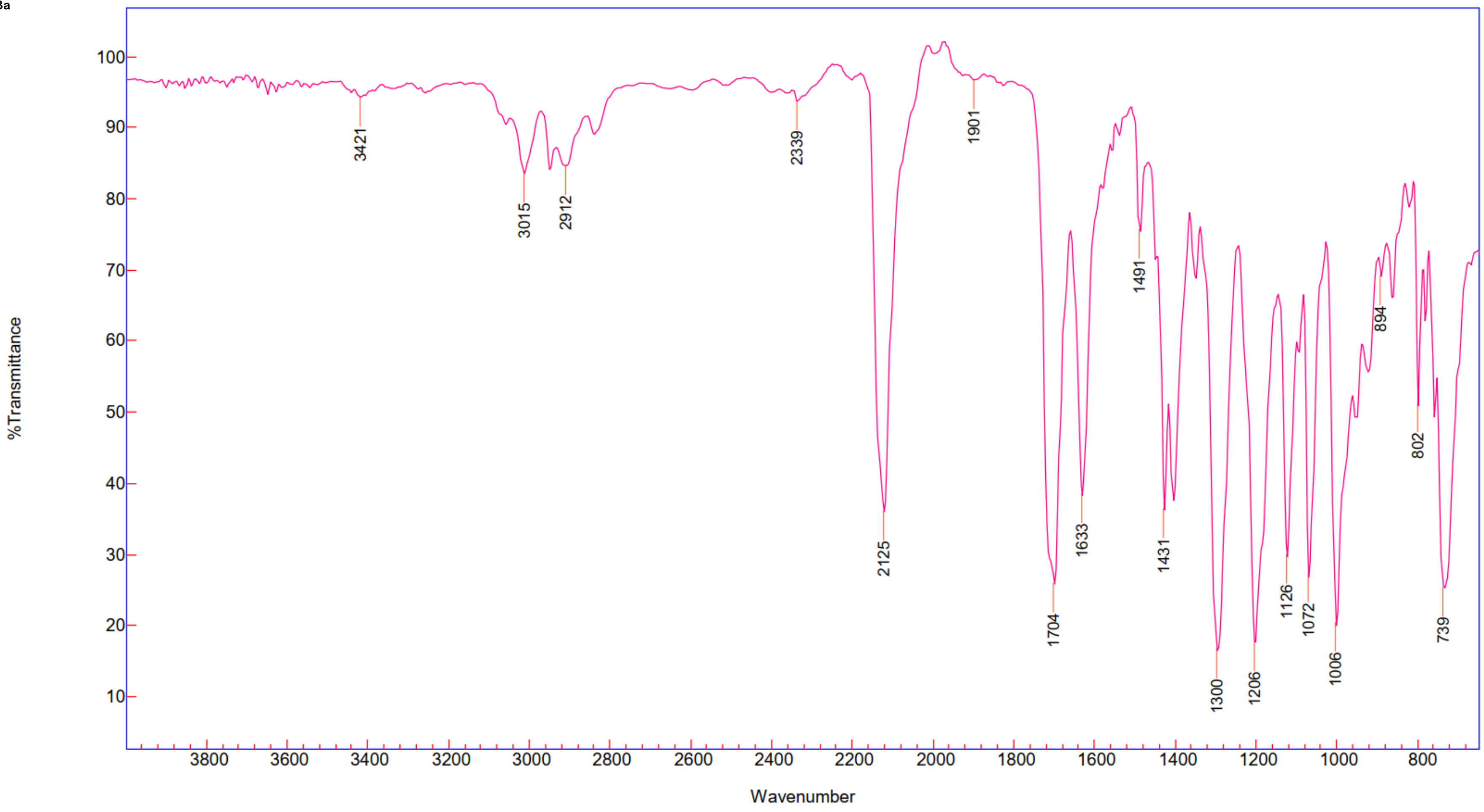
%Transmittance
Wavenumber
100
90
80
70
60
50
40
30
20
10
3800
3600
3400
3200
3000
2800
2600
2400
2200
2000
1800
1600
1400
1200
1000
800
3421
3015
2912
2339
2125
1901
1704
1633
1491
1431
1300
1206
1126
1072
1006
894
802
739

2.2.9 *tert*-Butyl 2-diazo-3-oxo-3-((1*s*,1a*R*,7a*S*)-1a,2,7,7a-tetrahydro-1*H*-cyclopropa[b]naphthalen-1-yl)propanoate (13b)

^{1}H NMR

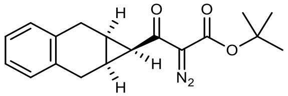

13b

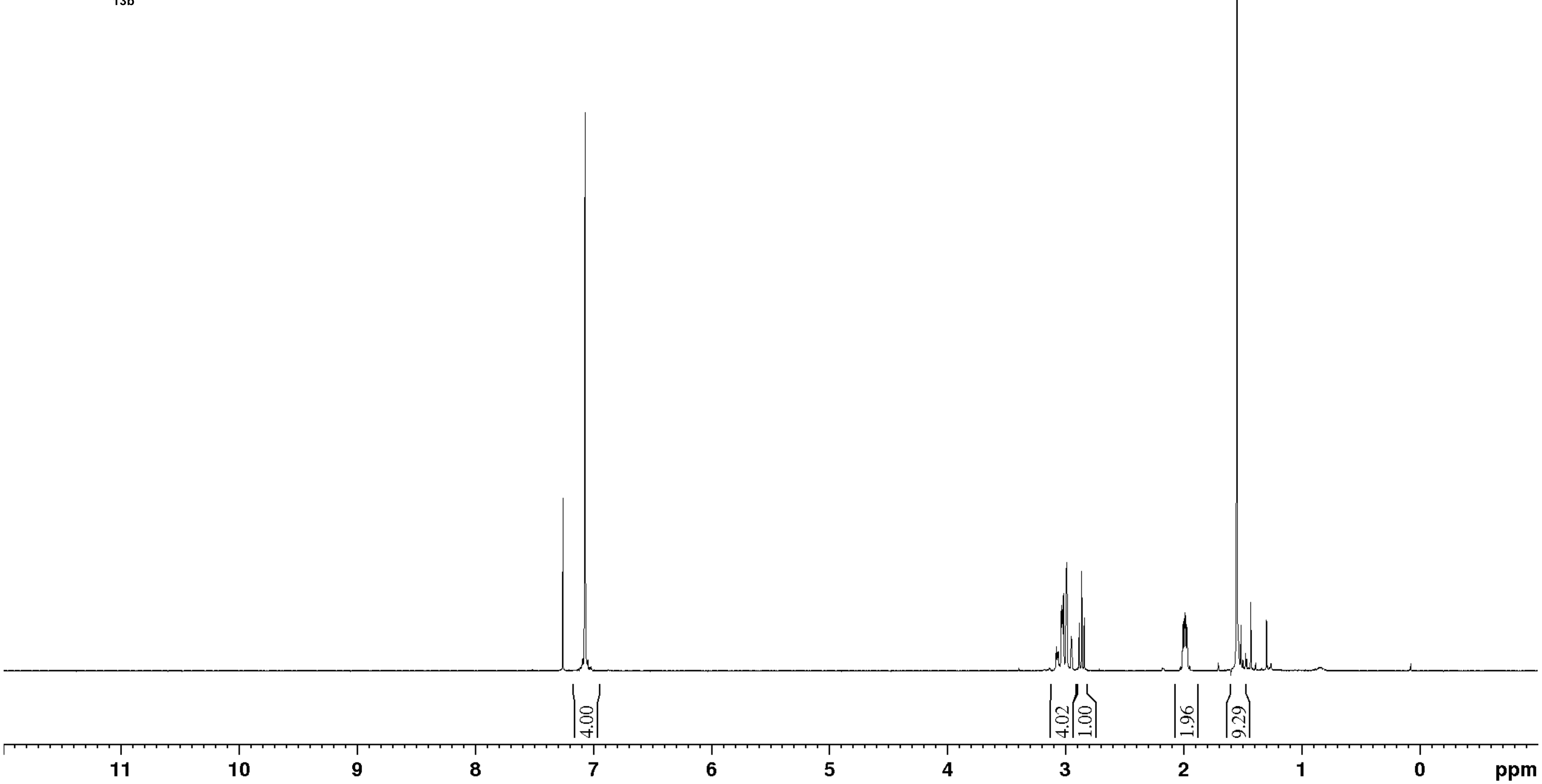

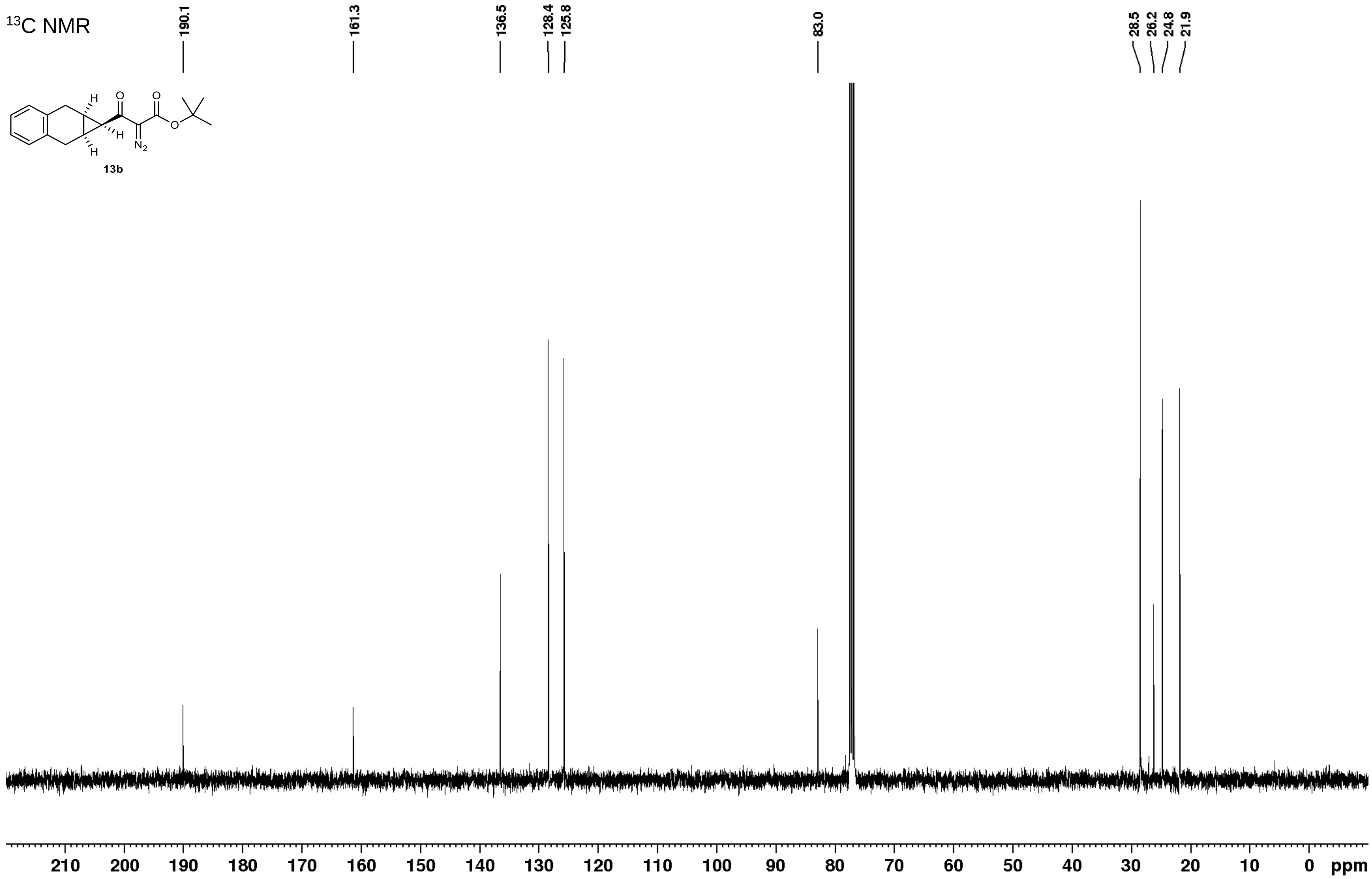

^{13}C NMR
190.1
161.3
136.5
128.4
125.8
83.0
28.5
26.2
24.8
21.9
13b
210 200 190 180 170 160 150 140 130 120 110 100 90 80 70 60 50 40 30 20 10 0 ppm

^1H, ^1H COSY

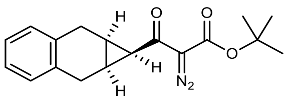

13b

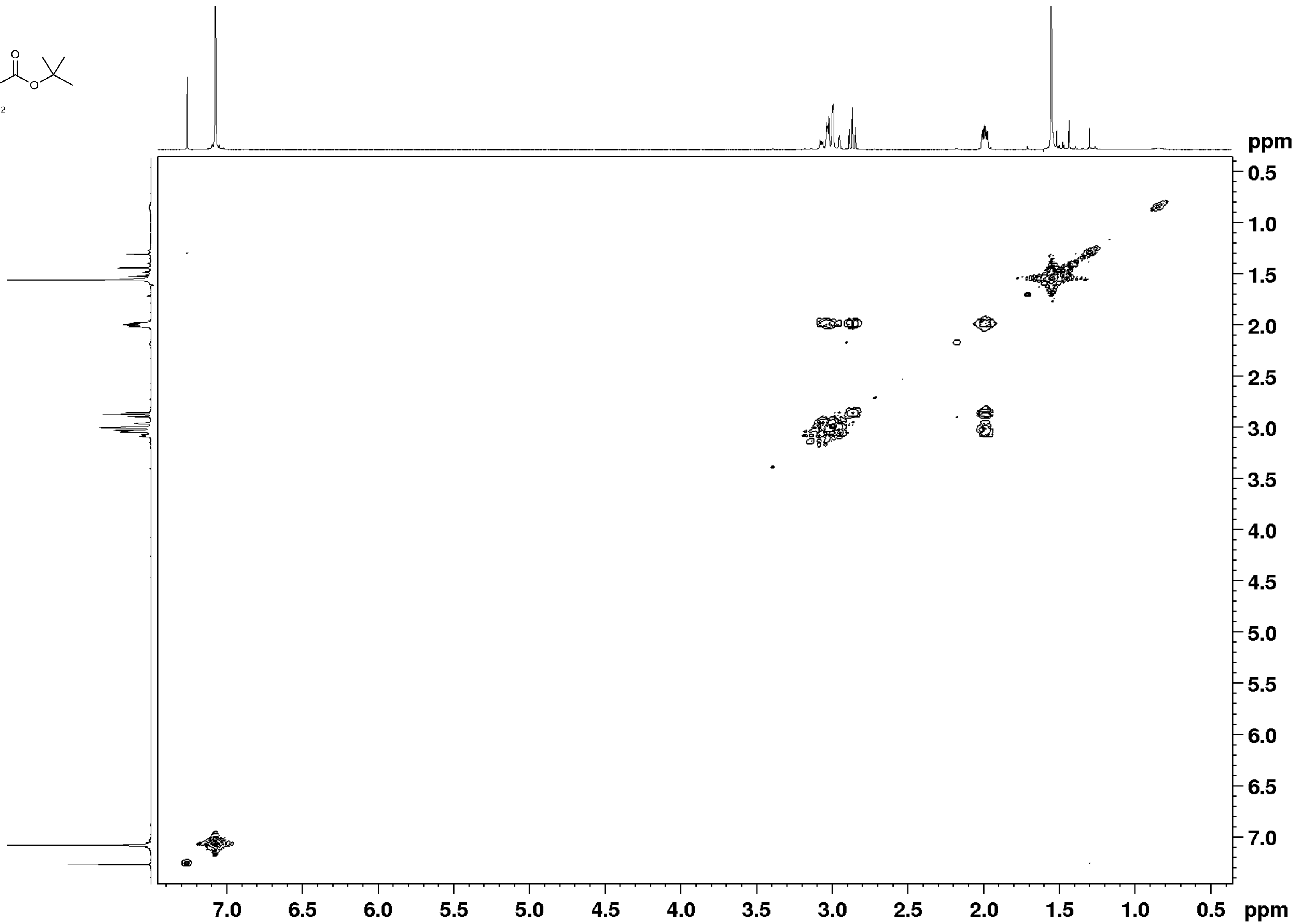

^{1}H, ^{13}C HSQC

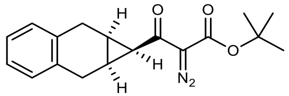

13b

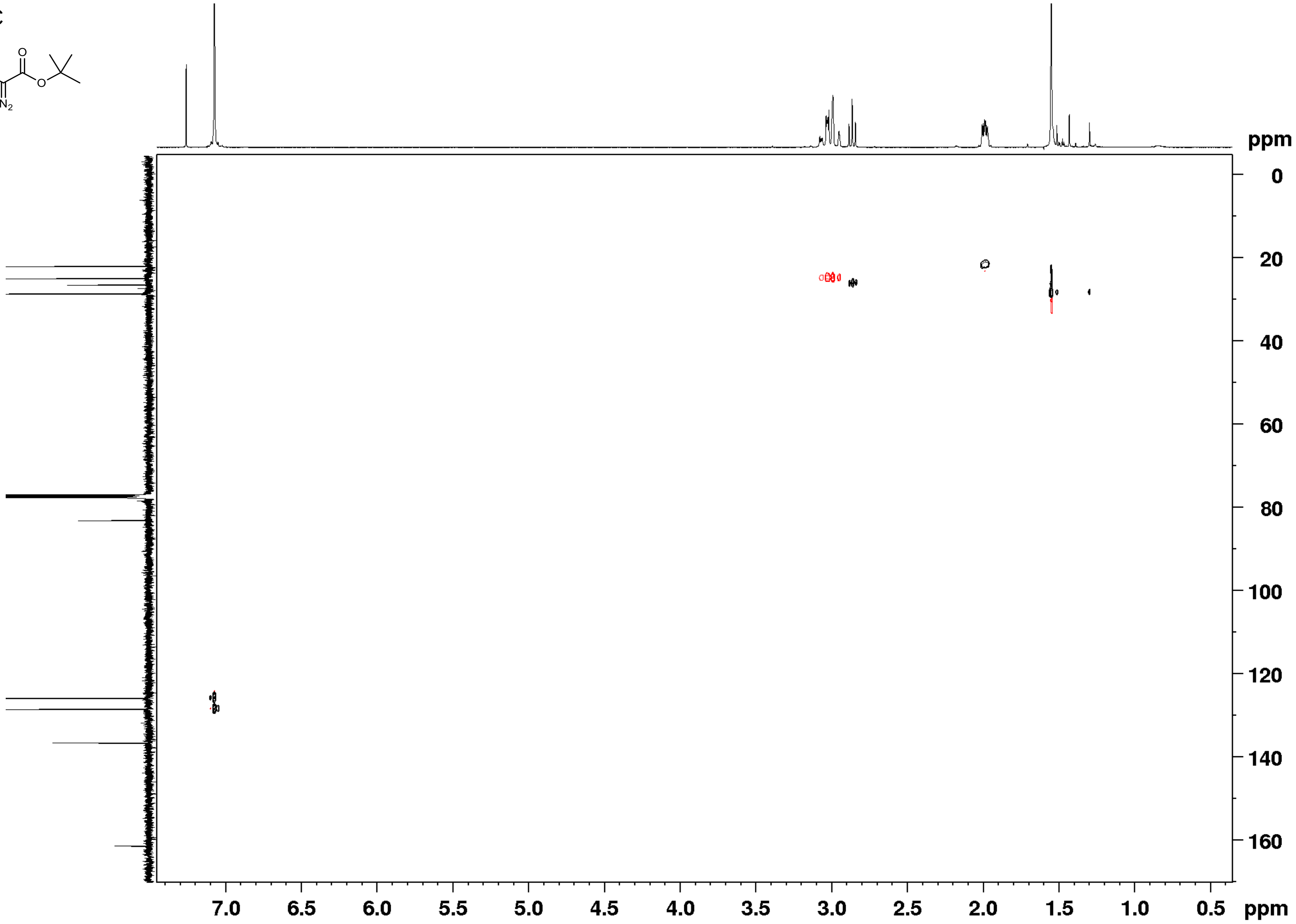

^{1}H, ^{13}C HMBC

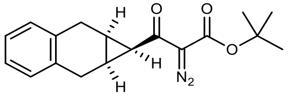

13b

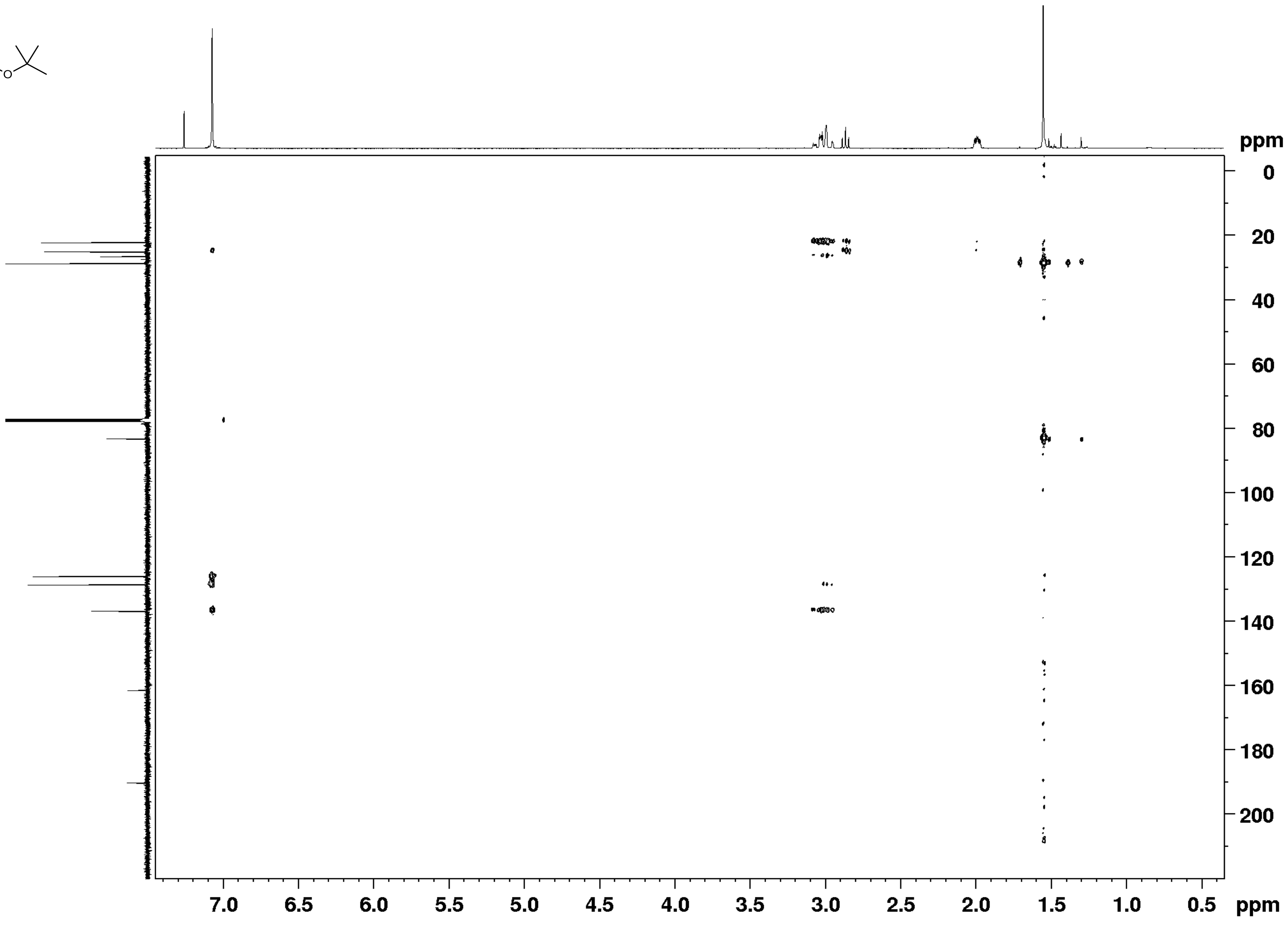

^1H, ^1H NOESY

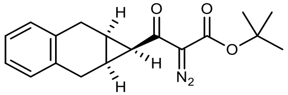

13b

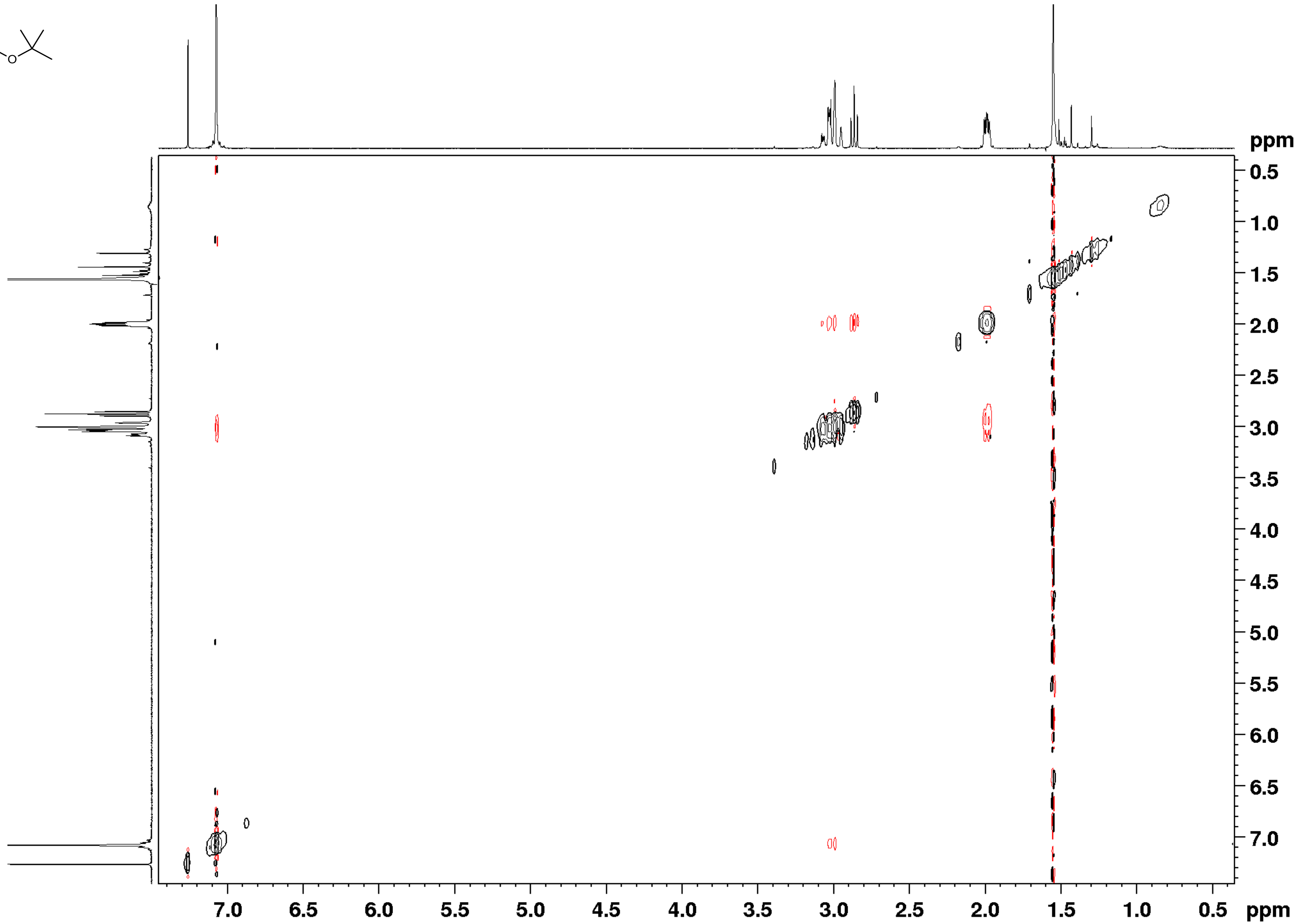

HRMS

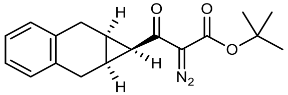

13b

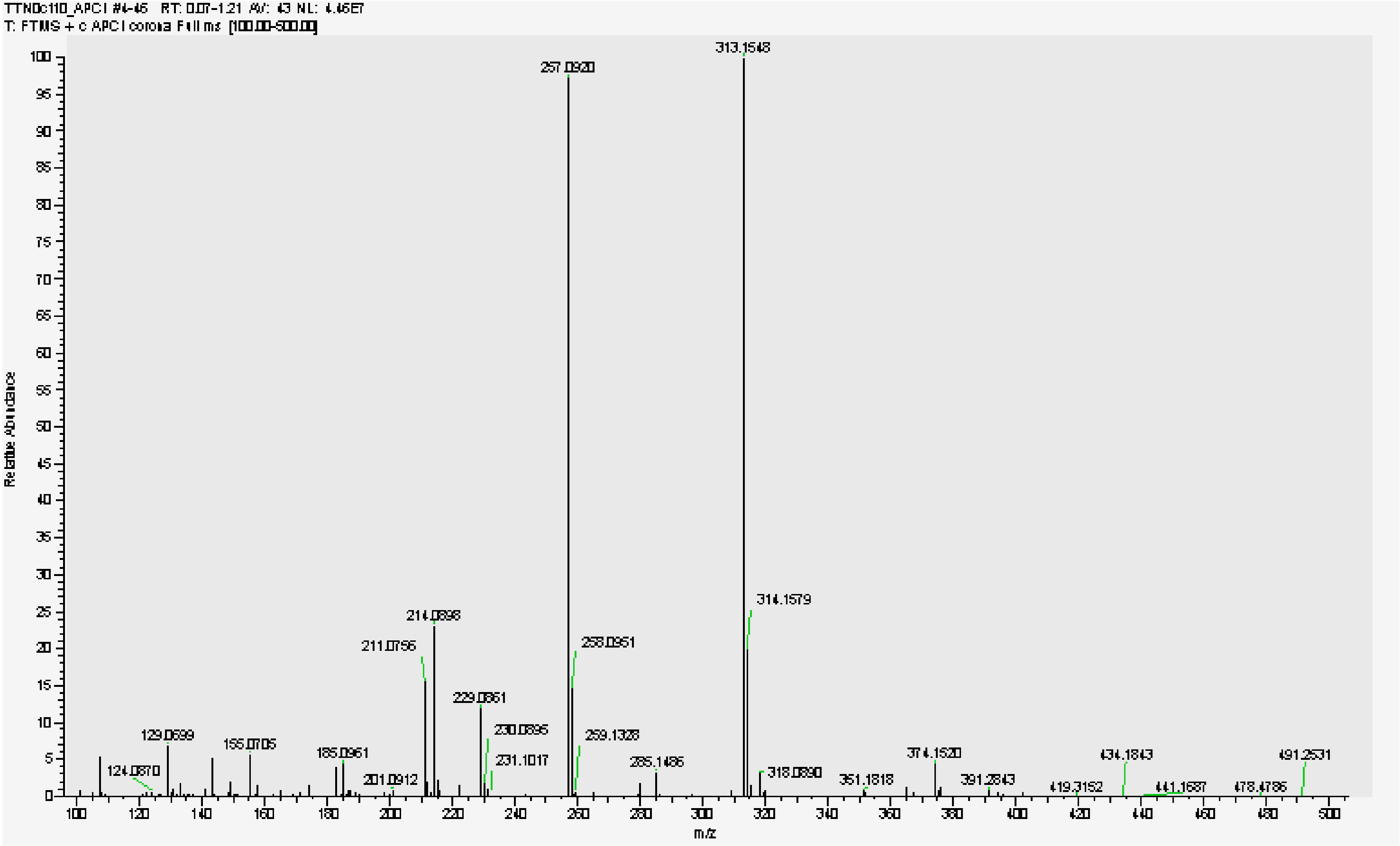
TTNDc110_APCI #4-45 RT: 0.07-1.21 AV: 43 NL: 4.46E7
T: FTMS + c APCI corona Full ms [100.00-500.00]
Relative Abundance
m/z
313.1548
257.0920
314.1579
214.0898
258.0951
211.0756
229.0861
230.0895
259.1328
129.0699
155.0705
231.1017
185.0961
374.1520
434.1843
491.2531
124.0870
285.1486
318.0890
201.0912
351.1818
391.2843
419.3152
441.1687
478.4786

IR

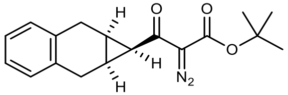
H
O
O
O
H
N2
H

13b

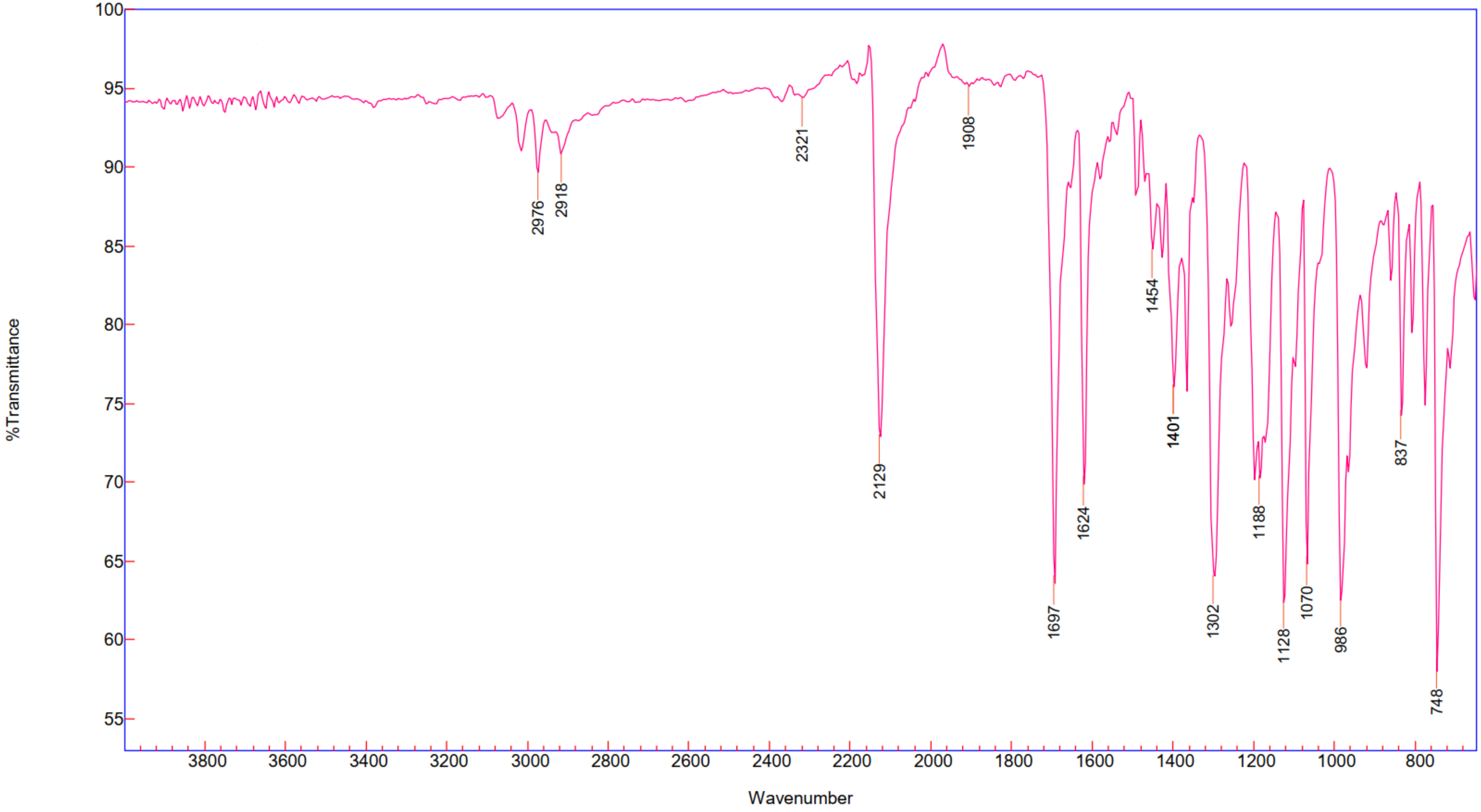
100
95
90
85
80
75
70
65
60
55
%Transmittance
2976
2918
2321
2129
1908
1697
1624
1454
1401
1302
1188
1128
1070
986
837
748
3800
3600
3400
3200
3000
2800
2600
2400
2200
2000
1800
1600
1400
1200
1000
800
Wavenumber

2.2.10 Triasterane 14a

^{1}H NMR

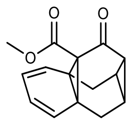

14a

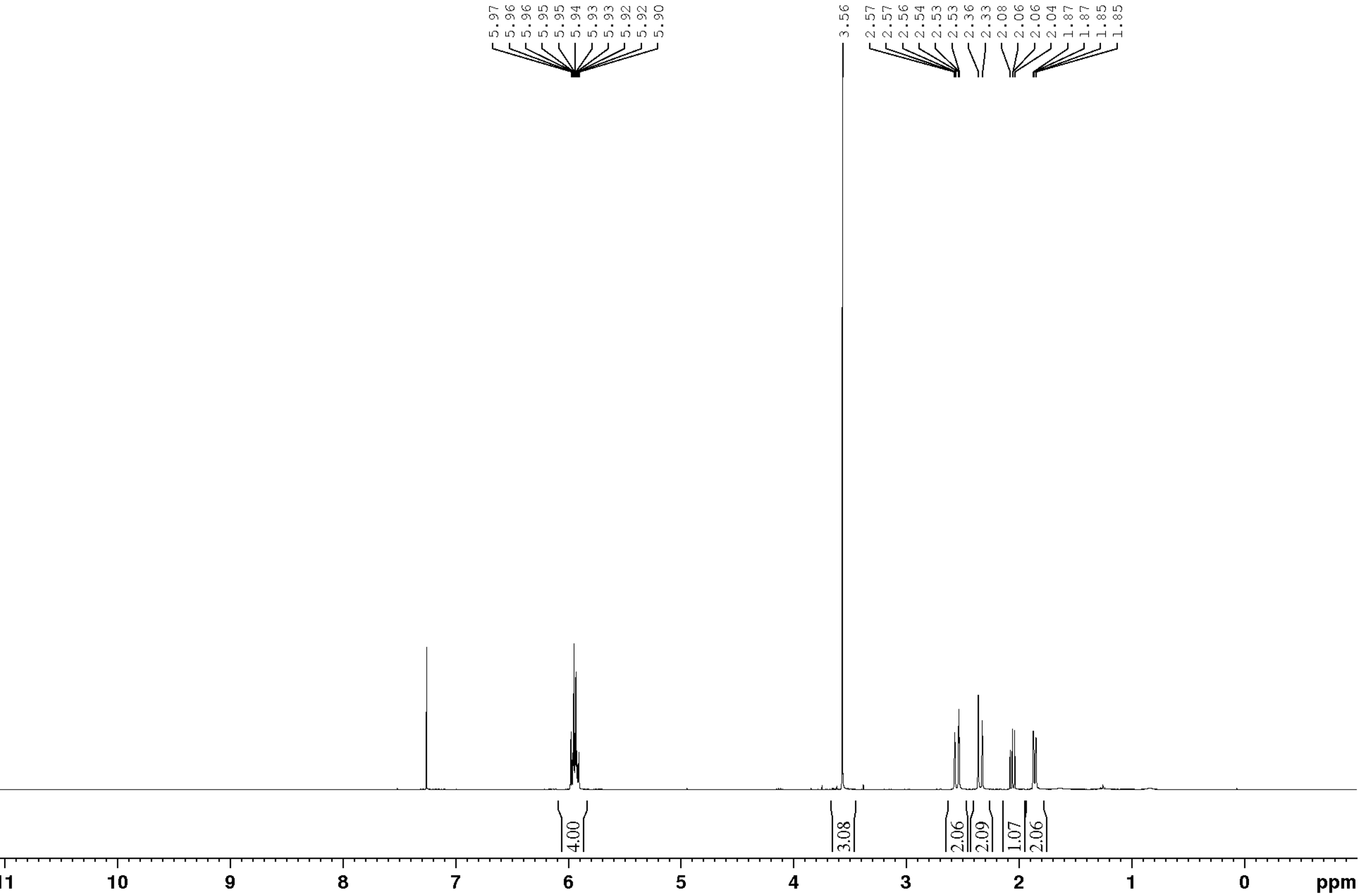

^{13}C NMR

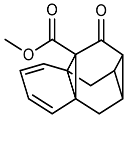

14a

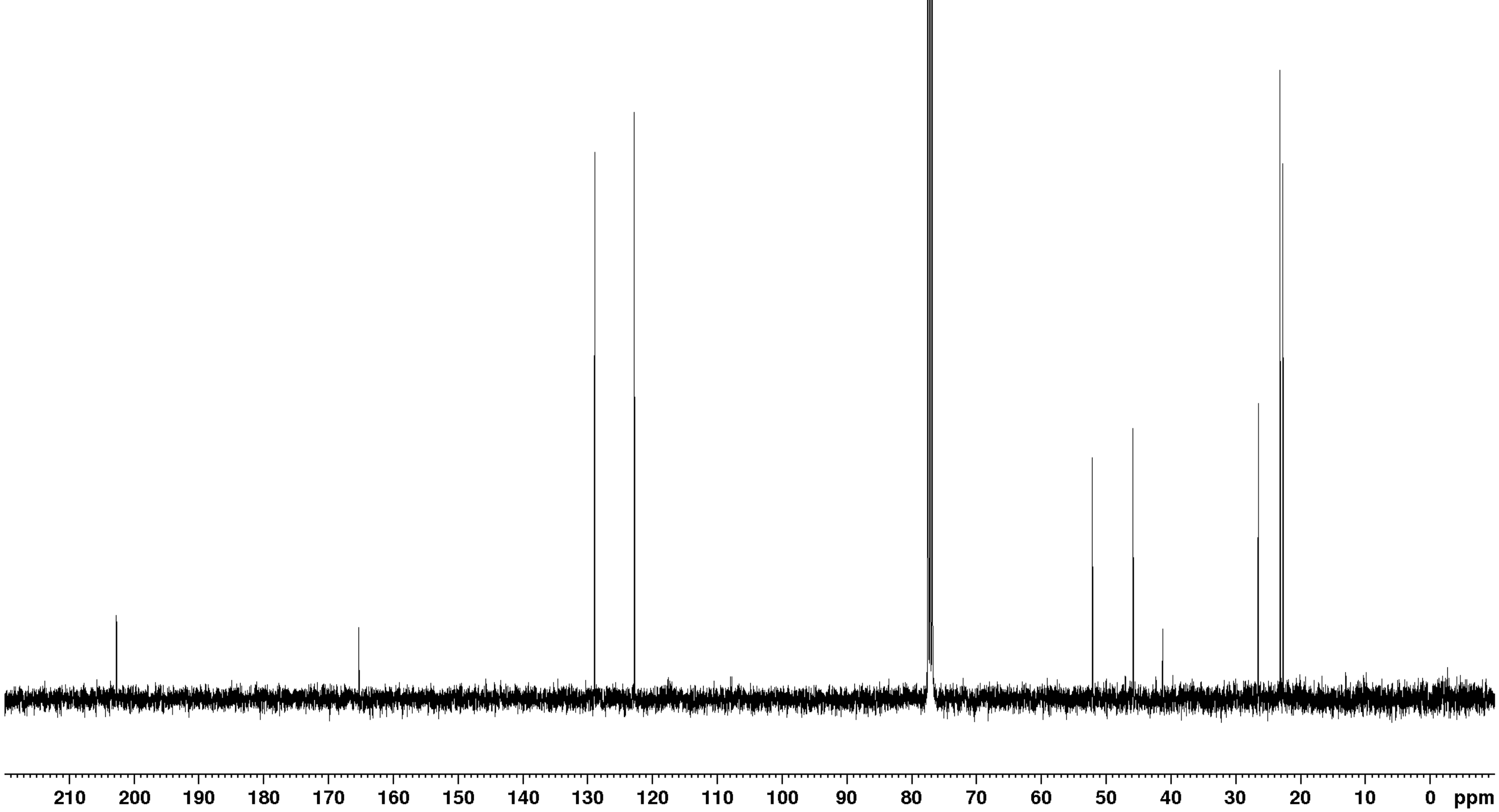
202.7
165.3
128.9
122.8
52.1
45.9
41.3
26.5
23.2
22.7
210 200 190 180 170 160 150 140 130 120 110 100 90 80 70 60 50 40 30 20 10 0 ppm

^{1}H, ^{1}H COSY

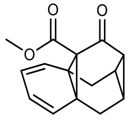

14a

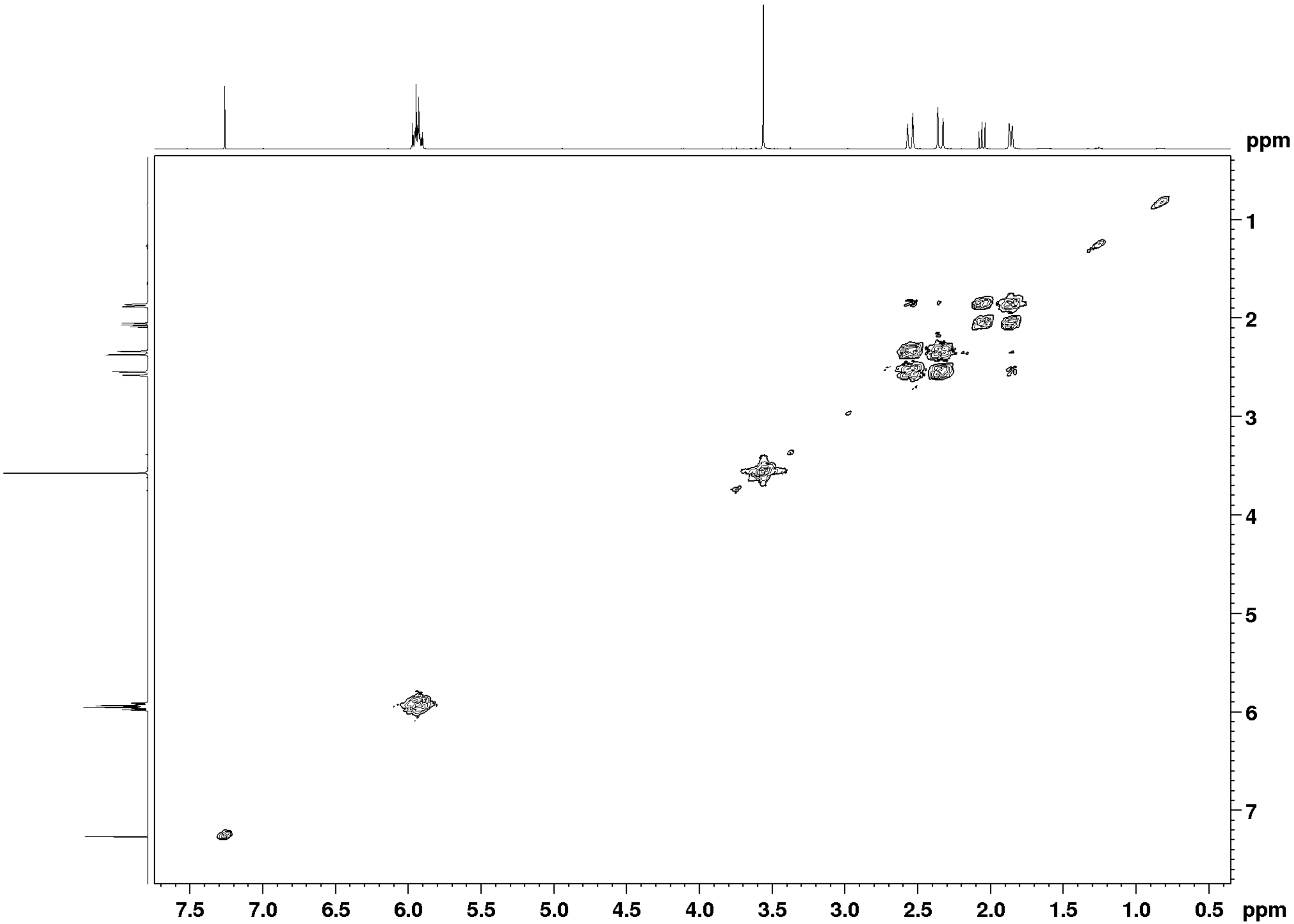

^{1}H, ^{13}C HSQC

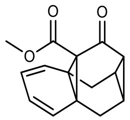

14a

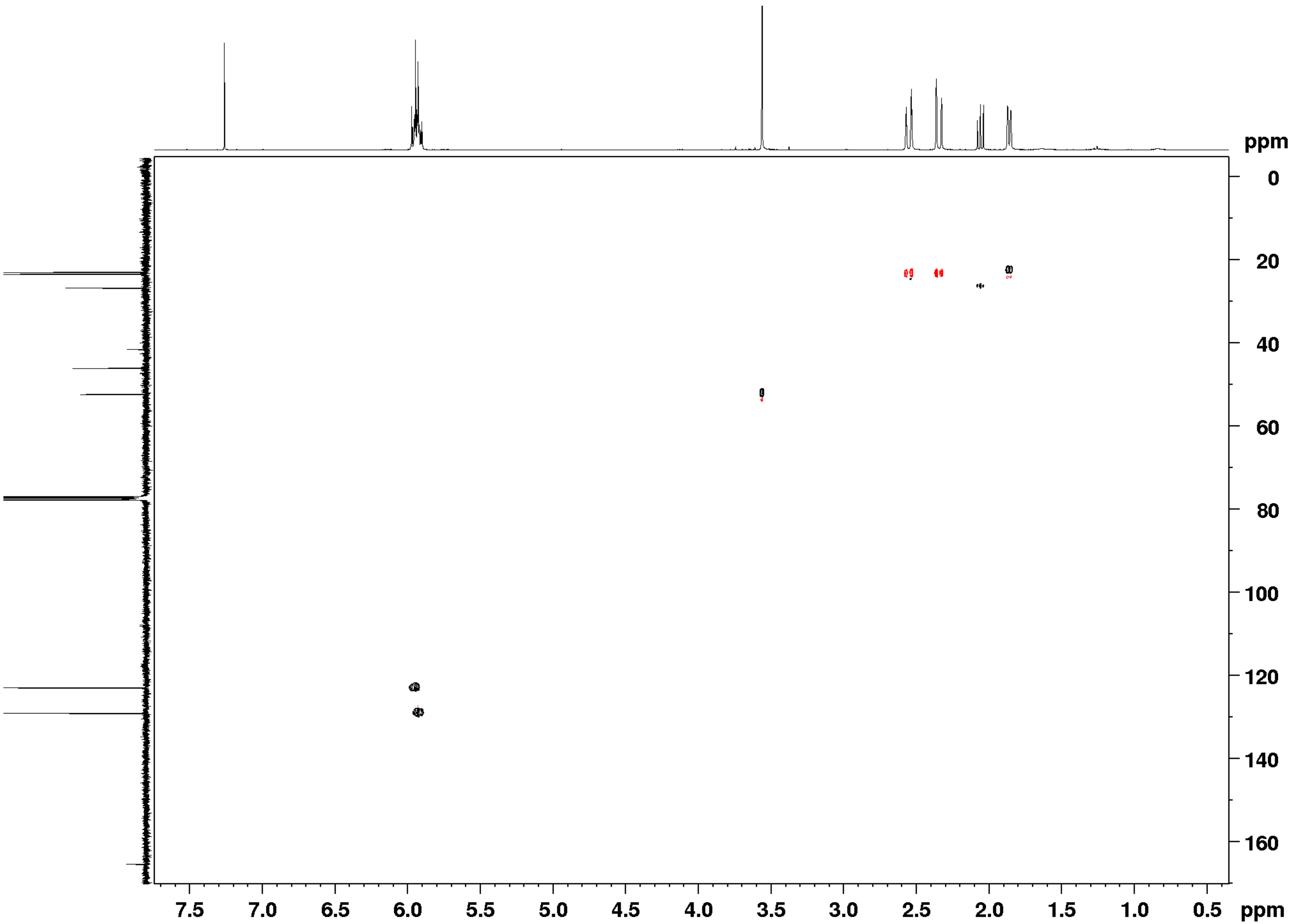

^{1}H, ^{13}C HMBC

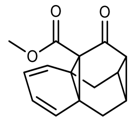

14a

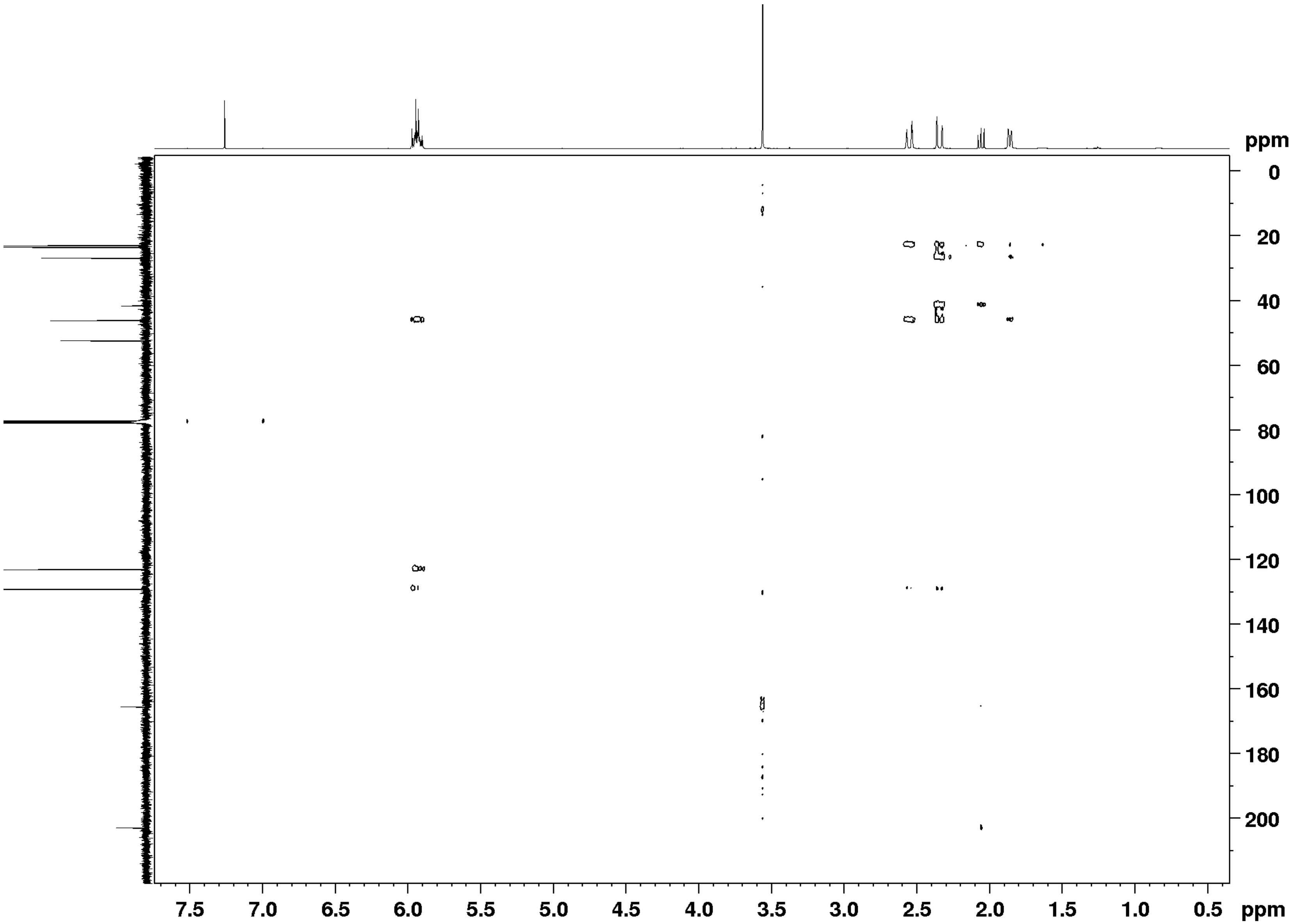

$^1H, ^1H$ NOESY

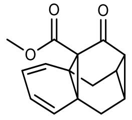

14a

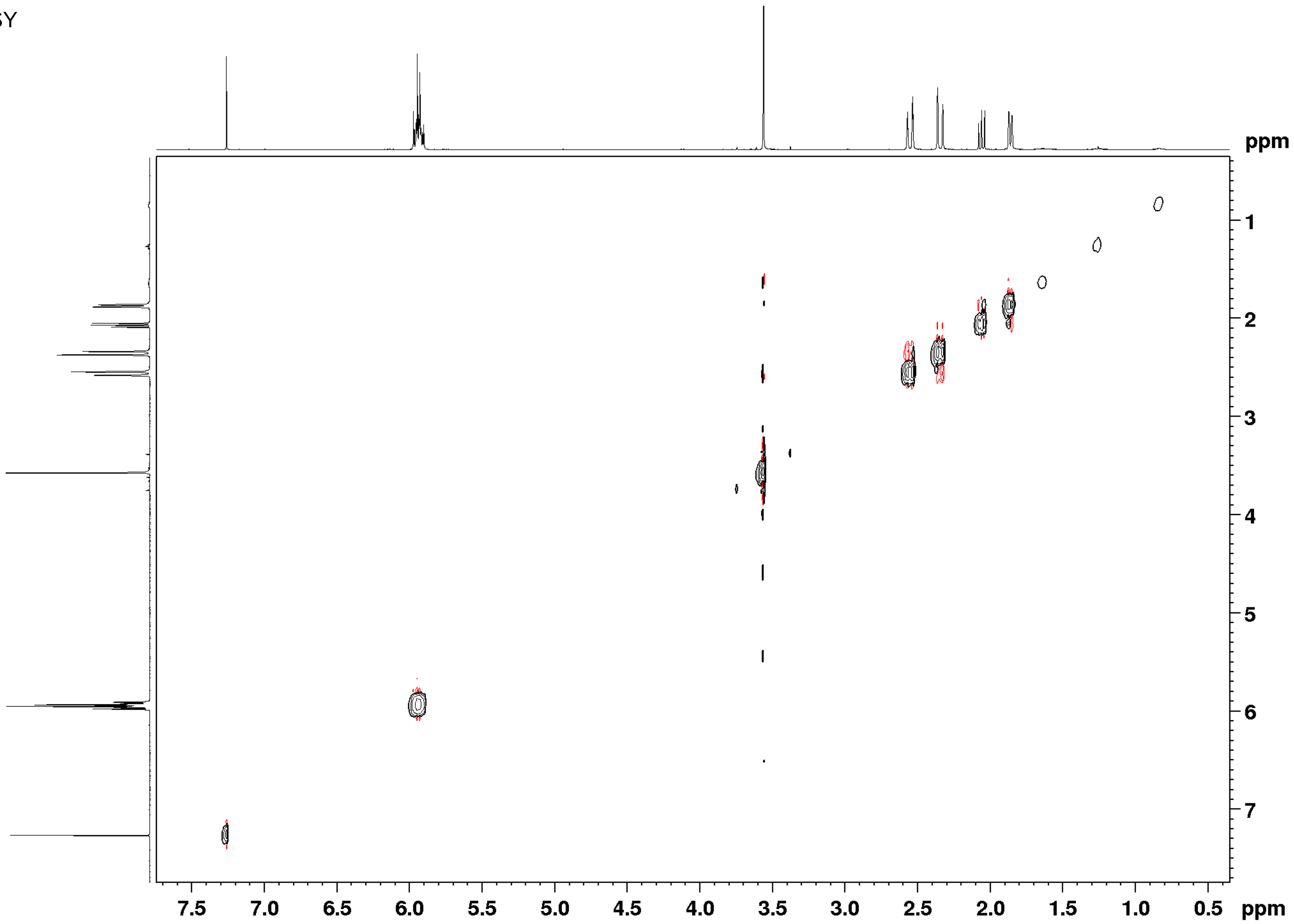

HRMS

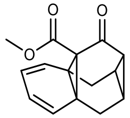
O
O
O
O

14a

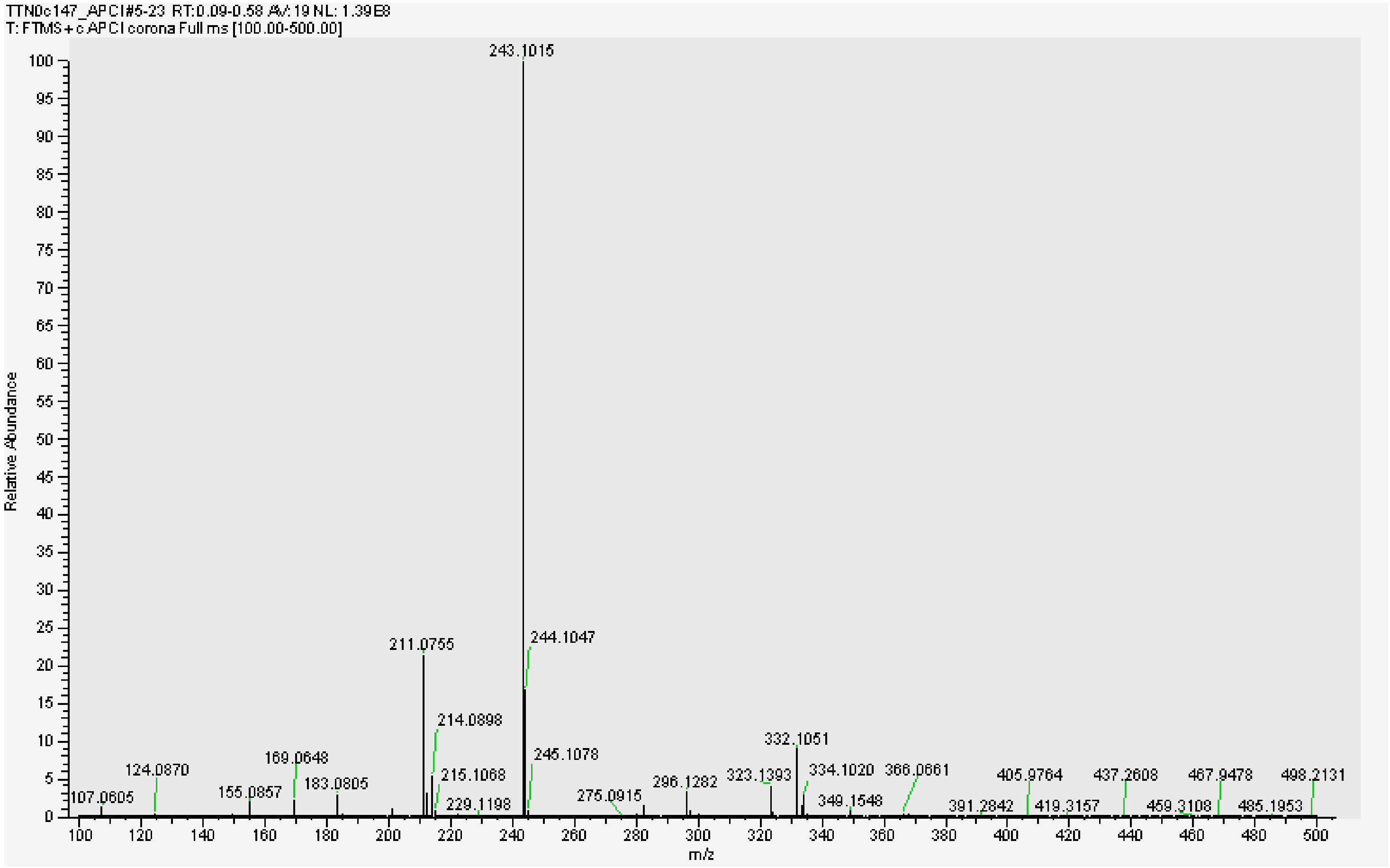
TTN0c147_APCI#5-23 RT:0.09-0.58 AV:19 NL: 1.39E8
T: FTMS + c APCI corona Full ms [100.00-500.00]
243.1015
211.0755
244.1047
214.0898
245.1078
215.1068
229.1198
107.0605
124.0870
155.0857
169.0648
183.0805
275.0915
296.1282
323.1393
332.1051
334.1020
349.1548
366.0661
391.2842
405.9764
419.3157
437.2608
459.3108
467.9478
485.1953
498.2131
Relative Abundance
m/z

IR

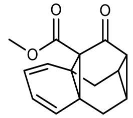

14a

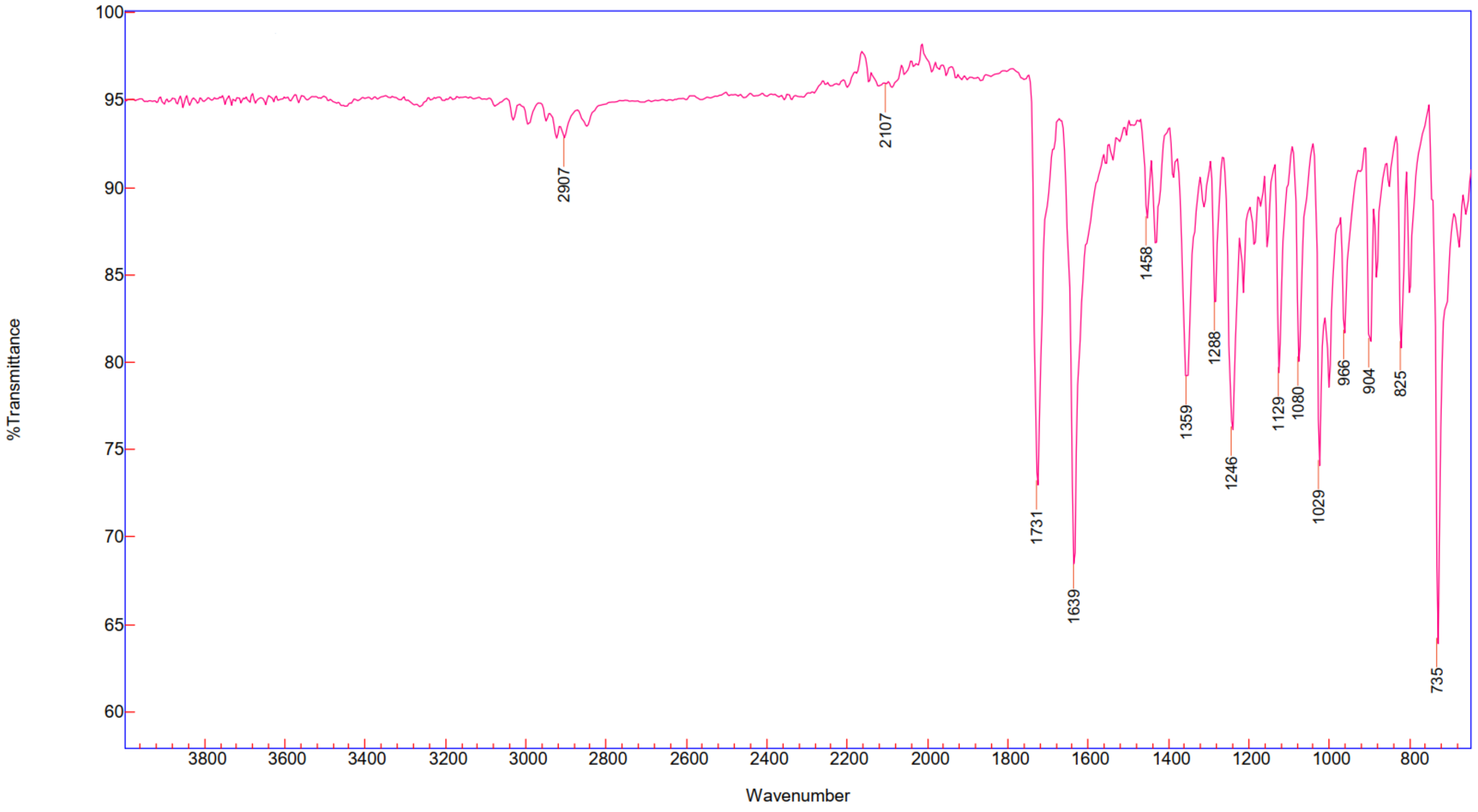

%Transmittance
Wavenumber
100
95
90
85
80
75
70
65
60
3800
3600
3400
3200
3000
2800
2600
2400
2200
2000
1800
1600
1400
1200
1000
800
2907
2107
1731
1639
1458
1359
1288
1246
1129
1080
1029
966
904
825
735

2.2.11 Triasterane 14b

^{1}H NMR

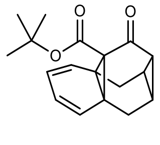

14b

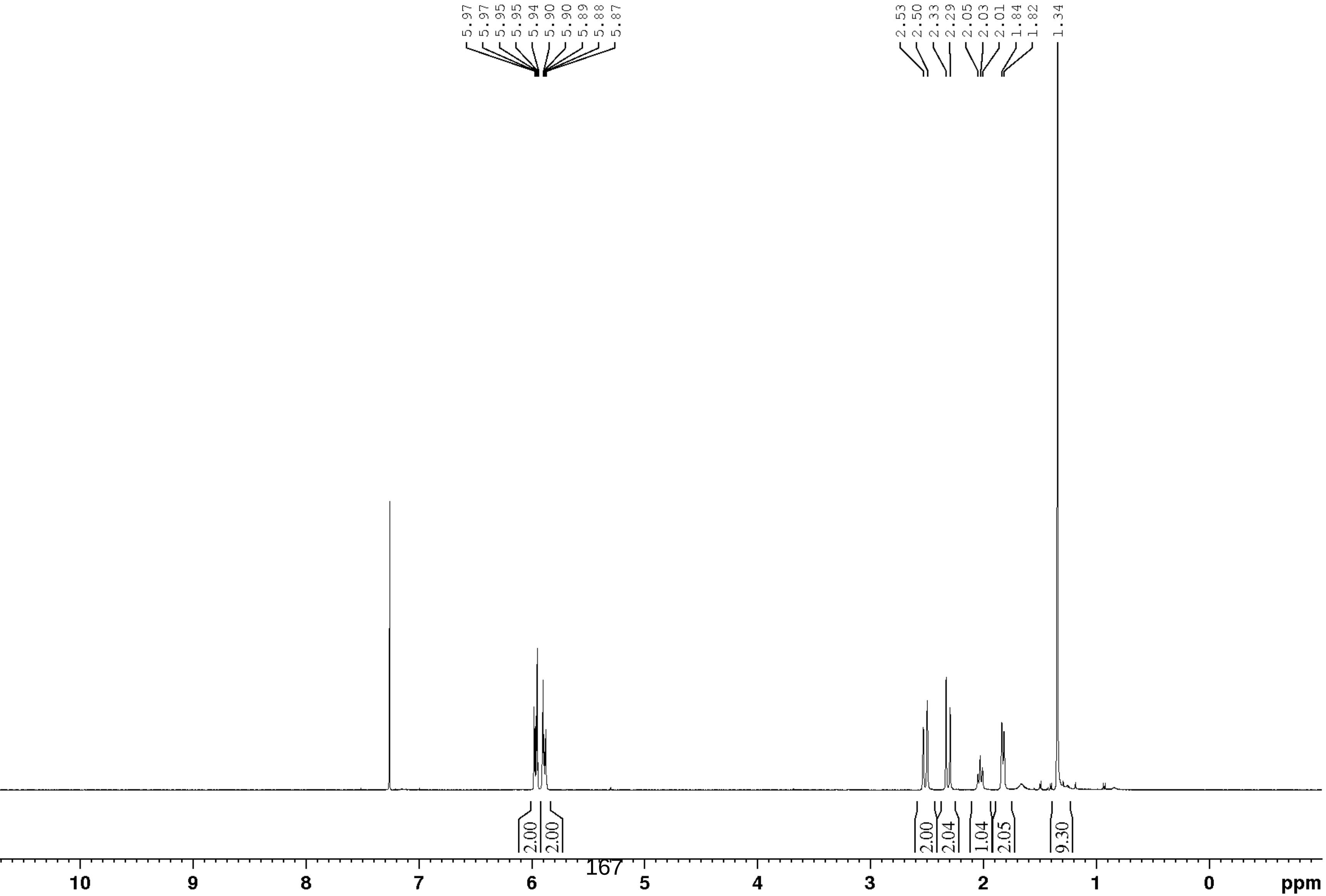

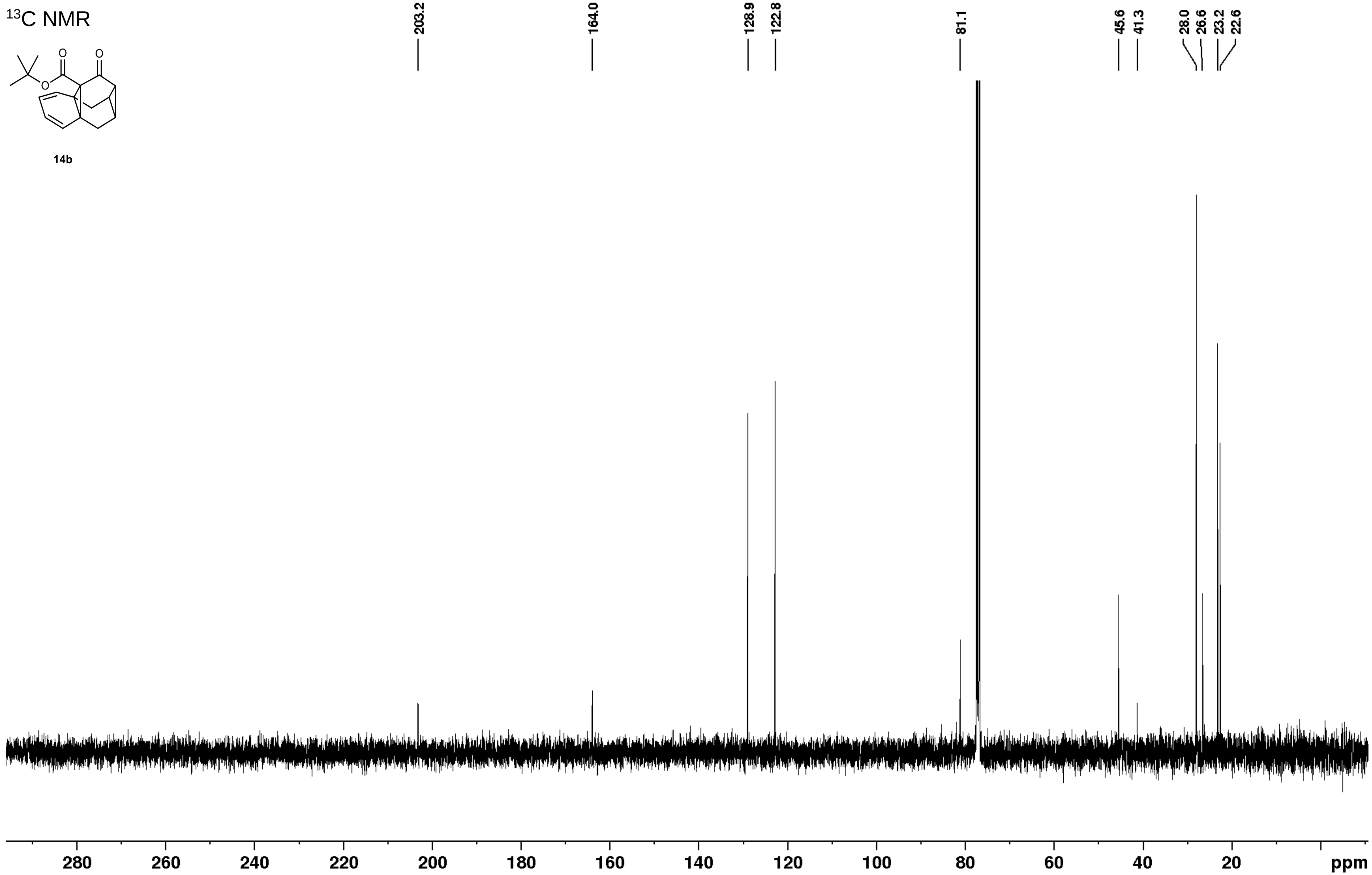

^{13}C NMR
14b
203.2
164.0
128.9
122.8
81.1
45.6
41.3
28.0
26.6
23.2
22.6
280
260
240
220
200
180
160
140
120
100
80
60
40
20
ppm

^1H, ^1H COSY

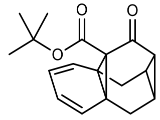

14b

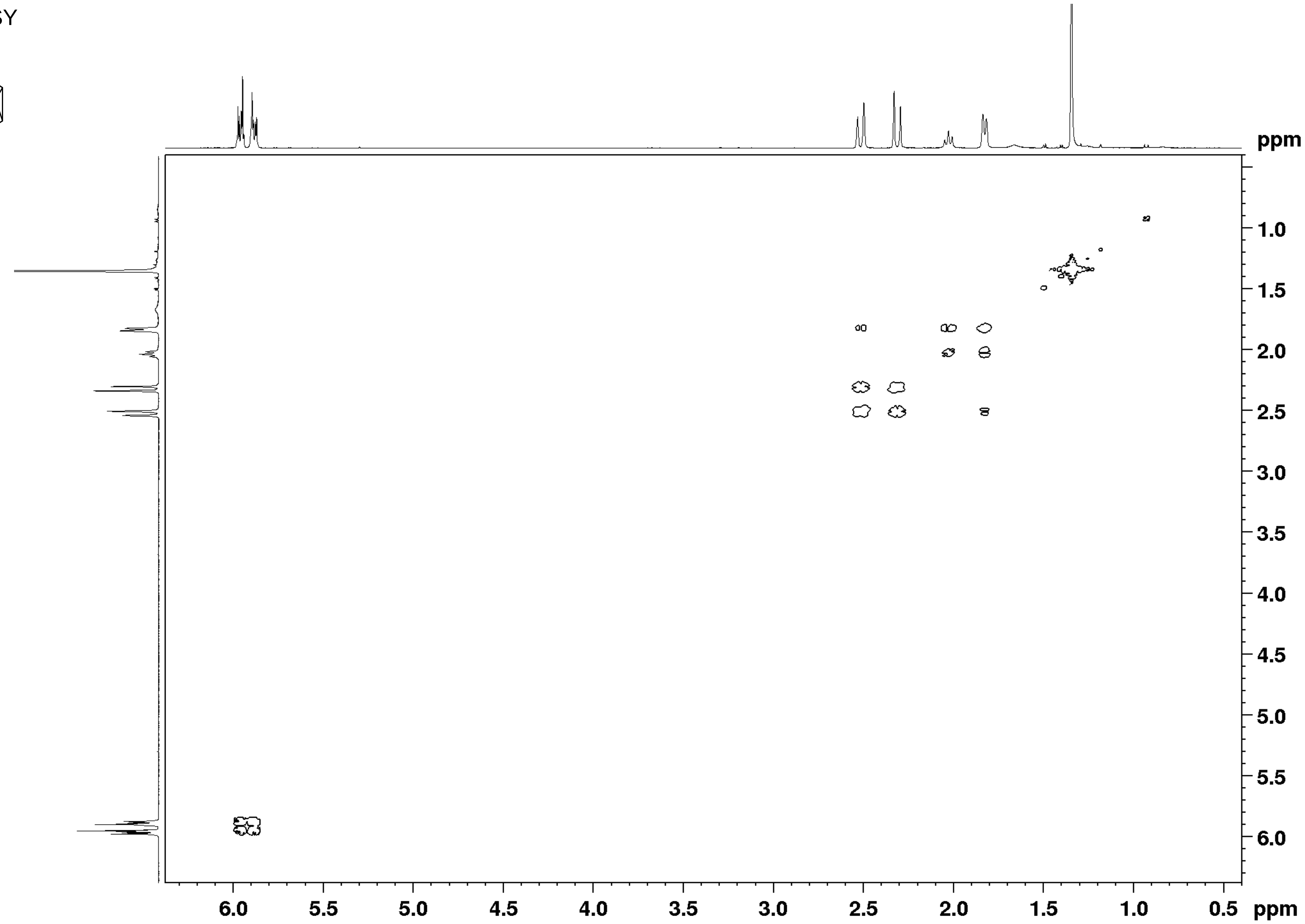

^{1}H, ^{13}C HSQC

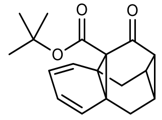

14b

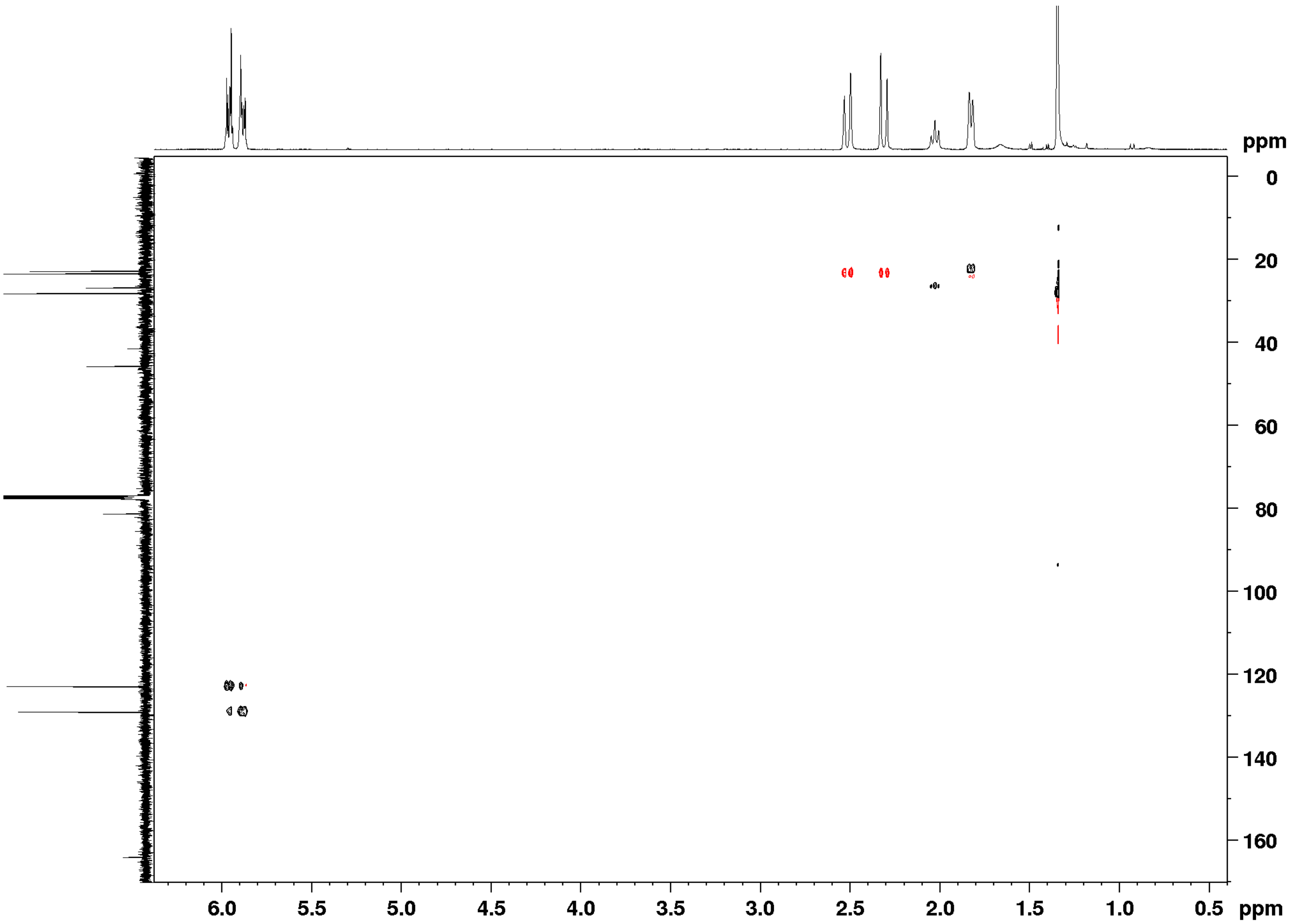

^1H, ^{13}C HMBC

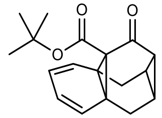

14b

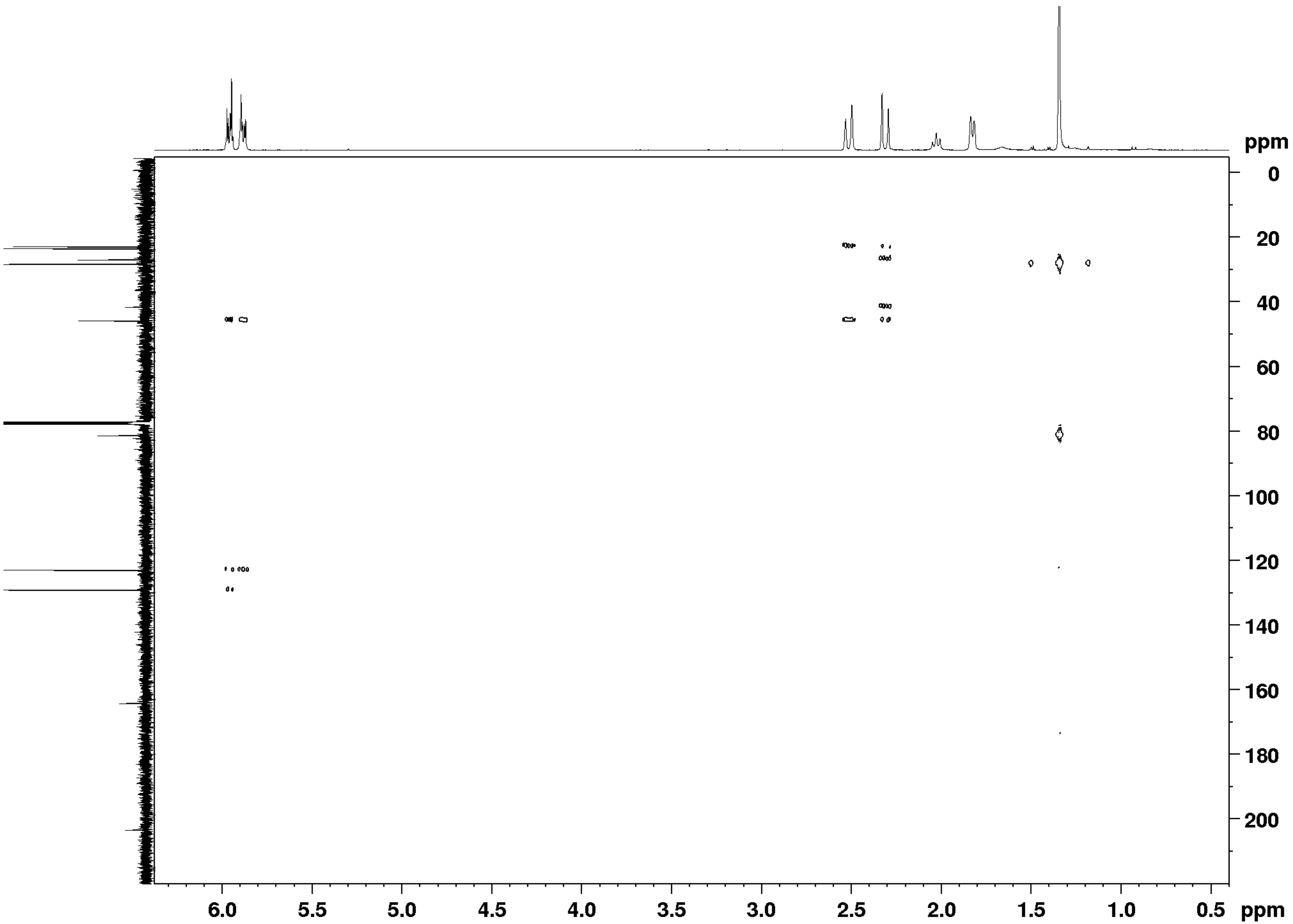

^{1}H, ^{1}H NOESY

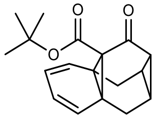

14b

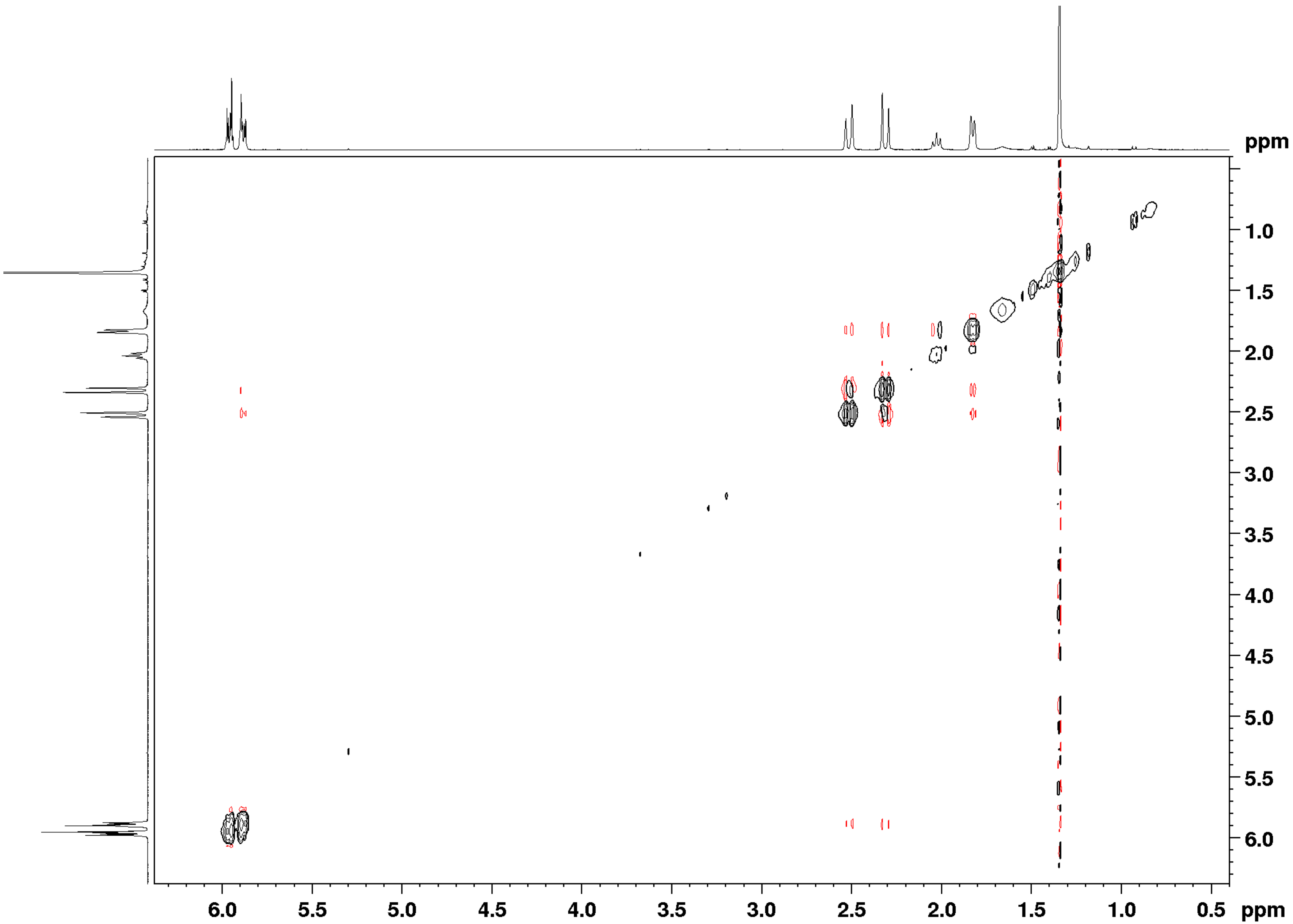

HRMS

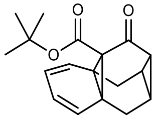

O
O
O

14b

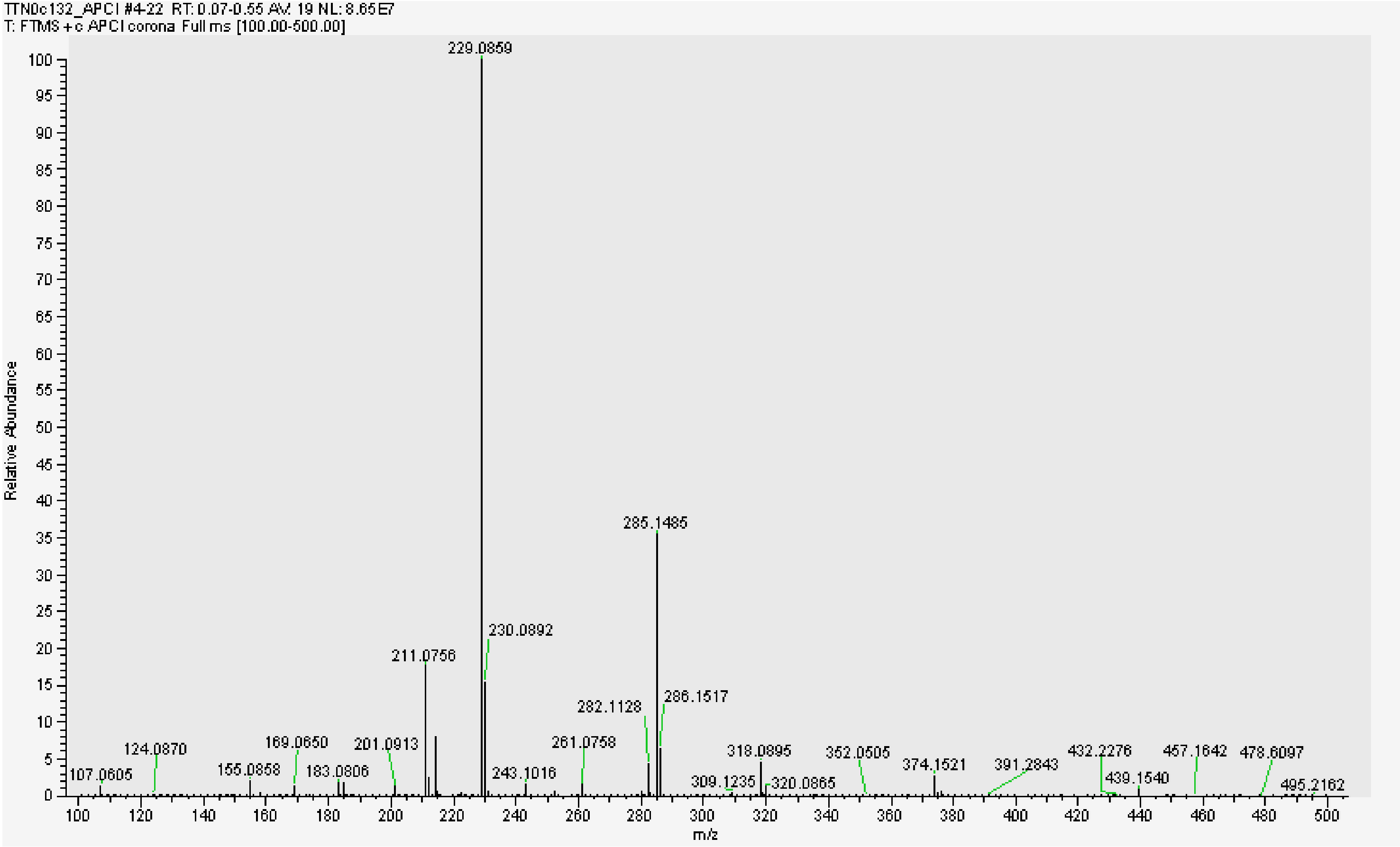

TTN0c132_APCI #4-22 RT: 0.07-0.55 AV: 19 NL: 8.65E7
T: FTMS + c APCI corona Full ms [100.00-500.00]
229.0859
285.1485
230.0892
211.0756
286.1517
282.1128
261.0758
201.0913
169.0650
124.0870
155.0858
183.0806
107.0605
243.1016
318.0895
352.0505
374.1521
391.2843
432.2276
457.1642
478.6097
439.1540
309.1235
320.0865
495.2162
Relative Abundance
m/z

IR

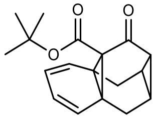

14b

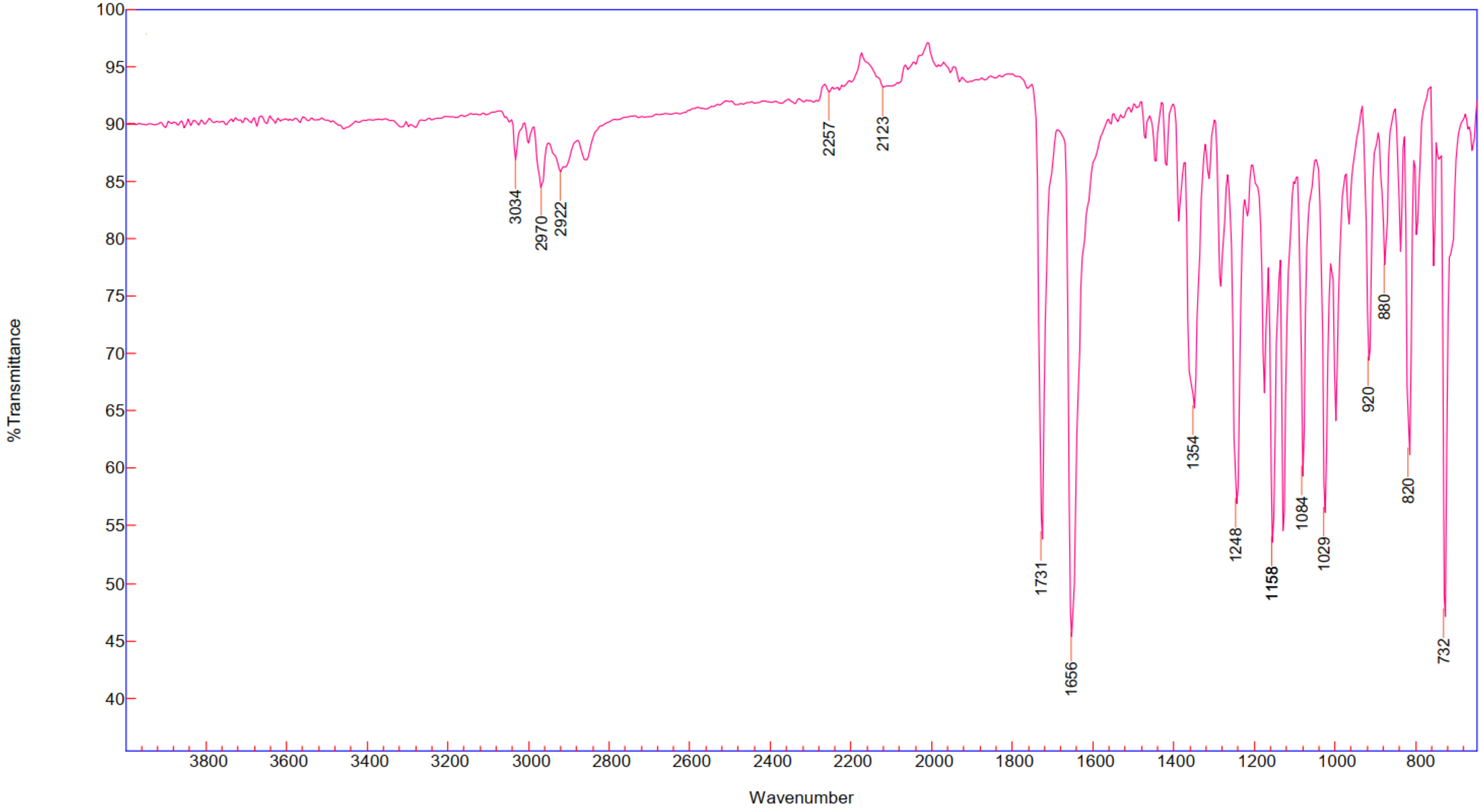

100
95
90
85
80
75
70
65
60
55
50
45
40
% Transmittance
3034
2970
2922
2257
2123
1731
1656
1354
1248
1158
1084
1029
920
880
820
732
3800
3600
3400
3200
3000
2800
2600
2400
2200
2000
1800
1600
1400
1200
1000
800
Wavenumber

2.2.12 Methyl 1-oxo-1,2-dihydro-9a*H*-2,9-ethenobenzo[7]annulene-9a-carboxylate (15)

^{1}H NMR

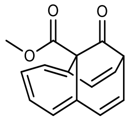

15

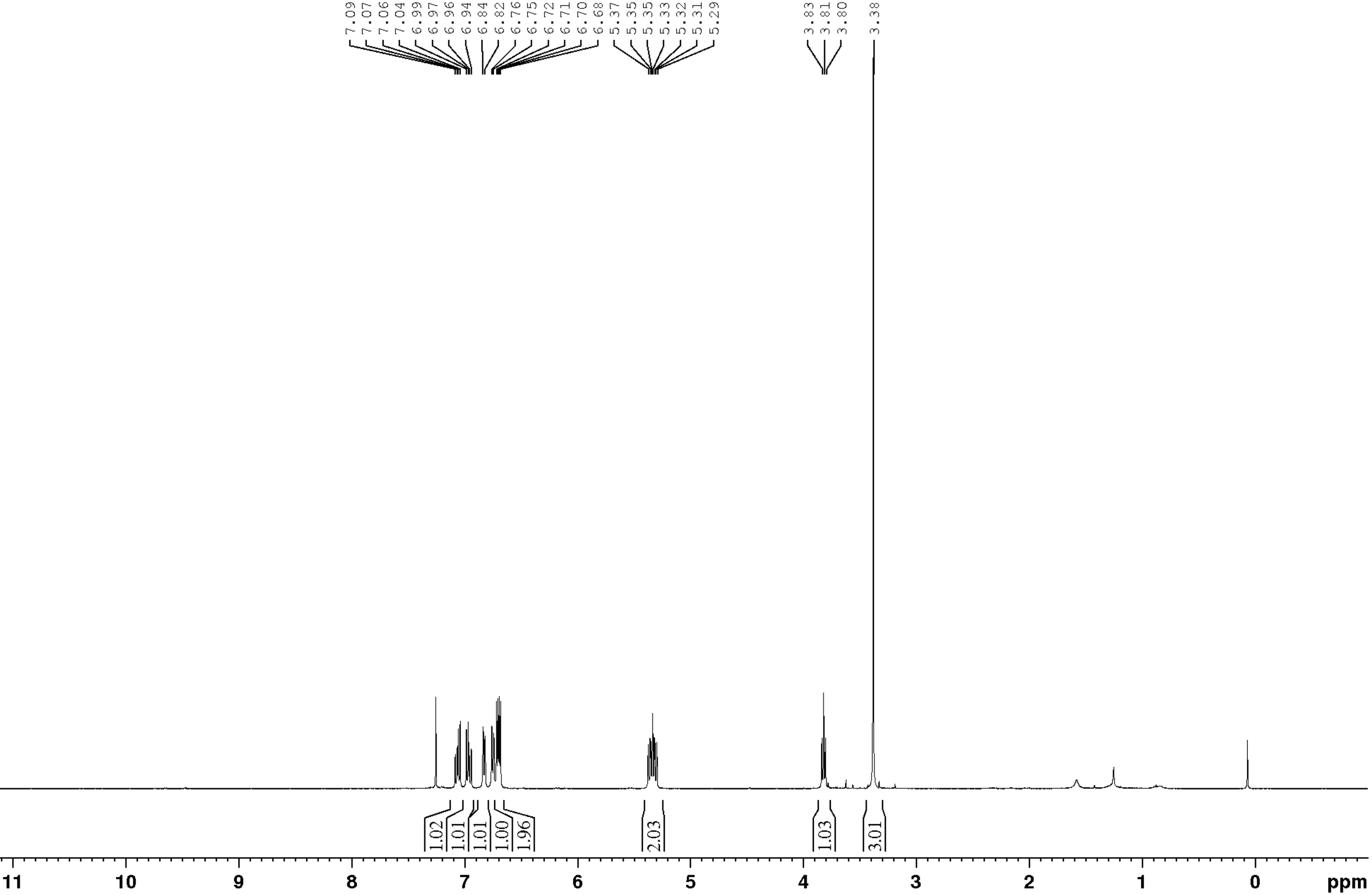

^{13}C NMR

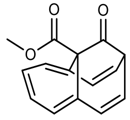

15

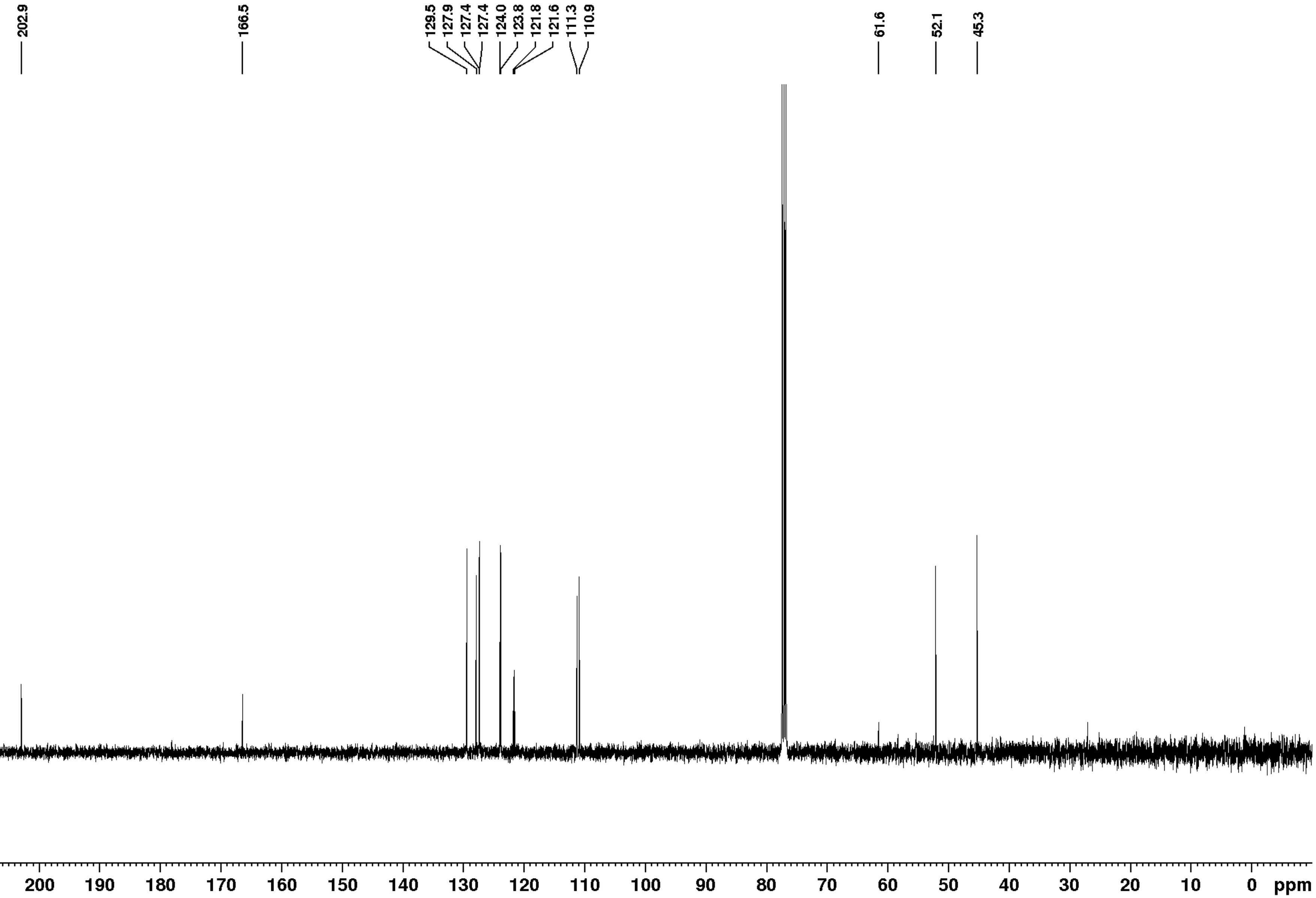

202.9
166.5
129.5
127.9
127.4
127.4
124.0
123.8
121.8
121.6
111.3
110.9
61.6
52.1
45.3
210 200 190 180 170 160 150 140 130 120 110 100 90 80 70 60 50 40 30 20 10 0 ppm

^1H, ^1H COSY

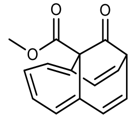

15

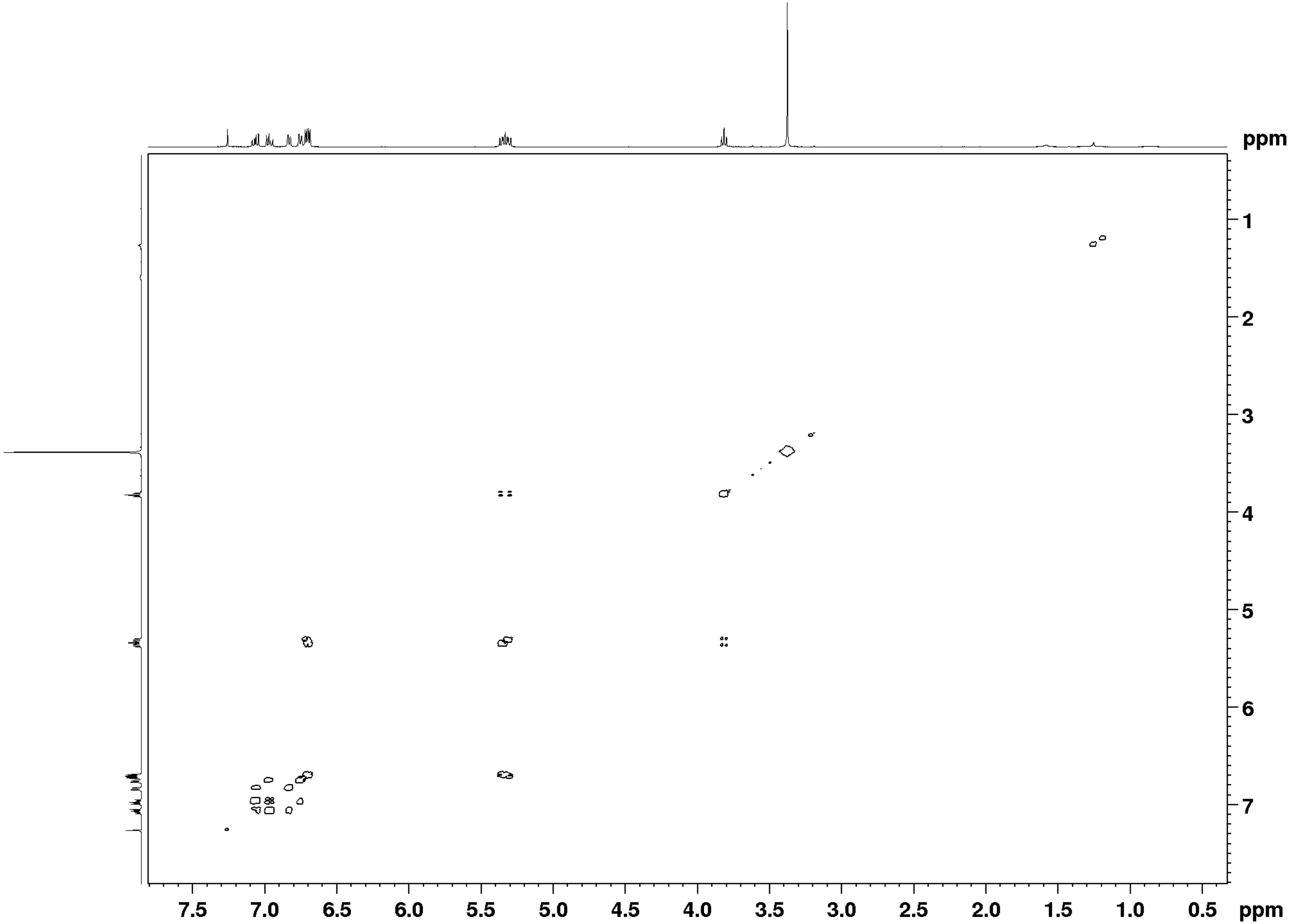

^{1}H, ^{13}C HSQC

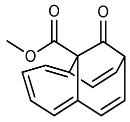

15

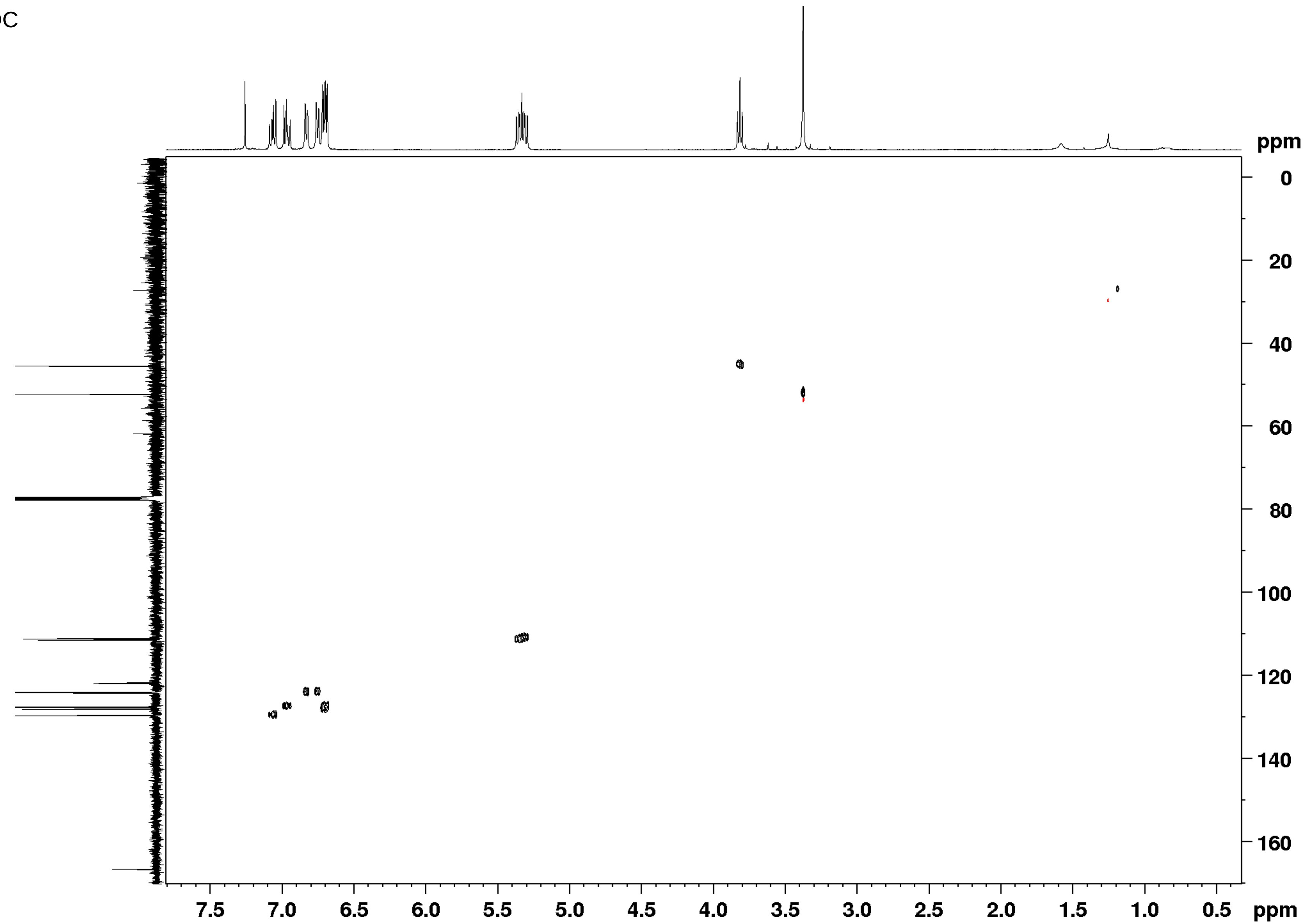

^{1}H, ^{13}C HMBC

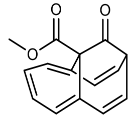

15

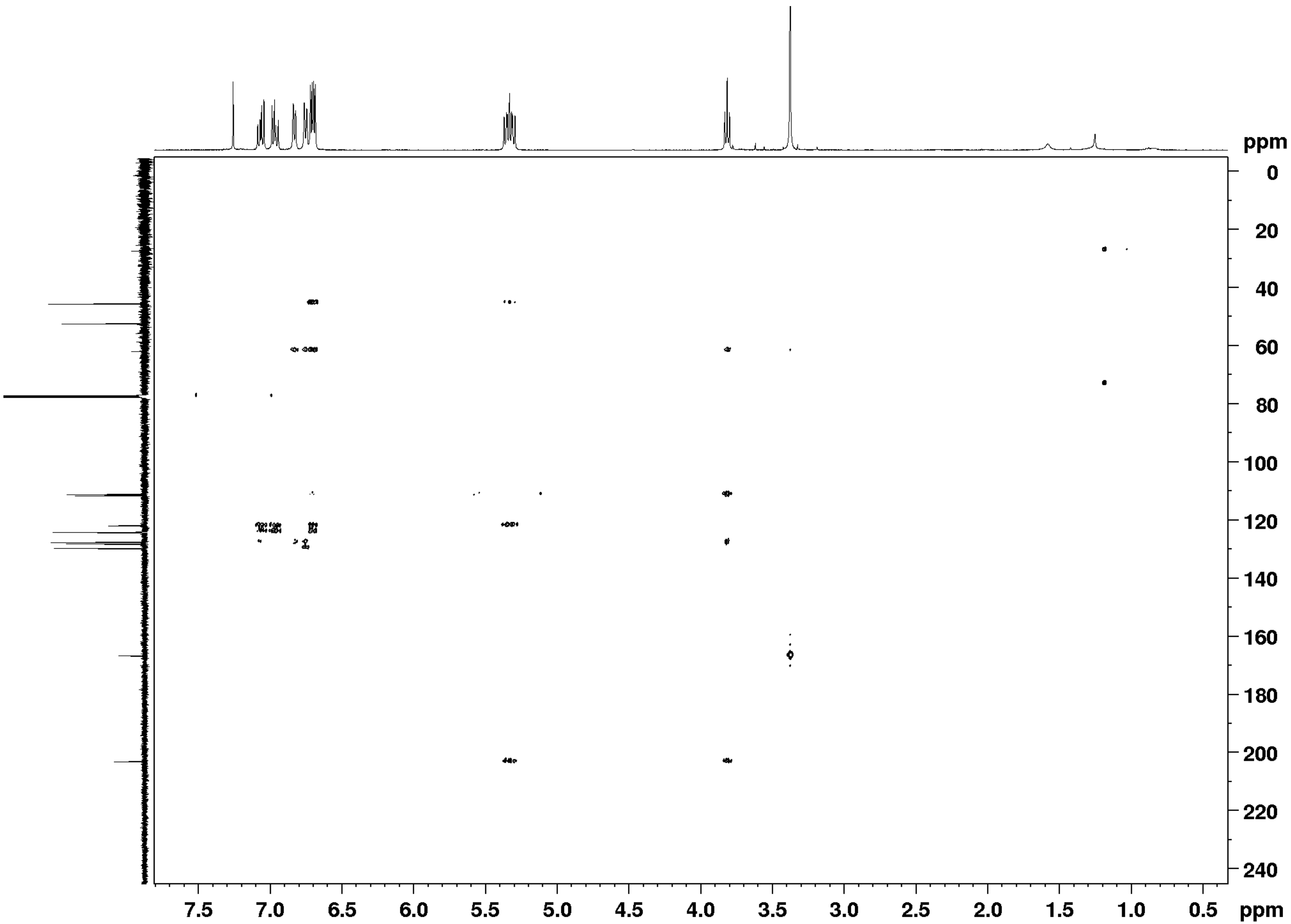

^1H, ^1H NOESY

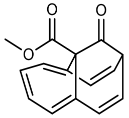

15

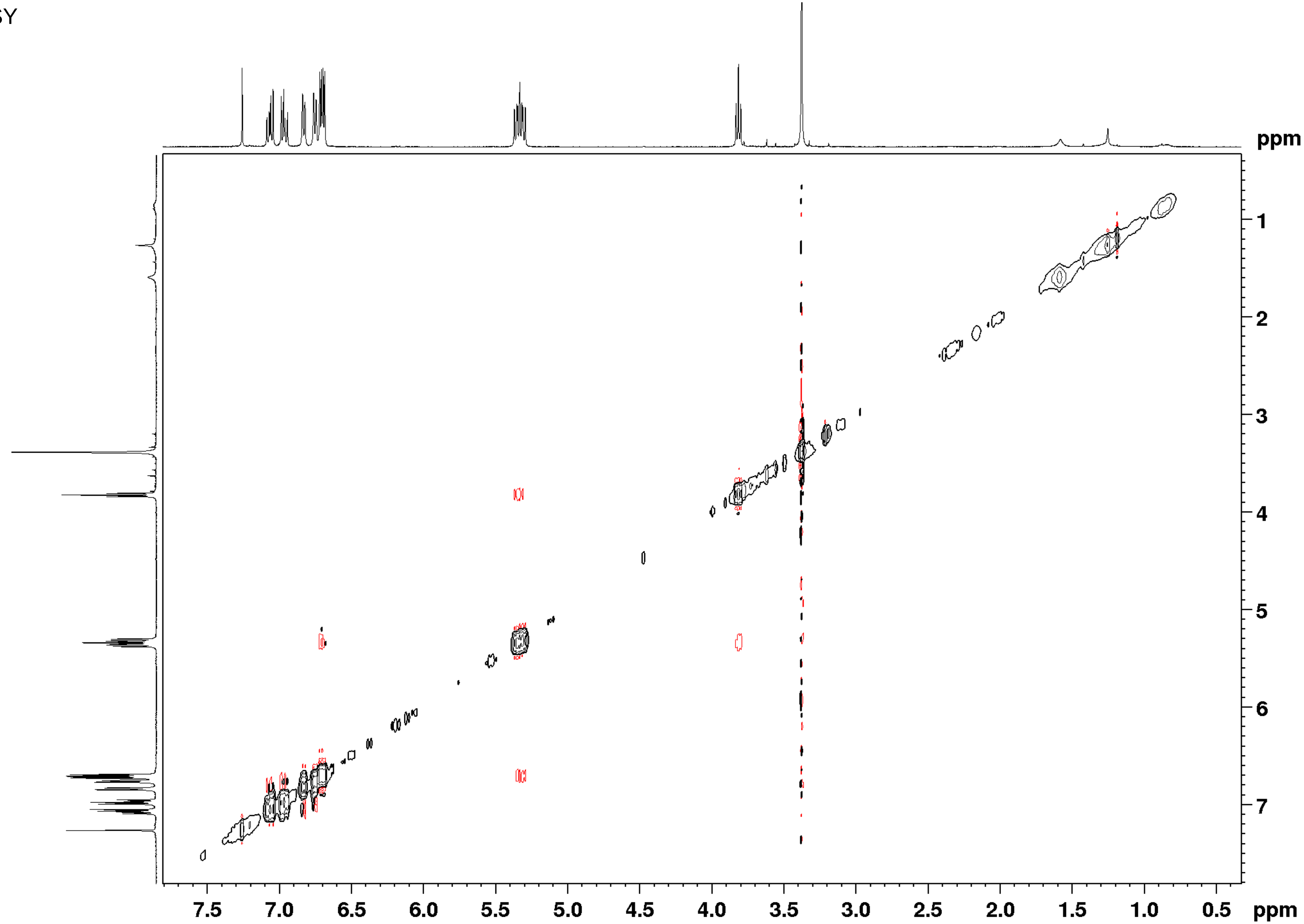

HRMS

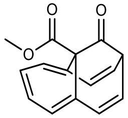

15

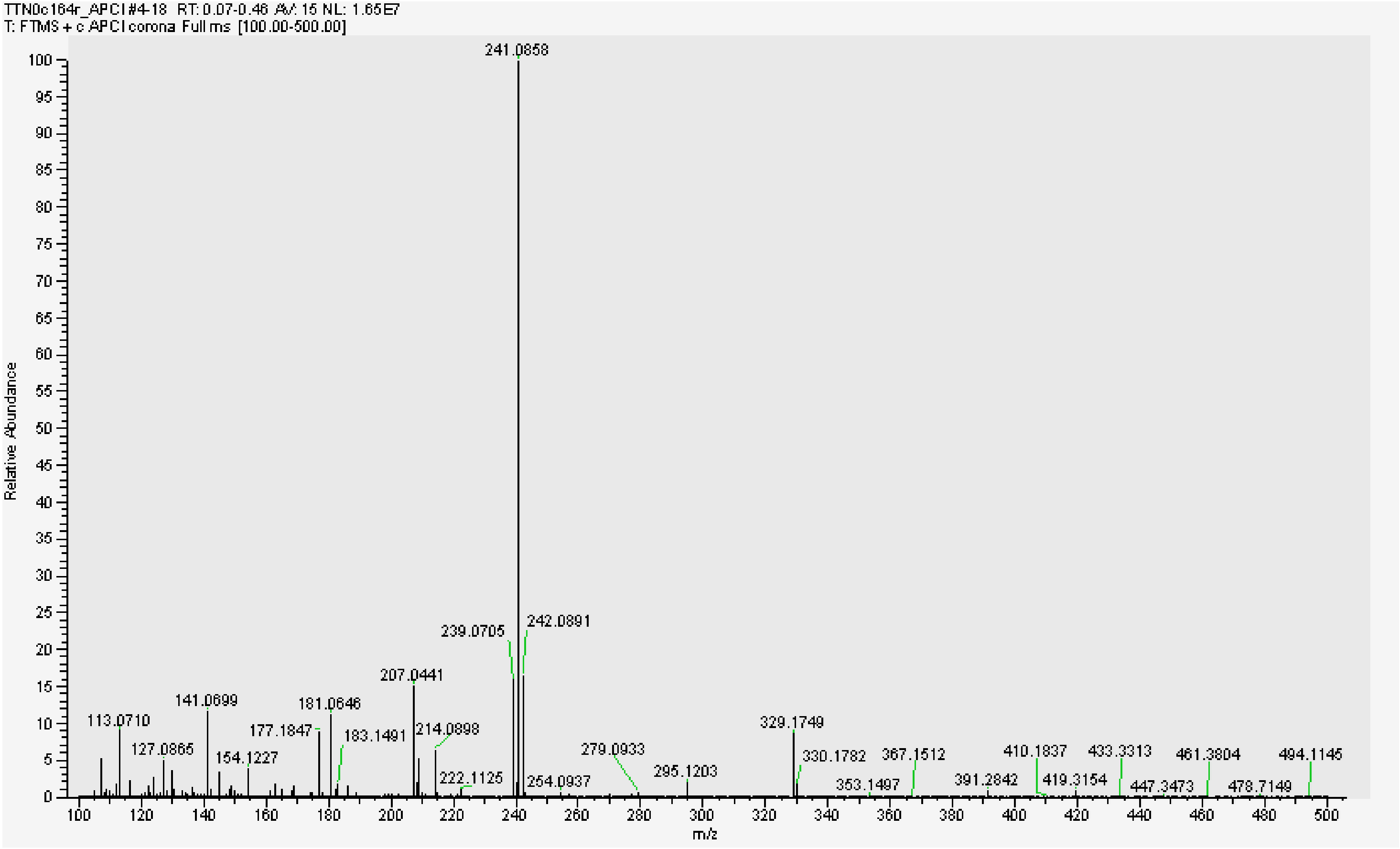

TTN0c164r_APCI #4-18 RT: 0.07-0.46 AV: 15 NL: 1.65E7
T: FTMS + c APCI corona Full ms [100.00-500.00]
241.0858
242.0891
239.0705
207.0441
181.0646
141.0699
113.0710
177.1847
183.1491
214.0898
127.0865
154.1227
222.1125
254.0937
279.0933
295.1203
329.1749
330.1782
353.1497
367.1512
391.2842
410.1837
419.3154
433.3313
447.3473
461.3804
478.7149
494.1145
Relative Abundance
m/z

IR

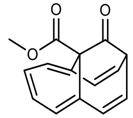

15

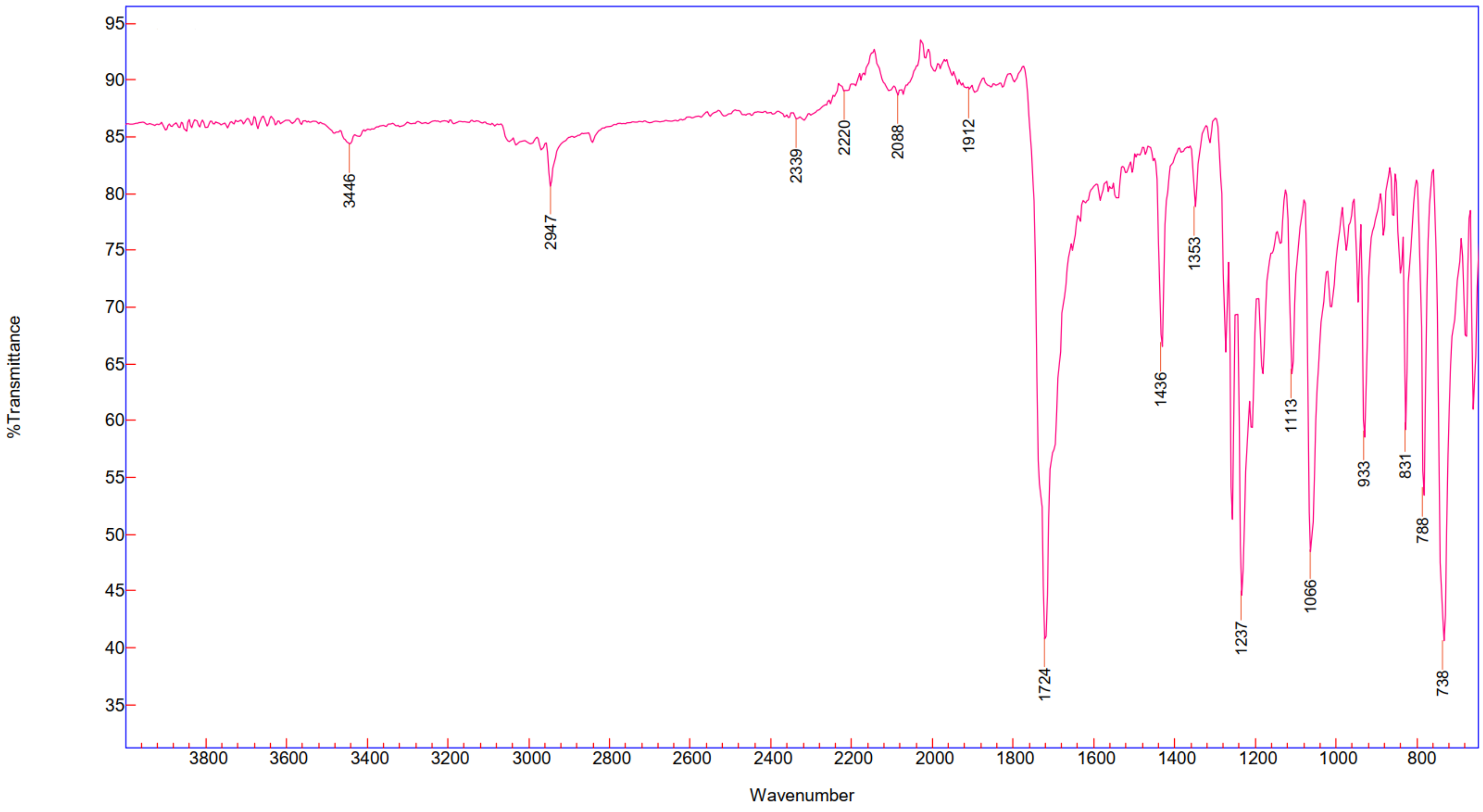
%Transmittance
Wavenumber
3446
2947
2339
2220
2088
1912
1724
1436
1353
1237
1113
1066
933
831
788
738

2.2.13 1-Oxo-1,9a-dihydro-2-2,9-ethenobenzo[7]annulene-2-carboxylic acid (16)

^{1}H NMR

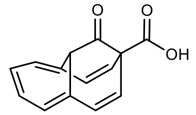

16

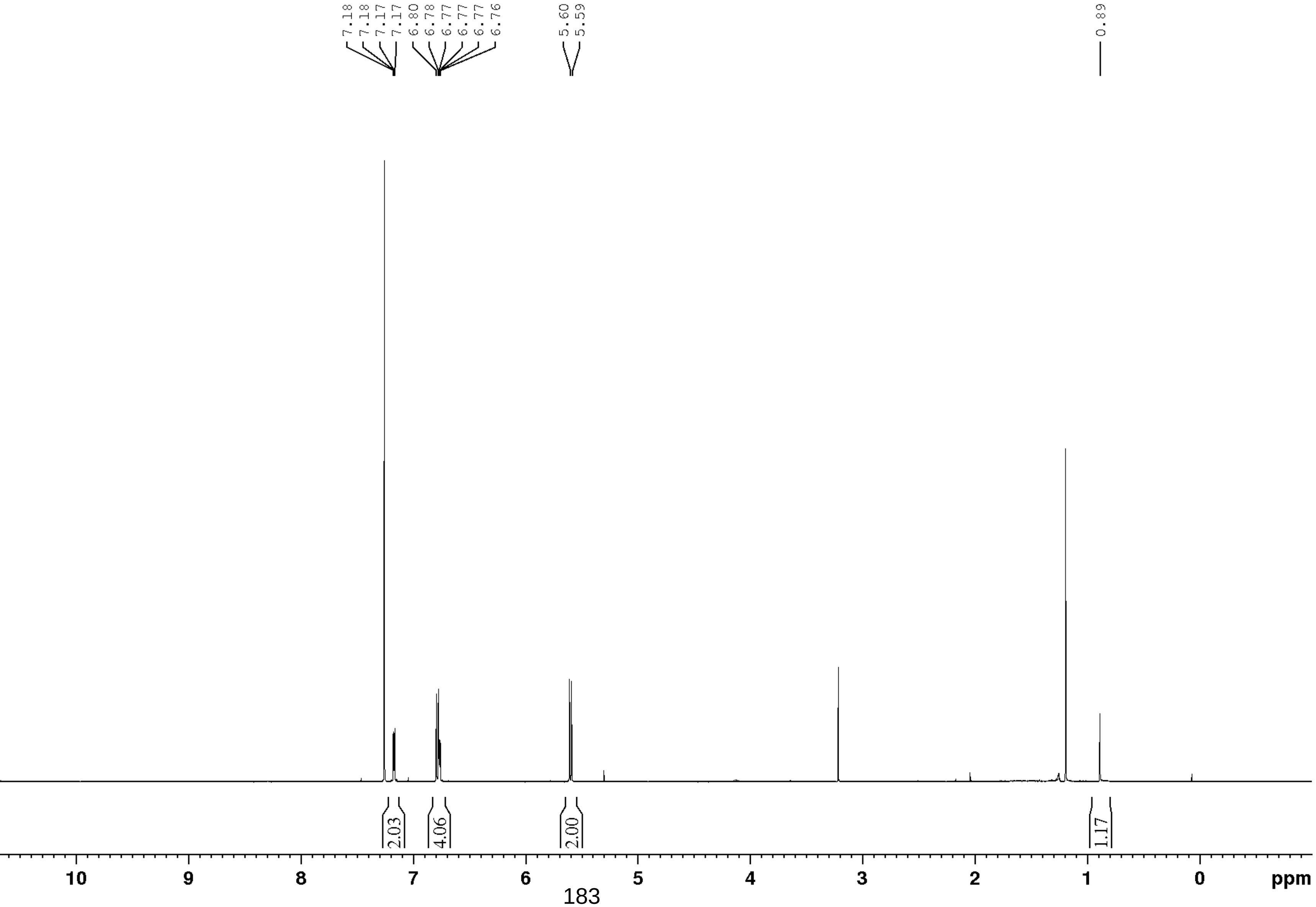

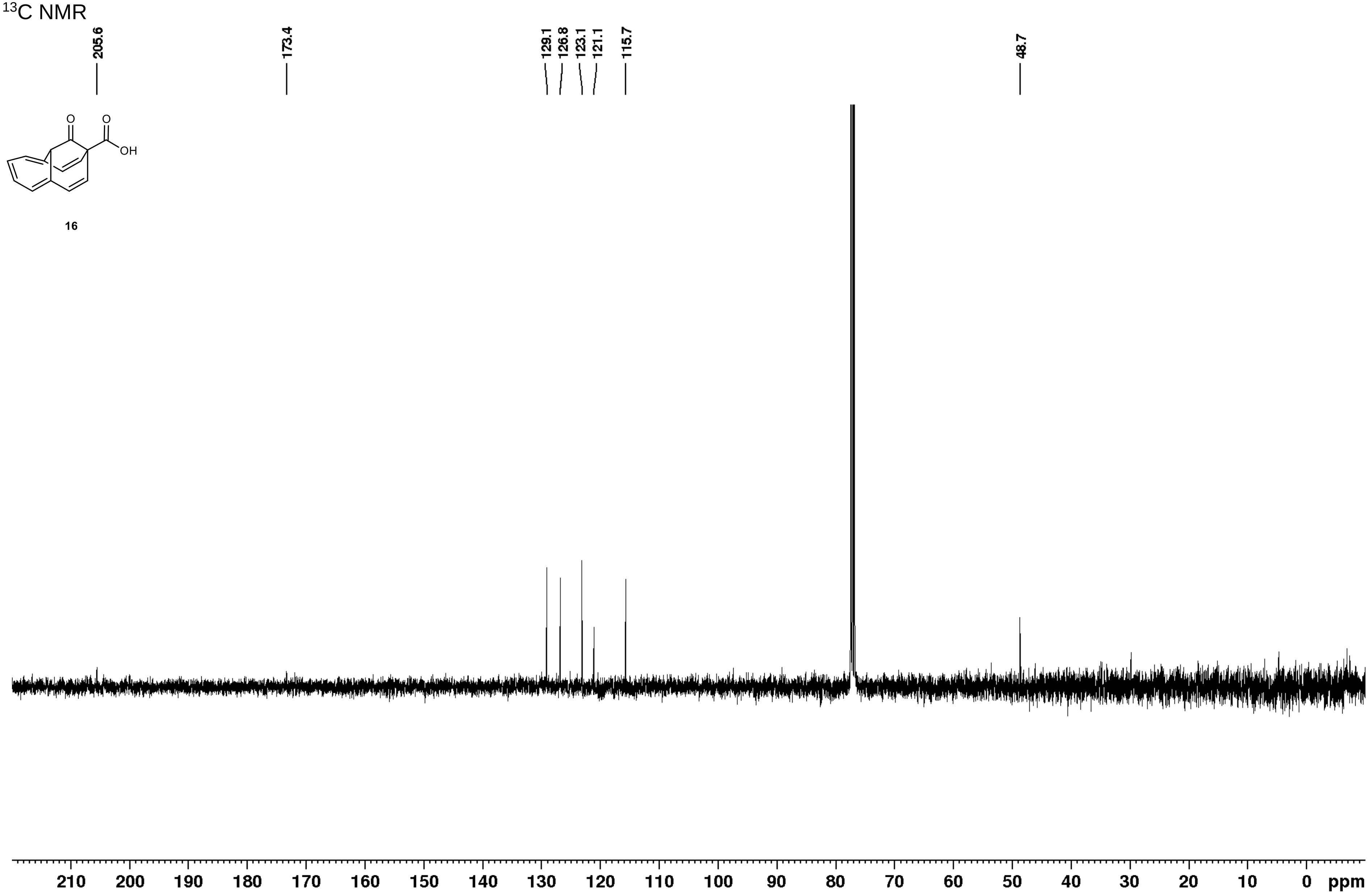

13C NMR
205.6
173.4
129.1
126.8
123.1
121.1
115.7
48.7
O
O
OH
16
210
200
190
180
170
160
150
140
130
120
110
100
90
80
70
60
50
40
30
20
10
0
ppm

^{1}H, ^{1}H COSY

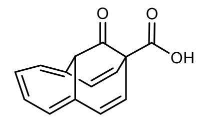

16

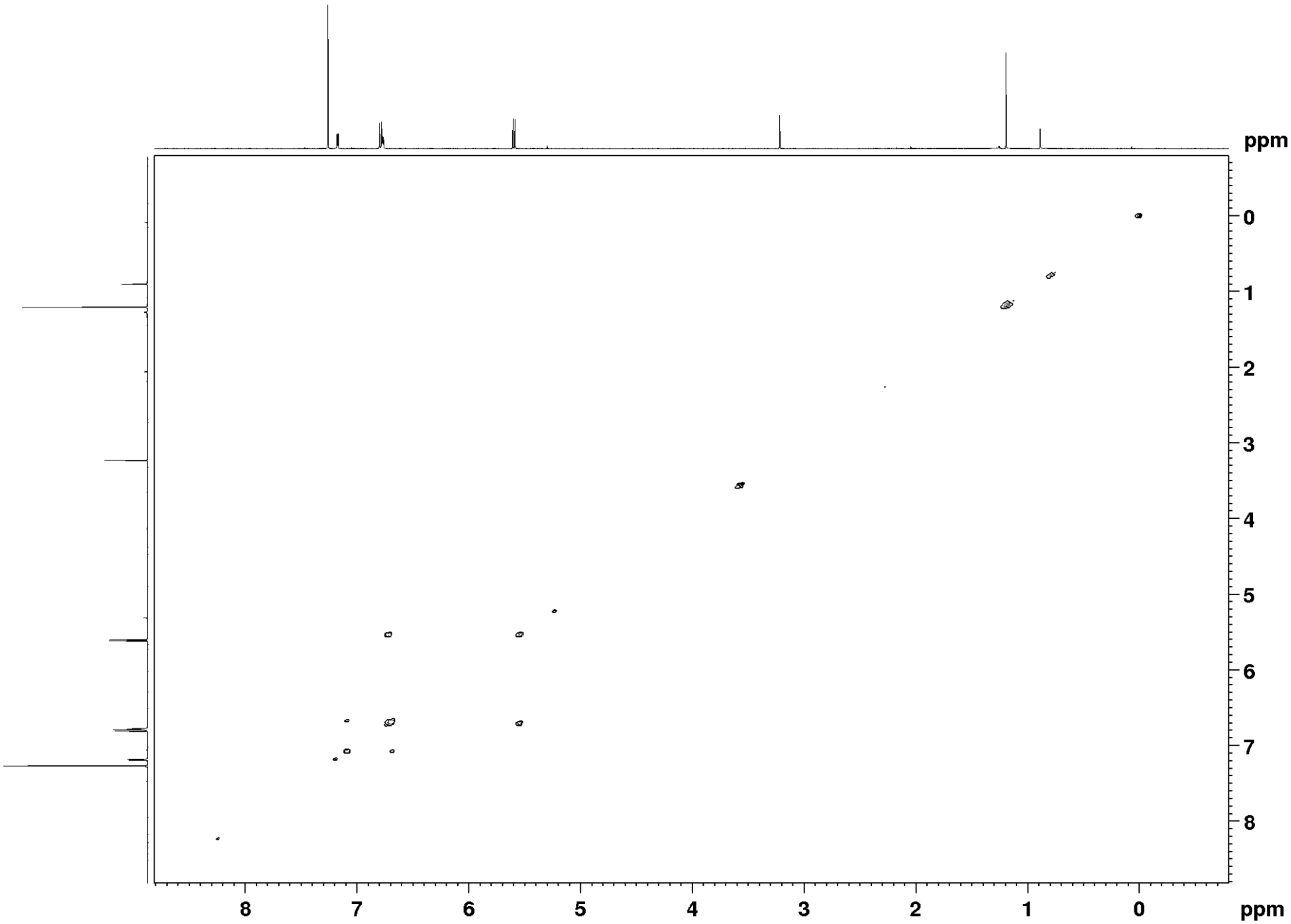

^{1}H, ^{13}C HSQC

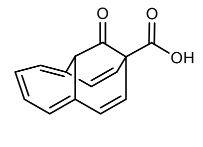

16

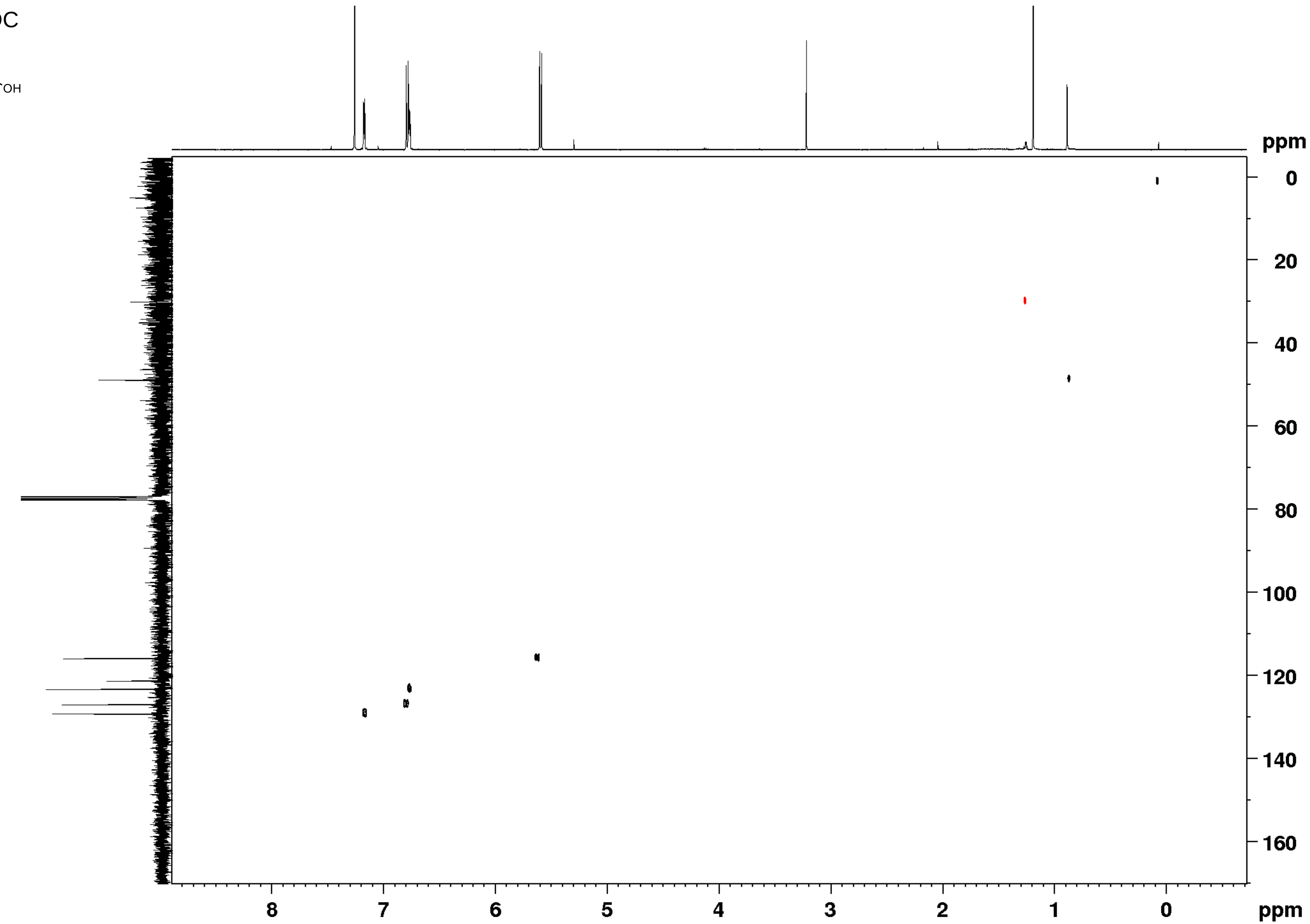

^{1}H, ^{13}C HMBC

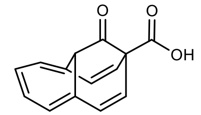

16

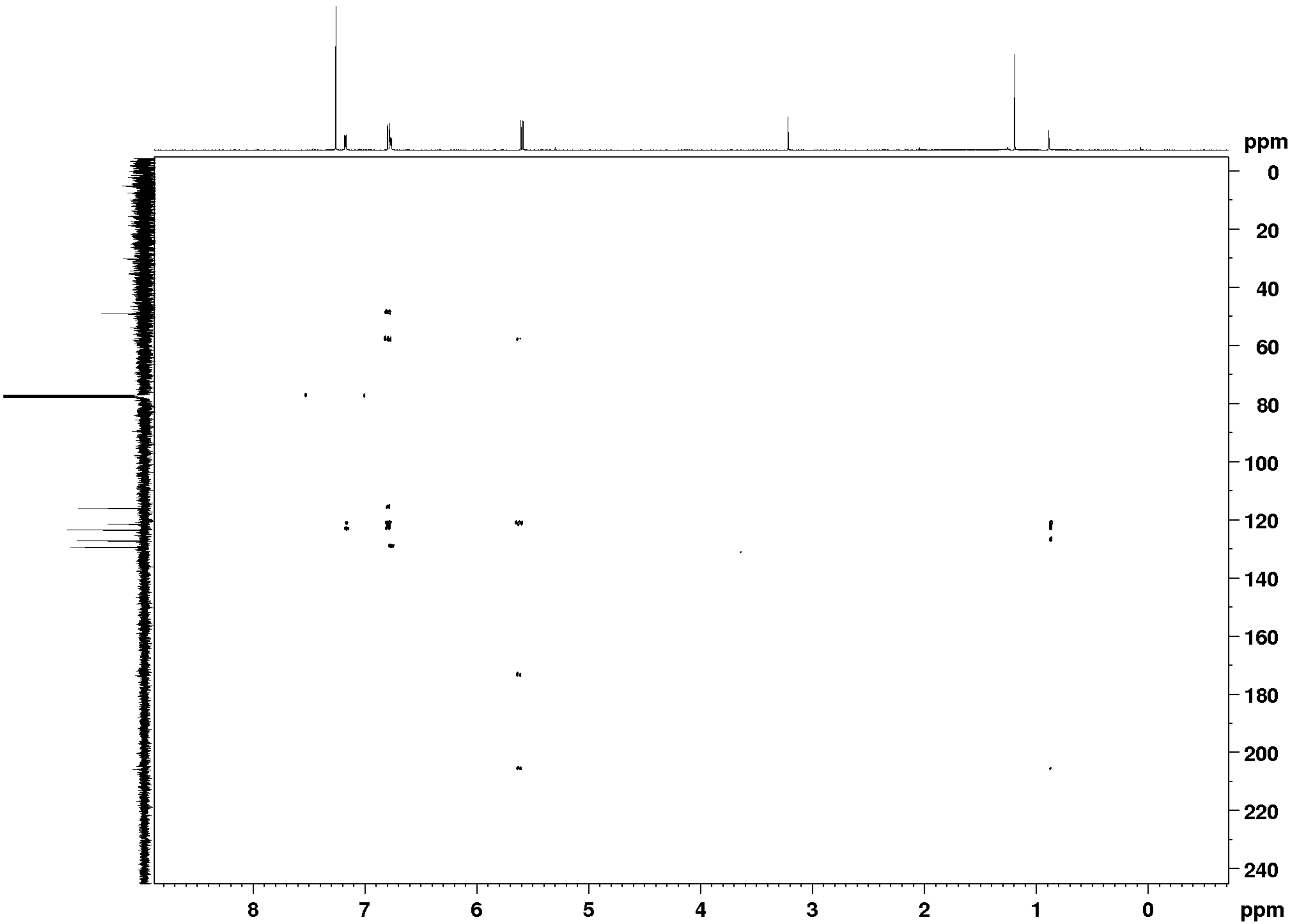

^1H, ^1H NOESY

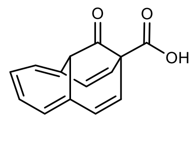

16

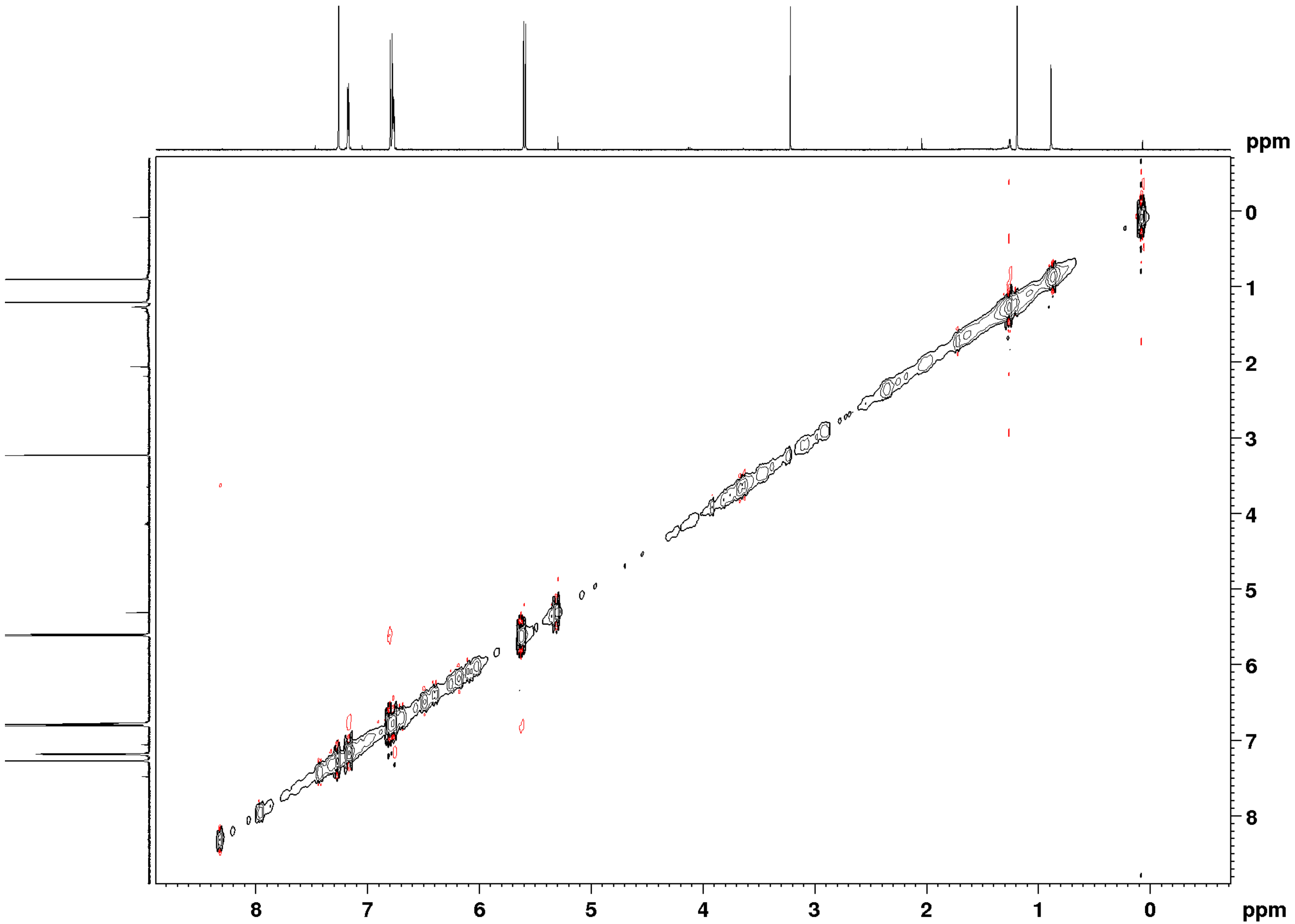

HRMS

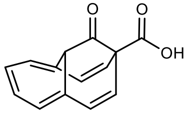

O
O
OH

16

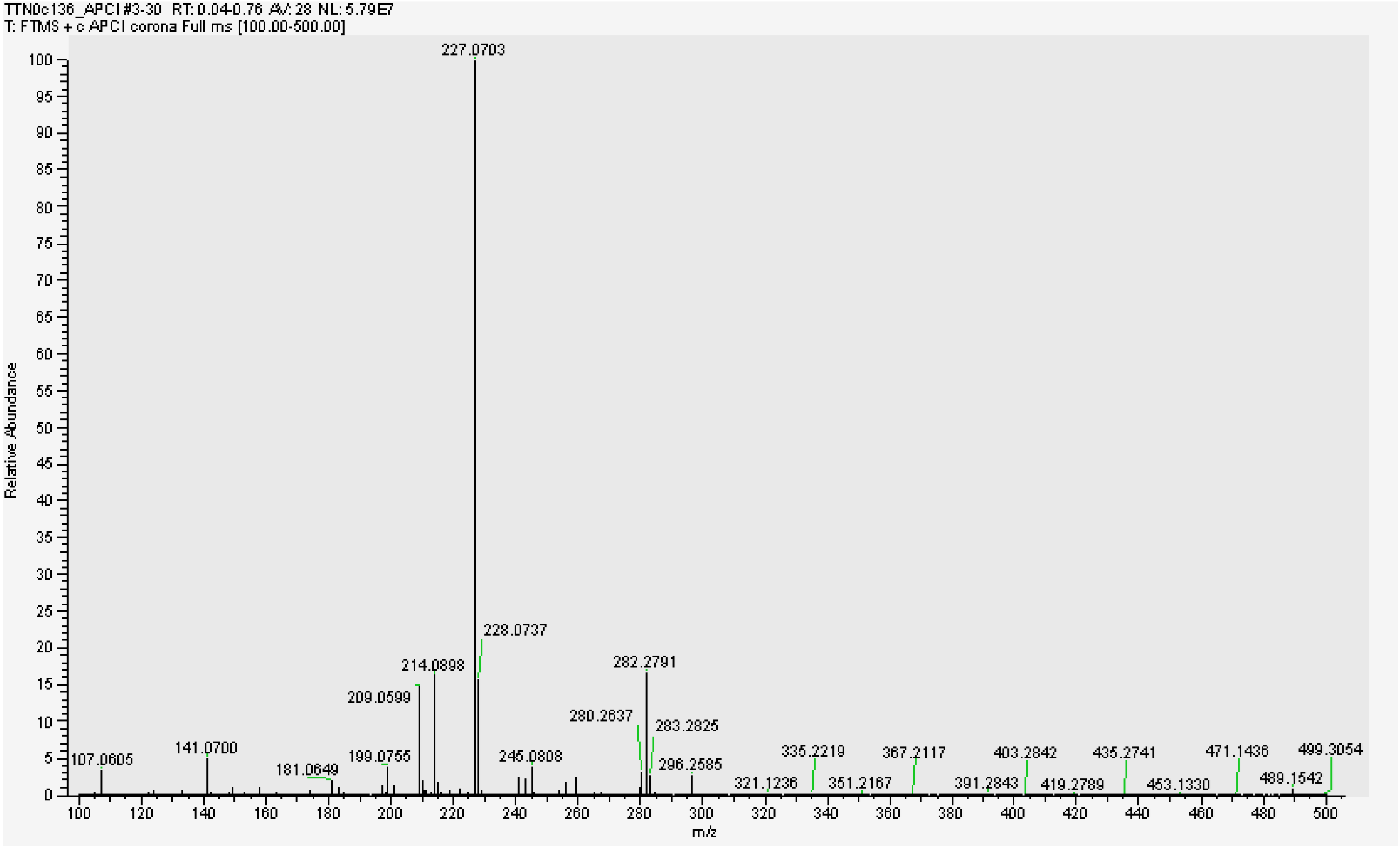

TTN0c136_APCI #3-30 RT: 0.04-0.76 AV: 28 NL: 5.79E7
T: FTMS + c APCI corona Full ms [100.00-500.00]
Relative Abundance
m/z
227.0703
228.0737
214.0898
209.0599
199.0755
181.0649
141.0700
107.0605
245.0808
282.2791
280.2637
283.2825
296.2585
321.1236
335.2219
351.2167
367.2117
391.2843
403.2842
419.2789
435.2741
453.1330
471.1436
489.1542
499.3054

2.2.14 Methyl 10-oxo-9,10-dihydro-10a*H*-1,9-(metheno)heptalene-10a-carboxylate (18 – CD_2Cl_2)

^{1}H NMR

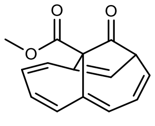

18

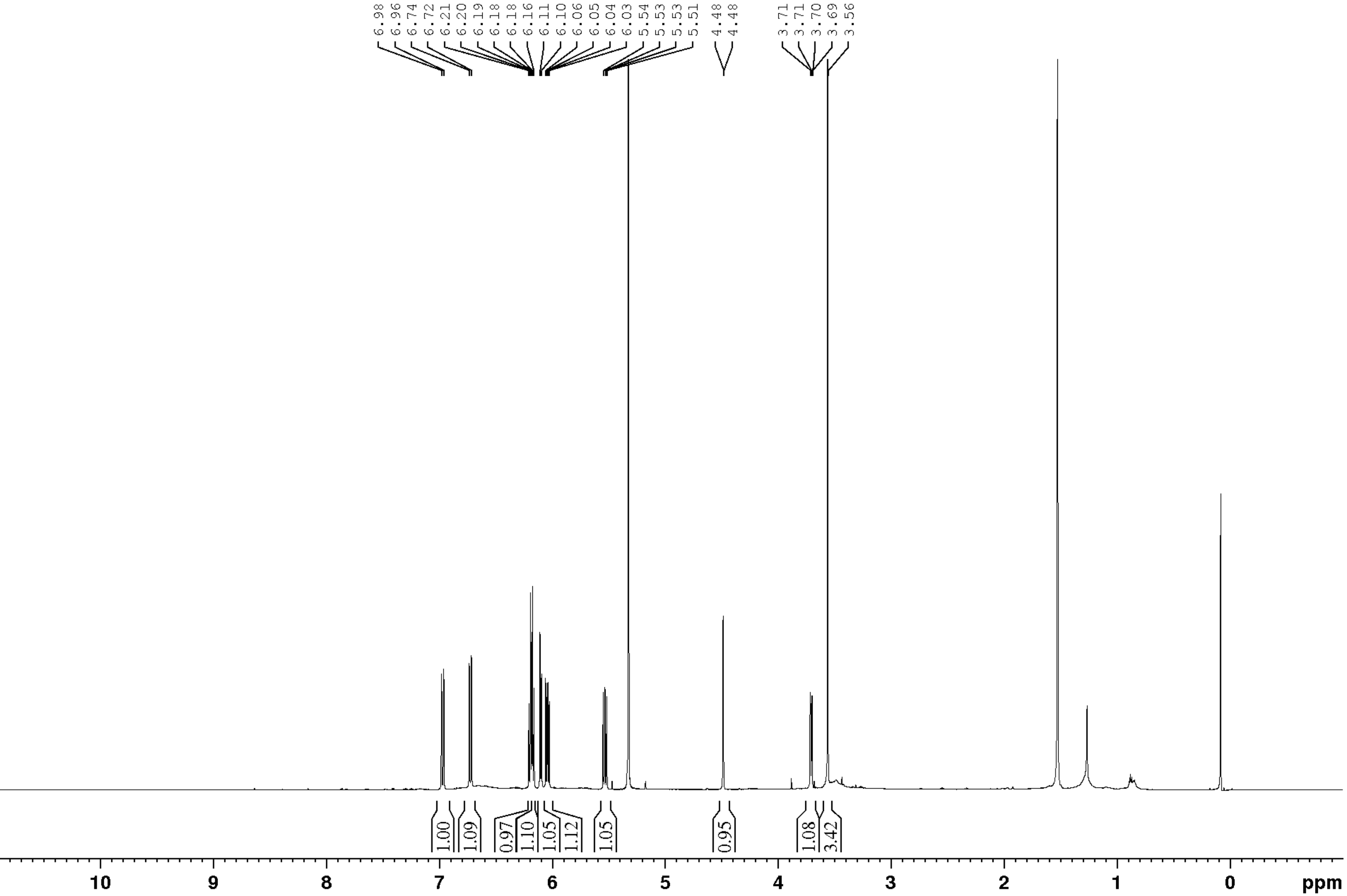

^{13}C NMR

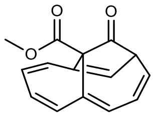

18

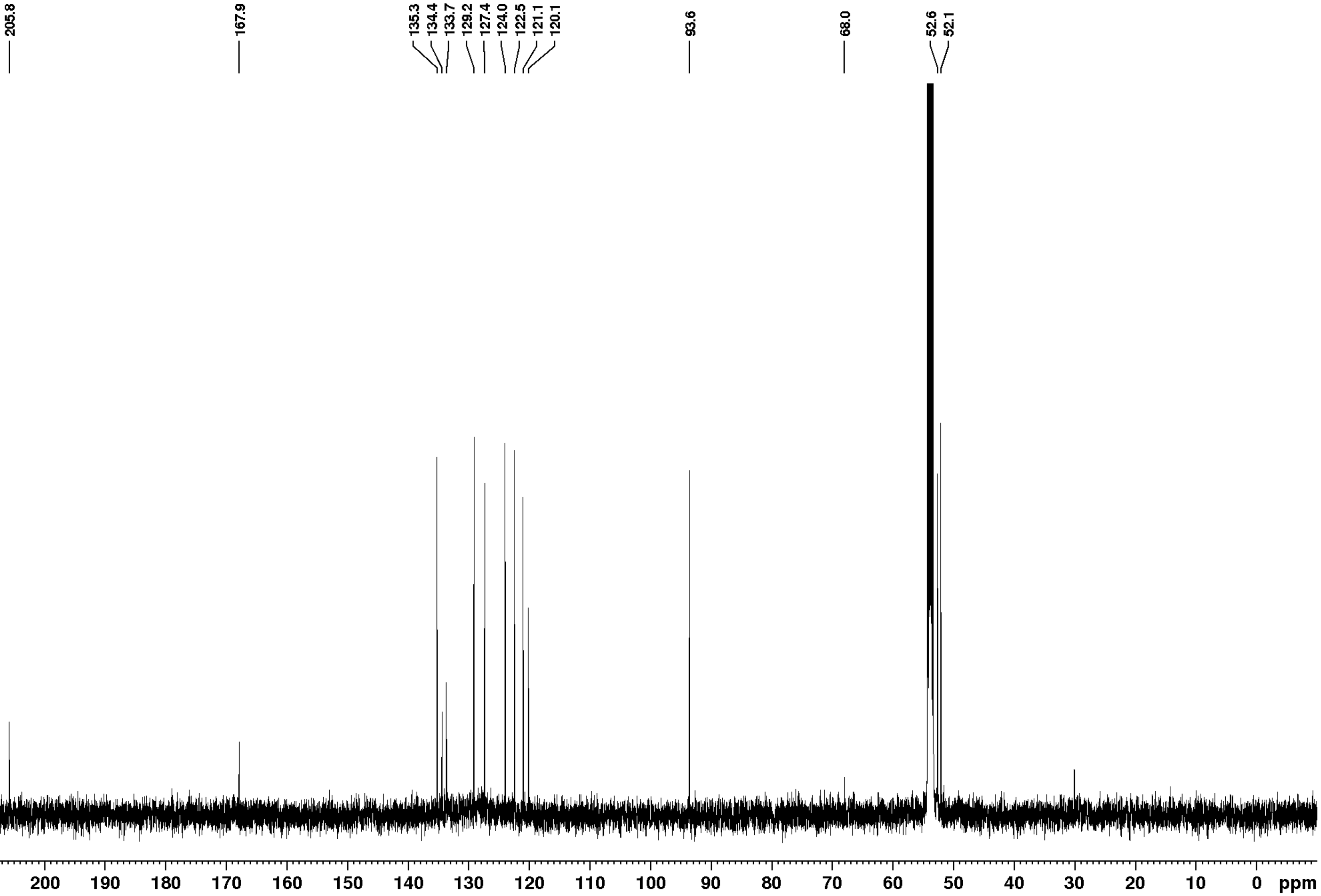
205.8
167.9
135.3
134.4
133.7
129.2
127.4
124.0
122.5
121.1
120.1
93.6
68.0
52.6
52.1
210
200
190
180
170
160
150
140
130
120
110
100
90
80
70
60
50
40
30
20
10
0
ppm

^{1}H, ^{1}H COSY

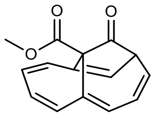

18

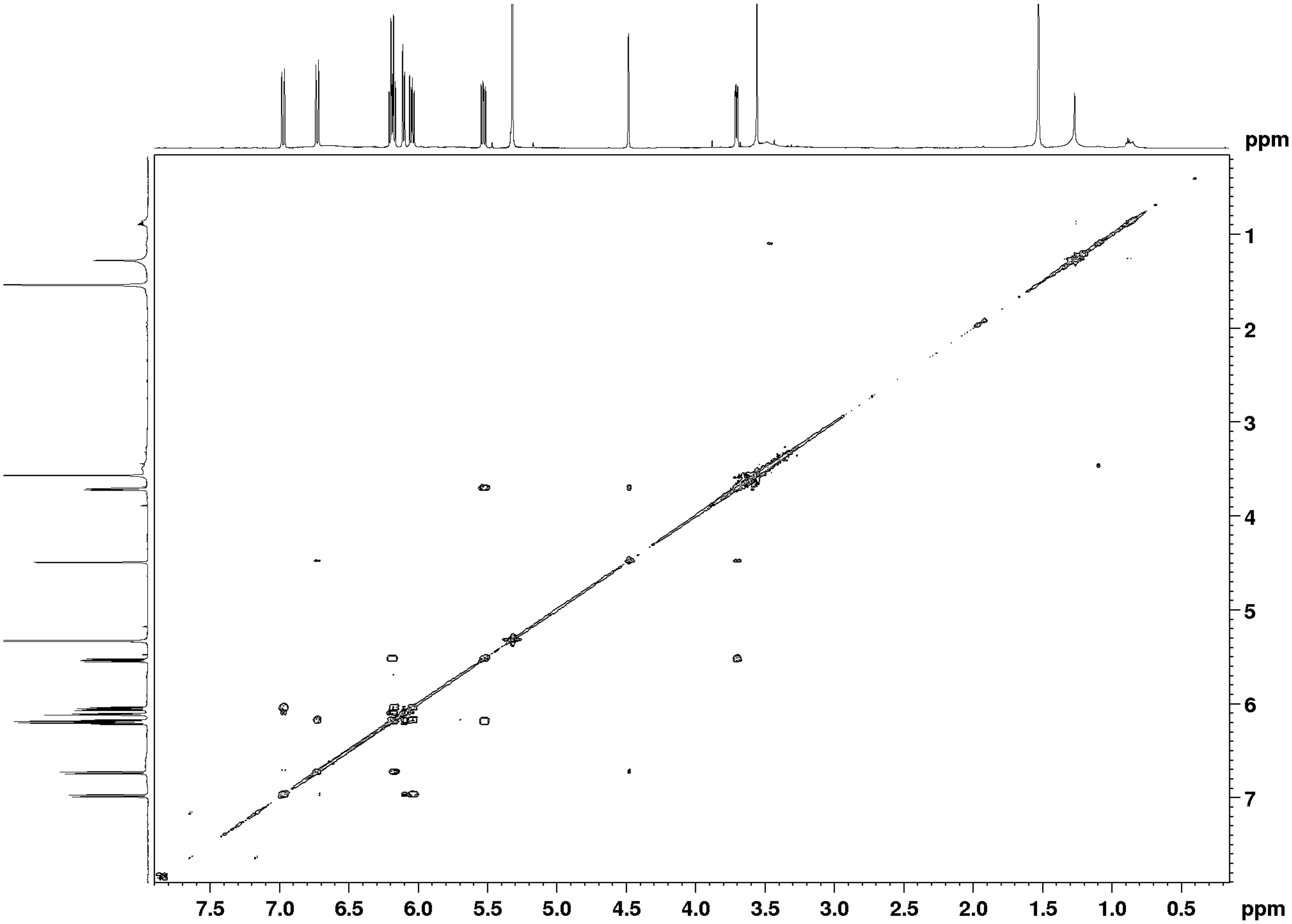

^{1}H, ^{13}C HSQC

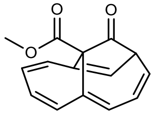

18

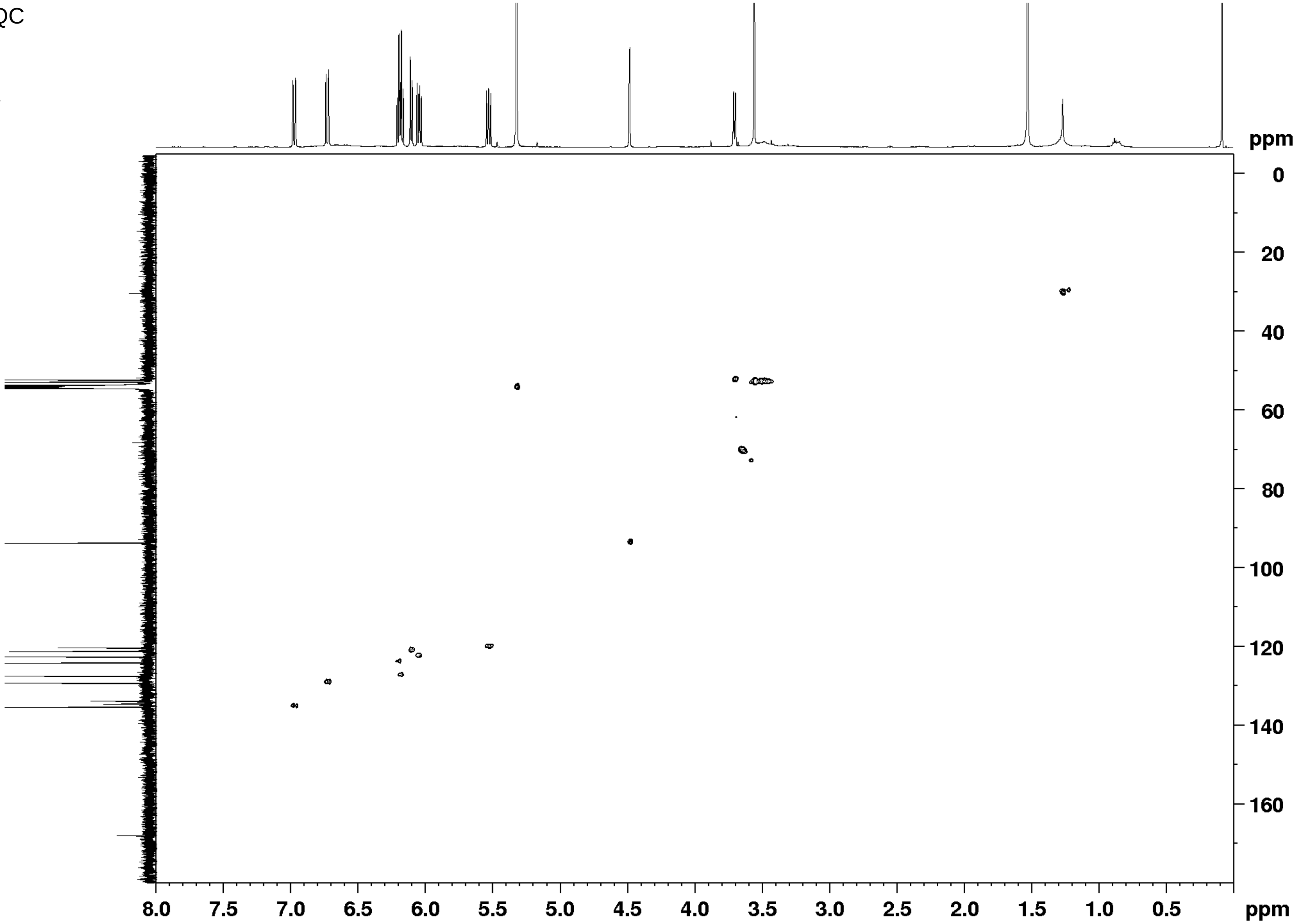

^{1}H, ^{13}C HMBC

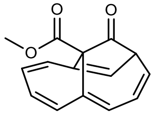

18

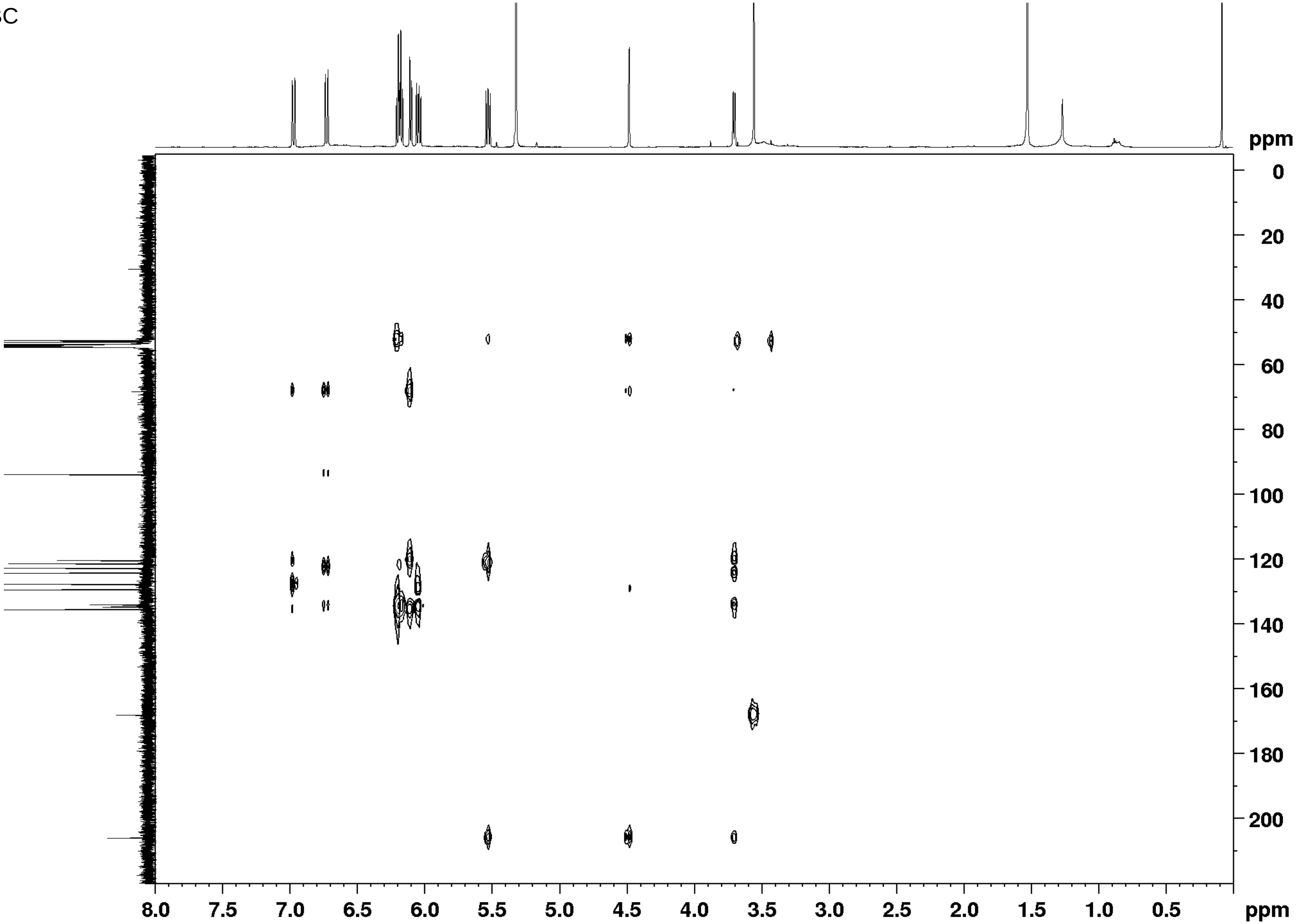

2.2.15 Methyl 10-oxo-9,10-dihydro-10a*H*-1,9-(metheno)heptalene-10a-carboxylate (18– $CDCl_3$)

1H NMR

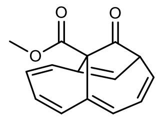

18

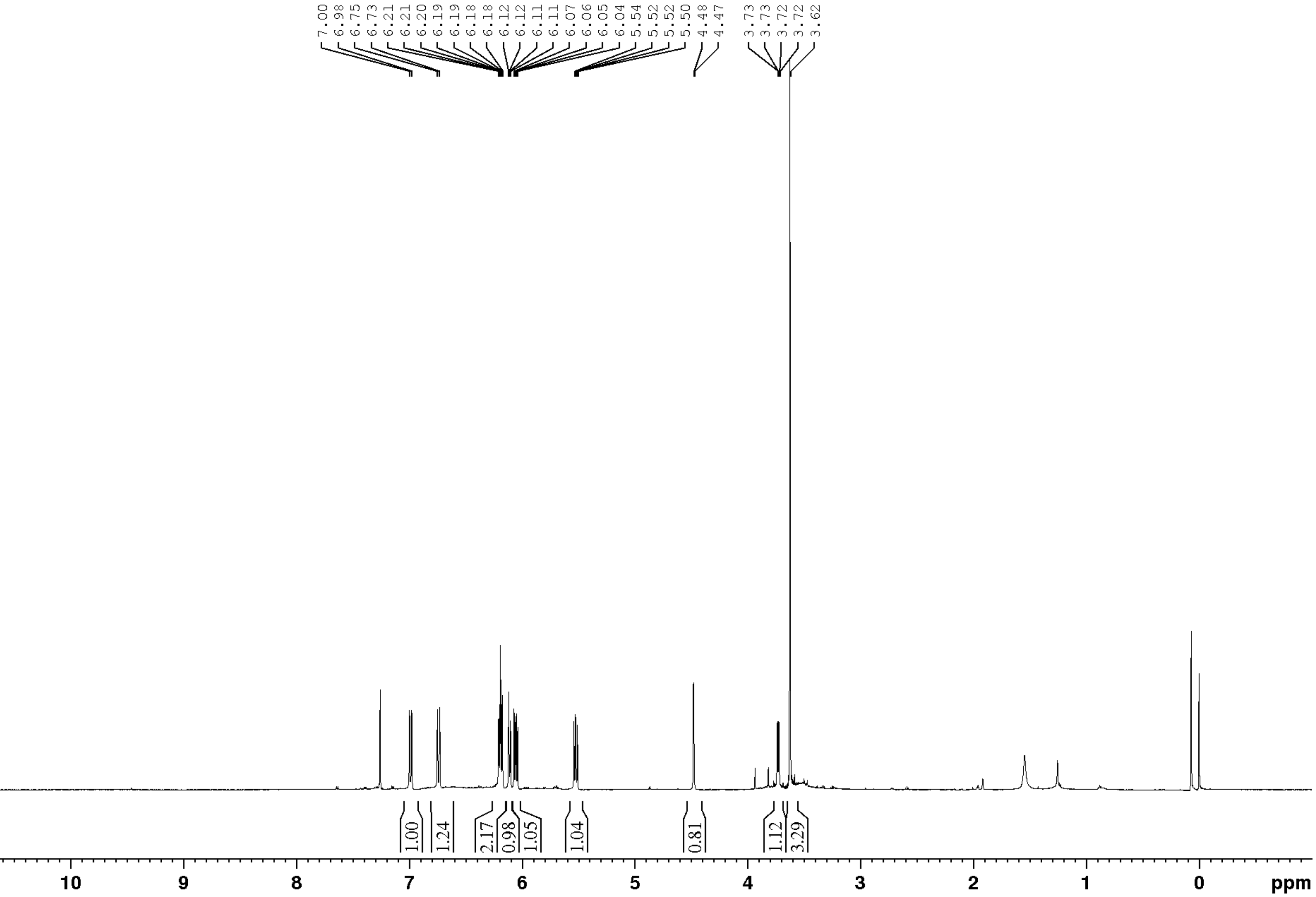

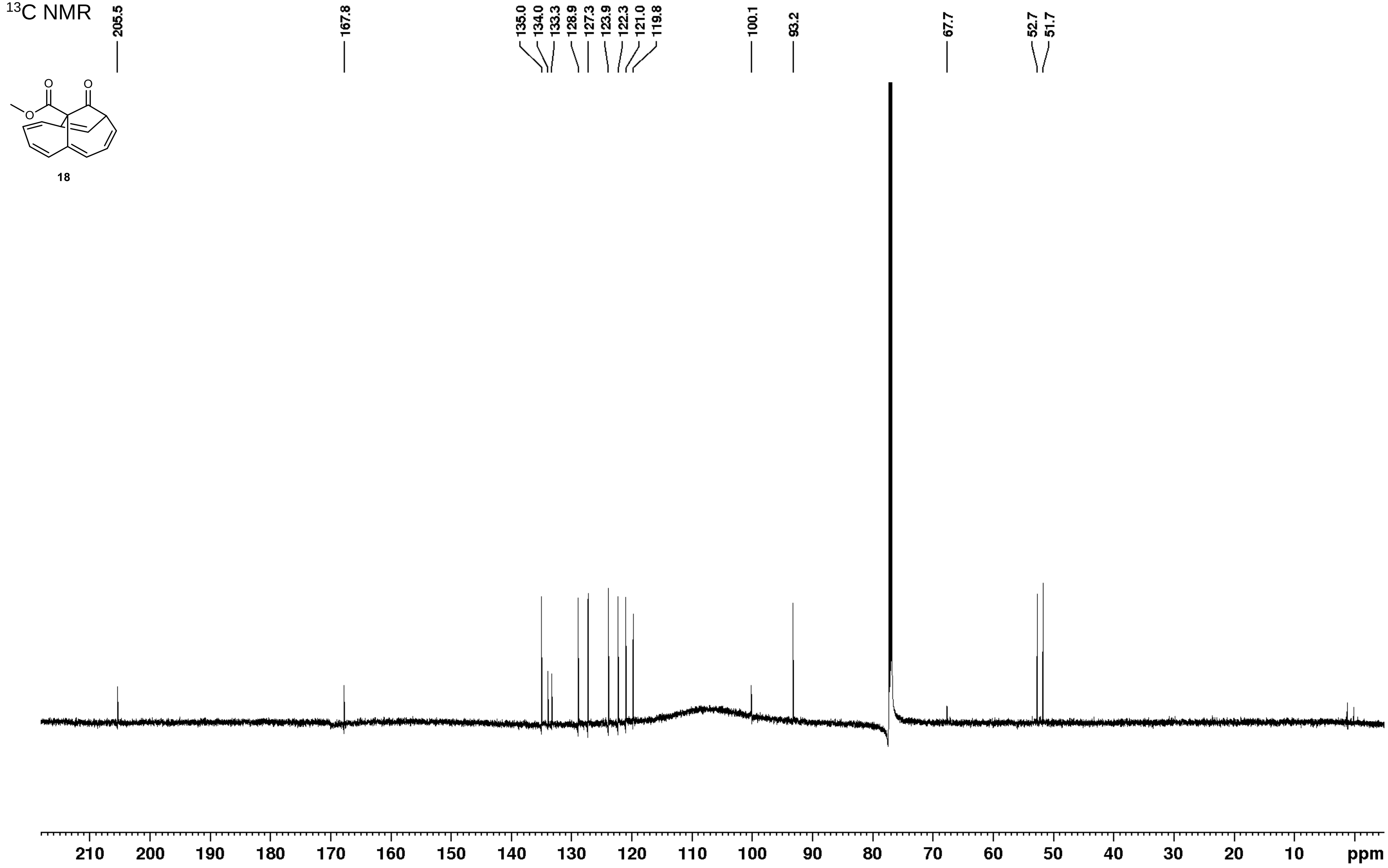

13C NMR
205.5
167.8
135.0
134.0
133.3
128.9
127.3
123.9
122.3
121.0
119.8
100.1
93.2
67.7
52.7
51.7
18
210 200 190 180 170 160 150 140 130 120 110 100 90 80 70 60 50 40 30 20 10 ppm

^1H, ^1H COSY

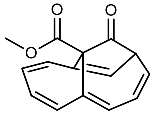

18

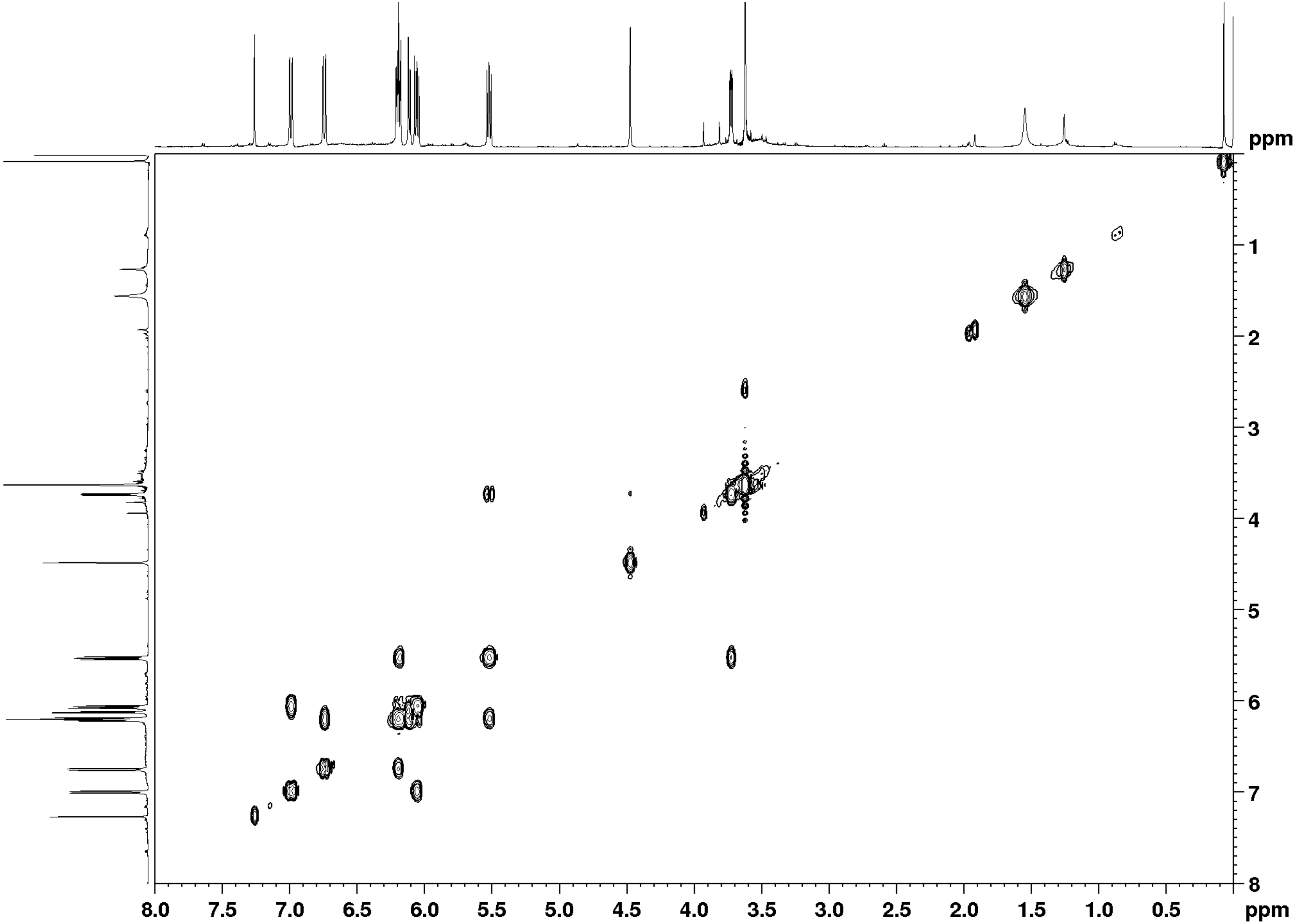

^{1}H, ^{13}C HSQC

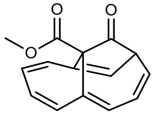

18

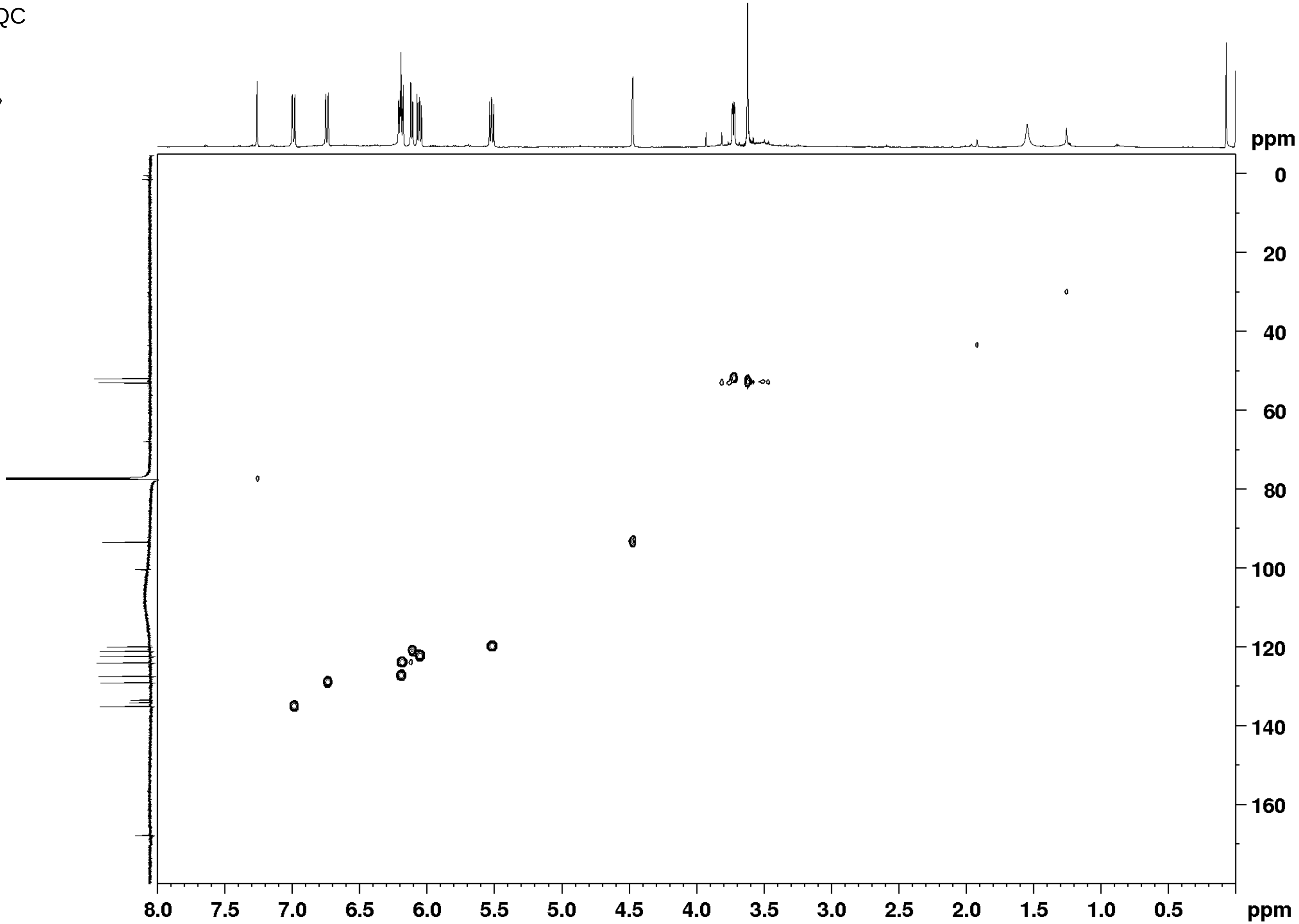

^1H, ^{13}C HMBC

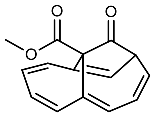

18

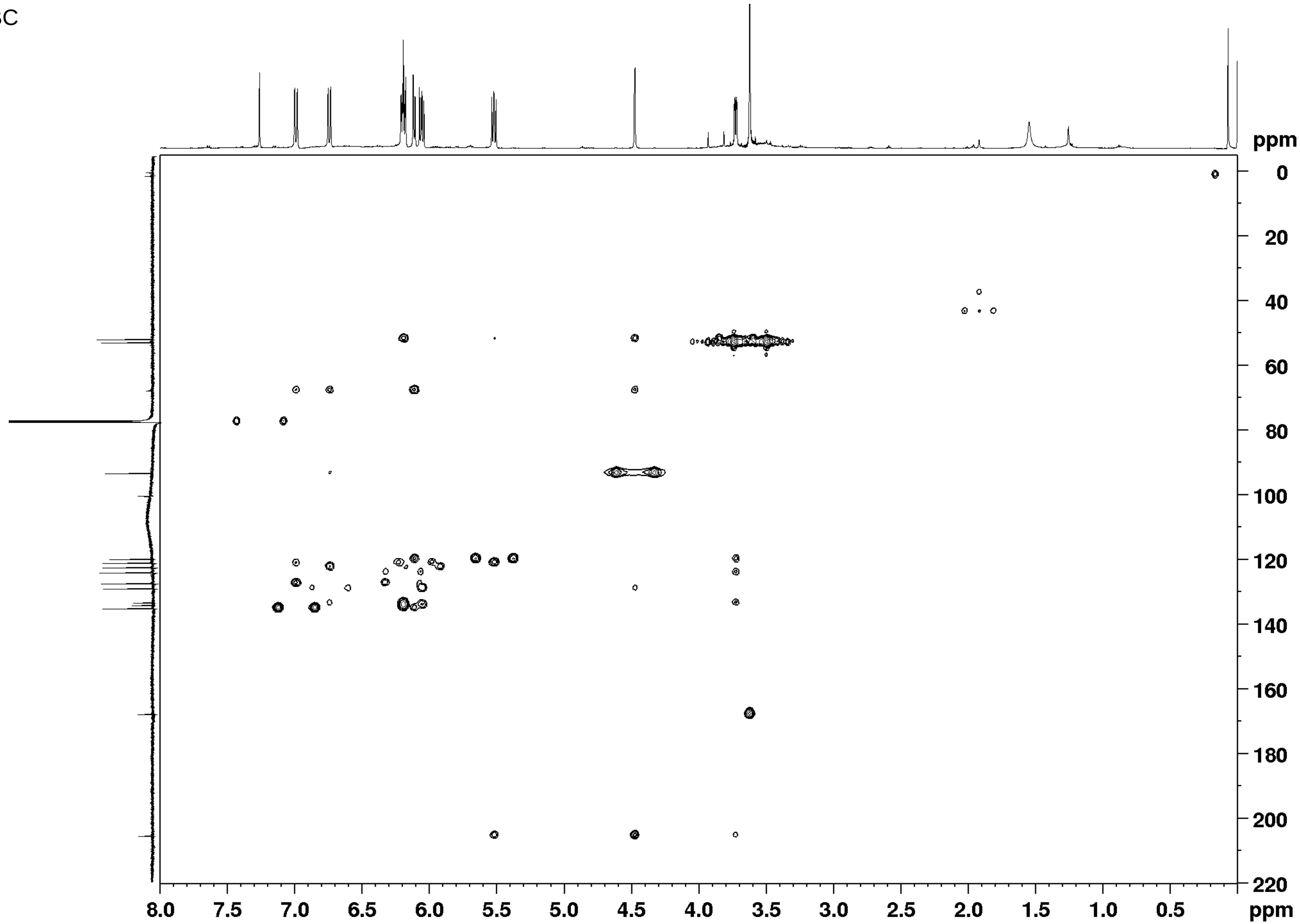

^{1}H, ^{1}H NOESY

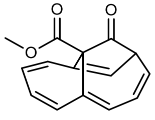

18

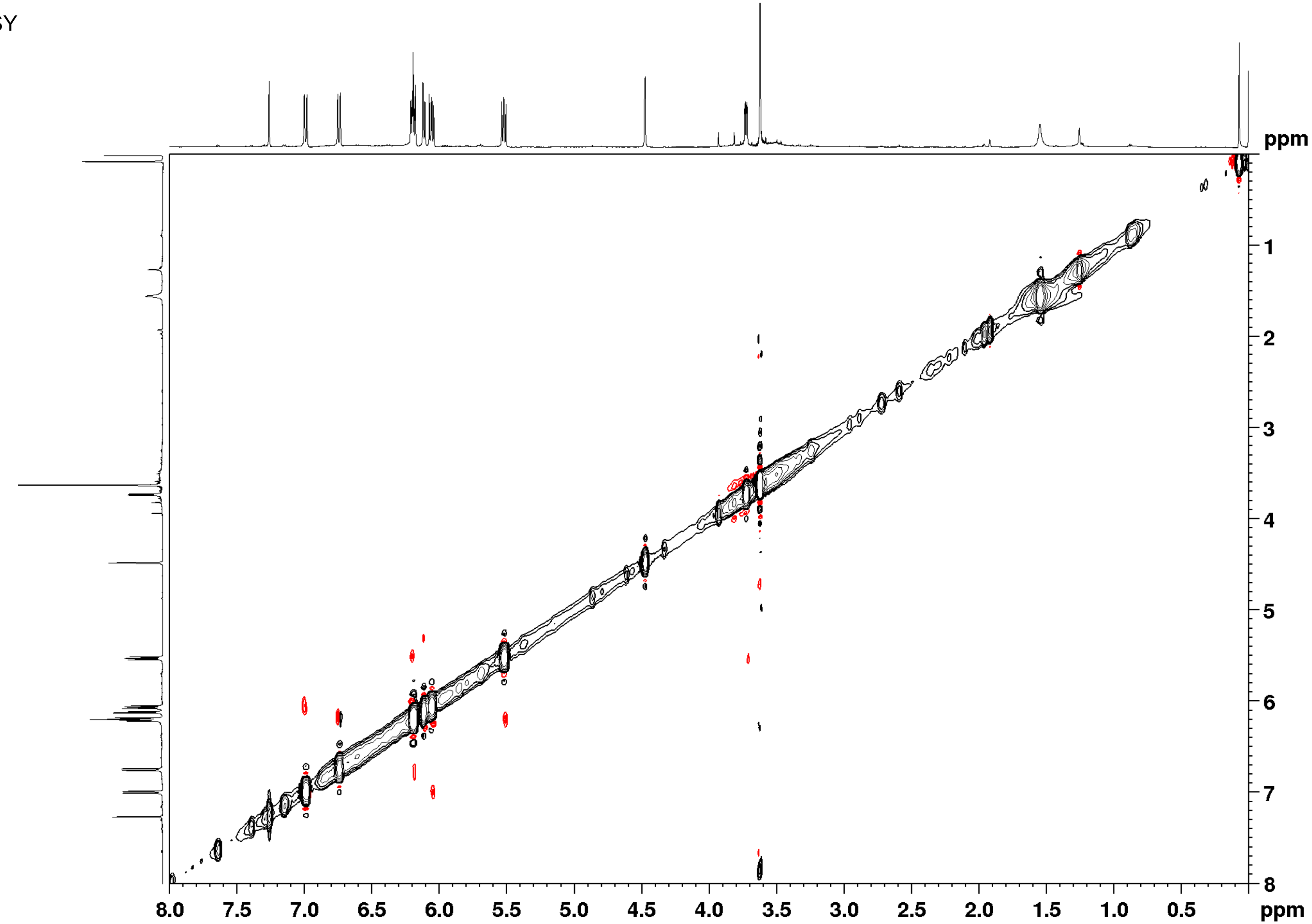

^1H, ^1H TOCSY

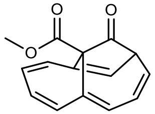

18

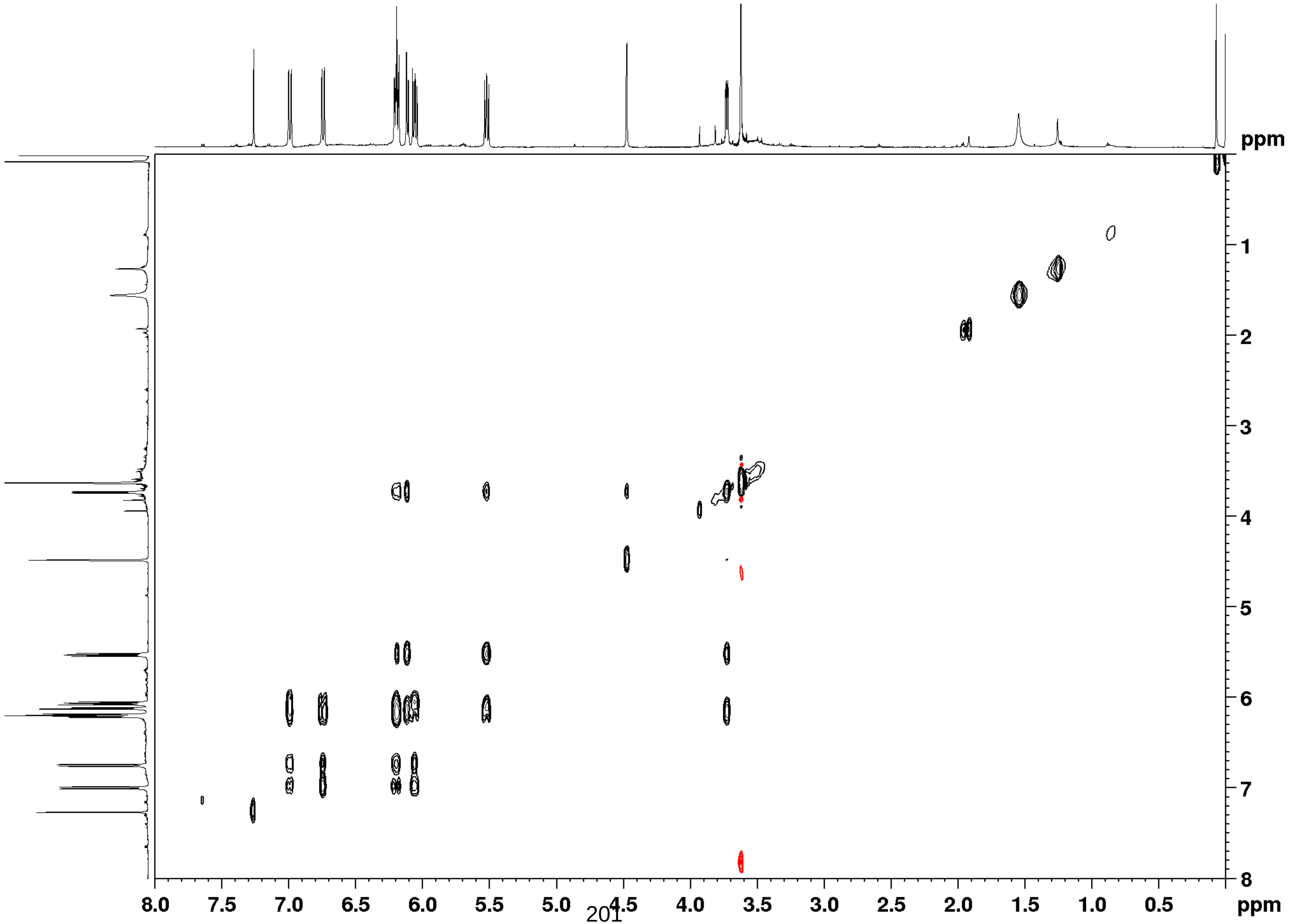

[1]H, ^{1}H ROESY

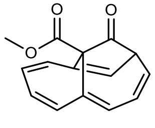

18

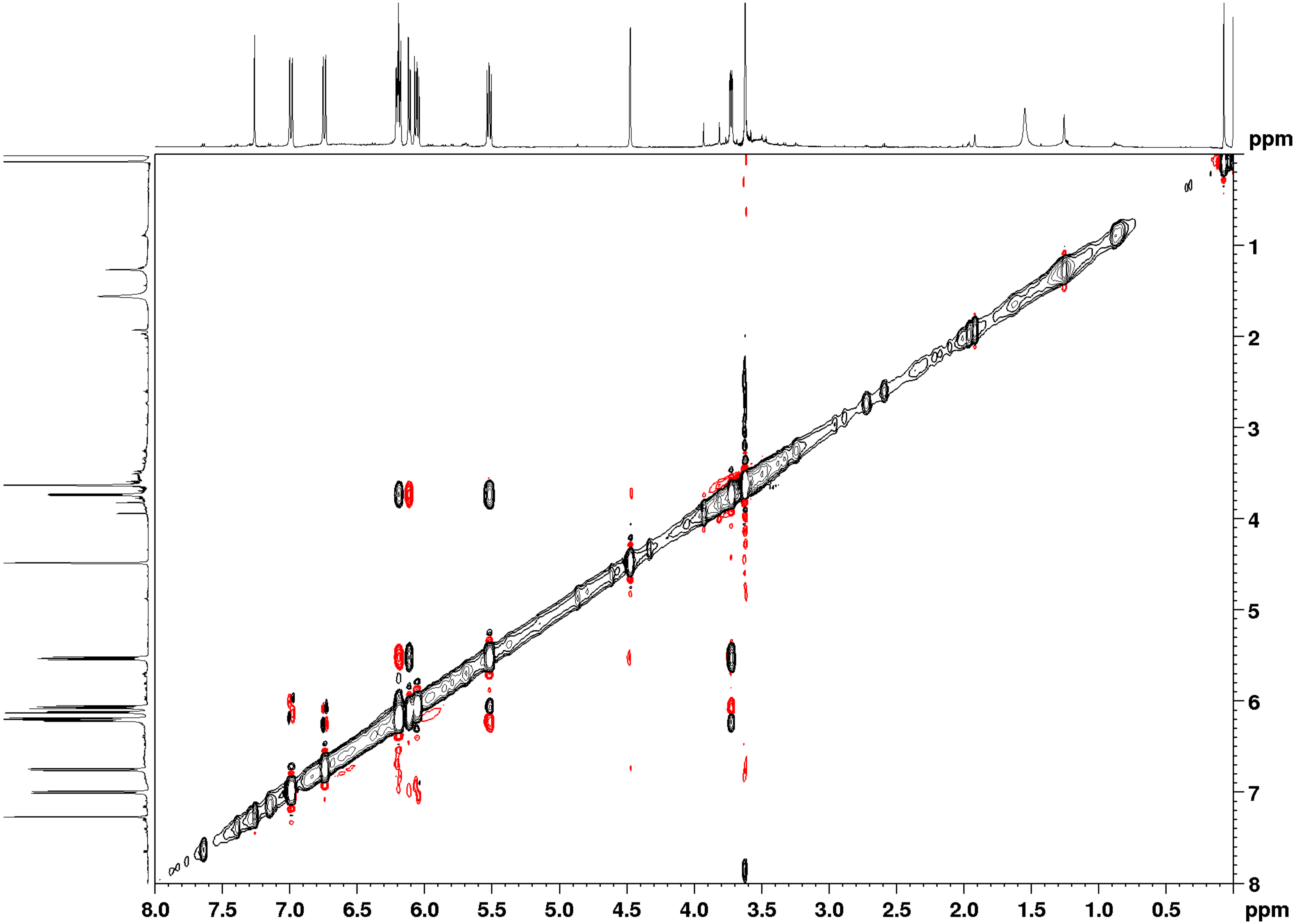

HRMS

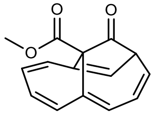

18

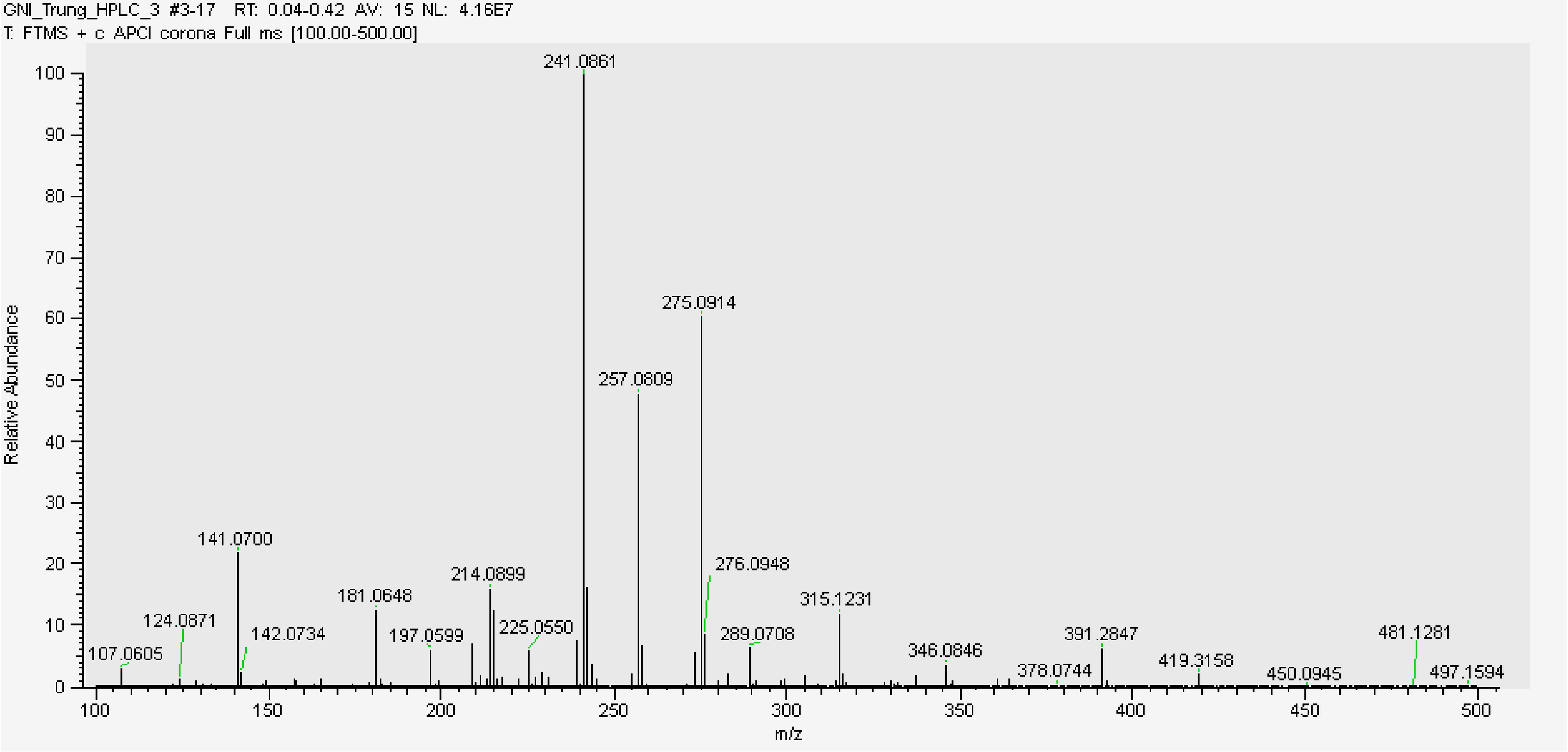

GNI_Trung_HPLC_3 #3-17 RT: 0.04-0.42 AV: 15 NL: 4.16E7
T: FTMS + c APCI corona Full ms [100.00-500.00]
241.0861
275.0914
257.0809
141.0700
276.0948
214.0899
181.0648
315.1231
124.0871
225.0550
142.0734
197.0599
289.0708
391.2847
481.1281
107.0605
346.0846
419.3158
378.0744
450.0945
497.1594
Relative Abundance
m/z

2.2.16 2-Diazo-1-((1*s*,1a*R*,7a*S*)-1a,2,7,7a-tetrahydro-1*H*-cyclopropa[b]naphthalen-1-yl)ethan-1-one (*S*10)

^{1}H NMR

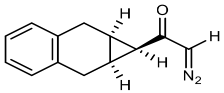

S10

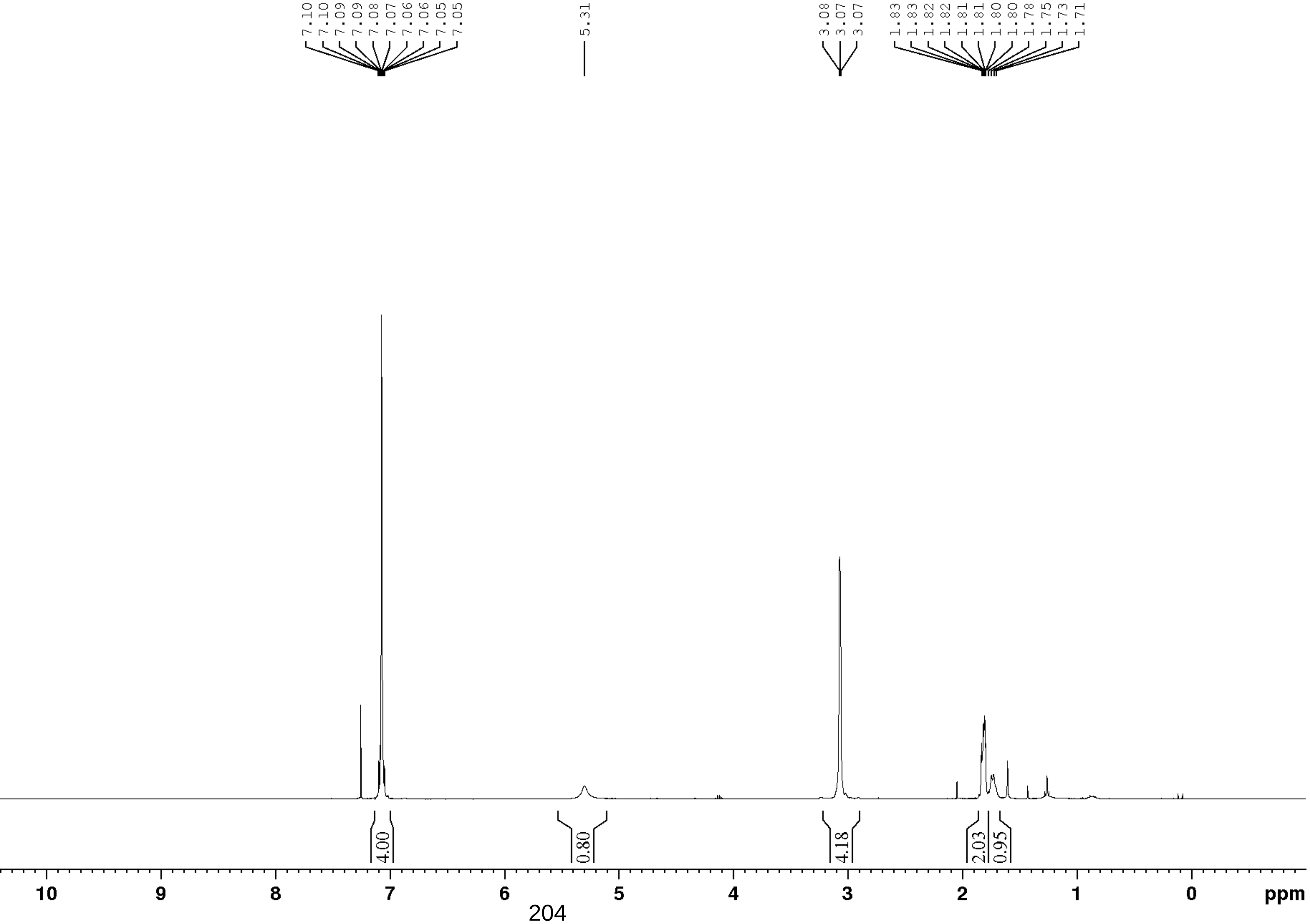

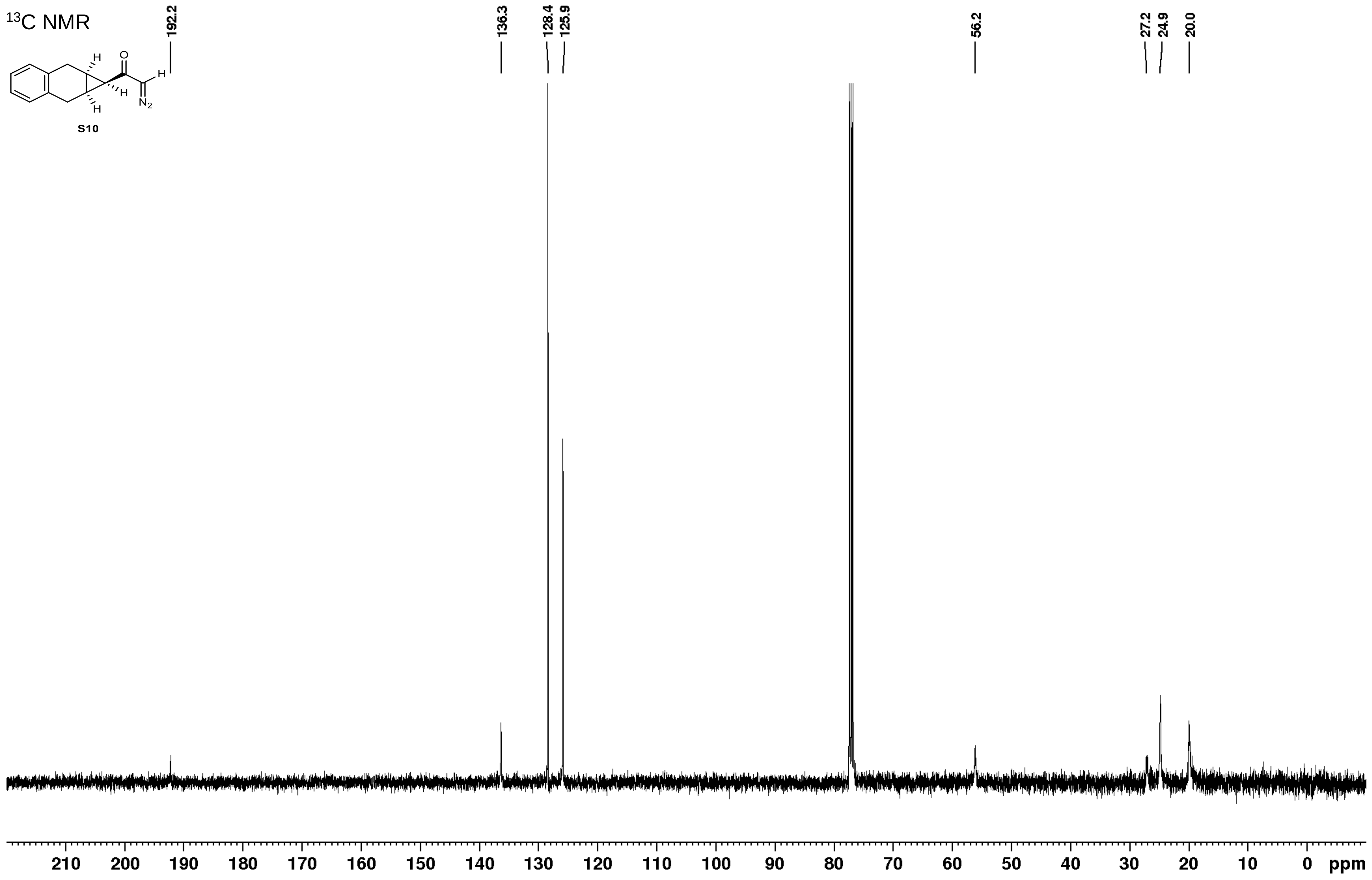

^{13}C NMR
192.2
136.3
128.4
125.9
56.2
27.2
24.9
20.0
H
O
H
H
H
N_2
S10
210 200 190 180 170 160 150 140 130 120 110 100 90 80 70 60 50 40 30 20 10 0 ppm

^{1}H, ^{1}H COSY

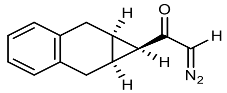

S10

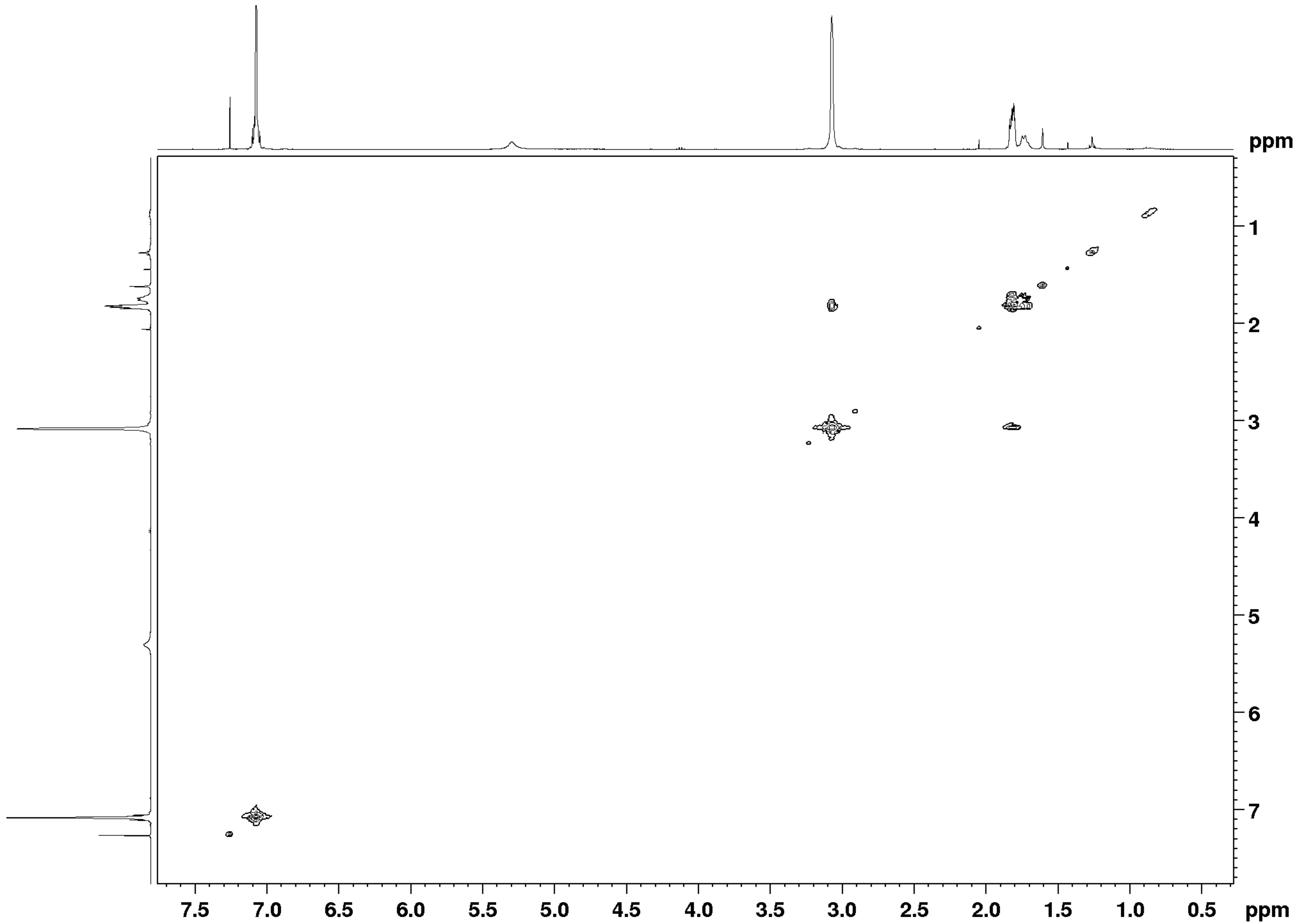

^{1}H, ^{13}C HSQC

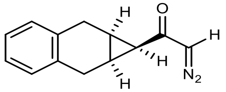

S10

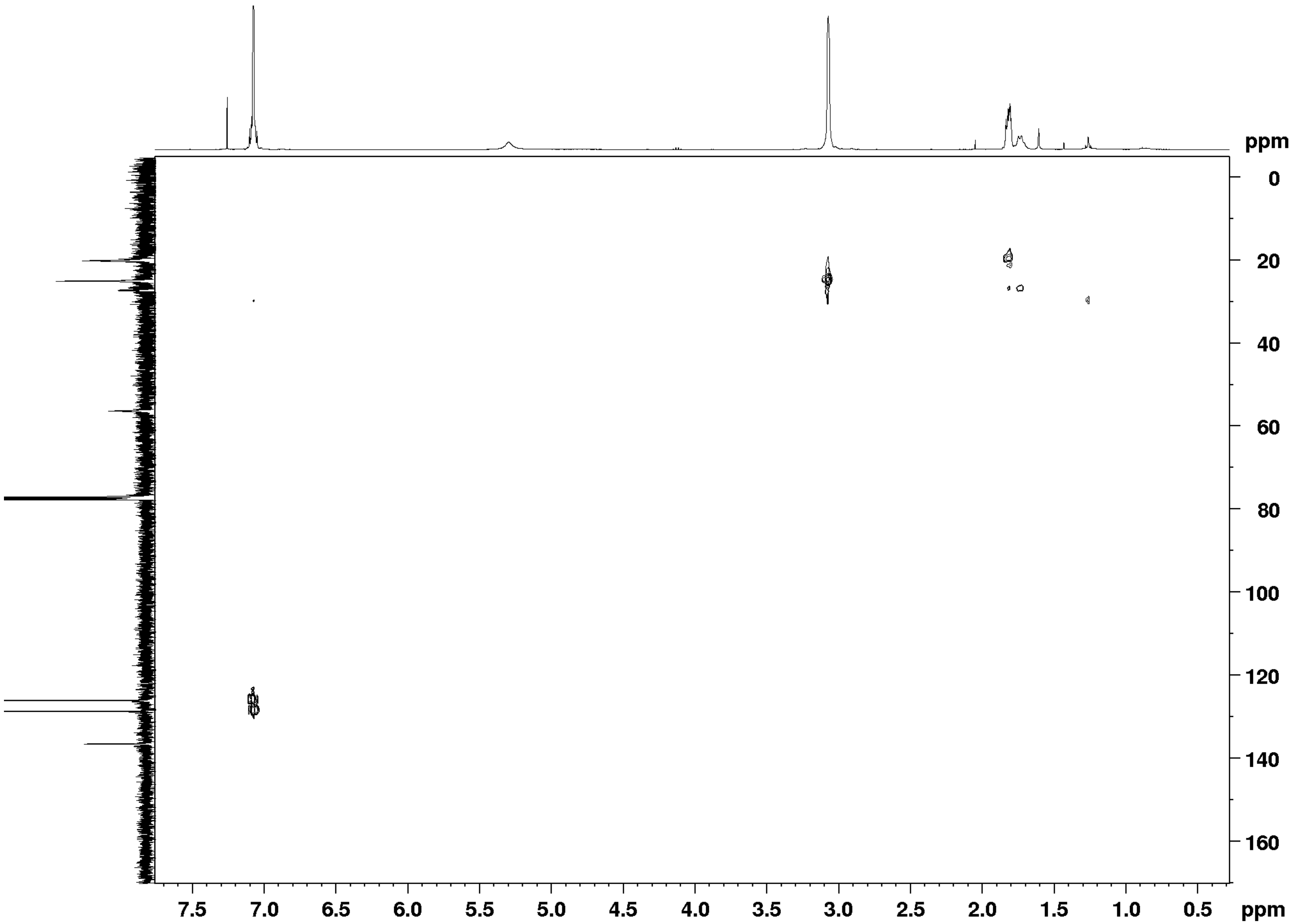

^{1}H, ^{13}C HMBC

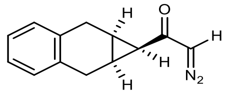

S10

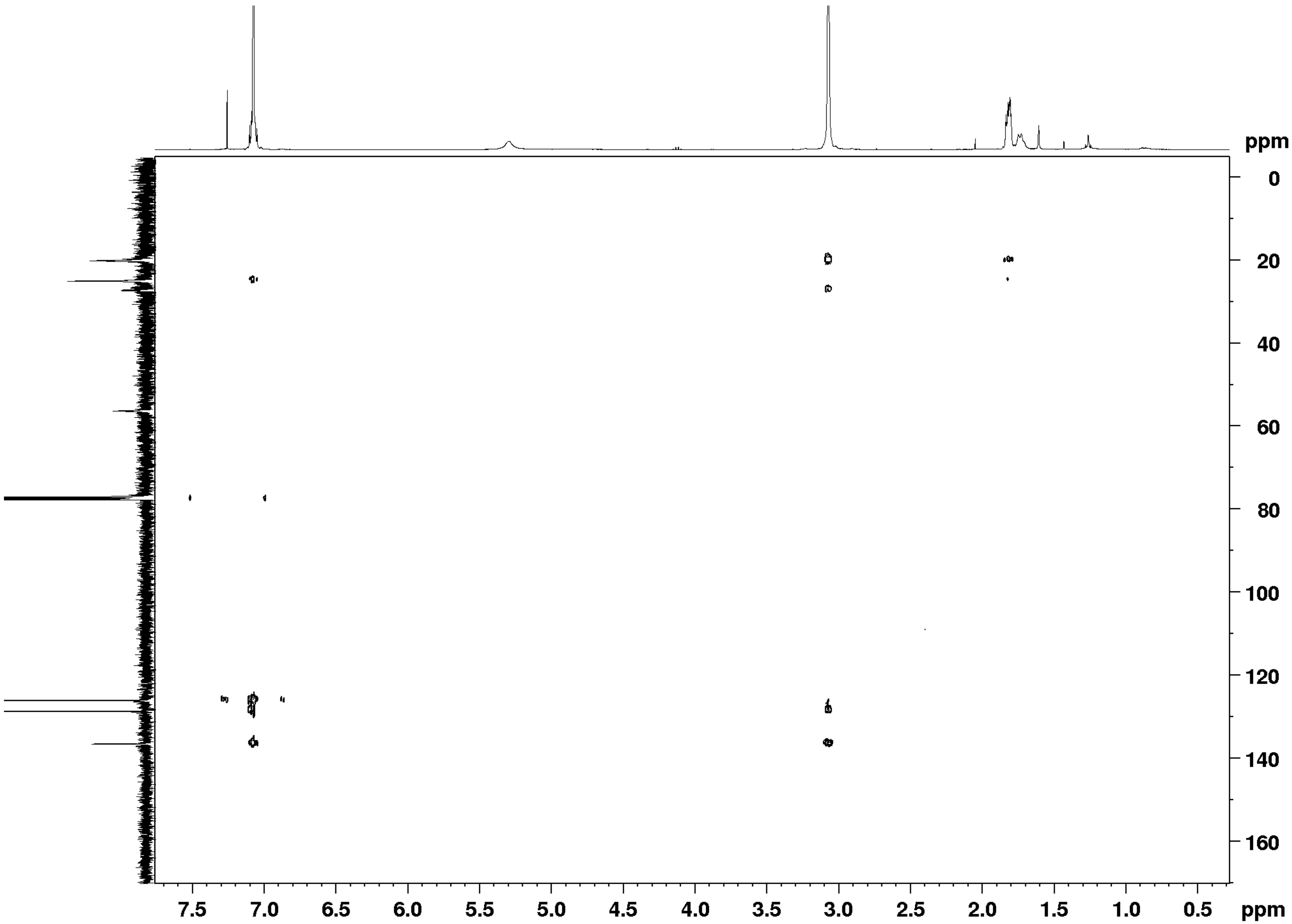

^{1}H, ^{1}H NOESY

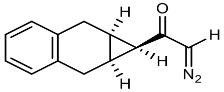

S10

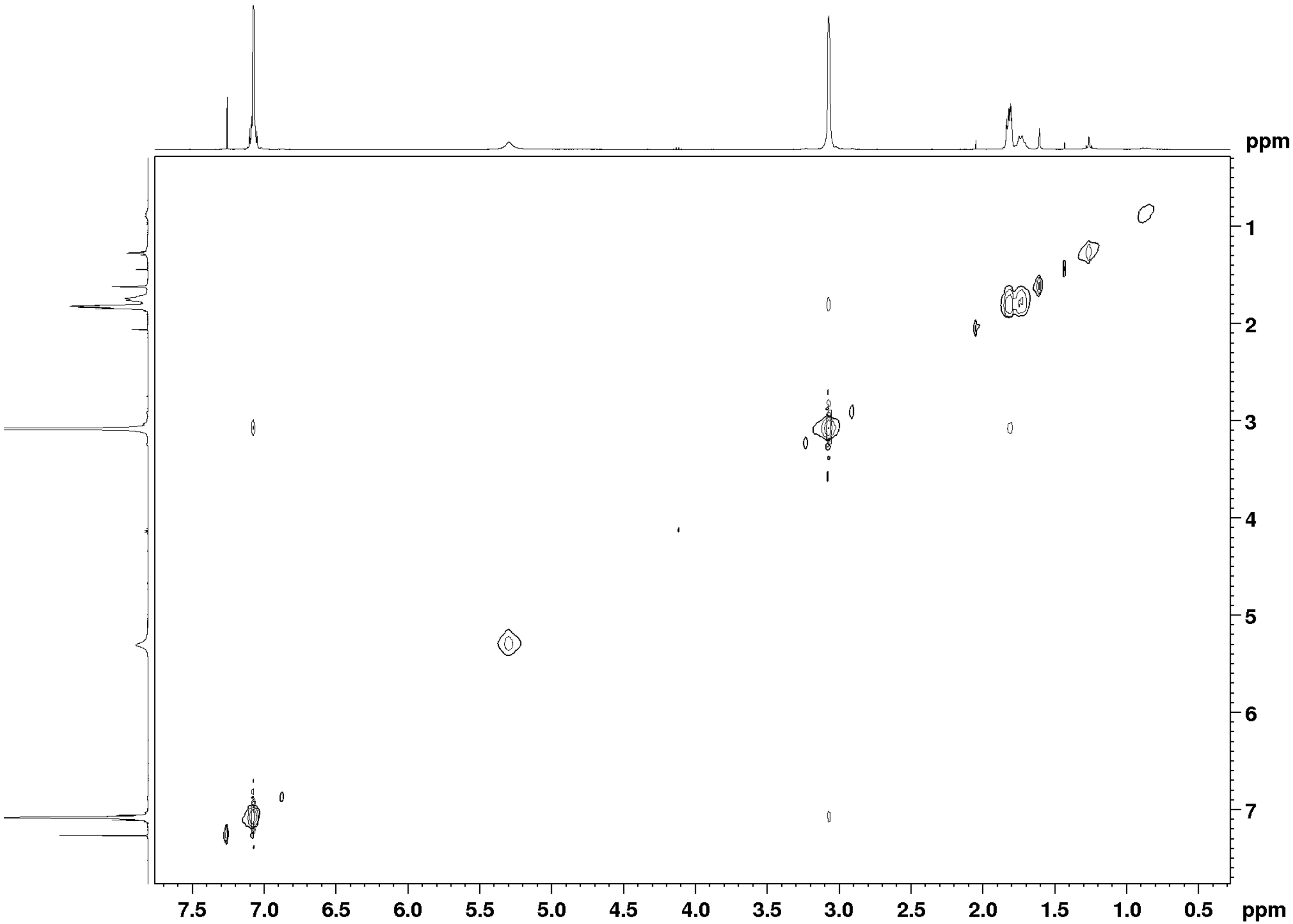

HRMS

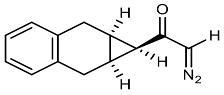

S10

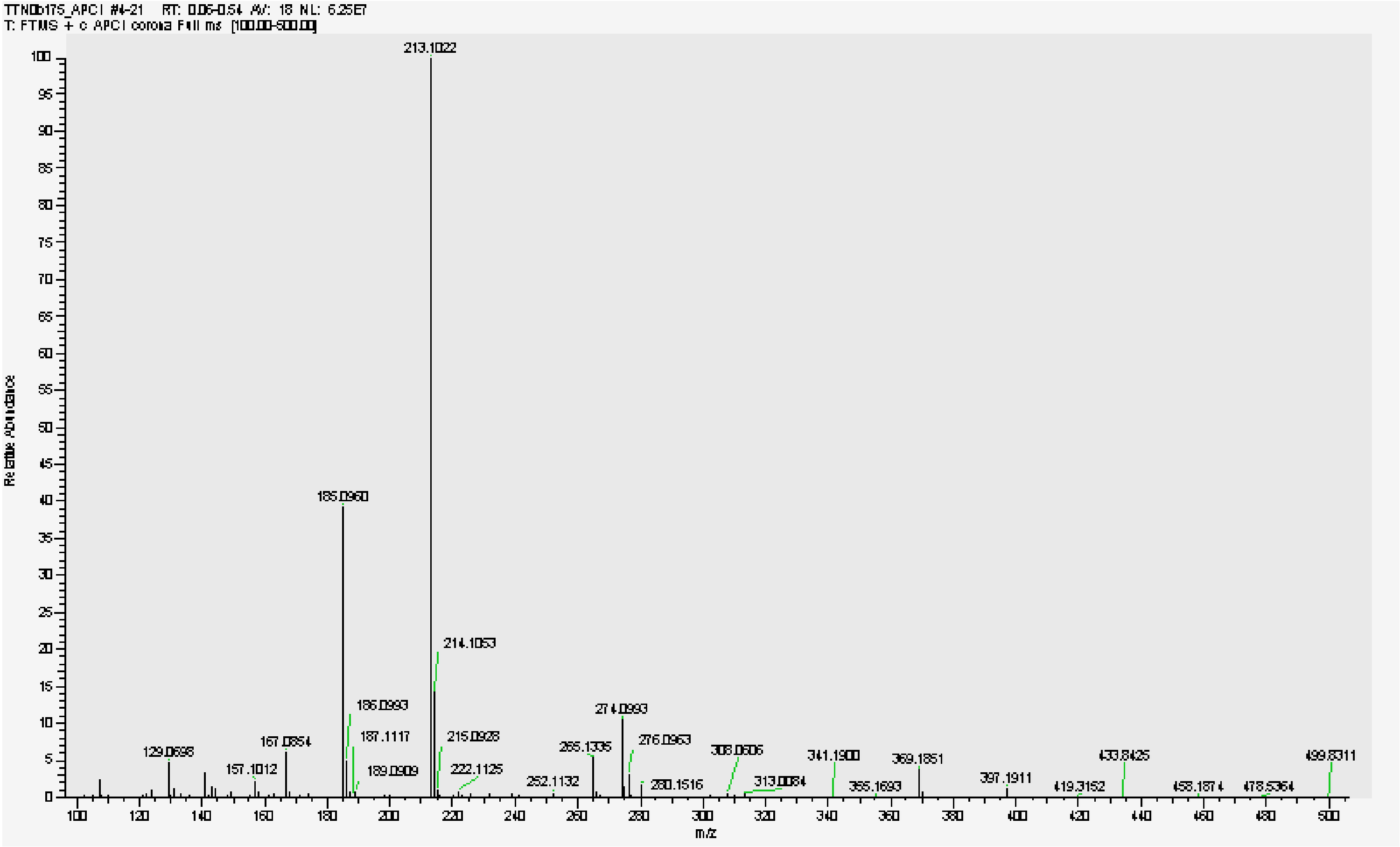

TTN0b175_APCI #4-21 RT: 0.06-0.54 AV: 18 NL: 6.25E7
T: FTMS + c APCI corona Full ms [100.00-500.00]
213.1022
185.0960
214.1053
186.0993
187.1117
189.0909
129.0698
157.1012
167.0854
215.0928
222.1125
252.1132
265.1335
274.0993
276.0963
280.1516
308.0606
313.0084
341.1900
355.1693
369.1851
397.1911
419.3152
433.8425
458.1874
478.5364
499.8311
Relative Abundance
m/z

IR

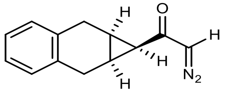
H
O
H
H
N2
H

S10

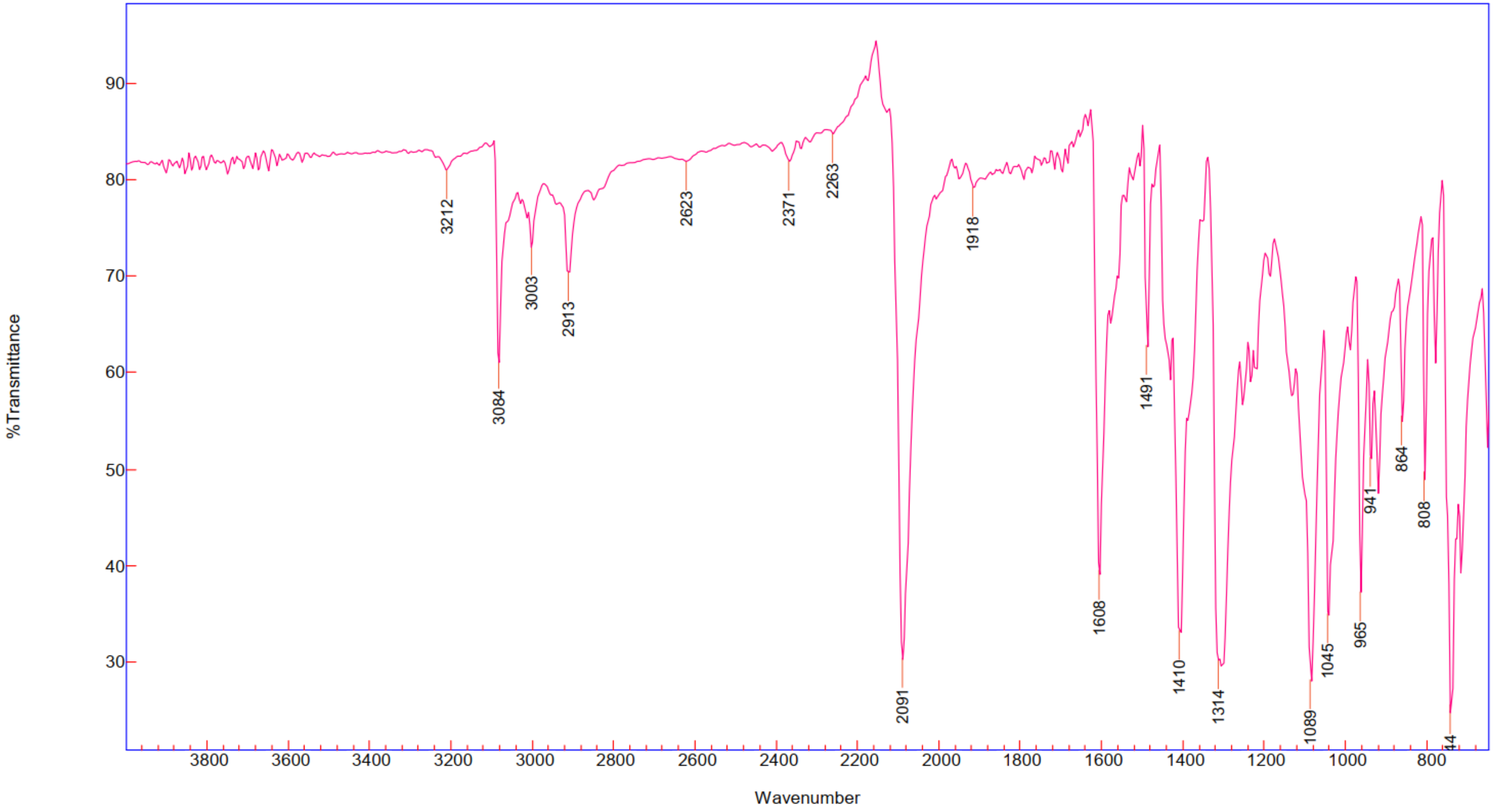
%Transmittance
Wavenumber
90
80
70
60
50
40
30
3800
3600
3400
3200
3000
2800
2600
2400
2200
2000
1800
1600
1400
1200
1000
800
3212
3084
3003
2913
2623
2371
2263
2091
1918
1608
1491
1410
1314
1089
1045
965
941
864
808

2.2.17 Triasterane S11

^{1}H NMR

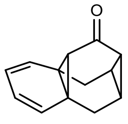

S11

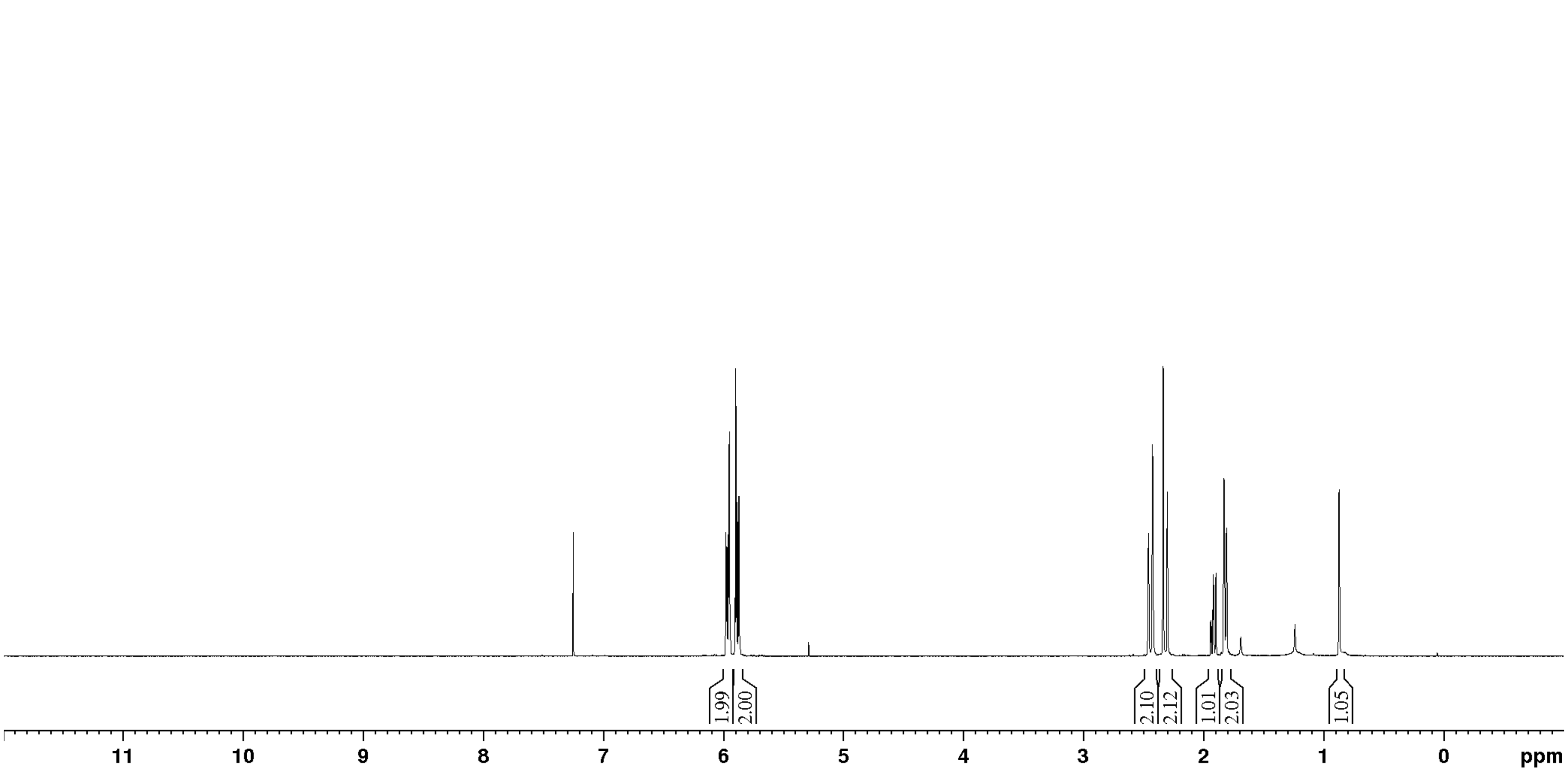

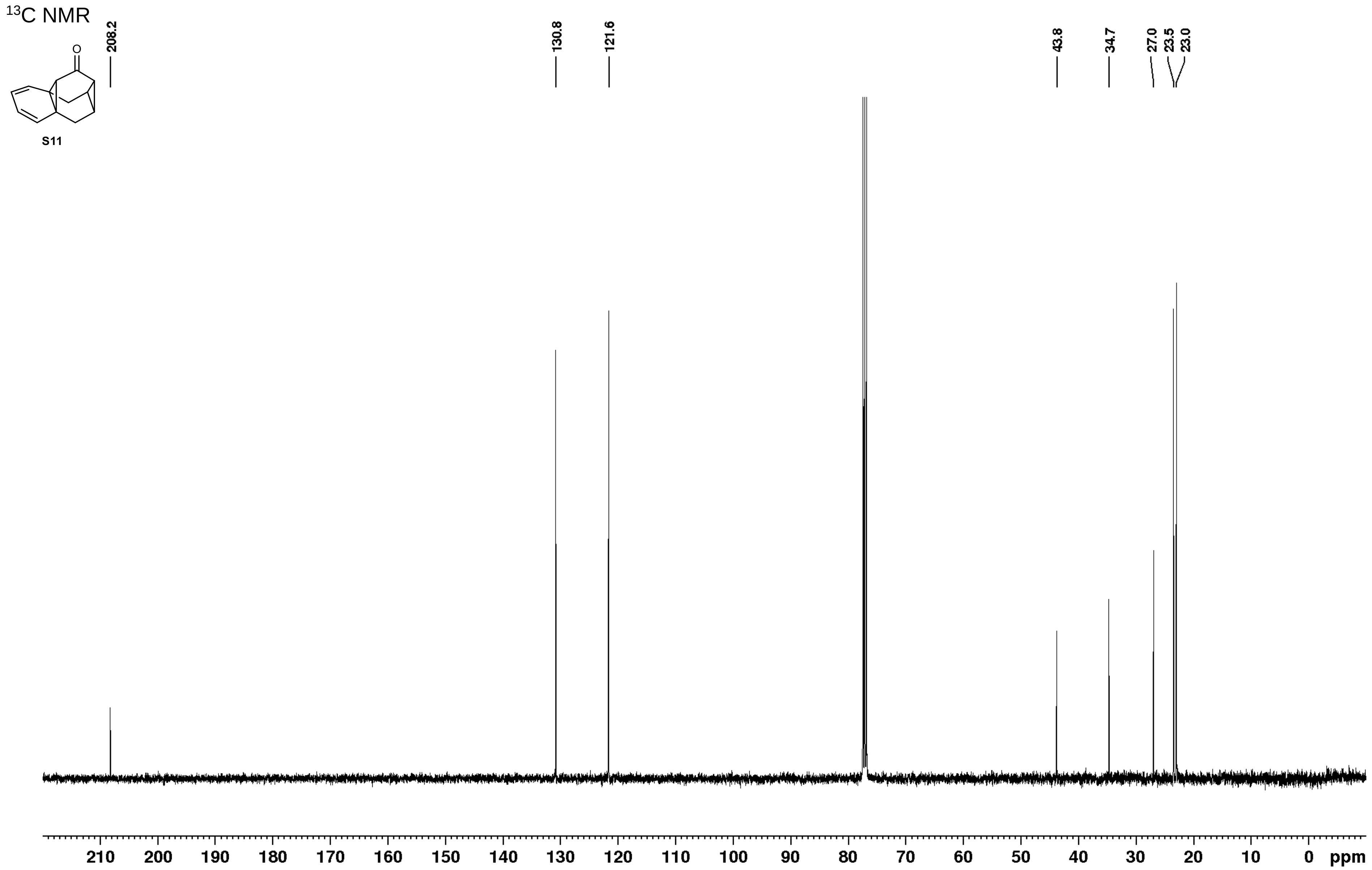

13C NMR
208.2
130.8
121.6
43.8
34.7
27.0
23.5
23.0
O
S11
210
200
190
180
170
160
150
140
130
120
110
100
90
80
70
60
50
40
30
20
10
0
ppm

^{1}H, ^{1}H COSY

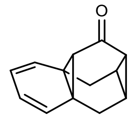

S11

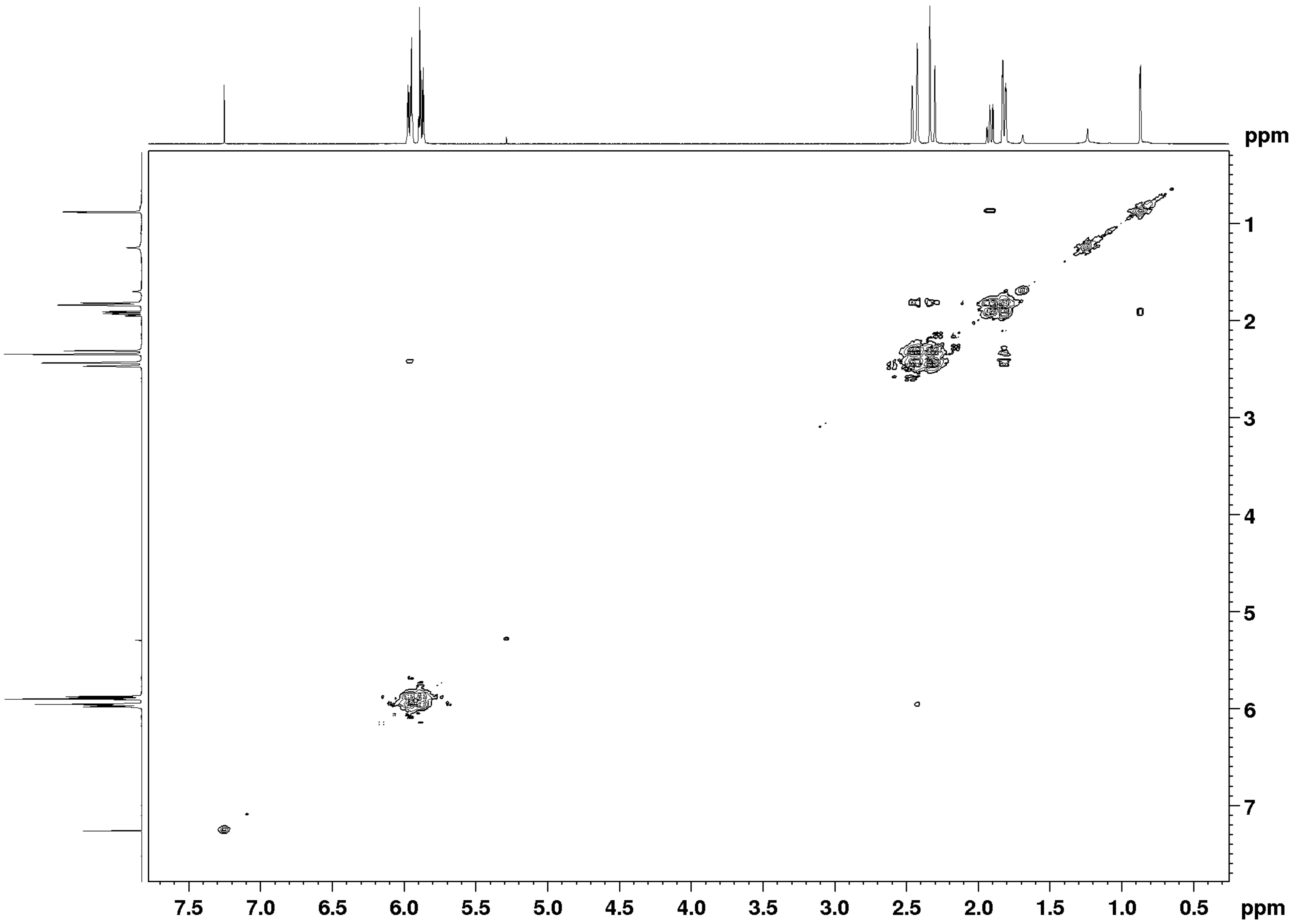

^{1}H, ^{13}C HSQC

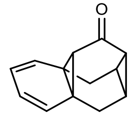

S11

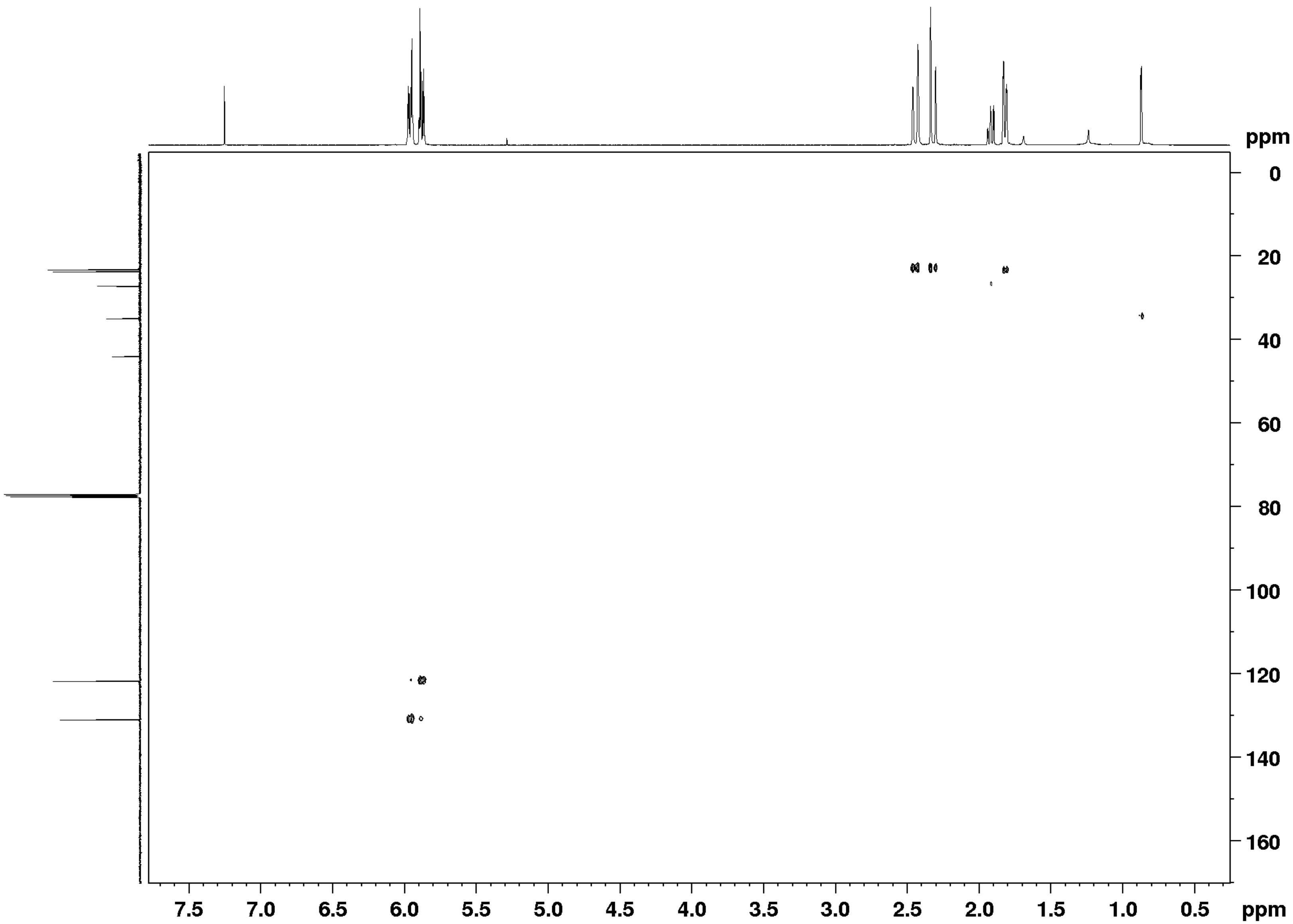

^{1}H, ^{13}C HMBC

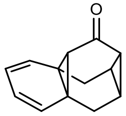

S11

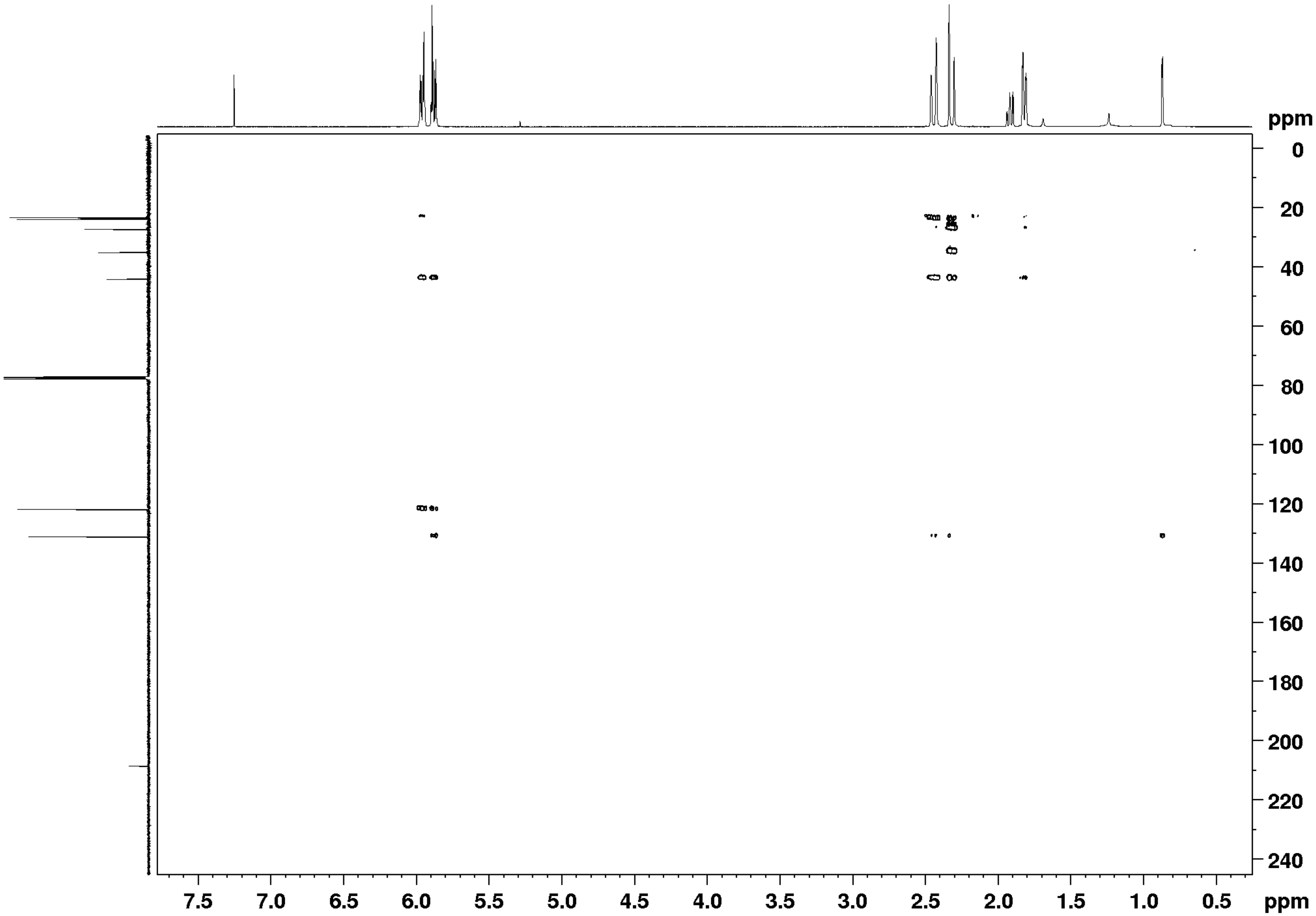

^1H, ^1H NOESY

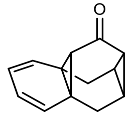

S11

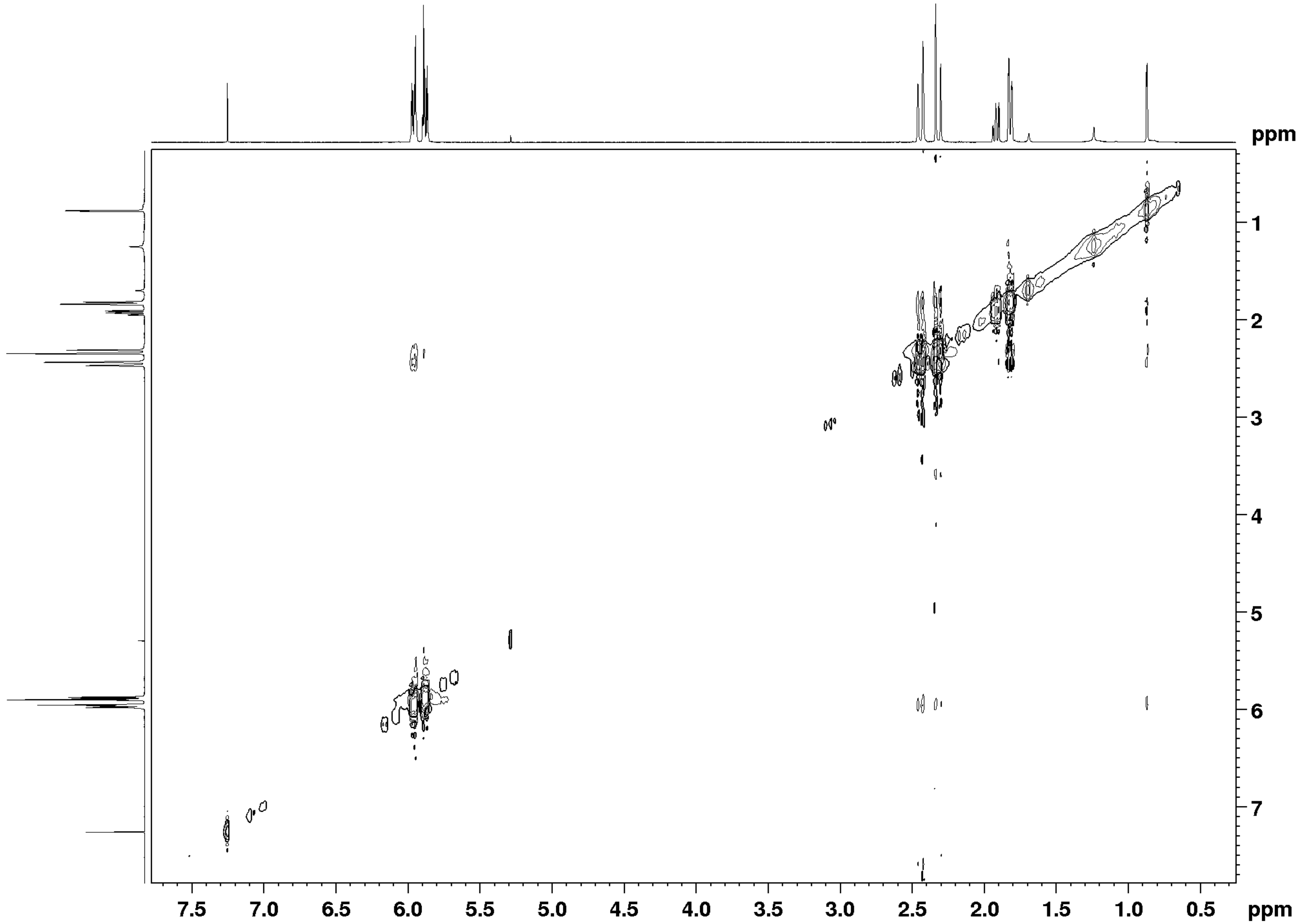

HRMS

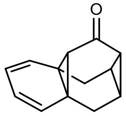

S11

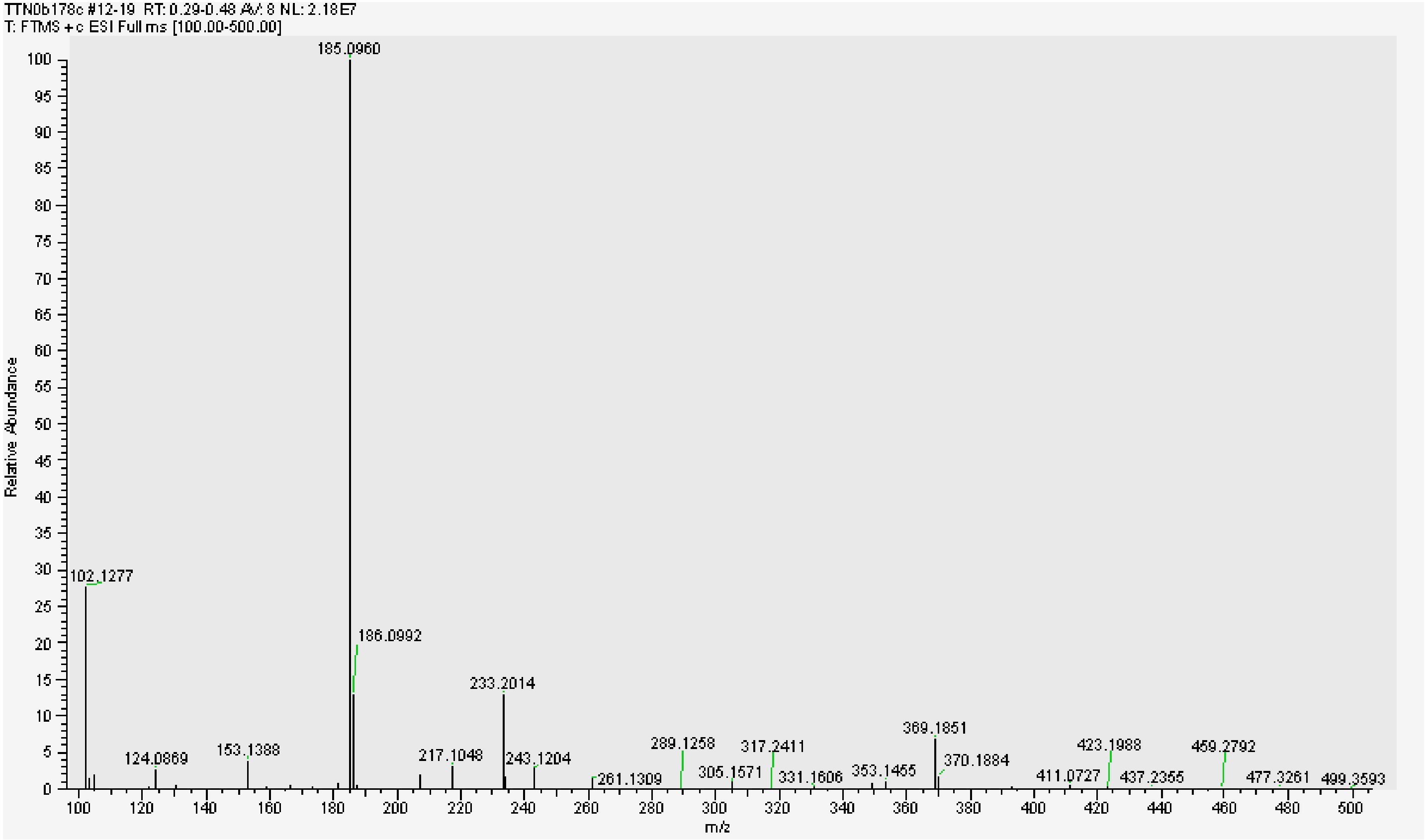

TTN0b178c #12-19 RT: 0.29-0.48 AV: 8 NL: 2.18E7
T: FTMS + c ESI Full ms [100.00-500.00]
185.0960
102.1277
186.0992
233.2014
369.1851
124.0869
153.1388
217.1048
243.1204
261.1309
289.1258
305.1571
317.2411
331.1606
353.1455
370.1884
411.0727
423.1988
437.2355
459.2792
477.3261
499.3593
Relative Abundance
m/z

IR

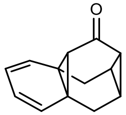

S11

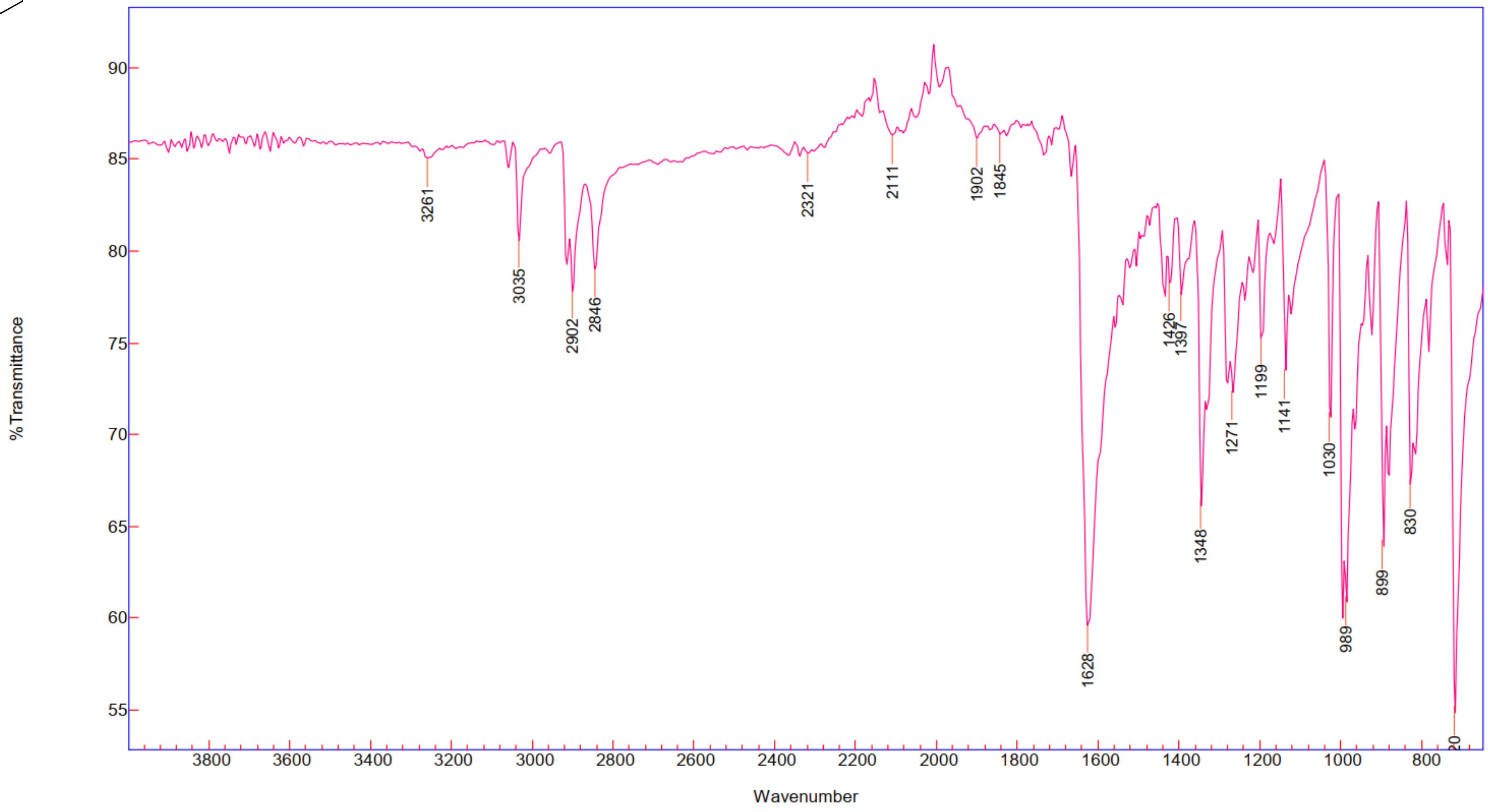

%Transmittance
Wavenumber
90
85
80
75
70
65
60
55
3800
3600
3400
3200
3000
2800
2600
2400
2200
2000
1800
1600
1400
1200
1000
800
3261
3035
2902
2846
2321
2111
1902
1845
1628
1426
1397
1348
1271
1199
1141
1030
989
899
830

2.2.18 Methyl-2-(2a1,2b,3,7b-tetrahydrobenzo[g]cyclopropa[cd]benzofuran-2(2a*H*)-ylidene)acetate (S12)

^{1}H NMR

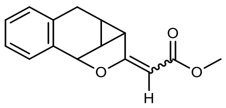

S12

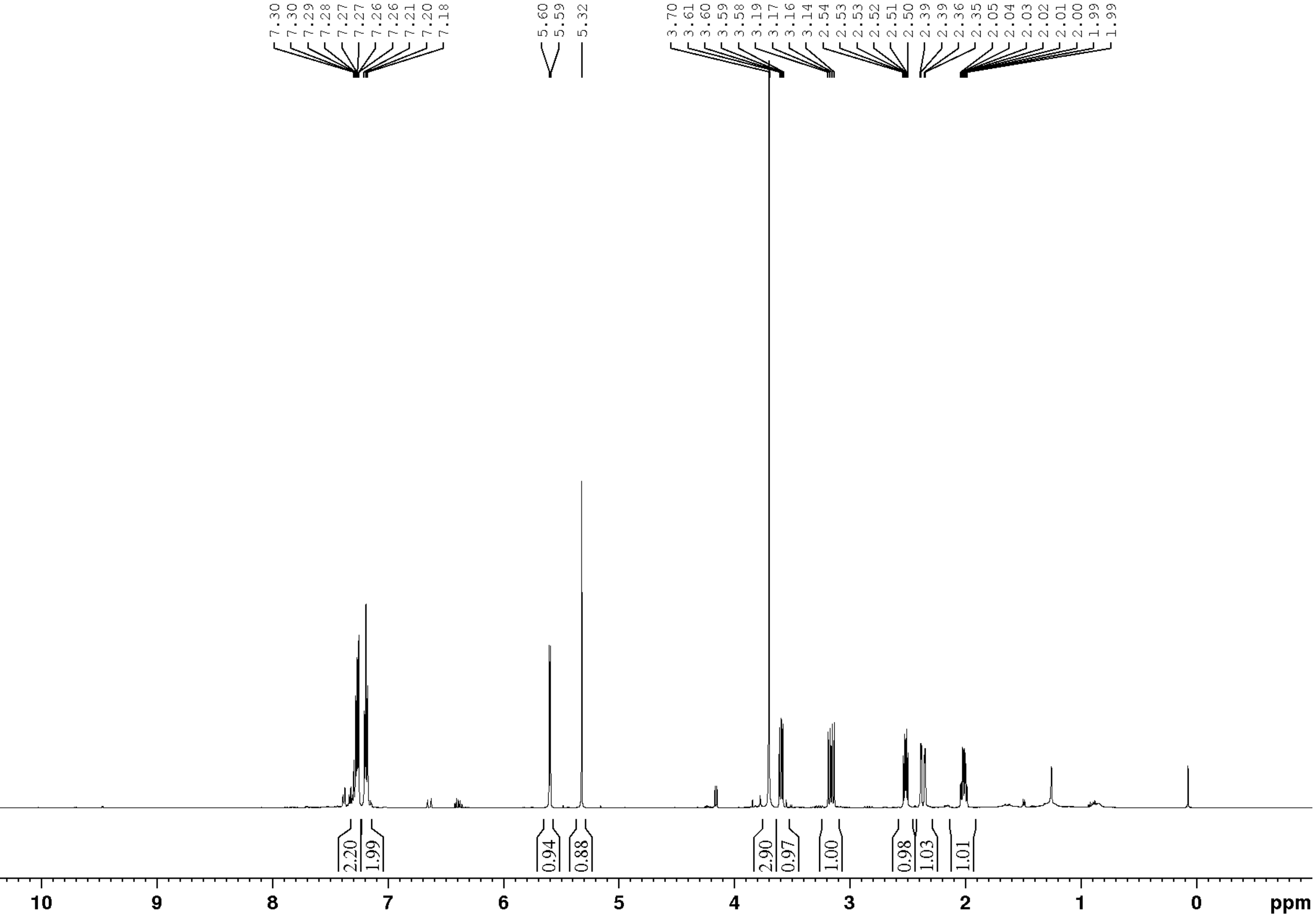

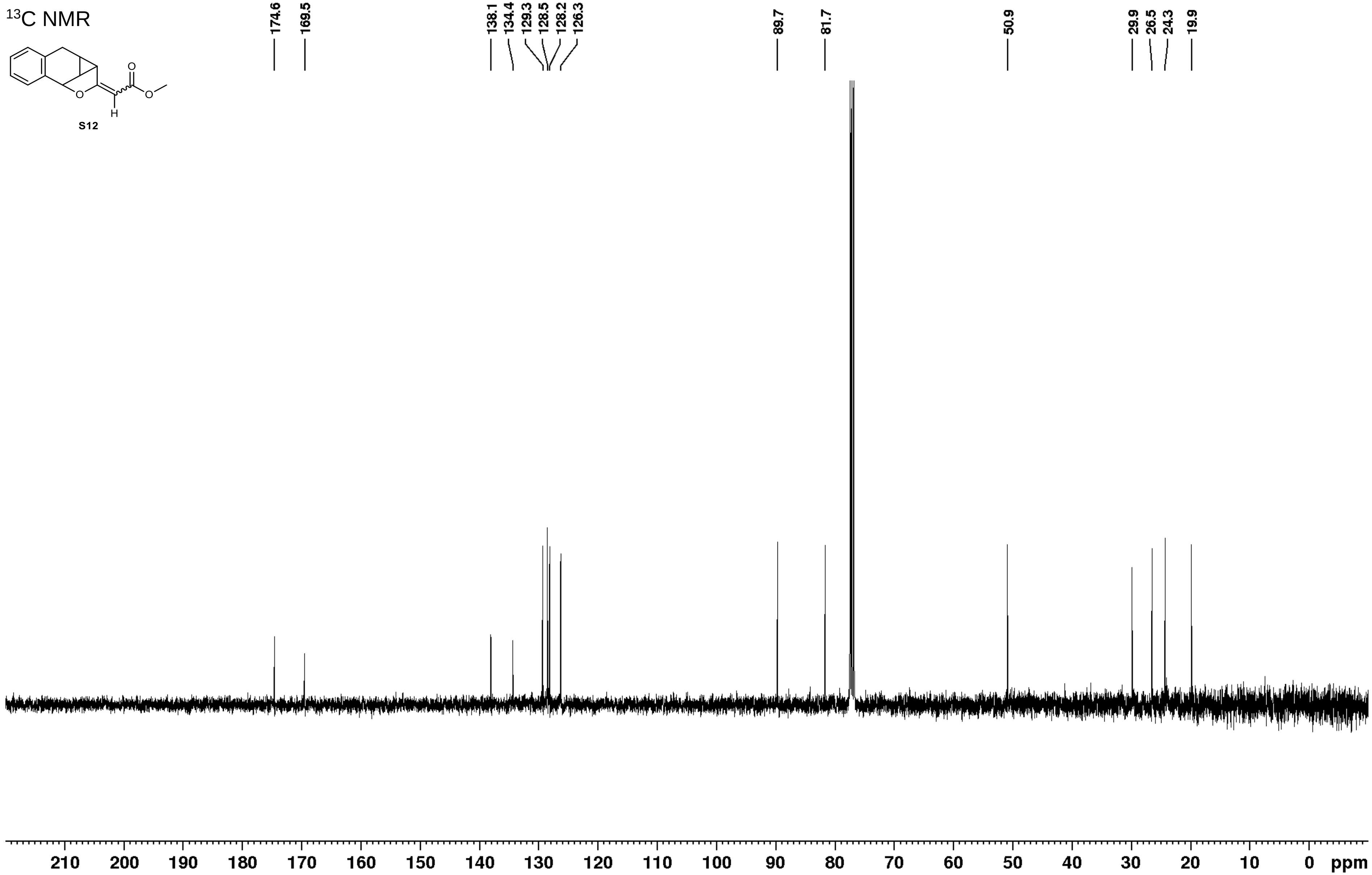
13C NMR
O
O
O
H
S12
174.6
169.5
138.1
134.4
129.3
128.5
128.2
126.3
89.7
81.7
50.9
29.9
26.5
24.3
19.9
210
200
190
180
170
160
150
140
130
120
110
100
90
80
70
60
50
40
30
20
10
0
ppm

^{1}H, ^{1}H COSY

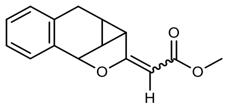

S12

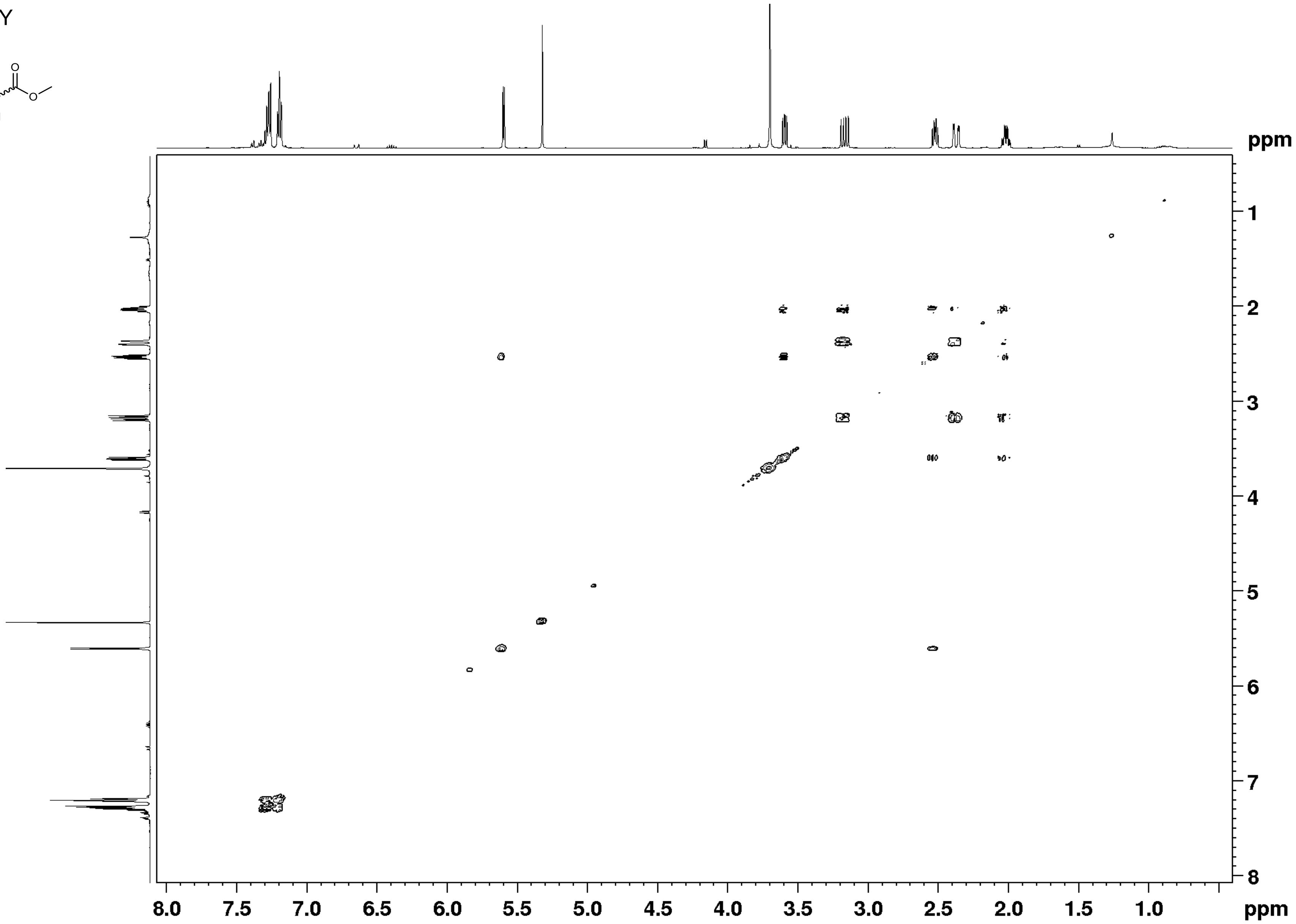

^{1}H, ^{13}C HSQC

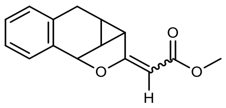

S12

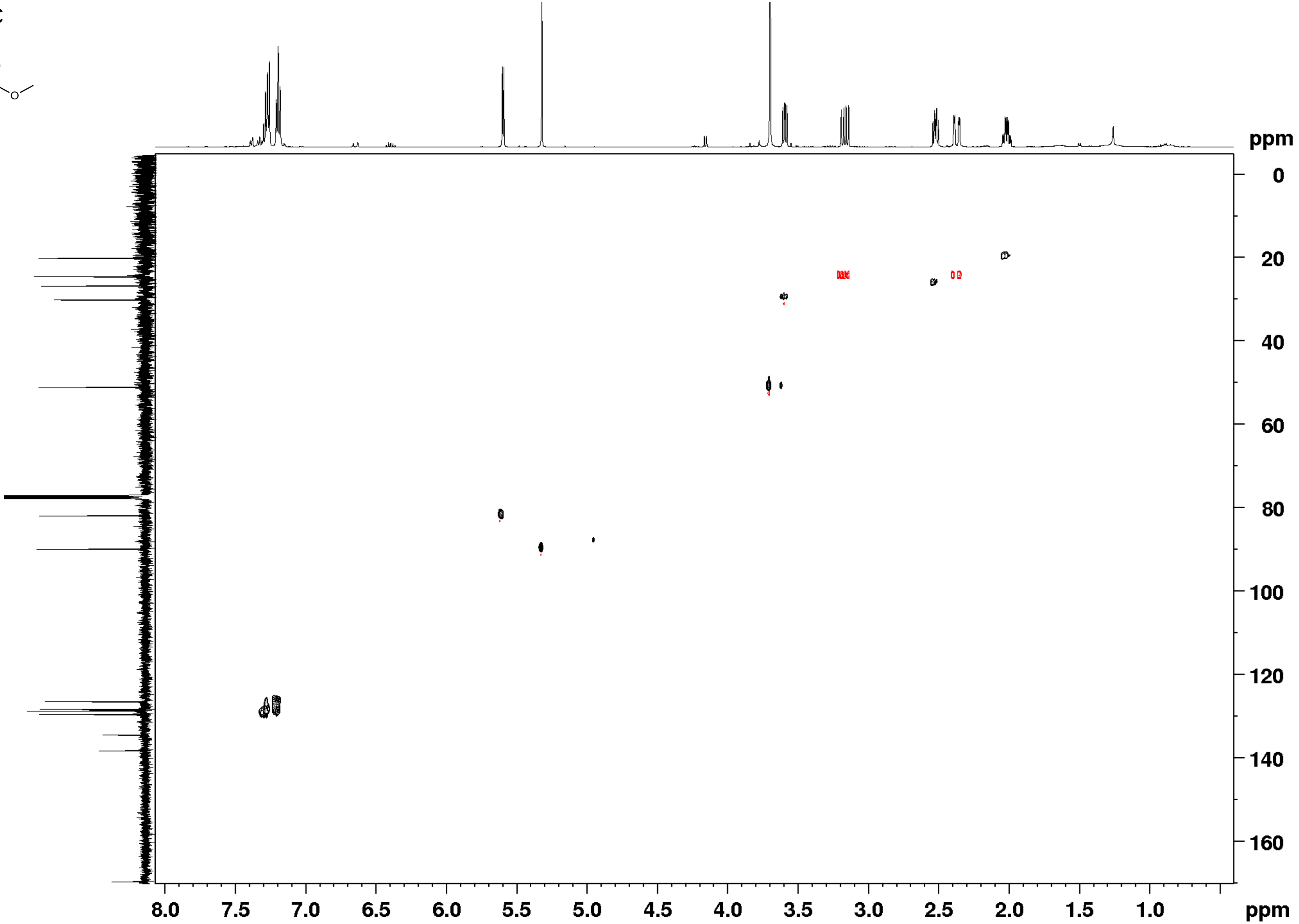

^1H, ^{13}C HMBC

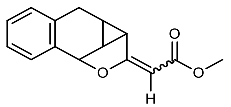

S12

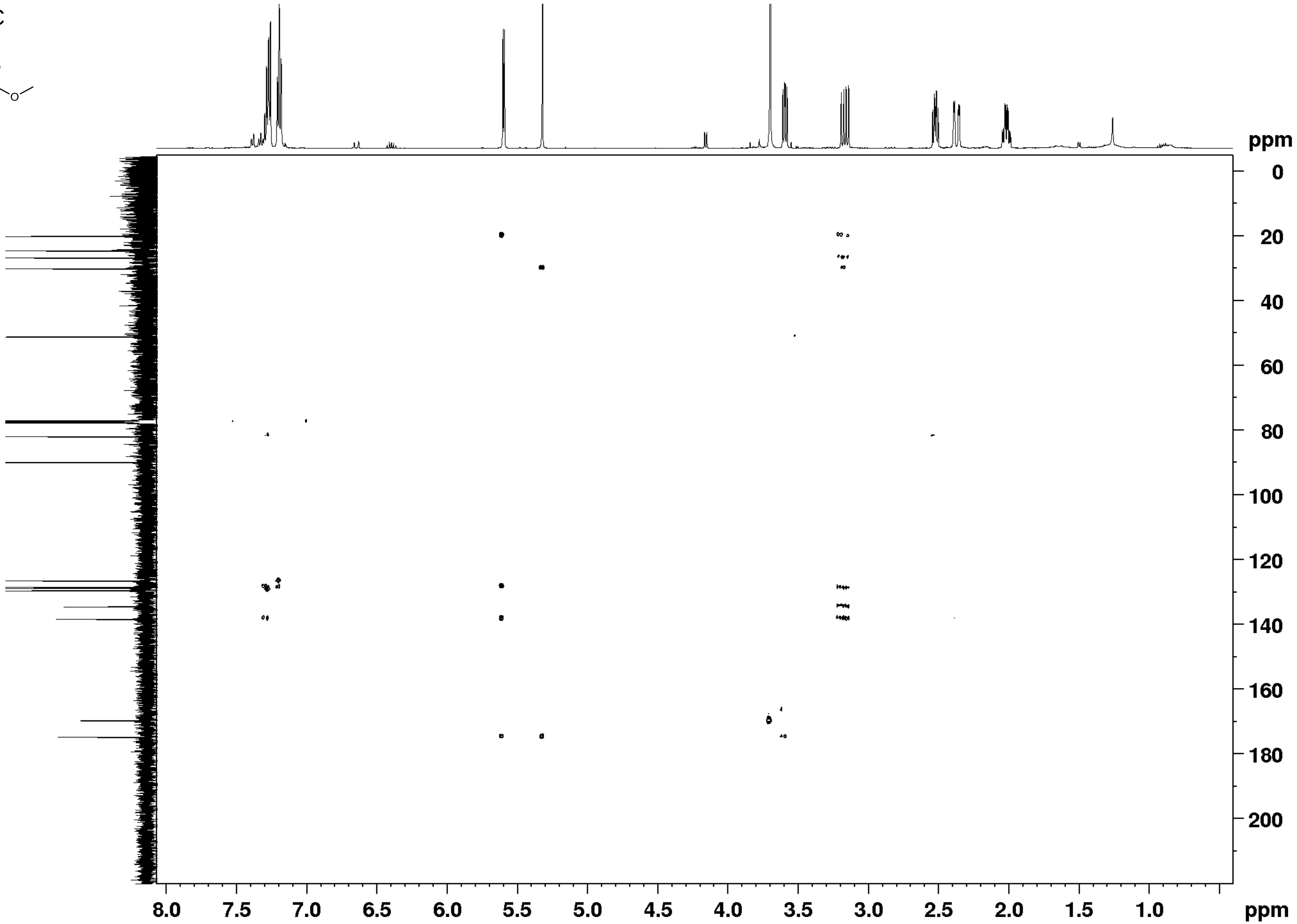

^1H, ^1H NOESY

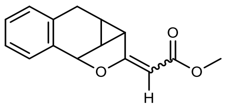

S12

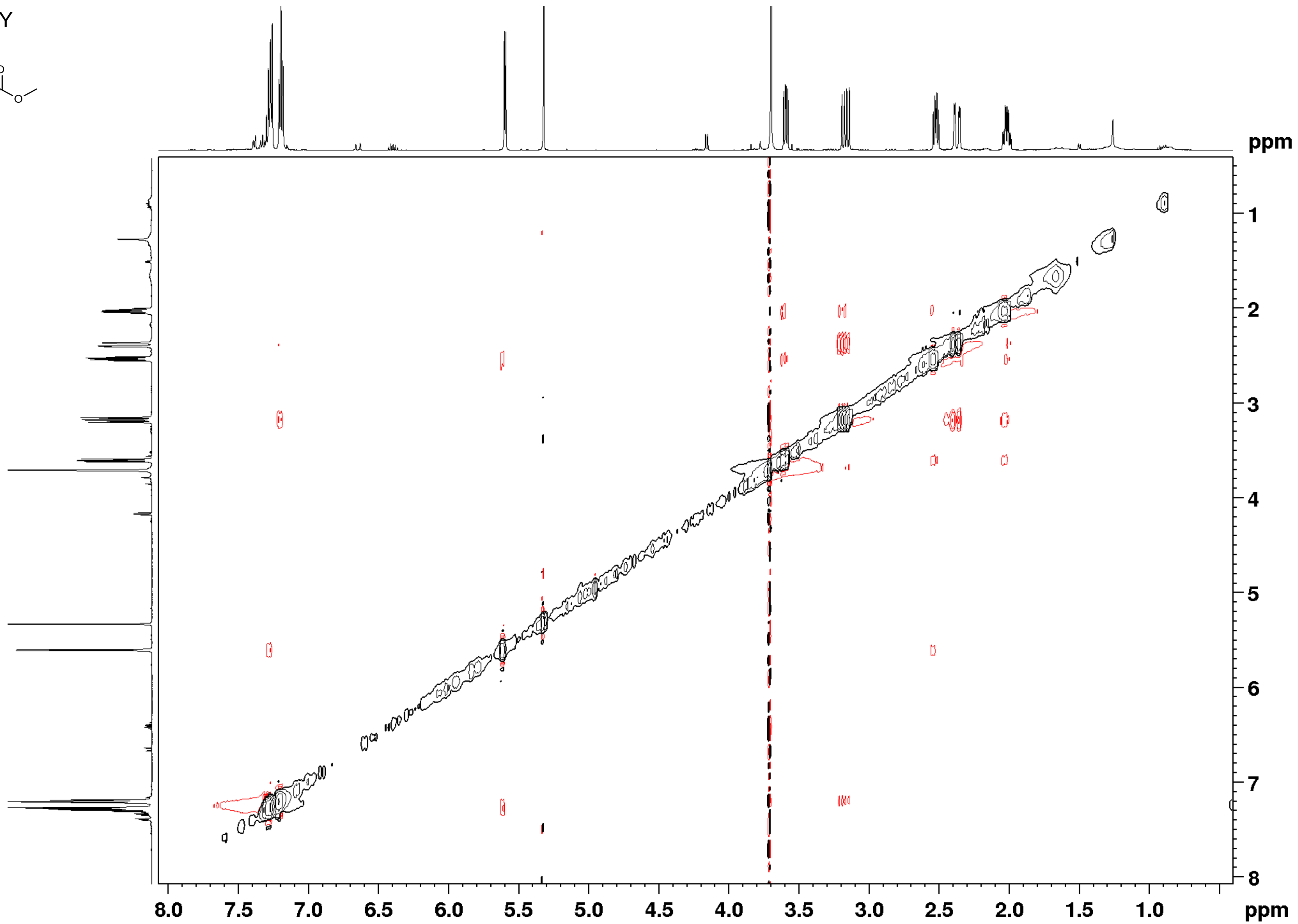

2.3 Atomic coordinates, methods and energies of calculated compounds, and TD-DFT results, template input files and fragmentation used for strain energy analysis

Energies in the following are Gibbs Energies: Δ*G*

Me-15
Energy (E) +Thermal free Energy Correction: –1617246.41869 kJ/mol
Method: B3LYP-D3
Basis set: def2-TZVP
XYZ-Coordinates:

C	2.24394800	-1.32745400	1.11421800
C	2.05513600	-2.07728500	-0.03176300
C	0.84187900	-2.19910900	-0.73622500
C	-0.21726700	-1.31959900	-0.70872700
C	0.09744600	-0.07010600	1.20071200
C	1.25357300	-0.56431700	1.76215300
C	-0.00058500	0.13474400	-0.30659700
C	-1.56733800	-1.78208700	-0.90983900
C	-1.08820400	0.12046600	1.99756300
C	-2.31080900	0.28273900	1.46120100
C	-2.64700800	-1.05179800	-0.57843400
C	-2.50741000	0.35336000	-0.03667000
C	1.19531200	0.82412300	-0.95476800
C	-1.25758600	0.94660000	-0.63101400
O	-1.25134400	1.95387300	-1.29152500
H	3.16840900	-1.49001900	1.65613300
H	2.84651800	-2.76842400	-0.29776900
H	0.67879200	-3.13869900	-1.25595800
H	1.35340400	-0.45938200	2.83852400
H	-1.69344200	-2.80776800	-1.23737500
H	-0.98356500	0.01169300	3.07107000
H	-3.19145600	0.31482800	2.08919100
H	-3.64175100	-1.47281800	-0.64291700
H	-3.36387000	0.97136000	-0.29989600
H	2.12753700	0.30696200	-0.76795700
H	1.03489900	0.88504300	-2.02998900
H	1.27320800	1.84092000	-0.57325700

15
Energy (E) +Thermal free Energy Correction: –2112465.95335 kJ/mol
Method: B3LYP-D3
Basis set: def2-TZVP
XYZ-Coordinates:

C	2.25801800	-1.25054900	1.09462000
C	2.07456100	-2.00016500	-0.04970100
C	0.84240700	-2.19660200	-0.70497000
C	-0.22622500	-1.33908500	-0.67757400
C	0.07273500	-0.09614700	1.24277600
C	1.23583100	-0.57257300	1.79277700
C	-0.01158300	0.10694700	-0.25886500
C	-1.57673900	-1.78669100	-0.89910200
C	-1.12566800	0.10714600	2.01611000

C	-2.33323900	0.29576400	1.45522400
C	-2.65336300	-1.04336000	-0.59193600
C	-2.50507500	0.36222200	-0.04872900
C	1.20232900	0.75608200	-0.96917200
O	1.59764000	0.34975600	-2.02244500
O	1.79748500	1.82780200	-0.41955200
C	1.29312900	2.56848900	0.69393900
C	-1.24310100	0.94148900	-0.62248000
O	-1.19296600	1.94876500	-1.28010200
H	3.21412500	-1.34851200	1.59478700
H	2.90324600	-2.62322400	-0.36346200
H	0.70303300	-3.14142200	-1.22003800
H	1.33979500	-0.49997700	2.87119900
H	-1.70589400	-2.80969100	-1.23230200
H	-1.04356300	-0.00349800	3.09126500
H	-3.22483100	0.35050600	2.06557000
H	-3.65290700	-1.44747500	-0.67930600
H	-3.34800400	0.99125600	-0.32834000
H	1.70350600	3.56943400	0.58077400
H	0.20833900	2.63388300	0.68745200
H	1.63825500	2.13130000	1.62928800

H-15
Energy (E) +Thermal free Energy Correction: –1514055.88811 kJ/mol
Method: B3LYP-D3
Basis set: def2-TZVP
XYZ-Coordinates:

C	2.25502800	-1.22827700	1.04655000
C	2.06639500	-1.97733600	-0.09813000
C	0.82246200	-2.23503600	-0.72711100
C	-0.26453200	-1.40593400	-0.66619700
C	0.05507500	-0.13683200	1.27321300
C	1.23817500	-0.58435700	1.79540800
C	-0.03138000	0.02027800	-0.22660300
C	-1.62493400	-1.82252700	-0.87906600
C	-1.14469800	0.08465600	2.03547000
C	-2.33612300	0.30469200	1.44800000
C	-2.67155500	-1.02715000	-0.58754600
C	-2.48104300	0.38285000	-0.06033800
C	-1.18095000	0.90968700	-0.61932400
O	-1.07256700	1.90551100	-1.28932500
H	3.23232300	-1.29746500	1.51075400
H	2.91234900	-2.56806900	-0.43094700
H	0.70748800	-3.19374000	-1.22327900
H	1.38194500	-0.51564400	2.86924400
H	-1.79629300	-2.84238200	-1.20341700
H	-1.08519300	-0.01786800	3.11278800
H	-3.23806400	0.38107900	2.04117700
H	-3.68547200	-1.39549900	-0.67402500
H	-3.30394100	1.03327200	-0.35031900
H	0.90149300	0.40533700	-0.63231200

Me-18
Energy (E) +Thermal free Energy Correction: –1617677.41293 kJ/mol
Method: B3LYP-D3
Basis set: def2-TZVP
XYZ-Coordinates:

C	-0.99294600	-0.37747500	0.41167400
C	-0.98037200	-1.11075500	-0.83427100
C	-1.92377200	-1.12970100	-1.80527500
C	-3.09997700	-0.27848600	-1.81757900
C	-2.96474600	1.10862800	0.10344600
C	-1.89766600	0.52333600	0.87030700
C	-2.88075100	1.17534800	-1.41006700
C	-4.32274100	-0.85320800	-1.96828100
C	-4.16953600	1.55200700	0.53750700
C	-5.07690200	1.92263800	-0.60615100
C	-5.61902900	-0.39095000	-1.53484400
C	-5.97630800	0.74060300	-0.88863100
C	-1.62723800	1.81223100	-2.00266400
C	-4.09868400	2.03531100	-1.76199200
O	-4.23349400	2.69929100	-2.75206400
H	-0.22070800	-0.67682100	1.11133100
H	-0.17065900	-1.82456600	-0.93231900
H	-1.85117500	-1.88974600	-2.57574300
H	-1.82237900	0.82732000	1.90869300
H	-4.31216600	-1.89209300	-2.28309900
H	-4.50228500	1.52028500	1.56659000
H	-5.65601000	2.83615400	-0.47736800
H	-6.40638900	-1.12613200	-1.65932000
H	-6.99593300	0.81642300	-0.53019900
H	-0.72521000	1.27836400	-1.72291600
H	-1.70822100	1.82858300	-3.08910800
H	-1.54540200	2.84268000	-1.65585400

18
Energy (E) +Thermal free Energy Correction: –2112409.60749 kJ/mol
Method: B3LYP-D3
Basis set: def2-TZVP
XYZ-Coordinates:

C	-0.97940000	-0.33486000	0.43588800
C	-0.95806600	-1.06553900	-0.81046500
C	-1.90838100	-1.10767200	-1.77053500
C	-3.09680800	-0.27372300	-1.78850300
C	-2.98100800	1.10113200	0.13629600
C	-1.90921700	0.53251900	0.90703600
C	-2.88419400	1.17746900	-1.37601800
C	-4.31442000	-0.84795600	-1.95522200
C	-4.19852500	1.52514400	0.55168000
C	-5.08626700	1.91925300	-0.59768800
C	-5.62042500	-0.38268700	-1.55783100
C	-5.98795000	0.74790400	-0.91794900
C	-1.63724700	1.75453000	-2.07141600
O	-1.19642400	1.25804900	-3.06791000
O	-1.06229600	2.87635900	-1.60174700
C	-1.56593000	3.65564900	-0.51649300
C	-4.09195800	2.06475100	-1.73485900
O	-4.19419100	2.76230200	-2.70248800

H	-0.19513700	-0.61571200	1.12970400
H	-0.12579700	-1.74958500	-0.92193000
H	-1.81706800	-1.84741200	-2.55687800
H	-1.85195100	0.82380500	1.94994400
H	-4.29376100	-1.88783300	-2.26469100
H	-4.55055000	1.46724000	1.57261200
H	-5.66481100	2.83208200	-0.45960800
H	-6.40835300	-1.11193000	-1.70887500
H	-7.01624400	0.83311500	-0.58841200
H	-1.00403100	4.58576800	-0.55200600
H	-2.62478600	3.88011800	-0.63509600
H	-1.39156000	3.15864200	0.43621700

H-18
Energy (E) +Thermal free Energy Correction: –1513994.945 kJ/mol
Basis set: def2-TZVP
XYZ-Coordinates:

C	-0.96350400	-0.31518000	0.37486500
C	-0.95010200	-1.03695800	-0.88013000
C	-1.93406600	-1.14092400	-1.80849900
C	-3.13567500	-0.33749000	-1.79546200
C	-3.00350000	1.06396300	0.14440500
C	-1.91156400	0.51033300	0.89058900
C	-2.91914400	1.09601200	-1.35963500
C	-4.36203000	-0.89624700	-1.96181900
C	-4.20255100	1.54660200	0.55518900
C	-5.07392800	1.92854200	-0.61728600
C	-5.64806900	-0.39242600	-1.54360700
C	-5.98502700	0.75676800	-0.91583500
C	-4.05183300	2.03008700	-1.74116500
O	-4.10798800	2.73316000	-2.71183600
H	-0.14982600	-0.58037200	1.04069100
H	-0.10117500	-1.69943900	-1.00364200
H	-1.85273000	-1.91849500	-2.56041200
H	-1.83005200	0.78874500	1.93541600
H	-4.37199600	-1.93104400	-2.28864300
H	-4.54676800	1.56529500	1.58056300
H	-5.64409400	2.84913100	-0.50235000
H	-6.45584100	-1.10495400	-1.66973800
H	-7.00812000	0.85524600	-0.57259800
H	-1.96674500	1.45903200	-1.74235200

Cycloheptatriene
Energy (E) +Thermal free Energy Correction: –712929.831345 kJ/mol
Method: B3LYP-D3
Basis set: def2-TZVP
XYZ-Coordinates:

C	1.35157773	0.00000000	-0.04110105
C	0.66863618	-1.17459592	-0.04110469
C	-0.76459070	-1.31502790	-0.00000412
C	0.76459584	1.31503419	0.00000408
C	-1.64520519	-0.44797786	-0.52876742
C	-0.42456672	1.65140797	-0.52875884
C	-1.20266213	0.69926768	-1.39178231
H	2.42930645	-0.05277101	0.07178262

H	1.24765436	-2.08510288	0.07177647
H	-1.14736923	-2.18580714	0.52277064
H	1.33199239	2.07846761	0.52278469
H	-2.70496021	-0.58066091	-0.34381173
H	-0.83352452	2.63803778	-0.34379687
H	-0.55698979	0.32385869	-2.19412441
H	-2.05594148	1.19538764	-1.85150322

Ellassovalene:
Energy (E) +Thermal free Energy Correction: –1216399.03157 kJ/mol
Method: B3LYP-D3
Basis set: def2-TZVP
XYZ-Coordinates:

C	1.22320000	0.00000000	-0.00000259
C	1.54674801	1.29726742	-0.24460293
C	0.68685825	2.41437601	-0.00006693
C	-0.68663850	2.41433237	0.00006903
C	-1.54672052	1.29731705	-0.24439379
C	-1.22320457	-0.00000171	0.00000554
C	-1.89905641	-1.20571111	-0.42455369
C	-1.24888472	-2.30814363	-0.00382482
C	0.00019139	-1.96381013	0.78087975
C	1.24972273	-2.30809099	-0.00293664
C	1.89958675	-1.20557931	-0.42399241
C	0.00003954	-0.40447590	0.79072574
H	2.51473768	1.51206489	-0.69087339
H	-2.51439472	1.51223890	-0.69043751
H	-2.79334205	-1.20318650	-1.03610107
H	-1.53605448	-3.32836820	-0.22627990
H	-0.00045843	-2.41434500	1.77899048
H	1.53740638	-3.32830420	-0.22450385
H	2.79391827	-1.20268988	-1.03524774
H	0.00011547	0.04383788	1.78757748
H	-1.16772061	3.38739267	0.04528972
H	1.16789843	3.38742534	0.04522118

Norcaradiene:
Energy : –712893.531179 kJ/mol
Method: B3LYP-D3
Basis set: def2-TZVP
XYZ-Coordinates:

C	1.45811516	0.00000000	-0.06362797
C	0.71284241	-1.25888626	0.00000001
C	-0.63065236	-1.29557436	-0.04763795
C	-1.42159584	-0.08632487	0.04837202
C	-0.84263419	1.11934180	0.18875941
C	0.60165863	1.30932043	0.04011429
C	1.28744867	0.92024419	-1.23721028
H	2.43378750	0.00940637	0.40576442
H	1.28084819	-2.17407049	0.11532077
H	-1.14497048	-2.24816479	-0.03397168
H	-2.49696662	-0.18114107	0.13030570
H	-1.43986665	1.98584996	0.44559757
H	1.03785957	2.14371315	0.57479219
H	0.66231257	0.57560122	-2.04951704
H	2.14781647	1.50619658	-1.52850104

Benzene:
Energy: –609860.39146kJ/mol
Method: B3LYP-D3
Basis set: def2-TZVP
XYZ-Coordinates:

C	1.39018413	0.00000000	-0.00014980
C	0.69508158	1.20395173	-0.00014442
C	-0.69511151	1.20393075	0.00000637
C	-1.39018499	-0.00000558	0.00015146
C	-0.69510743	-1.20391400	0.00015176
C	0.69510120	-1.20391292	-0.00000439
H	2.47265077	0.00002858	-0.00026899
H	1.23634528	2.14137844	-0.00025867
H	-1.23637695	2.14135512	0.00000615
H	-2.47265145	0.00003753	0.00026939
H	-1.23630982	-2.14137691	0.00026992
H	1.23630517	-2.14137280	-0.00000781

TD-DFT results
15
Level of Theory: cam-B3LYP-D3/def2tzvp Solvent: SMD(MeCN)

```
Excited State   1:      Singlet    3.6555 eV  339.17 nm  f=0.1373  <S**2>=0.000
      62 -> 65        0.10140
      63 -> 64        0.69332
Excited State   2:      Singlet    3.9910 eV  310.66 nm  f=0.1016  <S**2>=0.000
      62 -> 64        0.62034
      63 -> 65       -0.32042
Excited State   3:      Singlet    4.2126 eV  294.32 nm  f=0.0153  <S**2>=0.000
      59 -> 66        0.15939
      61 -> 66       -0.36795
      63 -> 66        0.56464
Excited State   4:      Singlet    4.6249 eV  268.08 nm  f=1.4129  <S**2>=0.000
      62 -> 64        0.31808
      63 -> 65        0.62178
Excited State   5:      Singlet    5.0982 eV  243.19 nm  f=0.2101  <S**2>=0.000
      62 -> 65        0.68832
Excited State   6:      Singlet    5.2472 eV  236.29 nm  f=0.0012  <S**2>=0.000
      57 -> 66       -0.12629
      59 -> 66       -0.18061
      61 -> 64        0.13416
      61 -> 66        0.47751
      63 -> 66        0.40938
Excited State   7:      Singlet    5.4277 eV  228.43 nm  f=0.0493  <S**2>=0.000
      62 -> 66        0.68748
Excited State   8:      Singlet    5.5180 eV  224.69 nm  f=0.1177  <S**2>=0.000
      61 -> 64        0.67339
      61 -> 66       -0.12337
Excited State   9:      Singlet    5.6764 eV  218.42 nm  f=0.0075  <S**2>=0.000
      60 -> 64       -0.37675
      63 -> 67        0.56237
      63 -> 68        0.12511
 Excited State  10:      Singlet    5.7561 eV  215.39 nm  f=0.1120  <S**2>=0.000
      59 -> 64       -0.12153
```

60 -> 64 0.50102
63 -> 67 0.41035
63 -> 68 -0.20132

18
Level of Theory: cam-B3LYP-D3/def2tzvp Solvent: SMD(MeCN)

Excited State 1: Singlet 3.2179 eV 385.30 nm f=0.1825 <S**2>=0.000
63 -> 64 0.69584
Total Energy, E(TD-HF/TD-DFT) = -804.235515510
Excited State 2: Singlet 4.0949 eV 302.78 nm f=0.0573 <S**2>=0.000
60 -> 65 0.14489
60 -> 66 0.19426
61 -> 65 -0.12524
61 -> 66 -0.17090
62 -> 64 -0.26280
62 -> 65 0.15964
62 -> 66 0.22000
63 -> 65 0.28861
63 -> 66 0.36447
Excited State 3: Singlet 4.1705 eV 297.29 nm f=0.0259 <S**2>=0.000
62 -> 64 0.50772
63 -> 65 0.45735
63 -> 66 -0.10179
Excited State 4: Singlet 4.4602 eV 277.98 nm f=0.7525 <S**2>=0.000
62 -> 64 0.39648
63 -> 65 -0.35706
63 -> 66 0.41759
Excited State 5: Singlet 4.7569 eV 260.64 nm f=0.0079 <S**2>=0.000
60 -> 64 0.10431
60 -> 65 -0.22211
60 -> 66 -0.30605
61 -> 66 0.11966
62 -> 65 -0.22062
62 -> 66 -0.19552
63 -> 65 0.22947
63 -> 66 0.39700
Excited State 6: Singlet 5.1019 eV 243.01 nm f=0.2291 <S**2>=0.000
60 -> 64 0.15924
61 -> 64 0.65533
63 -> 65 -0.11250
Excited State 7: Singlet 5.3040 eV 233.76 nm f=0.1635 <S**2>=0.000
60 -> 66 -0.12241
61 -> 65 0.10511
61 -> 66 0.10541
62 -> 65 0.61947
62 -> 66 -0.21862
Excited State 8: Singlet 5.4257 eV 228.51 nm f=0.0365 <S**2>=0.000
60 -> 64 0.55050
61 -> 64 -0.11004
61 -> 65 0.11306
62 -> 66 0.34051
63 -> 67 0.14272
Excited State 9: Singlet 5.5150 eV 224.81 nm f=0.0102 <S**2>=0.000

60 -> 64 0.10744
61 -> 65 -0.14584
61 -> 66 -0.18089
62 -> 66 -0.32193
63 -> 67 0.53793
Excited State 10: Singlet 5.5621 eV 222.91 nm f=0.0243 <S**2>=0.000
60 -> 64 -0.34511
60 -> 65 -0.10798
60 -> 66 -0.21429
61 -> 66 0.15382
62 -> 66 0.32398
63 -> 67 0.39518

Templates for Input Files

Geometry Optimization

```
-----------------------------------------------------------------------------------------------------------------
%chk=cal137.chk
%nprocshared=10
%mem=10GB
# opt freq=noraman b3lyp def2tzvp empiricaldispersion=gd3bj nosymmetry output=wfn

cal137

0 1
####Coordinates####
cal137.wfn
```

ACID Calculation

```
-----------------------------------------------------------------------------------------------------------------
nprocshared=10
%mem=10GB
# b3lyp def2tzvp empiricaldispersion=gd3bj scf=tight nmr=csgt iop(10/93=1)

AICD022 from cal137

0 1
####Coordinates####
AICD022.txt

-----------------------------------------------------------------------------------------------------------------
```

NICS-XY-Scan/ Plane

```
%chk=cal152.chk
%nprocshared=10
%mem=10GB
# nmr=giao b3lyp/6-311g(d,p)

cal152

0 1
####Coordinates####

--------------------------------------------------------------------------------------------------------------------
```

Fragmentation of **H-15**, **H-18** and elassovalene for StrainViz Calculations.

	**H-15**	**H-18**	Elassovalene
Geometry optimized structure	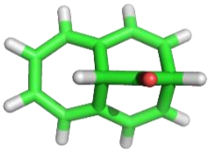	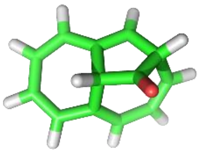	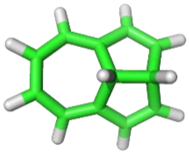
Fragment 1	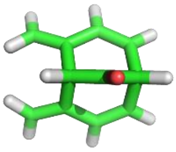	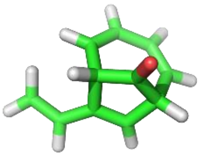	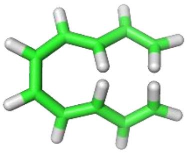
Fragment 2	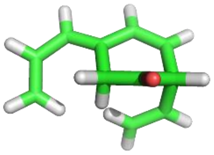	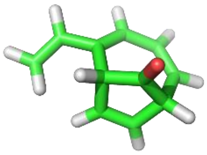	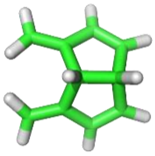
Fragment 3	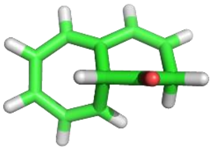	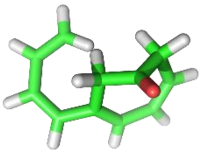	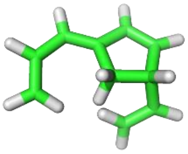
Fragment 4	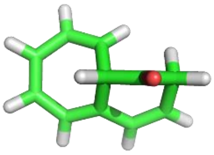	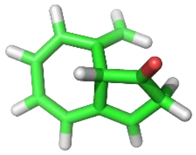	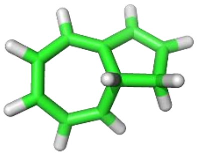
Fragment 5	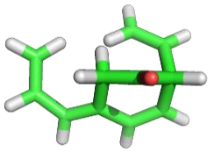	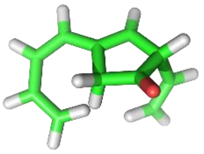	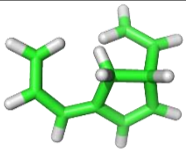
Fragment 6	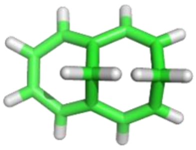	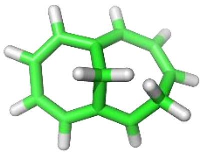	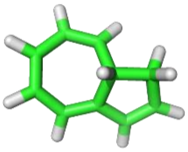

3 References

References

1. Fulmer, G. R., Miller, A. J. M., Sherden, N. H., Gottlieb, H. E., Nudelman, A., Stoltz, B. M., Bercaw, J. E. & Goldberg, K. I. NMR Chemical Shifts of Trace Impurities: Common Laboratory Solvents, Organics, and Gases in Deuterated Solvents Relevant to the Organometallic Chemist. *Organometallics* **29**, 2176–2179 (2010).
2. Gottlieb, H. E., Kotlyar, V. & Nudelman, A. NMR Chemical Shifts of Common Laboratory Solvents as Trace Impurities. *J. Org. Chem.* **62**, 7512–7515 (1997).
3. Paquette, L. A., Liao, C. C., Burson, R. L., Wingard, R. E., Shih, C. N., Fayos, J. & Clardy, J. Synthesis and electronic properties of 2a,8b-dihydrocyclopent[cd]azulenes (elassovalenes). *J. Am. Chem. Soc.* **99**, 6935–6945 (1977).
4. Vogel, E., Brinker, U. H., Nachtkamp, K., Wassen, J. & Müllen, K. Semibullvalenes as Potential Homoaromatic Compounds. *Angew. Chem. Int. Ed. Engl.* **12**, 758–760 (1973).
5. Vogel, E., Vogel, A., Kübbeler, H.-K. & Sturm, W. 1,6:8,13-Propanediylidene[14]annulene. *Angew. Chem. Int. Ed. Engl.* **9**, 514–516 (1970).
6. Vogel, E. & Reel, H. Bent [14]annulenes. Synthesis of 1,6:8,13-ethanediylidene[14]annulene. *J. Am. Chem. Soc.* **94**, 4388–4389 (1972).
7. Dobrowolski, J. C. Three Queries about the HOMA Index. *ACS Omega* **4**, 18699–18710 (2019).
8. Ostrowski, S. & Dobrowolski, J. C. What does the HOMA index really measure? *RSC Adv.* **4**, 44158–44161 (2014).
9. Krygowski, T. M., Szatylowicz, H., Stasyuk, O. A., Dominikowska, J. & Palusiak, M. Aromaticity from the viewpoint of molecular geometry: application to planar systems. *Chem. Rev.* **114**, 6383–6422 (2014).
10. Quast, H., Seefelder, M., Peters, E.-M. & Peters, K. Synthesis, Crystal Structure, and Circular Dichroism Spectra of (1S)-4,8-Diphenylbarbaralane-2,6-dicarbonitrile – Chiroptical Properties of the Transition State of a Degenerate Cope Rearrangement. *Eur. J. Org. Chem.* **1999**, 1811–1823 (1999).
11. Bianchi, R., Pilati, T. & Simonetta, M. Structure of 1,6-methano[10]annulene. *Acta Cryst. B* **36**, 3146–3148 (1980).
12. Menzek, A., Altundas, A. & Gültekin, D. A new, safe and convenient procedure for reduction of naphthalene and anthracene: synthesis of tetralin in a one-pot reaction. *J. Chem. Res.* **2003**, 752–753 (2003).

13. Yus, M., Herrera, R. P. & Guijarro, A. On the Mechanism of Arene-Catalyzed Lithiation: The Role of Arene Dianions—Naphthalene Radical Anion versus Naphthalene Dianion. *Chem. Eur. J.* **8**, 2574 (2002).

14. Cordi, A. A., Berque-Bestel, I., Persigand, T., Lacoste, J. M., Newman-Tancredi, A., Audinot, V. & Millan, M. J. Potential antidepressants displayed combined alpha(2)-adrenoceptor antagonist and monoamine uptake inhibitor properties. *J. Med. Chem.* **44**, 787–805 (2001).

15. Díaz, E., Reyes, E., Uria, U., Carrillo, L., Tejero, T., Merino, P. & Vicario, J. L. Carboxylates as Nucleophiles in the Enantioselective Ring-Opening of Formylcyclopropanes under Iminium Ion Catalysis. *Chem. Eur. J.* **24**, 8764–8768 (2018).

16. Frisch, M. J., Trucks, G. W., Schlegel, H. B., Scuseria, G. E., Robb, M. A., Cheeseman, J. R., Scalmani, G., Barone, V., Petersson, G. A. & Nakatsuji, H. Gaussian 16. *Wallingford CT* **2** (2016).

17. Chen, Z., Wannere, C. S., Corminboeuf, C., Puchta, R. & Schleyer, P. v. R. Nucleus-independent chemical shifts (NICS) as an aromaticity criterion. *Chem. Rev.* **105**, 3842–3888 (2005).

18. R. Gershoni-Poranne & A. Stanger. NICS - Nucleus-independent chemical shift. In *Aromaticity. Modern computational methods and applications,* edited by I. Fernández (Elsevier, S.l., 2021), pp. 99–154.

19. Stanger, A. Nucleus-independent chemical shifts (NICS): distance dependence and revised criteria for aromaticity and antiaromaticity. *J. Org. Chem.* **71**, 883–893 (2006).

20. Stanger, A. Reexamination of NICSπ,zz: Height Dependence, Off-Center Values, and Integration. *J. Phys. Chem. A* **123**, 3922–3927 (2019).

21. Gershoni-Poranne, R. & Stanger, A. The NICS-XY-scan: identification of local and global ring currents in multi-ring systems. *Chem. Eur. J.* **20**, 5673–5688 (2014).

22. Herges, R. & Geuenich, D. Delocalization of Electrons in Molecules. *J. Phys. Chem. A* **105**, 3214–3220 (2001).

23. Geuenich, D., Hess, K., Köhler, F. & Herges, R. Anisotropy of the induced current density (ACID), a general method to quantify and visualize electronic delocalization. *Chem. Rev.* **105**, 3758–3772 (2005).

24. Chen, Z., Jiao, H., Wu, J. I., Herges, R., Zhang, S. B. & Schleyer, P. v. R. Homobenzene: homoaromaticity and homoantiaromaticity in cycloheptatrienes. *J. Phys. Chem. A* **112**, 10586–10594 (2008).

25. Bruhn, T., Schaumlöffel, A., Hemberger, Y. & Bringmann, G. SpecDis: quantifying the comparison of calculated and experimental electronic circular dichroism spectra. *Chirality* **25**, 243–249 (2013).

26. Wiberg, K. B. The Concept of Strain in Organic Chemistry. *Angew. Chem. Int. Ed. Engl.* **25**, 312–322 (1986).

27. Colwell, C. E., Price, T. W., Stauch, T. & Jasti, R. Strain visualization for strained macrocycles. *Chemical science* **11**, 3923–3930 (2020).

28. Baeyer, A. Ueber Polyacetylenverbindungen. *Ber. Dtsch. Chem. Ges.* **18**, 2269–2281 (1885).

29. *cis*-**11**: CCDC 2128305; **12b**: CCDC 2128302; **13b**: CCDC 2128309; **14a**: CCDC 2128304; **14b**: CCDC 2128306; **15**: CCDC 2128303; **16**: CCDC 2128308; **S9**: CCDC 2128307; **S11**: CCDC 2133482.
